# Enantioselective Total Synthesis of (−)‐Finerenone Using Asymmetric Transfer Hydrogenation

**DOI:** 10.1002/anie.202011256

**Published:** 2020-11-23

**Authors:** Andreas Lerchen, Narasimhulu Gandhamsetty, Elliot H. E. Farrar, Nils Winter, Johannes Platzek, Matthew N. Grayson, Varinder K. Aggarwal

**Affiliations:** ^1^ School of Chemistry University of Bristol Cantock's Close Bristol BS8 1TS UK; ^2^ Department of Chemistry University of Bath Claverton Down Bath BA2 7AY UK; ^3^ Chemical Development Bayer AG 42096 Wuppertal Germany

**Keywords:** enantioselective synthesis, MR antagonists, partial transfer hydrogenation, pharmaceutical molecule

## Abstract

(−)‐Finerenone is a nonsteroidal mineralocorticoid receptor antagonist currently in phase III clinical trials for the treatment of chronic kidney disease in type 2 diabetes. It contains an unusual dihydronaphthyridine core. We report a 6‐step synthesis of (−)‐finerenone, which features an enantioselective partial transfer hydrogenation of a naphthyridine using a chiral phosphoric acid catalyst with a Hantzsch ester. The process is complicated by the fact that the naphthyridine exists as a mixture of two atropisomers that react at different rates and with different selectivities. The intrinsic kinetic resolution was converted into a kinetic dynamic resolution at elevated temperature, which enabled us to obtain (−)‐finerenone in both high yield and high enantioselectivity. DFT calculations have revealed the origin of selectivity.

## Graphical abstract

A short 6‐step enantioselective synthesis of (−)‐finerenone is reported featuring a partial transfer hydrogenation of a naphthyridine ring. The naphthyridine existed in two atropisomeric forms that reacted at different rates and selectivities; however, at elevated temperature kinetic dynamic resolution occurred, enabling (−)‐finerenone to be obtained with high enantioselectivity.

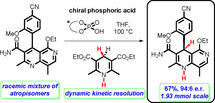

Mortality rates due to heart failure can be markedly reduced by treatment with certain steroids (spironolactone (**1**) and eplerenone (**2**)) which block the mineralocorticoid receptor (MR) (Figure 1).^[1]^ However, their clinical use is limited due to the significant side effects they can cause for example, hyperkalemia and worsening of kidney function^[2]^ and so new MR antagonists are required.^[3]^ As an alternative, it has been reported that 1,4‐dihydropyridines (DHPs), a compound class known for its Ca^2+^ channel modulating activity,^[4]^ can act as MR antagonists in vitro.^[5]^ Through a DHP‐based screening program, Bayer identified finerenone **(−)‐3** (BAY 94‐8862) as a novel, highly potent (IC_50_ 18 nM), nonsteroidal selective mineralocorticoid receptor antagonist.^[6]^ It is currently in phase III clinical trials for the treatment of chronic kidney disease in type 2 diabetes. Finerenone is presently prepared in a racemic form and the active (*S*)‐enantiomer is separated by chiral column chromatography. Although the unwanted (*R*)‐enantiomer can be recycled back to the racemate in two steps,^[7]^ this overall process is wasteful and time‐consuming, especially when conducted on the scales currently required for phase III clinical trials (>1000 kg). Because of these significant short comings, we were interested in developing an enantioselective synthesis of this challenging molecule.

**Figure 1:**
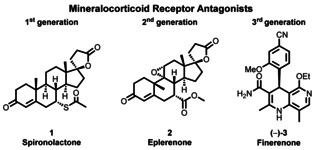
Spironolactone, eplerenone, and finerenone as representative mineralocorticoid receptor antagonists.

There are no asymmetric methods to dihydronaphthyridines, the core structure of finerenone **(−)‐3**, but there are two general methods available to the structurally related dihydroquinolines.[^8^, ^9^, ^10^] These are (i) formal [4+2]‐cyclization reaction between an *ortho*‐quinone methide imine precursor **4** and β‐keto‐amide **5** in the presence of a chiral phosphoric acid catalyst (Figure 2 a)^[8]^ and (ii) site‐selective partial transfer hydrogenation of a naphthyridine **6** using a Hantzsch ester derivative, again in the presence of a chiral phosphoric acid catalyst (Figure 2 b).[^9^, ^11^]

**Figure 2:**
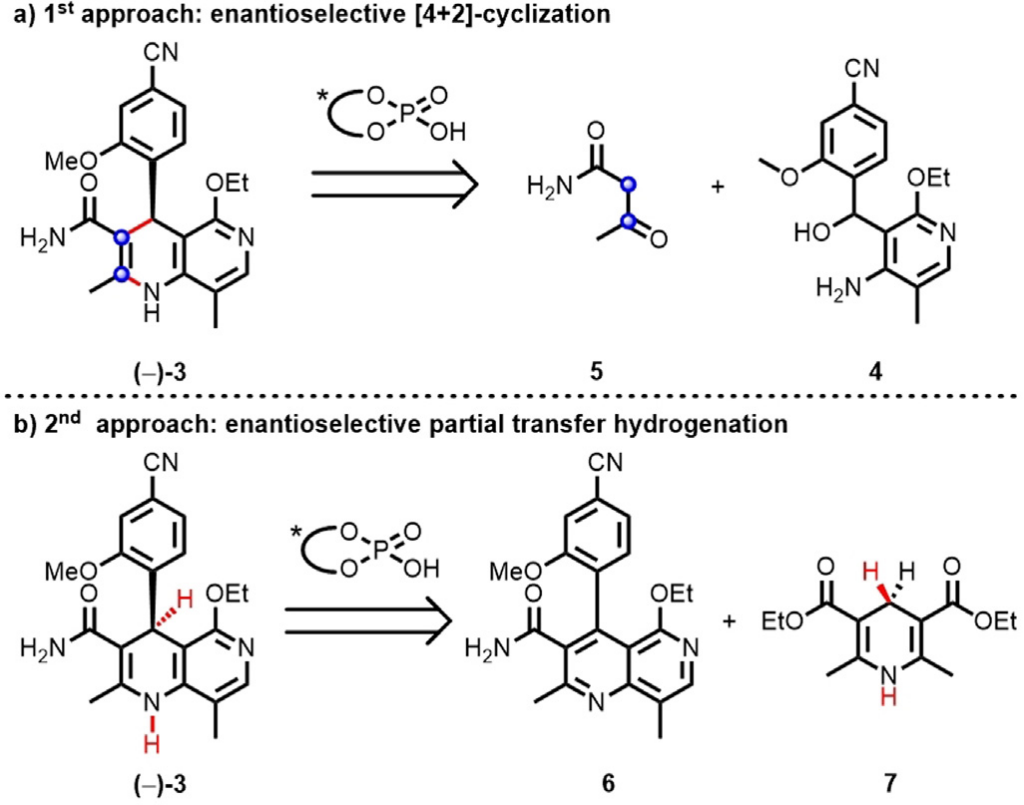
Two strategies for the enantioselective synthesis of finerenone.

We began our studies investigating the formal [4+2]‐cyclization approach for which we required the amino alcohol **4** (Figure 2 a). Starting from commercially available pyridone **8**, selective *O*‐alkylation in the presence of Ag_2_CO_3_ ^[12]^ followed by *N*‐protection using pivaloyl chloride gave amide **9** in 52 % yield over two steps. Directed lithiation of **9** and subsequent trapping with aldehyde **10** led to alcohol **11** in 91 % yield.^[13]^ A brief investigation of this step revealed that a solvent mixture of THF/Et_2_O was necessary together with TMEDA to obtain optimum results. Acid catalyzed deprotection using HCl gave amino alcohol (**4**) in 65 % yield (Scheme 1 a).^[14]^ With the amino alcohol (**4**) in hand, we investigated the enantioselective [4+2]‐cyclization with acetoacetamide **5** using a broad array of chiral phosphoric acids as catalyst (Scheme 1, see the Supporting Information for further details). However, despite an extensive screen of conditions and catalysts, the best result we obtained was 15 % *ee* using 10 mol % (*R*)‐TRIP.

**Scheme 1:**
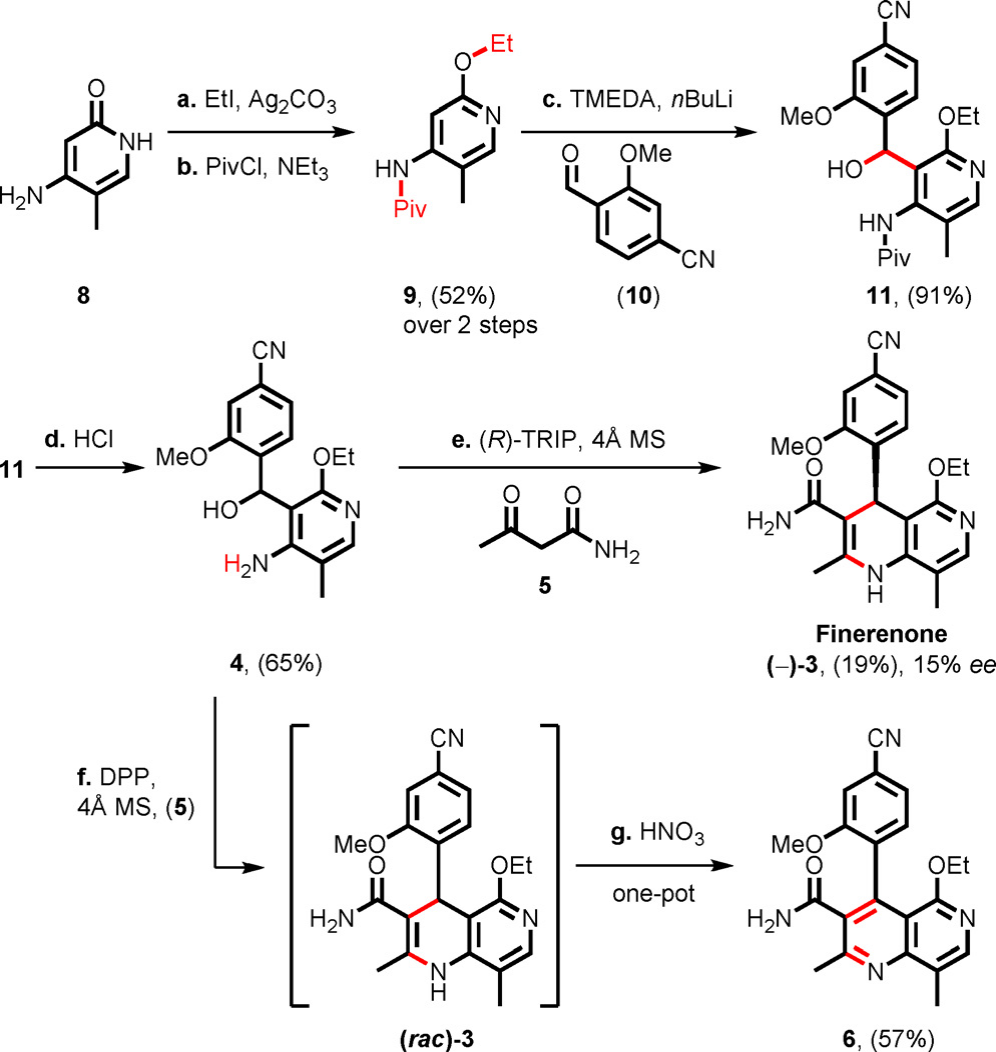
Isolated yields are given. a) Ethyl iodide (2.0 equiv), Ag_2_CO_3_ (2.2 equiv), toluene, 115 °C, 16 hours. b) PivCl (1.5 equiv), NEt_3_ (2.0 equiv), CH_2_Cl_2_, r.t. 48 hours. c) TMEDA (2.5 equiv), *n*BuLi (2.5 equiv), aldehyde (2.5 equiv), Et_2_O/THF, −78 °C to r.t. d) Conc. HCl, dioxane/H_2_O, 83 °C, 3 hours. e) (*R*)‐TRIP (10 mol %), acetoacetamide (3.0 equiv), 4 Å molecular sieves, toluene, 110 °C, 24 hours; *ee* of the product was determined by chiral SFC. f) DPP (20 mol %), acetoacetamide (3.0 equiv), 4 Å molecular sieves, toluene, 110 °C, 24 hours. g) HNO_3_ (1.5 equiv), *n*BuOH, 100 °C, 3 hours. DPP=diphenyl phosphate; (*R*)‐TRIP=((*R)*‐3,3′‐bis(2,4,6‐triisopropylphenyl)‐1,1′‐binaphthyl‐2,2′‐diyl hydrogenphosphate).

As an alternative, we explored the partial transfer hydrogenation reaction for which we required naphthyridine **6** (Figure 2 b). This compound was obtained from alcohol **4** by an acid catalyzed [4+2]‐cyclization with acetoacetamide **5** followed by oxidation with nitric acid in a one pot process in 57 % yield (Scheme 1).^[15]^ With the naphthyridine **6** in hand, we first explored a range of chiral phosphoric acids (5 mol %) for the partial transfer hydrogenation in combination with the Hantzsch ester **7** (Scheme 2 a). Although only 21 % *ee* was obtained using (*R*)‐TRIP (**12 a**) as chiral catalyst (entry 1), other chiral phosphoric acids performed better with the anthracenyl moiety **12 d** proving optimum, giving **(+)‐3** in 42 % yield and 94 % *ee* after 16 hours. Interestingly, in related reductions of quinolines to dihydroquinolines, **12 d** gave essentially racemic products but **12 a**/**b** gave high enantioselectivity.^[9]^ In screening other conditions, we found that the enantioselectivity was remarkably insensitive to solvent effects giving >90 % *ee* for all solvents tested (see the Supporting Information for further details). However, attempts to push the reaction to completion by extending reaction time, changing the concentration, adding molecular sieves or using an excess of catalyst came to no avail; the reaction slowed down considerably at around 50 % yield (Scheme 2 a). This prompted us to re‐isolate the starting material and to explore its properties further. Chiral SFC analysis revealed that the starting material existed in two atropisomeric forms and that the recovered starting material was now enriched in one of the atropisomers, with the ratio increasing with increasing conversion (Scheme 2 a). We were able to determine the relative rate of reaction of the two atropisomers by determining the extent of conversion at short reaction times (4 h, 40 °C, 18 % conversion; see the Supporting Information for further details). This revealed that the (*R*)‐atropisomer reacted ≈30 times faster with the (*R*)‐catalyst than the (*S*)‐atropisomer.

**Scheme 2:**
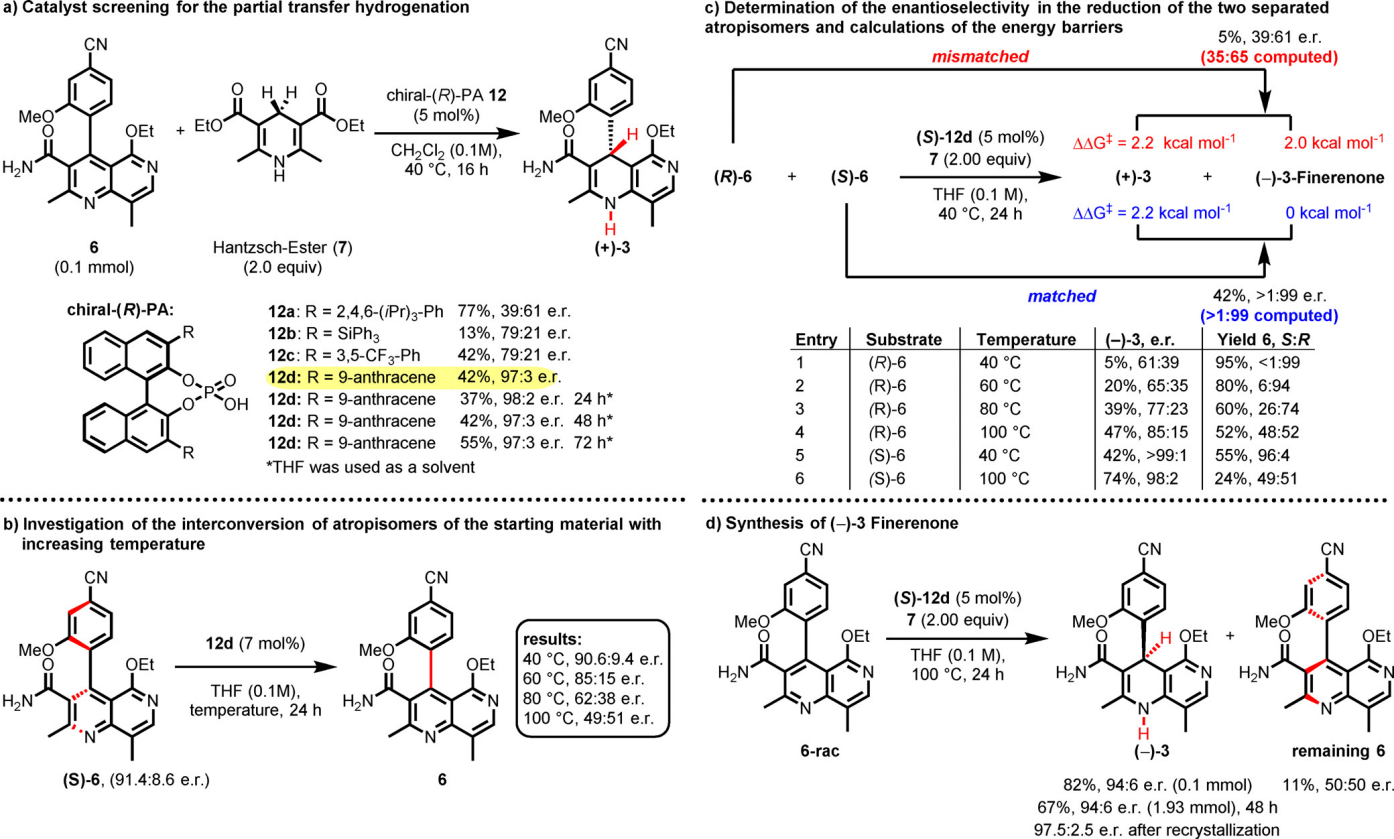
Isolated yields are given. The e.r. of (**3**) and (**6**) was determined by chiral SFC. a) Catalyst screening for partial transfer hydrogenation. b) Investigation of interconversion of atropisomers of starting material with temperature. c) Determination of the enantioselectivity in the reduction of the two separated atropisomers at different temperatures and calculations of the energy barriers. Computed ratios obtained by taking a Boltzmann weighting at 313 K over all conformers within 5 kcal mol^−1^ of the lowest in free energy. d) Optimized conditions for partial transfer hydrogenation.

The thermal stability of the atropisomers was subsequently explored (Scheme 2 b). Heating a 91.4:8.6 ratio of atropisomers at various temperatures for 24 hours showed that racemization was just beginning at 40 °C but was essentially complete at 100 °C.

In order to determine the enantioselectivity in the reduction of the two atropisomers, they were separated by semi‐prep chiral HPLC^[16]^ and reacted with the (*S*)‐catalyst at 40 °C for 24 hours. In the case of the (*S*)‐atropisomer, a 42 % yield of **3** was obtained with remarkably high enantioselectivity (>99:1 e.r., matched) but in the case of the (*R*)‐atropisomer, **3** was obtained in just 5 % yield and 61:39 e.r. (mismatched) (Scheme 2 c).

Since the two atropisomers reacted with markedly different selectivities, we needed to perform a dynamic kinetic resolution where the slow reacting and non‐selective atropisomer isomerized into the fast reacting and selective atropisomer before it reacted. We reasoned that at elevated temperature, the unimolecular racemization process should increase in rate faster than the reduction process which required 3 molecules to come together, due to entropy. We therefore carried out reactions at increasing temperatures (60–100 °C). In the mismatched case [(*R*)‐atropisomer] we were pleased to observe both increasing yields, and increasing enantioselectivity, validating our hypothesis (Scheme 2 c, entries 2–4). It is unusual to observe higher enantioselectivity at higher temperatures, but it occurs here because of the increased rate of isomerization of the atropisomers.

Interestingly, reaction of the matched (*S*)‐atropisomer at 100 °C for 24 hours leads to an increase in yield with only a marginal decrease in enantioselectivity (Scheme 2 c, entries 5,6). After reaction at 100 °C for 24 hours, the recovered starting material was racemic indicating that a dynamic kinetic resolution was occurring (Scheme 2 c, entries 4,6).

This detailed optimization and mechanistic analysis revealed that elevated temperature should provide both high yield and high enantioselectivity. Simply reacting the racemic mixture of atropisomers of **6** at 100 °C for 24 h gave finerenone **(−)**‐**3** in 82 % yield with 94:6 e.r. (Scheme 2 d). On ≈2 mmol scale similar e.r. but a slight reduction in yield was observed.

In order to understand the origin of the selectivity issues encountered we have carried out DFT[^17^, ^18^] calculations (Gaussian 16 Revision A.03,^[19]^ see the Supporting Information for further details) and determined the TSs for reaction of both atropisomers. Note that the Hantzsch ester was simplified to a methyl ester and the ethoxy group on **6** was replaced by a methoxy group. By taking a Boltzmann weighting at 313 K over all conformers within 5 kcal mol^−1^ of the lowest in free energy, an *ee* of 98 % at 40 °C was predicted, in close agreement with the experimental value of 94 % (Scheme 2 a). As summarized in Scheme 2 c, our calculations also match the observed atropisomer selectivities exceptionally closely.

The mechanism of the reaction involves protonation of the naphthyridine ring by the chiral phosphoric acid with simultaneous hydrogen bonding to the Hantzsch ester (Scheme 3). In this assembly concomitant hydride and proton transfer occurs to give the dihydronaphthyridine. The most favored arrangement places the bulk of the naphthyridine ring in open space leading to the major enantiomer (Scheme 3 a). In this TS assembly the (*R*)‐atropisomer is expected to hinder the approach of the incoming Hantzsch ester, accounting for its slow rate of reaction, whilst addition to the (*S*)‐atropisomer is un‐impeded. In the TSs leading to the opposite enantiomer (Scheme 3 b) the catalyst is rotated through 90°, avoiding any steric clash with the naphthyridine ring but at the expense of the strength and directionality of the hydrogen bonds between the catalyst and substrate.

**Scheme 3:**
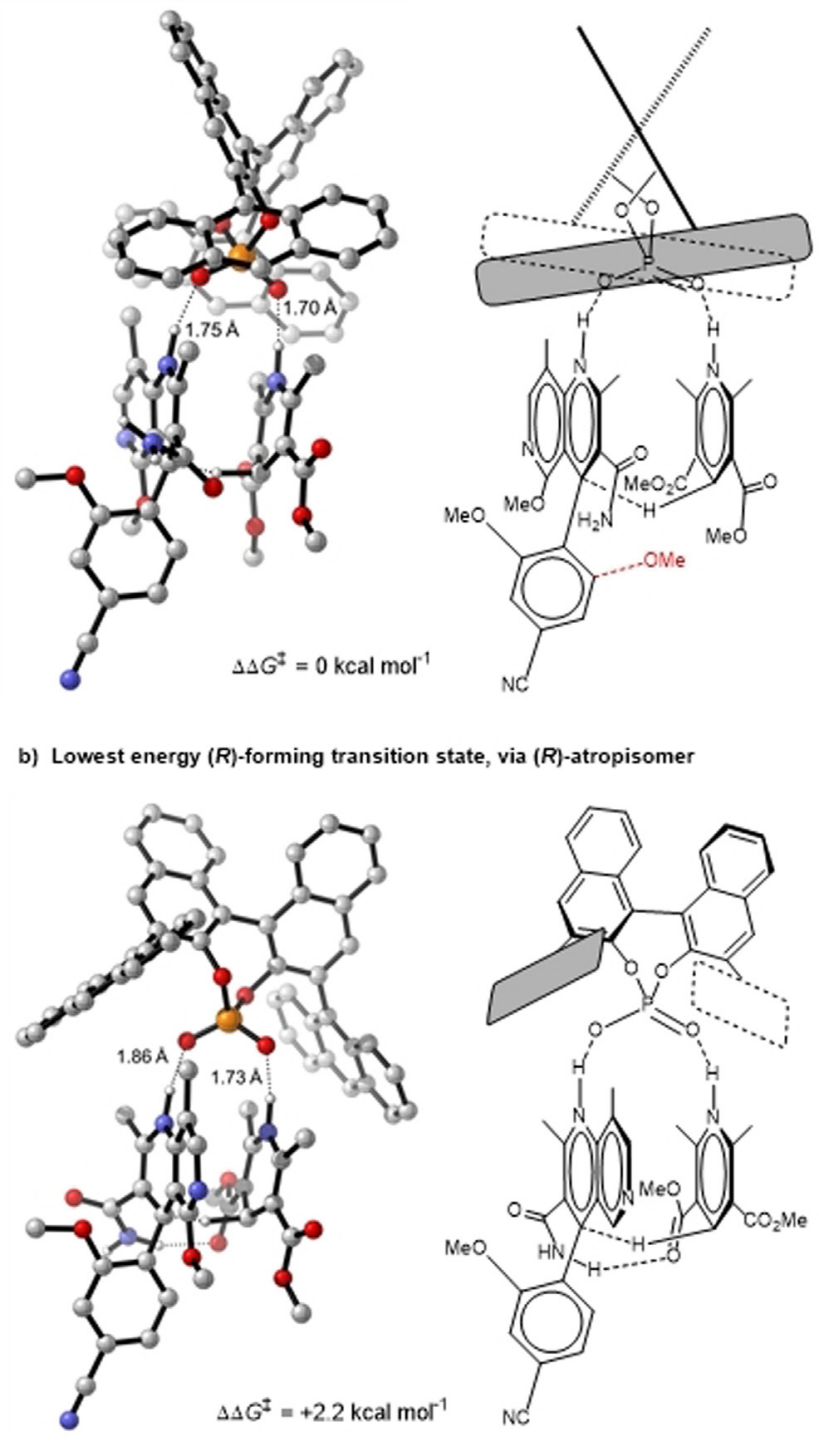
C−H bond‐forming TSs calculated at the M06‐2X/6–311G(d,p)‐SMD(tetrahydrofuran)//B3LYP/6‐31G(d) level of theory. Red indicates position of OMe in reaction of disfavored atropisomer.

In conclusion, we have developed a short 6‐step enantioselective synthesis of finerenone, a mineralocorticoid receptor antagonist candidate in phase III clinical trials. The key step in the synthesis was the partial transfer hydrogenation of naphthyridine **6**, which gave finerenone **(−)‐3** in high yield and with high enantiomeric excess. A detailed mechanistic analysis revealed that naphthyridine **6** existed in two atropisomeric forms that reacted at different rates and with different selectivities, leading to a kinetic resolution. The intrinsic kinetic resolution was transformed into a kinetic dynamic resolution at elevated temperature, enabling (−)‐finerenone to be obtained in both high yield and high enantioselectivity. A model to account for the stereocontrol has been included.

## Conflict of interest

The authors declare no conflict of interest.

## Supporting information

As a service to our authors and readers, this journal provides supporting information supplied by the authors. Such materials are peer reviewed and may be re‐organized for online delivery, but are not copy‐edited or typeset. Technical support issues arising from supporting information (other than missing files) should be addressed to the authors.

Supplementary
