# Supplementary material for: Enantioselective Total Synthesis of (−)‐Finerenone Using Asymmetric Transfer Hydrogenation
Source: Angew Chem Int Ed Engl. 2020 Nov 23;59(51):23107–11. doi: 10.1002/anie.202011256 (PMC7839499; doi:10.1002/anie.202011256)
Supplement: Supplementary file 1 — Supplementary [file ANIE-59-23107-s001.pdf]

Angewandte Chemie
Eine Zeitschrift der Gesellschaft Deutscher Chemiker

Supporting Information

Enantioselective Total Synthesis of (−)-Finerenone Using Asymmetric Transfer Hydrogenation

Andreas Lerchen[+], Narasimhulu Gandhamsetty[+], Elliot H. E. Farrar, Nils Winter, Johannes Platzek, Matthew N. Grayson, and Varinder K. Aggarwal**

anie_202011256_sm_miscellaneous_information.pdf

Contents

1. General Information ... 3

2. Experimental Procedures for the synthesis of the racemic mixture of atropisomers (6) ... 4

3. Optimization of the enantioselective [4+2]-cyclization: synthesis of finerenone (–)-3... 12

4. Optimization for the synthesis of finerenone (–)-3 enabled by the partial transfer hydrogenation ... 14

Catalyst Screen ... 14

Conditions Screen ... 15

Racemization Test of the starting material: ... 17

Full reaction profile of product yield and remaining starting material depending on temperature and reaction time ... 21

Full reaction profile of product yield and remaining starting material depending on temperature using enantiopure starting material ... 22

Rate determination ... 23

5. Synthesis of Finerenone (–)-3 via temperature gradient ... 26

6. Big Scale Synthesis of Finerenone (–)-3 ... 28

7. NMR Spectra ... 31

8. Computational Details ... 38

9. Energies and molecular geometries of all computed structures ... 39

10. References ... 166

1. General Information

Anhydrous solvents were either dried using an Anhydrous Engineering alumina column drying system (THF, toluene, CH_2Cl_2) or obtained as Acroseal bottles and used directly. All other employed solvents were reagent grade solvents and were used directly. Petroleum ether refers to the fraction collected between 40 – 60 °C. Reactions requiring anhydrous conditions (where specified) were conducted under a N_2 / Argon atmosphere using standard Schlenk techniques unless otherwise stated. All reagents were purchased from commercial sources and used as received, unless otherwise stated. Flash column chromatography was carried out using Aldrich silica gel (40-63 μm). All reactions were monitored by thin-layer chromatography (TLC) when practical, using Merck Kieselgel 60 F254 fluorescent treated silica which was visualized under UV light (254 nm) or by staining with an aqueous basic potassium permanganate or *p*-anisaldehyde solution. **^{1}H NMR** spectra were recorded using either Jeol ECS 400 MHz, Bruker 400 MHz, Bruker Cryo 500 MHz, or Varian VNMR (400 MHz or 500 MHz) spectrometers. Chemical shifts (*δ*) are given in parts per million (ppm) and coupling constants (*J*) are given in Hertz (Hz). **^{13}C NMR** spectra were recorded using either Varian VNMR 400 (101 MHz), Bruker 400 MHz or Bruker Cryo 500 (126 Hz) spectrometers. High resolution mass spectra (**HRMS**) were recorded on a Bruker Daltonics Apex IV by Electrospray Ionisation (ESI). **IR** spectra were recorded on a Perkin Elmer Spectrum One FT-IR as a thin film. Only selected absorption maxima (ν_{max}) are reported in wavenumbers (cm^{-1}). Melting points were recorded in degrees Celsius (°C) using a Stuart SMP30 melting point apparatus. Optical rotations (**$[\alpha]^{D}$ T**) were measured on a Bellingham & Stanley Ltd. ADP 220 polarimeter. Chiral supercritical fluid chromatography (**SFC**) was performed on a Waters TharSFC system using a Chiralpak® IB column (4.6 × 250 mm × 5μm) at an oven temperature of 40 °C and was monitored using a diode array detector (DAD). Chiral supercritical fluid chromatography (**SFC**) was performed on an Agilent 1290 Infinity chiral SFC using a Chiralpak® IB column (4.6 × 250 mm × 5μm) or a Chiralpak® IC column (4.6 × 250 mm × 5μm) at an oven temperature of 40 °C and was monitored using a diode array detector (DAD).

2. Experimental Procedures for the synthesis of the racemic mixture of atropisomers (6)

2-ethoxy-5-methylpyridin-4-amine (8-1)

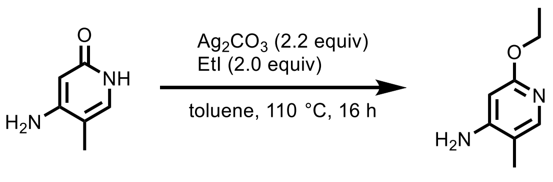

8 **8-1**

4-amino-5-methylpyridin-2(1*H*)-one (**8**) (124,1 mg, 1.00 mmol, 1.00 equiv) and Ag_2CO_3 (607 mg, 2.20 mmol, 2.20 equiv) were added to a flame-dried Young-type pressure tube equipped with a stirring bar. After 3 vacuum/nitrogen cycles toluene (3.3 mL) was added, followed by ethyl iodide (161 µL, 2.00 mmol, 2.00 equiv). The resulting suspension was heated to 110 °C for 16 hours in an oil bath. Afterwards, the reaction was cooled down to room temperature and diluted with dichloromethane (5 mL). The crude mixture was adsorbed on silica and purified via flash column chromatography using pentane/ethylacetate (4:1 to 1:1) as eluent. The desired product 2-ethoxy-5-methylpyridin-4-amine (**8-1**) was obtained as a slightly yellow solid (102 mg, 0.67 mmol, 67%).

M.P.: 76 – 78 °C.

R*f*: 0.20 (pentane/EtOAc, 50:50).

^{1}H NMR (400 MHz, $CDCl_3$) δ 7.67 (s, 1H), 5.94 (s, 1H), 4.26 (q, J = 7.1 Hz, 2H), 4.01 (s, 2H), 2.03 (s, 3H), 1.34 (t, J = 7.0 Hz, 3H).

^{13}C NMR (100 MHz, $CDCl_3$) δ 164.1, 153.6, 146.8, 112.1, 93.5, 61.3, 15.0, 13.5.

HRMS: (ESI) calculated for $C_8H_{13}N_2O$ $[M + H]^+$: 153.1022, found: 153.1015.

IR: (film) ν_{max}/cm^{-1}: 3463, 3343, 3216, 2978, 1634, 1613, 1568, 1498, 1460, 1424, 1387, 1226, 1183, 1041, 1010, 836.

N-(2-Ethoxy-5-methylpyridin-4-yl)pivalamide (9)

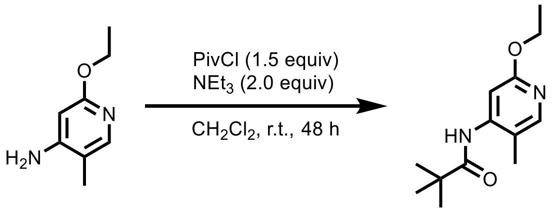

8-1 **9**

To an ice-cooled solution of 2-ethoxy-5-methylpyridin-4-amine (**8-1**, 5.02 g, 33.0 mmol, 1.00 equiv) and triethylamine (6.67 g, 66.0 mmol, 2.00 equiv) in dichloromethane (25 mL) was added a solution of pivaloyl chloride (6.0 g, 49.5 mmol, 1.50 equiv) in dichloromethane (6 mL) dropwise while stirring. Then, the reaction mixture was allowed to warm up to room temperature. After 24 hours, the reaction mixture was quenched by adding water (50 mL). Afterwards, the mixture was extracted with dichloromethane (3x 50 mL). The combined organic layers were washed with saturated aqueous sodium chloride (50 mL), dried over $MgSO_4$, evaporated under reduced pressure, and purified by column chromatography on silica gel (85:15; hexane/EtOAc) to give the corresponding *N*-pivaloylated compound (**9**, 6.0 g, 77%) as a colourless solid.

M.P.: 94 – 95 °C.
R*f*: 0.60 (EtOAc:hexane 30:70).
^{1}H NMR (400 MHz, $CDCl_3$) δ 7.85 (s, 1H), 7.65 (s, 1H), 7.36 (s, 1H), 4.26 (q, *J* = 7.1 Hz, 2H), 2.12 (s, 3H), 1.33 (t, *J* = 7.1 Hz, 3H), 1.30 (s, 9H).
^{13}C NMR (100 MHz, $CDCl_3$) δ 176.8, 164.3, 147.1, 145.3, 114.1, 100.1, 61.8, 40.3, 27.6 (3C), 14.7, 13.6.
HRMS: (ESI) calculated for $C_{13}H_{21}N_2O_2$ $[M + H]^+$: 237.1603, found: 237.1589.
IR: (film) ν_{max}/cm^{-1}: 3463, 3017, 2970, 2943, 1738, 1442, 1365$[M + H]^+$, 1228, 1216, 912.

***N*-(3-[(4-Cyano-2-methoxyphenyl)](hydroxy-methyl)-2-ethoxy-5-methylpyridin-4-yl)pivalamide (11)**

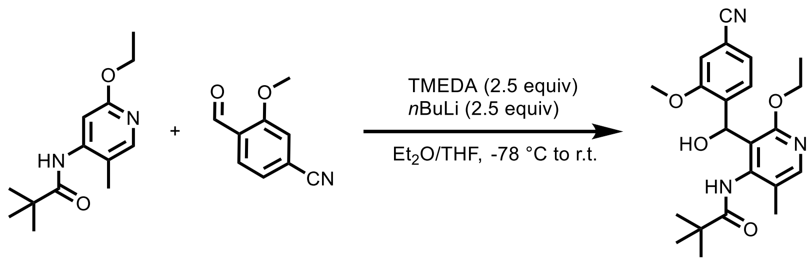

9 **10** **11**

4-Pivaloylaminopyridine derivative (**9**, 2.0 g, 8.47 mmol, 1.00 equiv) was dissolved in a mixture of anhydrous THF (30 mL) and anhydrous diethyl ether (60 mL) under argon atmosphere. The resultant solution was cooled to -78 °C and TMEDA (3.18 mL, 21.2 mmol, 2.50 equiv) and a 1.6 M solution of *n*-butyllithium in hexane (13.2 mL, 21.2 mmol, 2.50 equiv) were added dropwise leading simultaneously to a yellow colored solution. The resulting mixture was then stirred for 30 minutes at -78 °C and for 3 hours at room temperature giving a pale-yellow colored solution. A solution of aldehyde (**10**, 3.41 g, 21.2 mmol, 2.50 equiv) in anhydrous THF (30 mL) was added dropwise to the stirred solution at -78 °C and the stirring was continued for further 30 minutes. Then, the light green colored solution was warmed up to 0 °C, stirred for 2 hours leading to a yellow colored solution. The mixture was then hydrolyzed with saturated ammonium chloride (25 mL) at -78 °C and extracted with EtOAc (3x 25 mL). The organic phase was washed with saturated aqueous sodium chloride, dried over magnesium sulfate ($MgSO_4$), evaporated and purified by a column chromatography on silica (70:30; hexane/EtOAc) to obtain the product **11** (3.08 g, 91%) as a colourless solid.

M.P.: 168 – 170 °C.
R*f*: 0.15 (hexane/EtOAc, 70:30).
^{1}H NMR (400 MHz, $CDCl_3$) δ 8.72 (s, 1H), 7.96 (s, 1H), 7.13 (d, *J* = 7.7 Hz, 1H), 7.12 (s, 1H), 7.00 (d, *J* = 7.7 Hz, 1H), 6.48 (d, *J* = 4.1 Hz, 1H), 4.30 (d, *J* = 4.2 Hz, 1H), 4.27 (q, *J* = 7.1 Hz, 2H), 3.94 (s, 3H), 2.08 (s, 3H), 1.24 (t, *J* = 7.0 Hz, 3H), 0.99 (s, 9H).
^{13}C NMR (100 MHz, $CDCl_3$) δ 176.1, 159.8, 157.4, 147.9, 146.2, 134.5, 128.7, 125.1, 124.2, 118.6, 113.9, 113.4, 112.5, 65.8, 62.1, 56.2, 39.3, 27.3 (3C), 15.4, 14.6.
HRMS: (ESI) calculated for $C_{22}H_{27}N_3NaO_4$ $[M + Na]^+$: 420.1899; found: 420.1905.
IR: (film) ν_{max}/cm^{-1}: 3331, 2969, 2869, 2230, 1737, 1670, 1600, 1572, 1473, 1378, 1215, 1033, 929.

4-((4-Amino-2-ethoxy-5-methylpyridin-3-yl)(hydroxy)methyl)-3-methoxybenzonitrile (4)

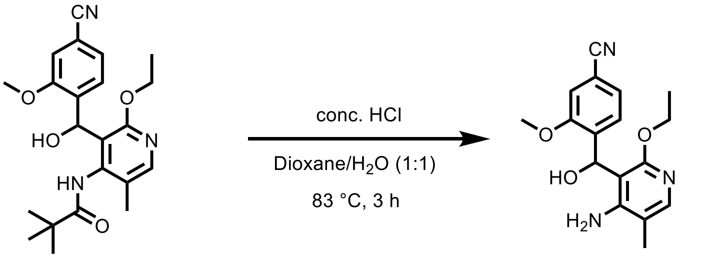

The compound **11** (2.38 g, 6.00 mmol) was dissolved in 1,4-dioxane (36.0 mL) and water (36.0 mL). Concentrated HCl (24.0 mL) was added dropwise simultaneously to the stirred solution. Then, the resulting mixture was heated to 83 °C for 3 hours and the reaction progress was monitored by TLC (hexane/EtOAc; 1:1). After cooling to 0 °C, 2 N NaOH was added slowly to neutralize the reaction mixture followed by extraction with ethyl acetate. The organic layer was washed with brine and dried over $MgSO_4$. After filtration and evaporation of the solvent under reduced pressure, the resulting crude residue was purified by silica gel column chromatography (60:40; hexane/EtOAc) to give the desired compound (**4**, 1.23 g, 65.5%) as a colourless solid.

M.P.: 144 – 146 °C.

R*f*: 0.30 (hexane/EtOAc, 50:50).

^{1}H NMR (500 MHz, $CDCl_3$) δ 7.66 (s, 1H), 7.19 (s, 2H), 7.14 (s, 1H), 6.50 (s, 1H), 4.84 (s, 2H), 4.32 – 4.17 (m, 2H), 3.99 – 3.91 (m, 1H), 3.95 (s, 3H), 2.05 (s, 3H), 1.25 (t, J = 7.1 Hz, 3H).

^{13}C NMR (125 MHz, $CDCl_3$) δ 160.5, 157.4, 152.7, 145.5, 135.5, 128.6, 125.1, 118.8, 113.6, 113.0, 112.3, 102.3, 66.1, 61.5, 56.1, 14.8, 13.8.

HRMS: (ESI) calculated for $C_{17}H_{20}N_3O_3$ [M + H]$^+$: 314.1505, found: 314.1501.

IR: (film) ν_{max}/cm^{-1}: 3477, 3371, 2972, 2934, 2229, 1710, 1571, 1405, 1218, 1112, 1032, 933.

4-(4-Cyano-2-methoxyphenyl)-5-ethoxy-2,8-dimethyl-1,6-naphthyridine-3-carboxamide (6)

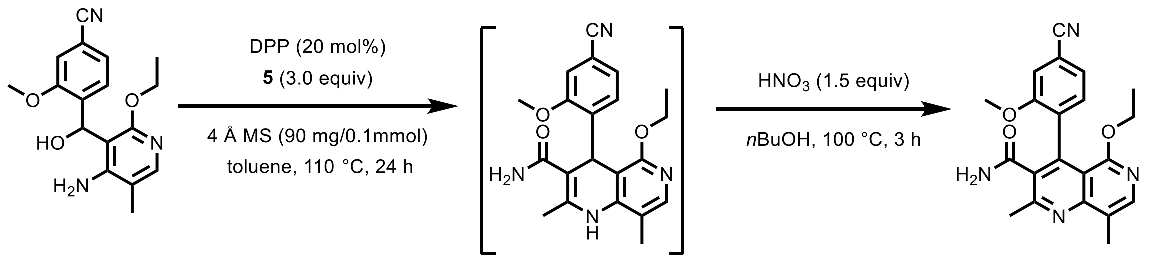

4 **(rac)-3** **6**

4-[(4-Amino-2-ethoxy-5-methylpyridin-3-yl) hydroxymethyl]-3-methoxybenzonitrile **4** (31.3 mg, 0.10 mmol; 1.00 equiv), acetoacetamide (**5**, 30.3 mg, 0.30 mmol; 3.00 equiv), diphenyl phosphate (DPP, 5 mg, 20 mol%) and flame dried 4 Å molecular sieves (90.0 mg/0.10 mmol) were loaded into an oven dried microwave vial and suspended in anhydrous toluene (1.00 mL) under an argon atmosphere. The vial was closed with a sealed cap. The reaction mixture was stirred at 110 °C for 24 hours and the reaction progress was monitored by TLC (hexane/EtOAc; 2:8). Then, the reaction mixture was cooled down to 0 °C and diluted with *n*-butanol (1.5 mL). Then, 90% concentrated HNO_3 (0.07 mL, 0.15 mmol, 1.50 equiv) was slowly added and the mixture was heated to react at 100 °C. After 3 hours, the reaction mixture was cooled down to 0 °C and quenched with saturated sodium bicarbonate (1 mL). Next, the reaction mixture was extracted with EtOAc (3x 3 mL). The combined organic layers were dried over $MgSO_4$, evaporated under reduced pressure, and purified by column chromatography on silica gel (50:50; hexane/EtOAc) to give **6** (21.4 mg, 57%) as a pale yellow solid.

M.P.: 245 – 246 °C.
$\mathbf{R}_f$: 0.27 (pentane/EtOAc, 50:50).
^{1}**H NMR** (500 MHz, Methanol-*d*4) *δ* 8.00 (s, 1H), 7.43 – 7.34 (m, 2H), 7.31 (d, *J* = 8.0 Hz, 1H), 4.16 – 3.96 (m, 2H), 3.74 (s, 3H), 2.81 (s, 3H), 2.58 (s, 3H), 0.81 (t, *J* = 7.1 Hz, 3H).
^{1}**H NMR** (400 MHz, $CDCl_3$) δ 8.03 (d, *J* = 1.2 Hz, 1H), 7.35 (dd, *J* = 7.7, 1.3 Hz, 1H), 7.21 (d, *J* = 7.7 Hz, 1H), 7.16 (d, *J* = 1.3 Hz, 1H), 5.63 (s, 1H), 5.45 (s, 1H), 4.17 – 3.95 (m, 2H), 3.74 (s, 3H), 2.83 (s, 3H), 2.58 (d, *J* = 1.0 Hz, 3H), 0.77 (t, *J* = 7.1 Hz, 3H).
^{13}**C NMR** (125 MHz, Methanol-*d*4) *δ* 172.2, 161.0, 159.5, 158.9, 153.1, 143.5, 142.0, 134.7, 133.2, 131.4, 124.9, 124.5, 119.7, 114.3, 113.7, 112.3, 62.9, 56.4, 23.7, 14.6, 13.8.
HRMS: (ESI) calculated for $C_{21}H_{21}N_4O_3$ $[M + H]^+$: 377.1608, found: 377.1598.
IR: (film) ν_{max}/cm^{-1}: 3336, 3181, 2980, 2231, 1668, 1602, 1568, 1508, 1480, 1440, 1406, 1383, 1326, 1307, 1195, 1120, 1034, 927, 853, 813.
Enantiomeric ratio of 50:50 was determined using chiral SFC analysis (IC, 30% MeOH, 3 mL/min, 125 bar): t_R = 2.5 [(*S*)], 2.8 [(*R*)].

Racemate of (**6**)

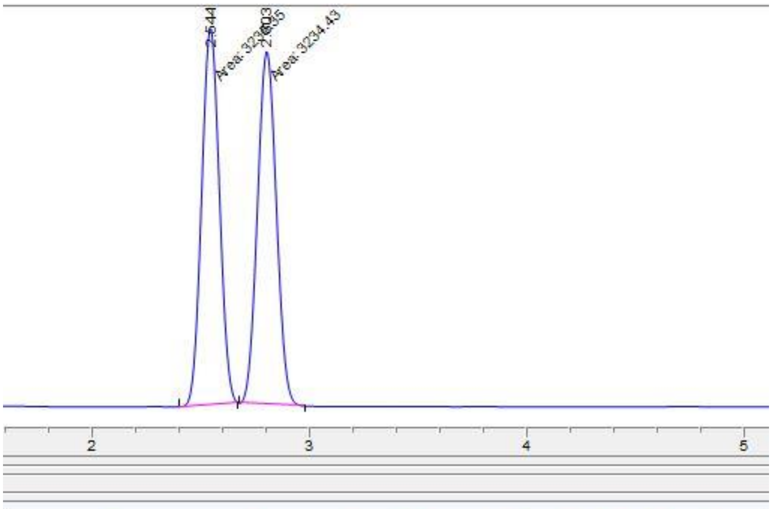

#	Time	Area	Height	Width	Area%	Symmetry
1	2.544	3235.3	572.2	0.0942	50.007	0.958
2	2.803	3234.4	535.9	0.1006	49.993	0.946

Pure (*R*)-atropisomer

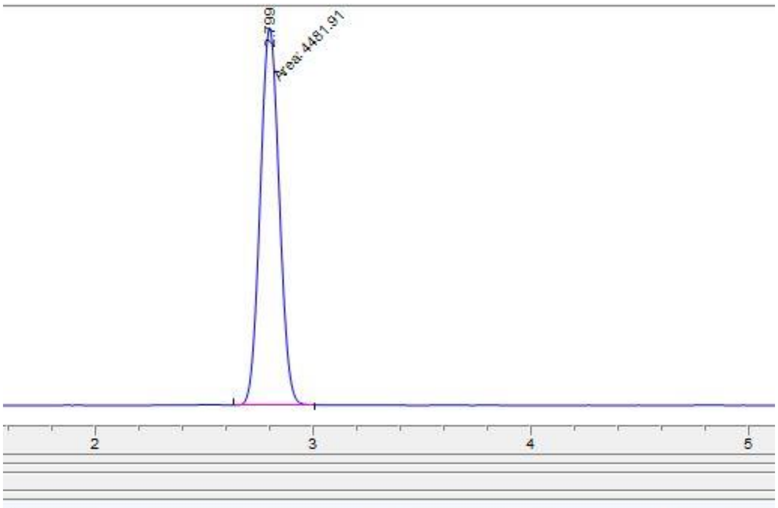

#	Time	Area	Height	Width	Area%	Symmetry
1	2.799	4481.9	741	0.1008	100.000	0.946

Pure (*S*)-atropisomer

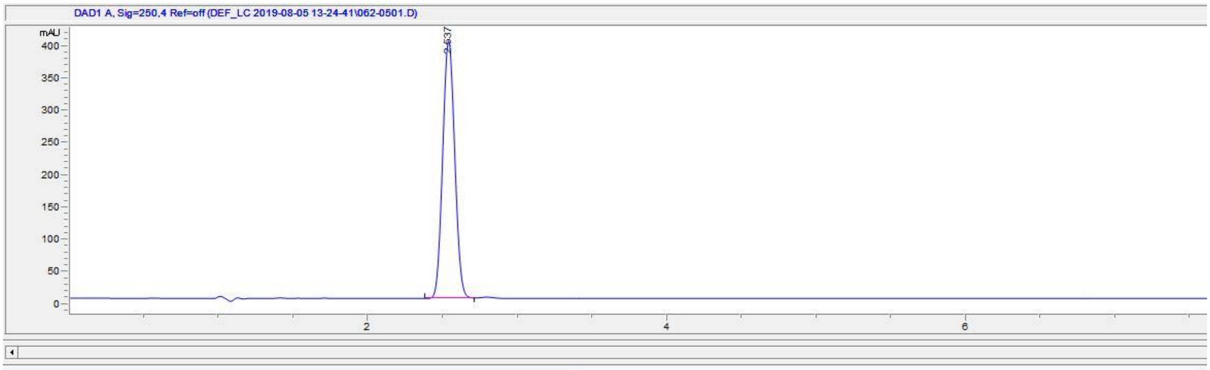

#	Time	Area	Height	Width	Area%	Symmetry
1	2.537	2243.5	400.4	0.0894	100.000	0.919

CD-Spectrum of (*R*)-atropisomer

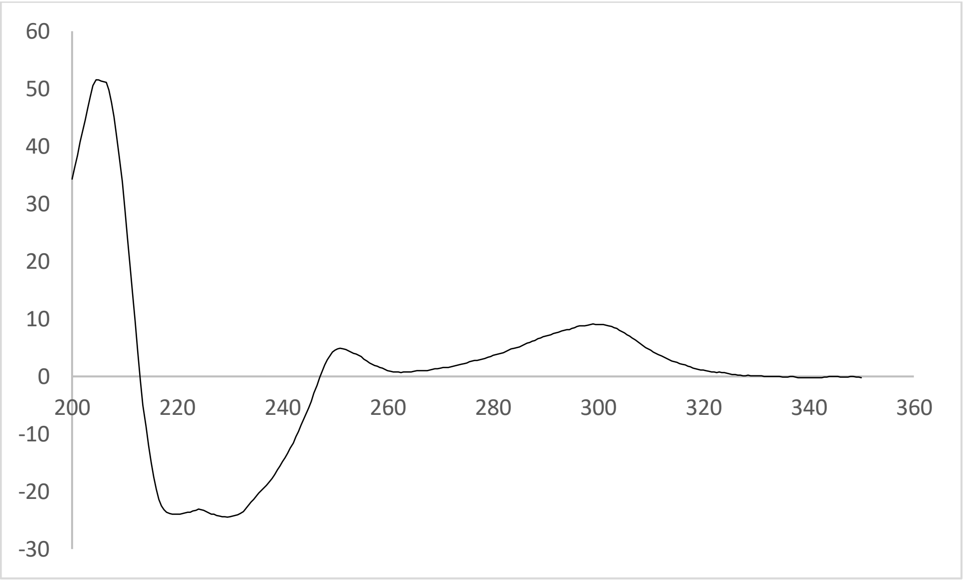

CD-Spectrum of (*S*)-atropisomer

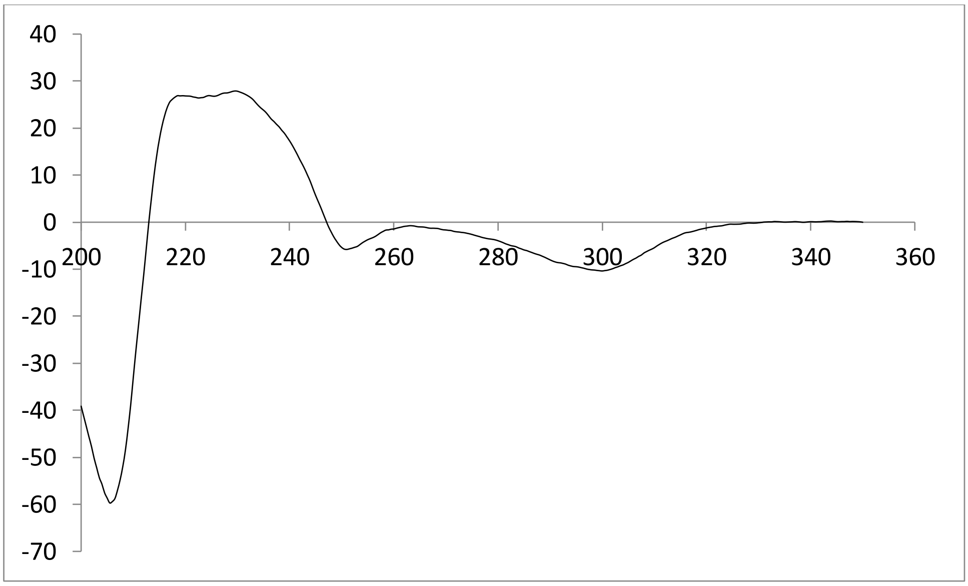

3. Optimization of the enantioselective [4+2]-cyclization: synthesis of finerenone (–)-3

(*S*)-4-(4-cyano-2-methoxyphenyl)-5-ethoxy-2,8-dimethyl-1,4-dihydro-1,6-naphthyridine-3-carboxamide; Finerenone (–)-3

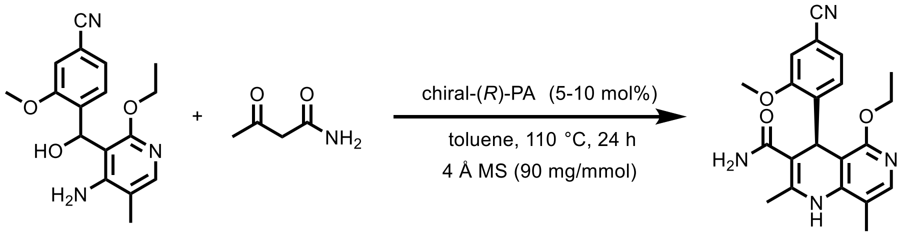

The compound **4** (31.3 mg, 0.10 mmol, 1.0 equiv), acetoacetamide (**5**, 30.3 mg, 0.30 mmol, 3.0 equiv), chiral-(*R*)-PA (5-10 mol%) and flame dried 4Å molecular sieves (90.0 mg/0.10 mmol) were loaded into an oven dried microwave vial and suspended in anhydrous toluene (1.00 mL) under an argon atmosphere. The vial was closed with a sealed cap. The reaction mixture was stirred at 110 °C for 24 hours and the reaction progress was monitored by TLC (hexane/EtOAc; 2:8). Then, the reaction mixture was quenched with saturated sodium bicarbonate (1 mL) and the reaction mixture was extracted with EtOAc (3x 3mL). The combined organic layers were dried over $MgSO_4$, evaporated under reduced pressure, and purified by column chromatography on silica gel (80:20; hexane/EtOAc to 98:2; EtOAc/methanol) to obtain the cyclized product **(–)-3** as a white solid.

R_f: 0.15 (hexane/EtOAc, 20:80).

^{1}H NMR (400 MHz, DMSO-d_6) δ 7.55 (s, 1H), 7.36 (s, 1H), 7.27 (d, J = 7.9 Hz, 1H), 7.14 (d, J = 7.8 Hz, 1H), 5.37 (s, 1H), 4.07 – 3.91 (m, 2H), 3.82 (s, 3H), 2.18 (s, 3H), 2.11 (s, 3H), 1.04 (t, J = 7.0 Hz, 3H).

^{13}C NMR (125 MHz, DMSO-d_6) δ 169.6, 159.3, 155.6, 144.2, 144.1, 141.6, 137.9, 130.8, 124.7, 118.9, 114.1, 111.3, 109.4, 105.3, 103.1, 60.5, 56.0, 32.4, 17.9, 14.2, 13.7.

HRMS: (ESI) calculated for $C_{21}H_{23}N_4O_3$ $[M + H]^+$: 379.1770, found: 379.1779.

Optimization table: Synthesis of (*S*)-4-(4-cyano-2-methoxyphenyl)-5-ethoxy-2,8-dimethyl-1,4-dihydro-1,6-naphthyridine-3-carboxamide; Finerenone (–)-3

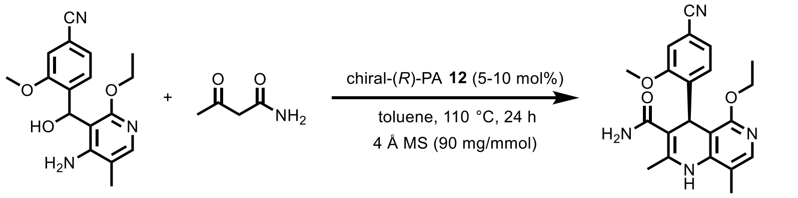

4 5 (–)-3

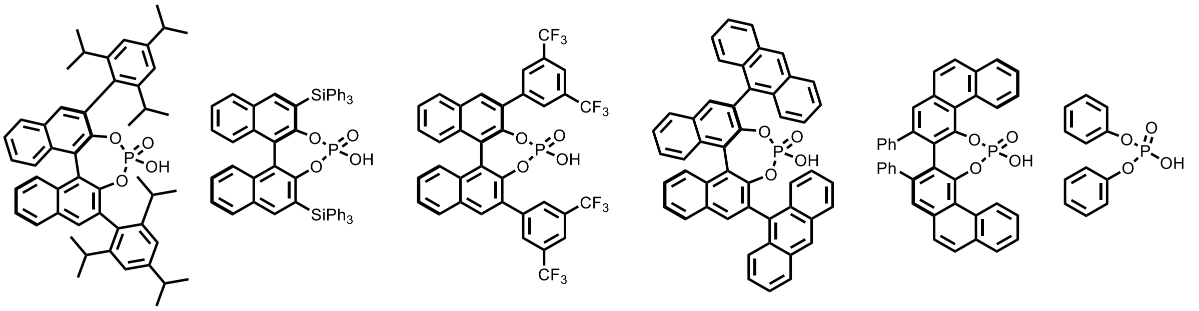

12a, (*R*)-TRIP **12b**, (*R*)-TIPSY **12c**, (*R*)-$(CF_3)_2$ **12d**, (*R*)-anthracene **12e**, (*R*)-VAPOL **12f**, DPP catalyst

entry	catalyst	isolated yield	*ee* (%)
1	12a	19%	15
2	12b	67%	7
3	12c	43%	2
4	12d	32%	2
5	12e	42%	3
6[a)]	12f	61%	0
7[b)]	12a	13%	9

ee was determined by chiral SFC (IB, MeOH 20%).
[a)] catalyst loading 20 mol%. [b)] catalyst loading 5 mol%, reaction time 20 hours.

4. Optimization for the synthesis of finerenone (–)-3 enabled by the partial transfer hydrogenation

Catalyst Screen

General procedure for the catalyst screen:

4-(4-Cyano-2-methoxyphenyl)-5-ethoxy-2,8-dimethyl-1,6-naphthyridine-3-carboxamide (**6**, 37.6 mg, 0.1 mmol, 1.00 equiv), the Hantzsch ester (**7**, 50.7 mg, 0.2 mmol, 2.00 equiv) and the chiral phosphoric acid catalyst **12** (5 mol%) were added in a flame-dried Young-type pressure tube equipped with a stirring bar. After 3 vacuum/nitrogen cycles dry CH_2Cl_2 (1 mL, 0.1M) was added to the tube. Finally, the reaction was stirred at 40 °C in an oil bath for 16 hours. Then, the reaction was cooled to room temperature and diluted with CH_2Cl_2 (5 mL). The organic solvents were evaporated and finally, the crude material was purified via flash column chromatography (eluent EtOAc). **(–)-3** or **(+)-3** were always isolated as a white solid.

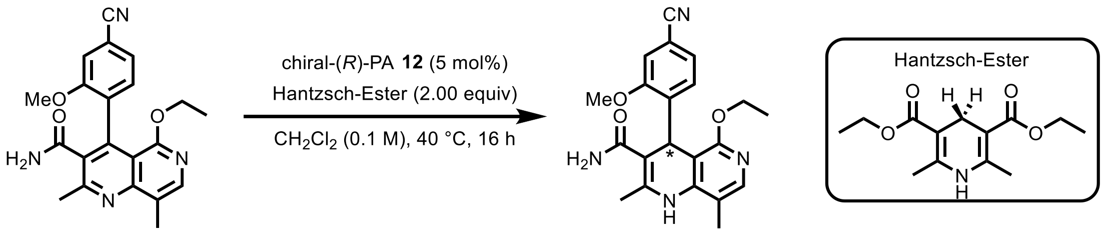

6, 0.1 mmol

3

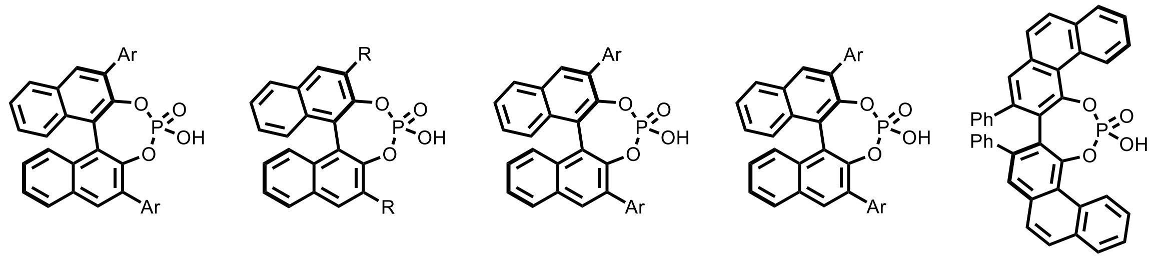

12a, Ar = 2,4,6-(iPr)$_3$C$_6$H$_2$ **12b**, R = SiPh$_3$ **12c**, Ar = 3,5-(CF$_3$)$_2$C$_6$H$_3$ **12d**, Ar = 9-anthracene **12e**

entry	catalyst	isolated yield	*ee* (%)
1	12a	77%	-21
2	12b	13%	58
4	12c	42%	58
**5**	**12d**	**42%**	**93**
3	12e	13%	22

ee was determined by chiral SFC (IB, MeOH 20%).

Conditions Screen

General procedure for the conditions screen:

4-(4-Cyano-2-methoxyphenyl)-5-ethoxy-2,8-dimethyl-1,6-naphthyridine-3-carboxamide (**6**, 37.6 mg, 0.1 mmol, 1.00 equiv), the Hantzsch ester (**7**, 50.7 mg, 0.2 mmol, 2.00 equiv) and the chiral phosphoric acid catalyst **12d** (3.5 mg, 5 mol%) were added in a flame-dried Young-type pressure tube equipped with a stirring bar. After 3 vacuum/nitrogen cycles the dry solvent (1 mL, 0.1M) was added to the tube. Finally, the reaction was stirred at the stated temperature in an oil bath for the stated time. Then, the reaction was cooled to room temperature and diluted with CH_2Cl_2 (5 mL). The organic solvents were evaporated and finally, the crude material was purified via flash column chromatography (eluent EtOAc). **(+)-3** was always isolated as a white solid.

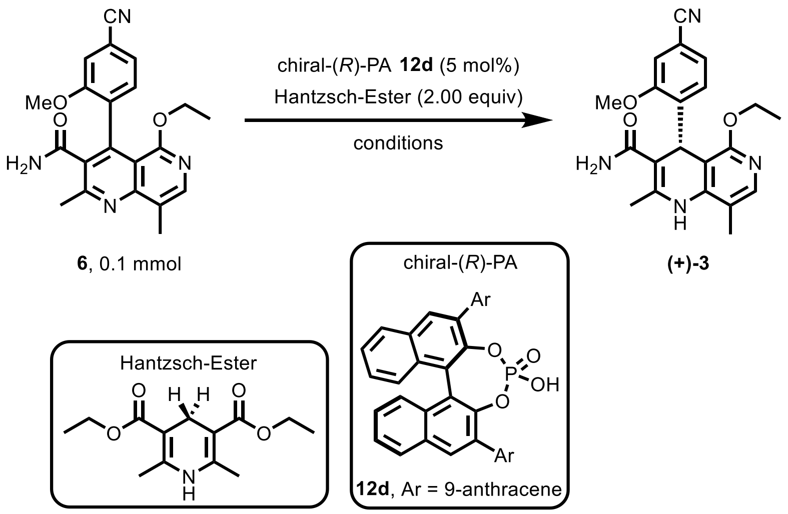

entry	Solvent (0.1 M)	temperature	time	isolated yield (+)-3	*ee* (%)
1	CH_2Cl_2	40 °C	16 h	42%	93
2	THF	40 °C	16 h	29%	98
3[(a)]	THF	40 °C	16 h	21%	98
4	THF	40 °C	60 h	51%	96
5	CH_2Cl_2	50 °C	24 h	53%	90
6	THF	50 °C	24 h	56%	93
7	2-MeTHF	50 °C	24 h	41%	95
8	MeCN	50 °C	24 h	33%	93
9	DCE	50 °C	24 h	55%	90
10	Et_2O	50 °C	24 h	34%	94
11	CF_3-Ph	50 °C	24 h	low conv.	---
**12**	**THF**	**50 °C**	**48 h**	**61%**	**93**
13	CH_2Cl_2	50 °C	48 h	58%	89
**14**	**THF**	**60 °C**	**24 h**	**66%**	**92**
15[(b)]	THF	60 °C	24 h	49%	91

[(a)] reaction concentration 0.2M. [(b)] reaction performed with 4Å molecular sieves (20 mg).
ee was determined by chiral SFC (IB, MeOH 20%).

Racemization Test of the starting material:

General procedure for the investigation of the racemization of starting material:

The enantioenriched starting material 4-(4-Cyano-2-methoxyphenyl)-5-ethoxy-2,8-dimethyl-1,6-naphthyridine-3-carboxamide (**6**, 7 mg, 0.02 mmol, 1.00 equiv, 91.4:8.6 *e.r.*) and the chiral phosphoric acid catalyst **12d** (1 mg, 7 mol%) were added in a flame-dried Young-type pressure tube equipped with a stirring bar. After 3 vacuum/nitrogen cycles dry THF (0.5 mL) was added to the tube. Finally, the reaction was stirred at the stated temperature in an oil bath for 24 hours. Then, the reaction was cooled to room temperature and diluted with CH_2Cl_2 (5 mL). The organic solvents were evaporated and finally, the crude material directly subjected for analysis on the chiral SFC.

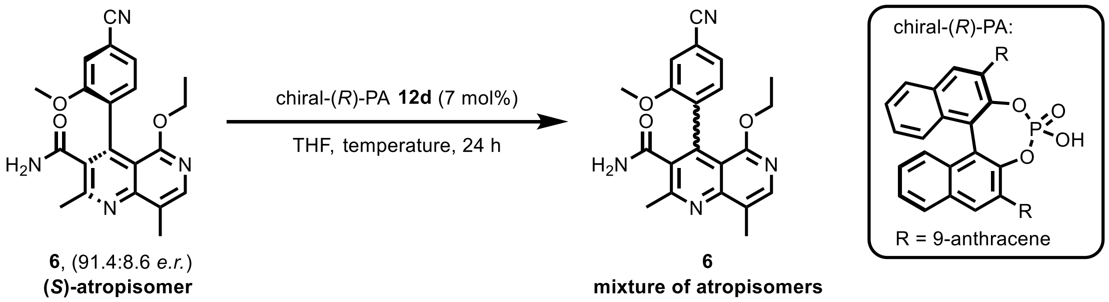

entry	temperature	*e.r.*
1	40 °C	90.6:9.4
2	60 °C	85:15
3	80 °C	62:38
4[(a)]	100 °C	49:51

[(a)] a 86:14 mixture of (**6**) was used. *ee* was determined by chiral SFC (IC, MeOH 30%).

Racemate of (**6**)

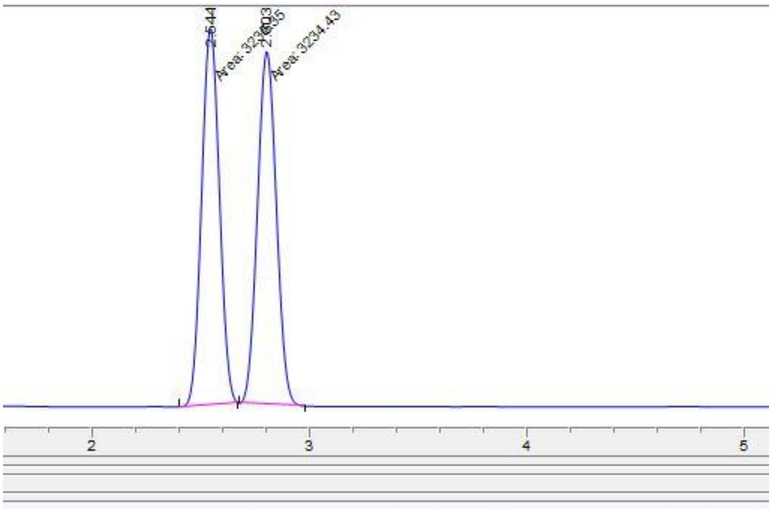

#	Time	Area	Height	Width	Area%	Symmetry
1	2.544	3235.3	572.2	0.0942	50.007	0.958
2	2.803	3234.4	535.9	0.1006	49.993	0.946

Enantioenriched SM of (**6**) used as starting material

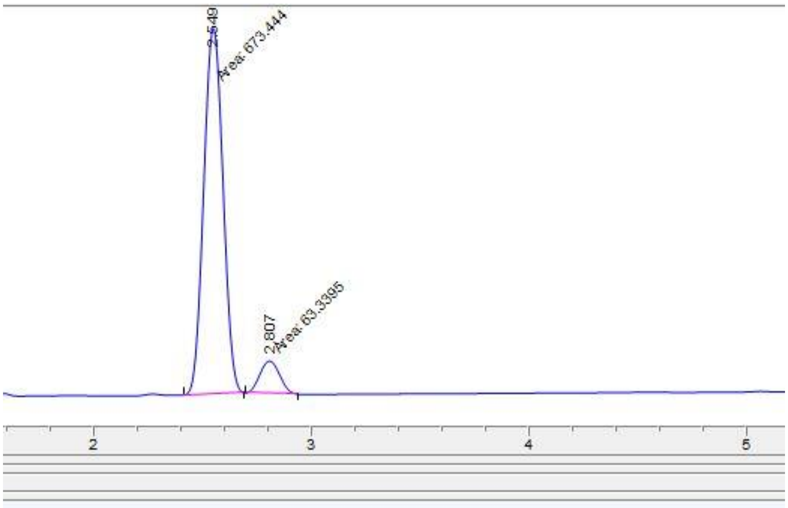

#	Time	Area	Height	Width	Area%	Symmetry
1	2.549	673.4	109.8	0.1023	91.403	0.96
2	2.807	63.3	9.9	0.107	8.597	0.925

Reaction at 40 °C

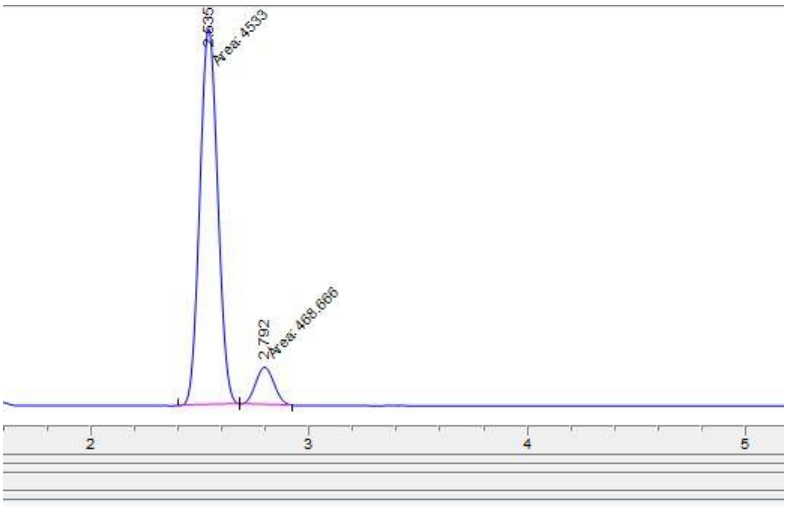

#	Time	Area	Height	Width	Area%	Symmetry
1	2.535	4533	795.1	0.095	90.630	0.939
2	2.792	468.7	79.1	0.0987	9.370	0.927

Reaction at 60 °C

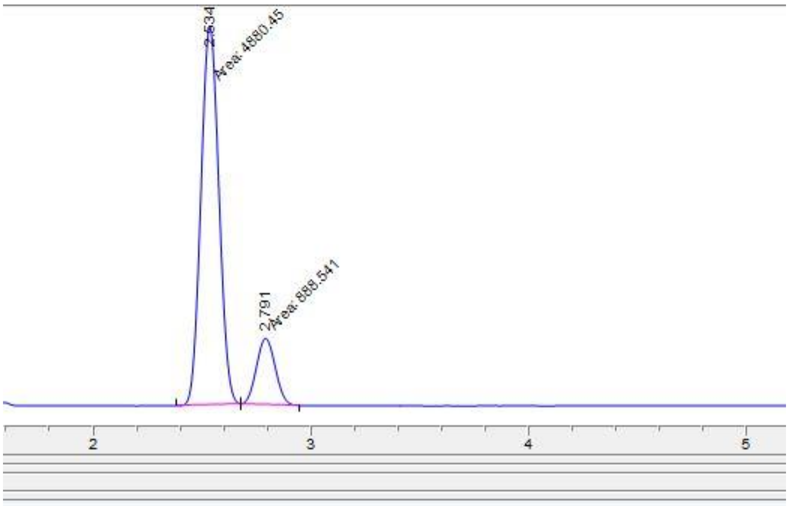

#	Time	Area	Height	Width	Area%	Symmetry
1	2.534	4880.4	852	0.0955	84.598	0.94
2	2.791	888.5	148.8	0.0995	15.402	0.927

Reaction at 80 °C

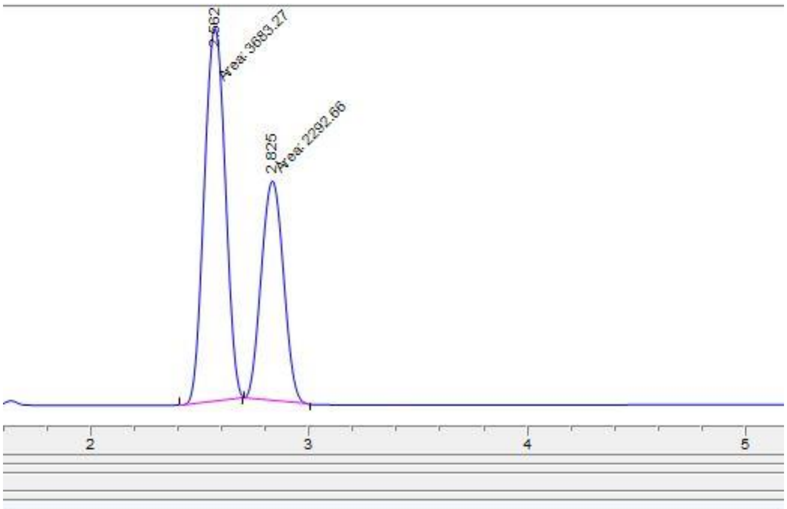

#	Time	Area	Height	Width	Area%	Symmetry
1	2.562	3683.3	551	0.1114	61.635	1.001
2	2.825	2292.7	324.4	0.1178	38.365	0.969

Reaction at 100 °C

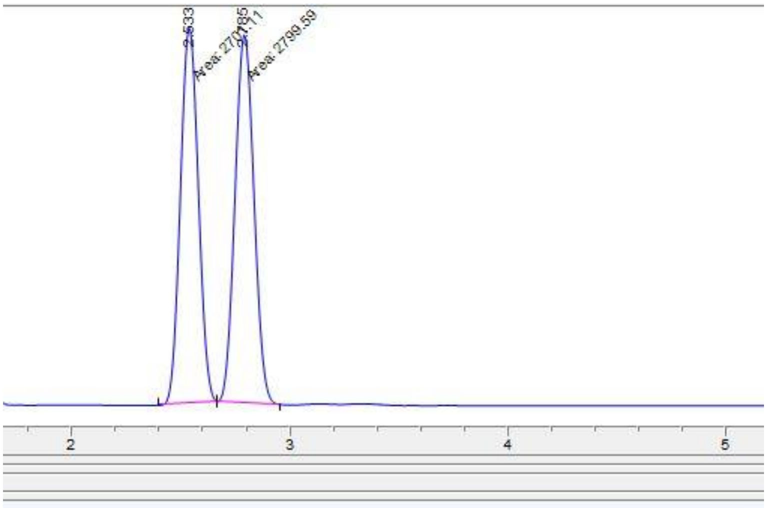

#	Time	Area	Height	Width	Area%	Symmetry
1	2.533	2701.1	474.9	0.0948	49.105	0.948
2	2.785	2799.6	464.6	0.1004	50.895	0.936

Full reaction profile of product yield and remaining starting material depending on temperature and reaction time

General procedure:

4-(4-Cyano-2-methoxyphenyl)-5-ethoxy-2,8-dimethyl-1,6-naphthyridine-3-carboxamide (**6**, 37.6 mg, 0.1 mmol, 1.00 equiv), the Hantzsch ester (**7**, 50.7 mg, 0.2 mmol, 2.00 equiv) and the chiral phosphoric acid catalyst **12d** (3.5 mg, 5 mol%) were added in a flame-dried Young-type pressure tube equipped with a stirring bar. After 3 vacuum/nitrogen cycles dry THF (1 mL, 0.1M) was added to the tube. Finally, the reaction was stirred at the indicated temperature in an oil bath for the stated time. Then, the reaction was cooled to room temperature and diluted with CH_2Cl_2 (5 mL). The organic solvents were evaporated and finally, the crude material was purified via flash column chromatography (eluent EtOAc). The remaining starting material (**6**) was always isolated as a white solid. **(+)-3** was always isolated as a white solid.

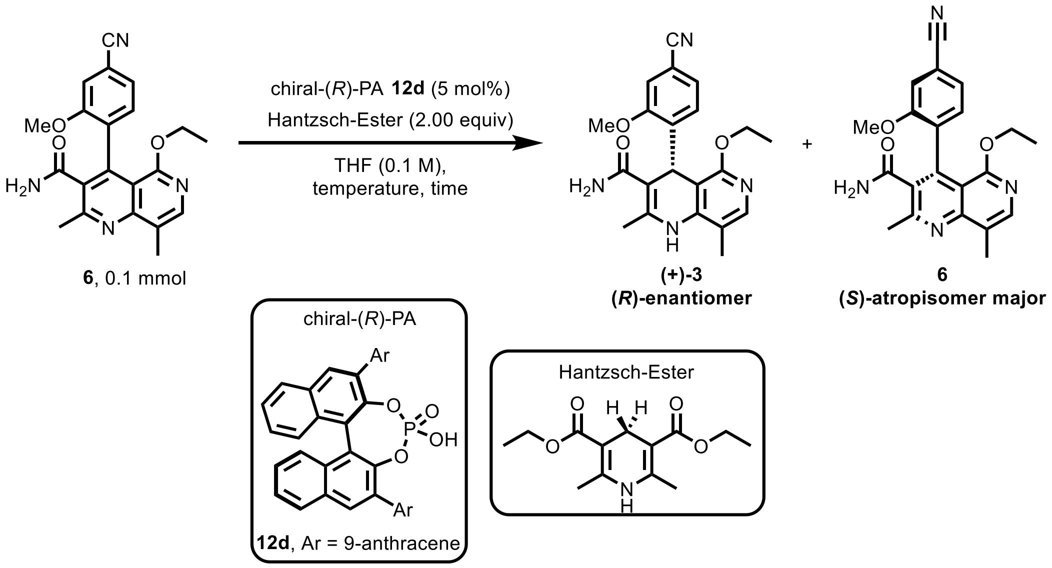

entry	temperature	time	(+)-3 yield	*e.r.* (+)-3	(6) yield	*e.r.* (6)	overall yield
1	40 °C	24 h	37%	98:2	60%	74:26	97%
2	40 °C	48 h	42%	97:3	53%	77:23	95%
3	40 °C	72 h	55%	97:3	45%	84:16	100%
4	60 °C	24 h	66%	96:4	32%	86:14	98%
5	70 °C	24 h	71%	94:6	24%	82:18	95%
6	80 °C	24 h	79%	93:7	16%	71:29	95%
7	100 °C	24 h	82%	94:6	11%	50:50	93%

For **(+)-3** the *ee* was determined by chiral SFC (IB, MeOH 20%). For (**6**) the *ee* was determined by chiral SFC (IC, MeOH 30%).

Full reaction profile of product yield and remaining starting material depending on temperature using enantiopure starting material

General procedure:

4-(4-Cyano-2-methoxyphenyl)-5-ethoxy-2,8-dimethyl-1,6-naphthyridine-3-carboxamide (**6**, 28.2 mg, 0.075 mmol, 1.00 equiv), the Hantzsch ester (**7**, 38 mg, 0.15 mmol, 2.00 equiv) and the chiral phosphoric acid catalyst **12d** (2.6 mg, 5 mol%) were added in a flame-dried Young-type pressure tube equipped with a stirring bar. After 3 vacuum/nitrogen cycles dry THF (0.75 mL, 0.1M) was added to the tube. Finally, the reaction was stirred at the indicated temperature in an oil bath for 24 h. Then, the reaction was cooled to room temperature and diluted with CH_2Cl_2 (5 mL). The organic solvents were evaporated and finally, the crude material was purified via flash column chromatography (eluent EtOAc). The remaining starting material (**6**) was always isolated as a white solid. **(–)-3** was always isolated as a white solid.

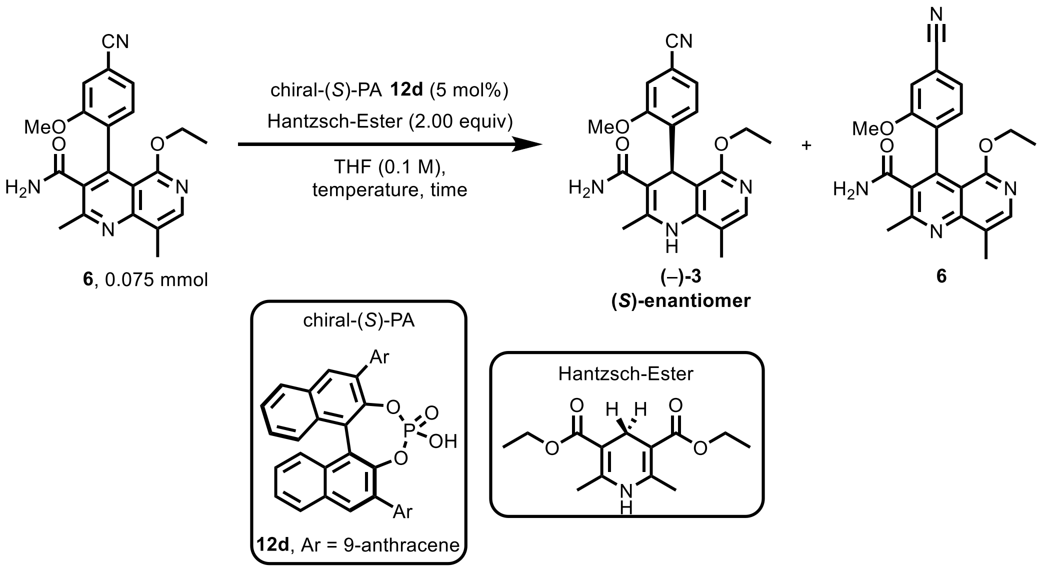

entry	atropisomer	temperature	(–)-3 yield	*e.r.* (–)-3	(6) yield	*e.r.* (*S*:*R* 6)	overall yield
1	*R*	40 °C	5%	61:39	95%	<1:99	100%
2	*R*	60 °C	20%	65:35	80%	6:94	100%
3	*R*	80 °C	39%	77:23	60%	26:74	99%
4	*R*	100 °C	47%	85:15	52%	48:52	99%
5	*S*	40 °C	42%	>99:1	55%	96:4	97%
6	*S*	100 °C	74%	98:2	24%	49:51	98%

For **3** the *ee* was determined by chiral SFC (IB, MeOH 20%). For (**6**) the *ee* was determined by chiral SFC (IC, MeOH 30%).

Rate determination

General procedure:

4-(4-Cyano-2-methoxyphenyl)-5-ethoxy-2,8-dimethyl-1,6-naphthyridine-3-carboxamide (**6**, 37.6 mg, 0.10 mmol, 1.00 equiv), the Hantzsch ester (**7**, 50.7 mg, 0.20 mmol, 2.00 equiv) and the chiral phosphoric acid catalyst **12d** (3.5 mg, 5 mol%) were added in a flame-dried Young-type pressure tube equipped with a stirring bar. After 3 vacuum/nitrogen cycles dry THF (1 mL, 0.1M) was added to the tube. Finally, the reaction was stirred at 40 °C in an oil bath for 4 hours. Then, the reaction was cooled to room temperature and diluted with CH_2Cl_2 (5 mL). 1,3,5-trimethoxybenzene (16.8 mg, 0.10 mmol, 1.00 equiv) was added to the tube. Next, the organic solvents were evaporated and finally, the crude material was analysed via H-NMR to determine the NMR-yield. Afterwards, the crude material was subjected to the chiral SFC to determine the enantiomeric ratio of the remaining starting material (SFC, IC, MeOH 30%).

Based on the NMR-yield of the remaining starting material and the obtained enantiomeric ratio we were able to determine the rate of the reaction for both atropisomers.

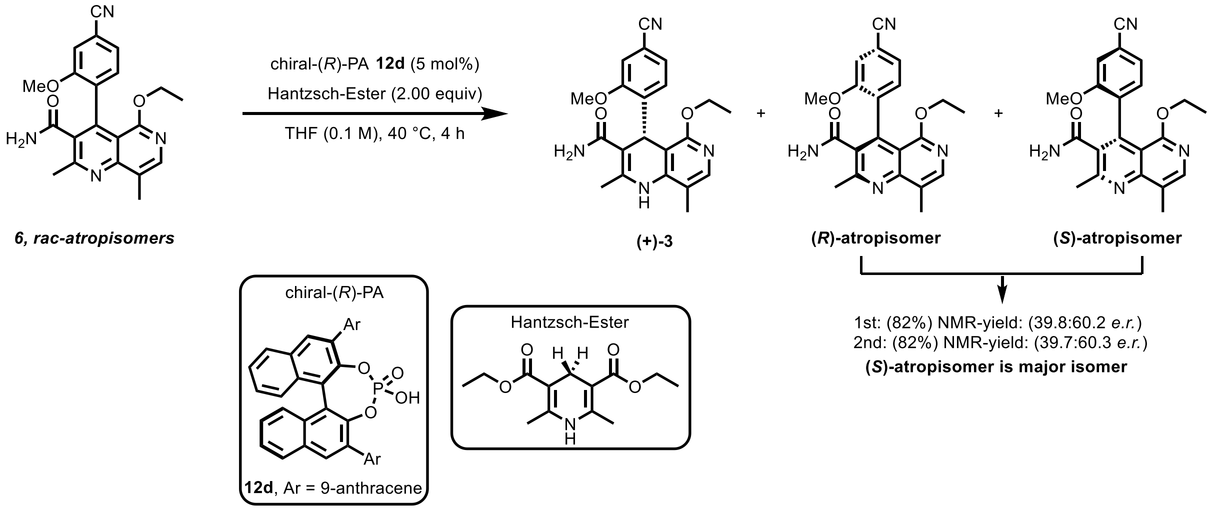

First run:

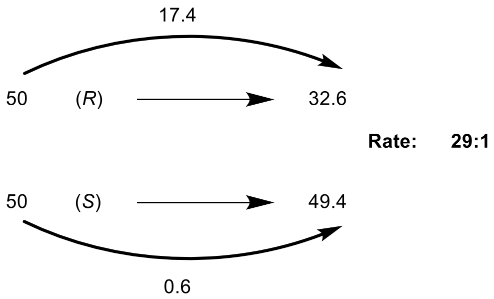

Second run:

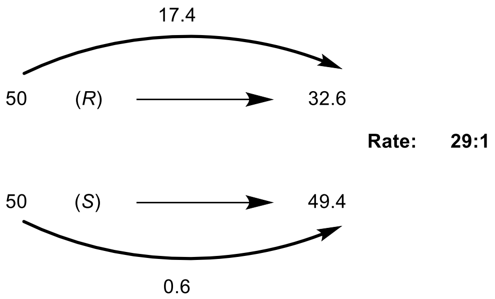

First run: NMR-yield of remaining SM

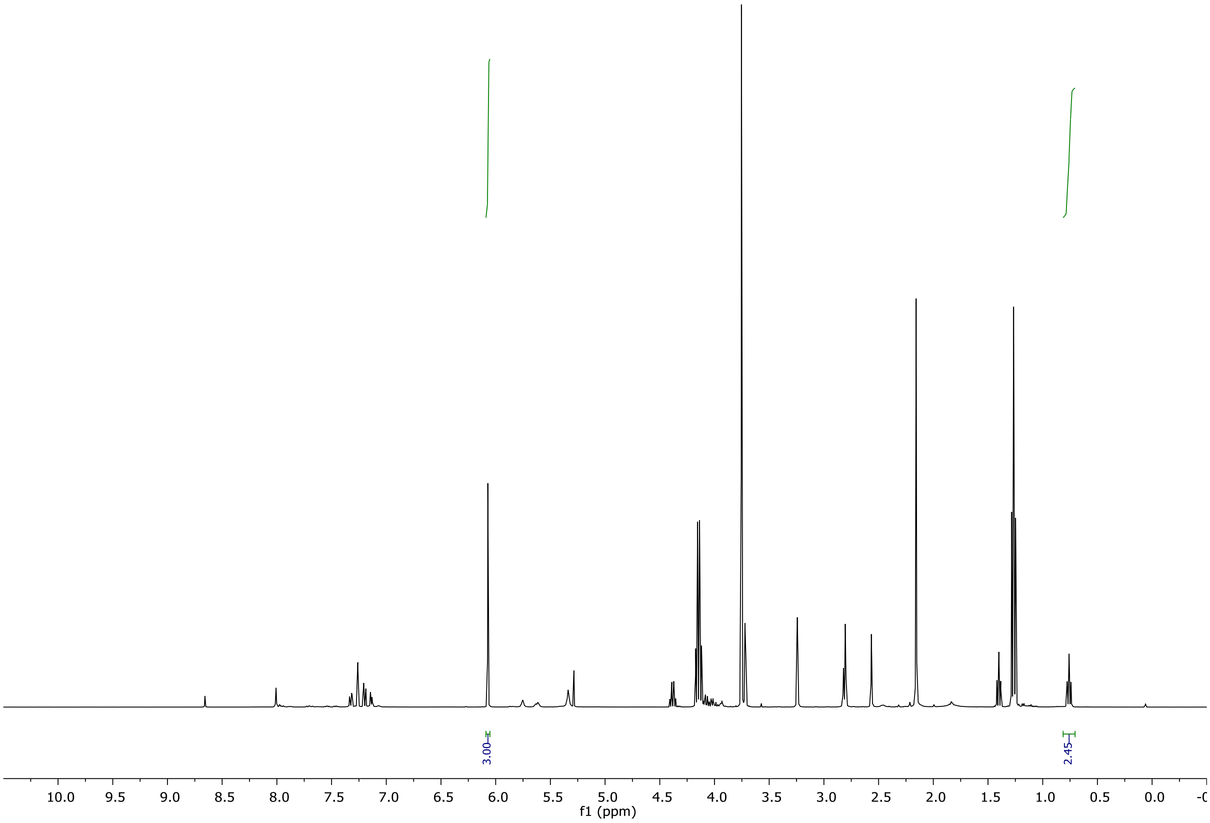

Second run: NMR-yield of remaining SM

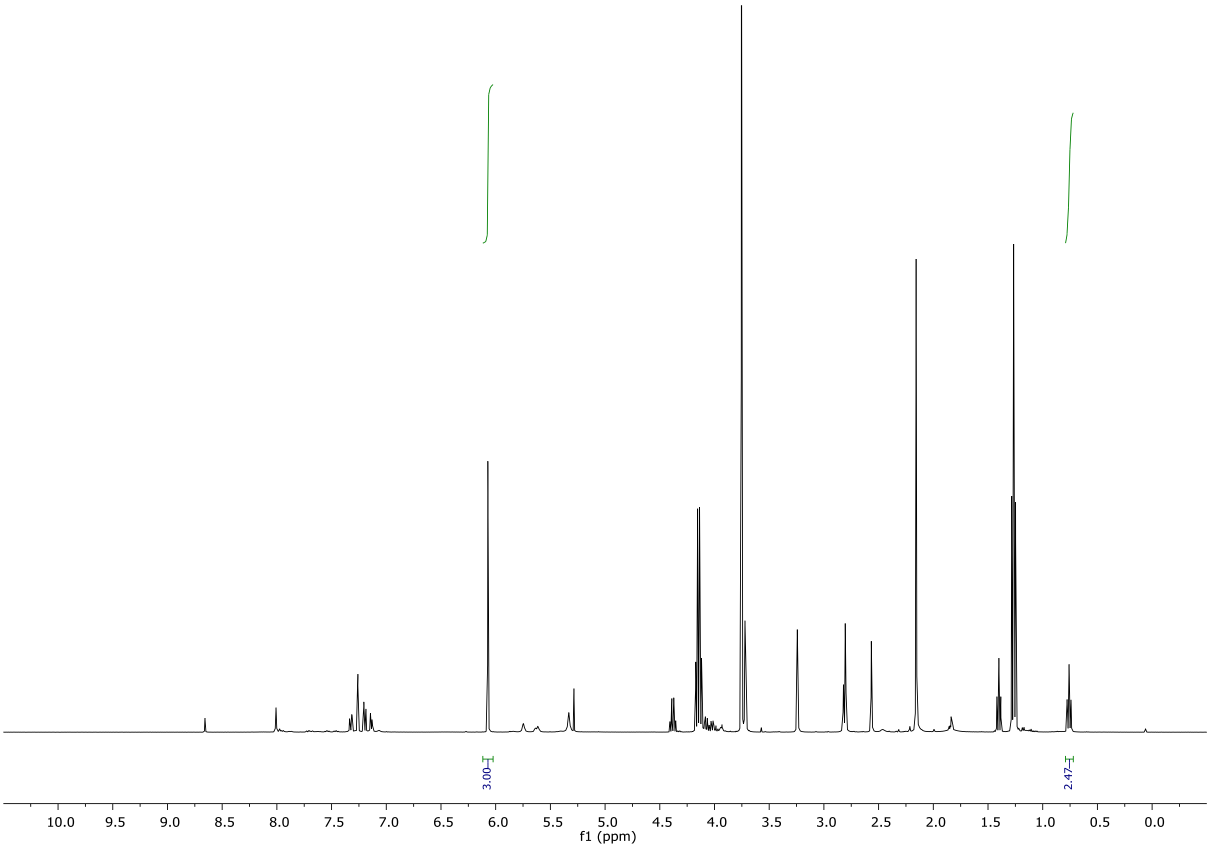

First run: crude SFC-trace

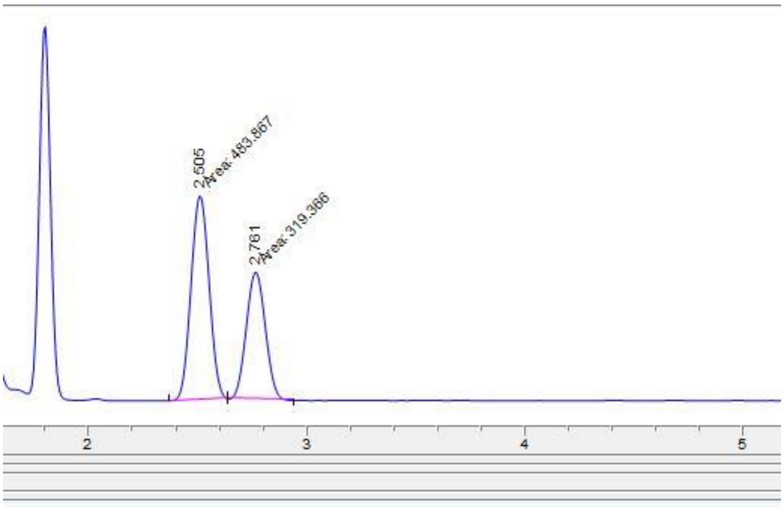

#	Time	Area	Height	Width	Area%	Symmetry
1	2.505	483.9	84.2	0.0958	60.240	0.958
2	2.761	319.4	52.6	0.1012	39.760	0.948

Second run: crude SFC-trace

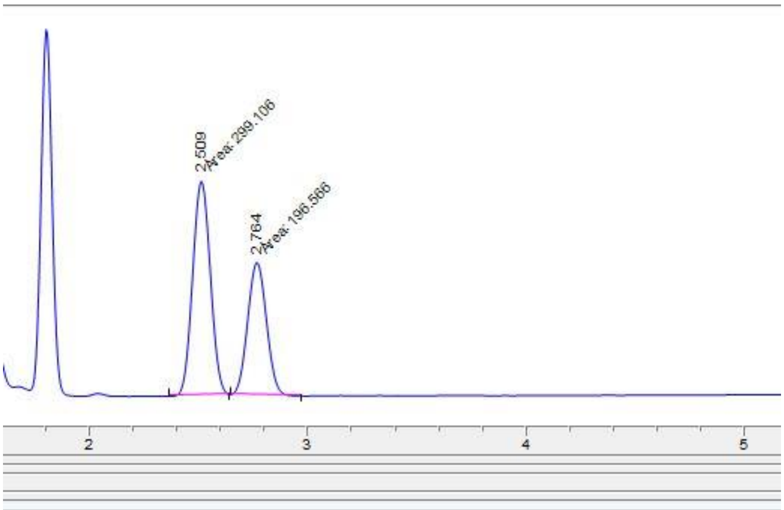

#	Time	Area	Height	Width	Area%	Symmetry
1	2.509	299.1	53	0.094	60.344	0.953
2	2.764	196.6	32.8	0.0998	39.656	0.943

5. Synthesis of Finerenone (–)-3 via temperature gradient

(*S*)-4-(4-cyano-2-methoxyphenyl)-5-ethoxy-2,8-dimethyl-1,4-dihydro-1,6-naphthyridine-3-carboxamide (–)-3 (Finerenone)

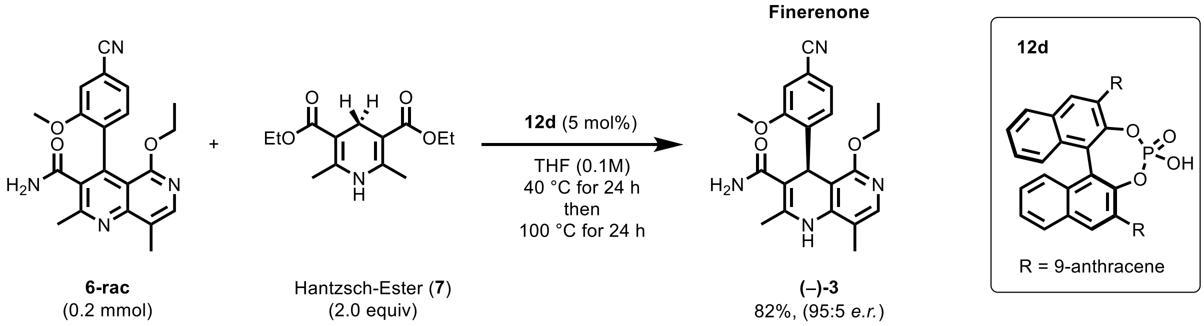

4-(4-Cyano-2-methoxyphenyl)-5-ethoxy-2,8-dimethyl-1,6-naphthyridine-3-carboxamide (**6**, 75.3 mg, 0.2 mmol, 1.00 equiv), the Hantzsch ester (**7**, 101.3 mg, 0.40 mmol, 2.00 equiv) and the chiral phosphoric acid catalyst **12d** (7 mg, 5 mol%) were added in a flame-dried Young-type pressure tube equipped with a stirring bar. After 3 vacuum/nitrogen cycles dry THF (2 mL, 0.1M) was added to the tube. Finally, the reaction was stirred at 40 °C in an oil bath for 24 hours. Afterwards, the reaction was stirred for additional 24 hours at 100 °C. Then, the reaction was cooled to room temperature and diluted with CH_2Cl_2 (10 mL). The organic solvents were evaporated and finally, the crude material was purified via flash column chromatography (eluent EtOAc). Finerenone **(–)-3** was isolated as a white solid (62 mg, 0.164 mmol, 82%).

M.P.: 235 – 238 °C.

R*f*: 0.21 (pentane/EtOAc, 20:80).

^{1}H NMR (400 MHz, DMSO-d_6) δ 7.67 (s, 1H), 7.55 (s, 1H), 7.36 (d, J = 1.5 Hz, 1H), 7.27 (dd, J = 7.9, 1.5 Hz, 1H), 7.15 (d, J = 7.9 Hz, 1H), 6.88 – 6.50 (bs, 2H), 5.37 (s, 1H), 4.08 – 3.95 (m, 2H), 3.82 (s, 3H), 2.18 (s, 3H), 2.12 (s, 3H), 1.05 (t, J = 7.0 Hz, 3H).

^{13}C NMR (125 MHz, DMSO-d_6) δ 169.6, 159.3, 155.7, 144.2, 144.1, 141.6, 138.0, 130.8, 124.7, 118.9, 114.1, 111.4, 109.5, 105.3, 103.1, 60.5, 56.0, 32.4, 18.0, 14.3, 13.7.

HRMS: (ESI) calculated for $C_{21}H_{23}N_4O_3$ [M + H]$^+$: 379.1765, found: 379.1753.

$[\alpha]_D^{20}$ ($CHCl_3$, 23.3 °C, 1mg/mL): –110.

IR: (film) ν_{max}/cm^{-1}: 3456, 3343, 2977, 2927, 2858, 2229, 1663, 1570, 1490, 1445, 1381, 1334, 1268, 1137, 1034, 925, 826.

Enantiomeric ratio of 5:95 was determined using chiral SFC analysis (IB, 20% MeOH, 3 mL/min, 125 bar): t_R = 4.8 [(*R*)], 5.2 [(*S*)].

Racemic (**3**)

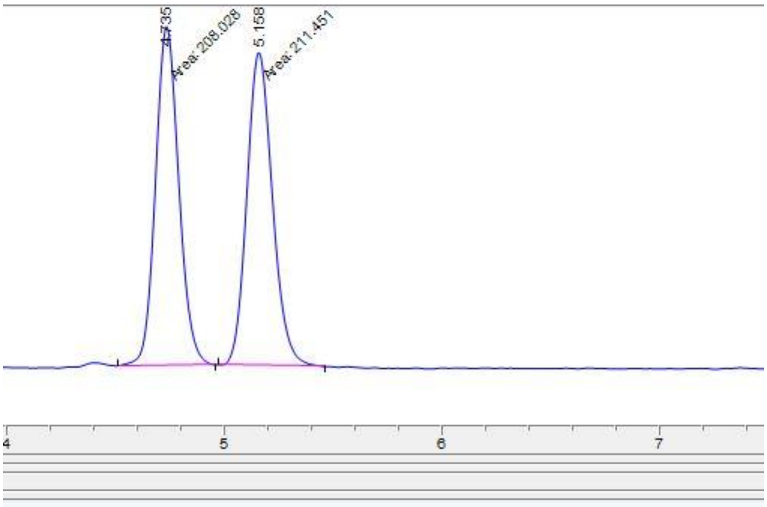

#	Time	Area	Height	Width	Area%	Symmetry
1	4.735	208	28	0.1238	49.592	0.89
2	5.158	211.5	25.9	0.1361	50.408	0.842

(–)-3 Finerenone

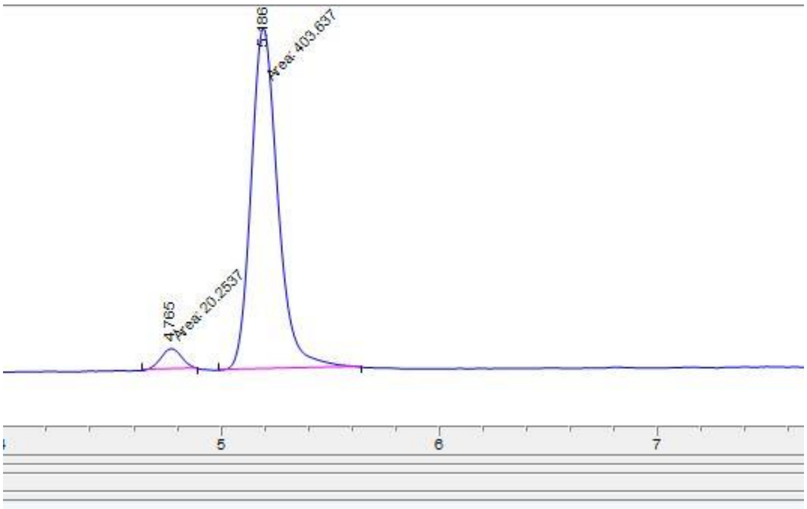

#	Time	Area	Height	Width	Area%	Symmetry
1	4.765	20.3	2.9	0.1155	4.778	0.987
2	5.186	403.6	47.1	0.1428	95.222	0.792

6. Big Scale Synthesis of Finerenone (–)-3

Big Scale: (*S*)-4-(4-cyano-2-methoxyphenyl)-5-ethoxy-2,8-dimethyl-1,4-dihydro-1,6-naphthyridine-3-carboxamide (–)-3 (Finerenone)

4-(4-Cyano-2-methoxyphenyl)-5-ethoxy-2,8-dimethyl-1,6-naphthyridine-3-carboxamide (**6**, 728 mg, 1.93 mmol, 1.00 equiv), the Hantzsch ester (**7**, 980 mg, 3.86 mmol, 2.00 equiv) and the chiral phosphoric acid catalyst **12d** (67.2 mg, 0.97 mmol, 5 mol%) were added in a flame-dried Young-type pressure tube equipped with a stirring bar. After 3 vacuum/nitrogen cycles dry THF (19 mL, 0.1M) was added to the tube. Finally, the reaction was stirred at 100 °C in an oil bath for 48 hours. Then, the reaction was cooled to room temperature and diluted with CH_2Cl_2 (50 mL). The organic solvents were evaporated and finally, the crude material was purified via flash column chromatography (eluent EtOAc). Finerenone **(–)-3** was isolated as a white solid (489 mg, 1.29 mmol, 67%).

M.P.: 235 – 238 °C.
R*f*: 0.21 (pentane/EtOAc, 20:80).
^{1}H NMR (400 MHz, DMSO-*d*$_6$) *δ* 7.67 (s, 1H), 7.55 (s, 1H), 7.36 (d, *J* = 1.5 Hz, 1H), 7.27 (dd, *J* = 7.9, 1.5 Hz, 1H), 7.15 (d, *J* = 7.9 Hz, 1H), 6.88 – 6.50 (bs, 2H), 5.37 (s, 1H), 4.08 – 3.95 (m, 2H), 3.82 (s, 3H), 2.18 (s, 3H), 2.12 (s, 3H), 1.05 (t, *J* = 7.0 Hz, 3H).
^{13}C NMR (125 MHz, DMSO-*d*$_6$) *δ* 169.6, 159.3, 155.7, 144.2, 144.1, 141.6, 138.0, 130.8, 124.7, 118.9, 114.1, 111.4, 109.5, 105.3, 103.1, 60.5, 56.0, 32.4, 18.0, 14.3, 13.7.
HRMS: (ESI) calculated for $C_{21}H_{23}N_4O_3$ $[M + H]^+$: 379.1765, found: 379.1753.
$[\alpha]_D^{20}$ ($CHCl_3$, 23.3 °C, 1mg/mL): –110.
$[\alpha]_D^{20}$ (MeOH, 23.3 °C, c=1): –173.
IR: (film) ν_{max}/cm^{-1}: 3456, 3343, 2977, 2927, 2858, 2229, 1663, 1570, 1490, 1445, 1381, 1334, 1268, 1137, 1034, 925, 826.

Enantiomeric ratio of 6:94 was determined using chiral SFC analysis (IB, 20% MeOH, 3 mL/min, 125 bar): t_R = 4.8 [(*R*)], 5.2 [(*S*)].

Recrystallization of the product from EtOH/H_2O improved the *e.r.* to 97.5:2.5.

Racemic (**3**)

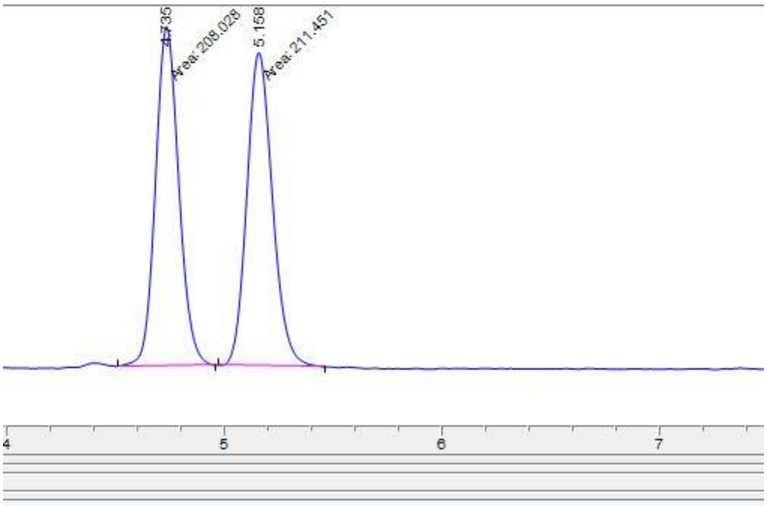

#	Time	Area	Height	Width	Area%	Symmetry
1	4.735	208	28	0.1238	49.592	0.89
2	5.158	211.5	25.9	0.1361	50.408	0.842

(–)-3 Finerenone

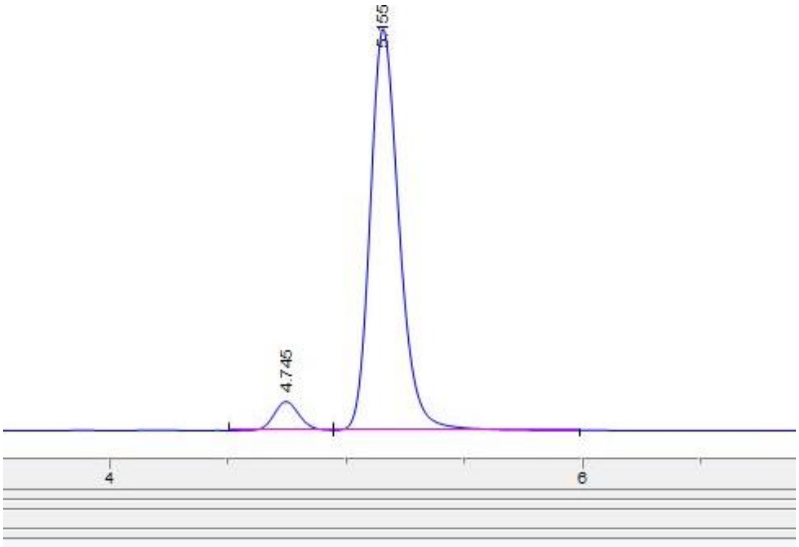

#	Time	Area	Height	Width	Area%	Symmetry
1	4.745	107.6	14.3	0.116	6.005	0.878
2	5.155	1684.6	197.8	0.1294	93.995	0.751

Enatiomeric access after recrystallization: (–)-3 Finerenone

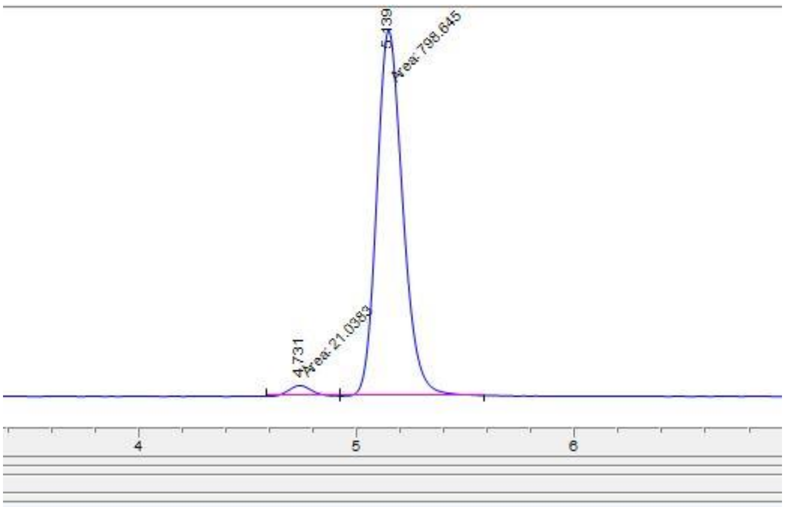

#	Time	Area	Height	Width	Area%	Symmetry
1	4.731	21	2.8	0.1235	2.567	0.901
2	5.139	798.6	96.3	0.1382	97.433	0.804

7. NMR Spectra

Spectra in $CDCl_3$

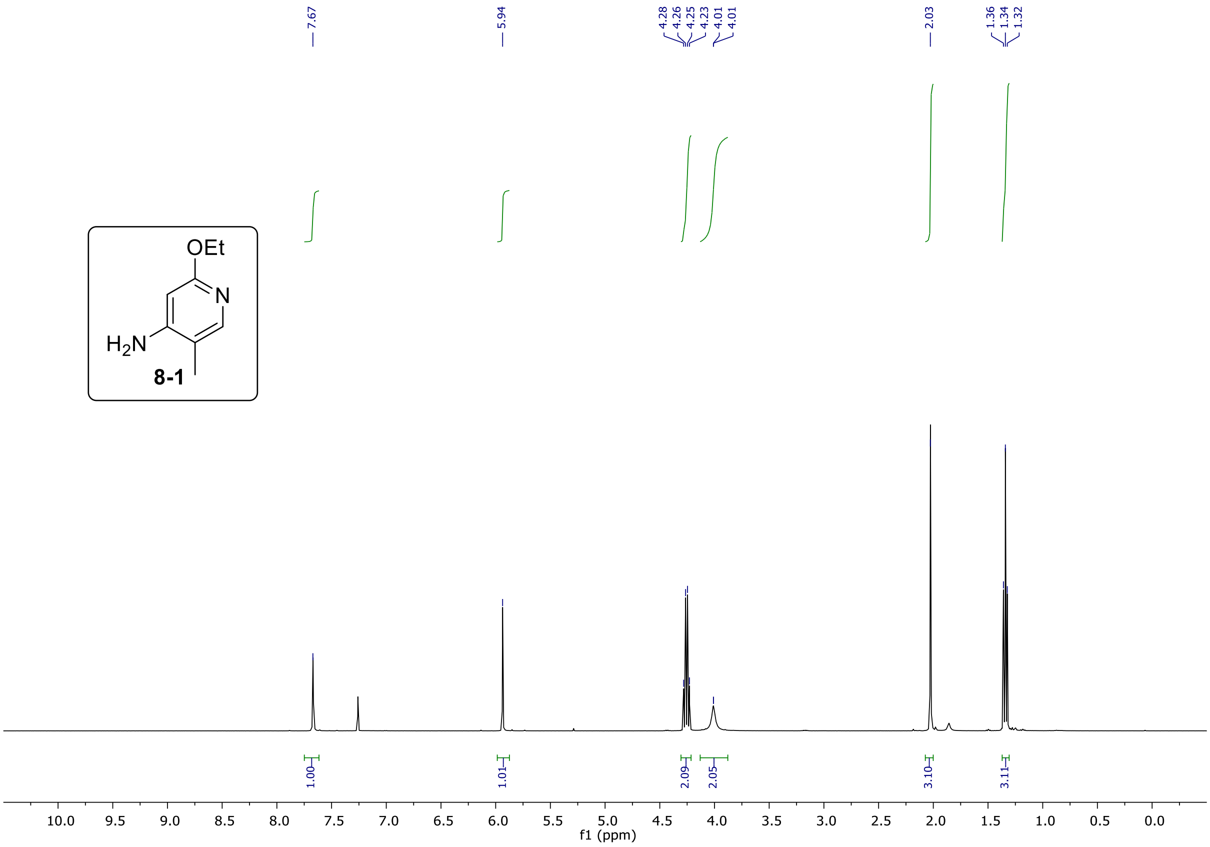

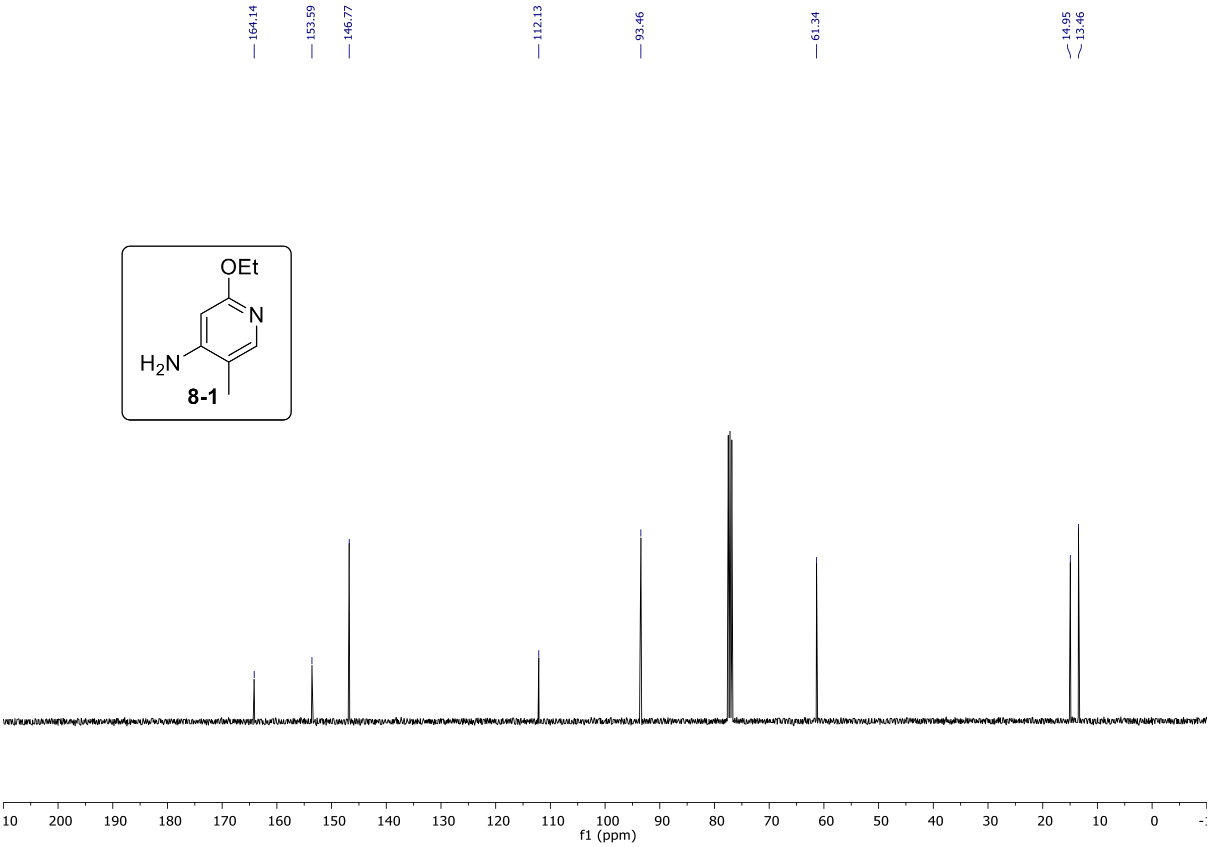

Spectra in $CDCl_3$

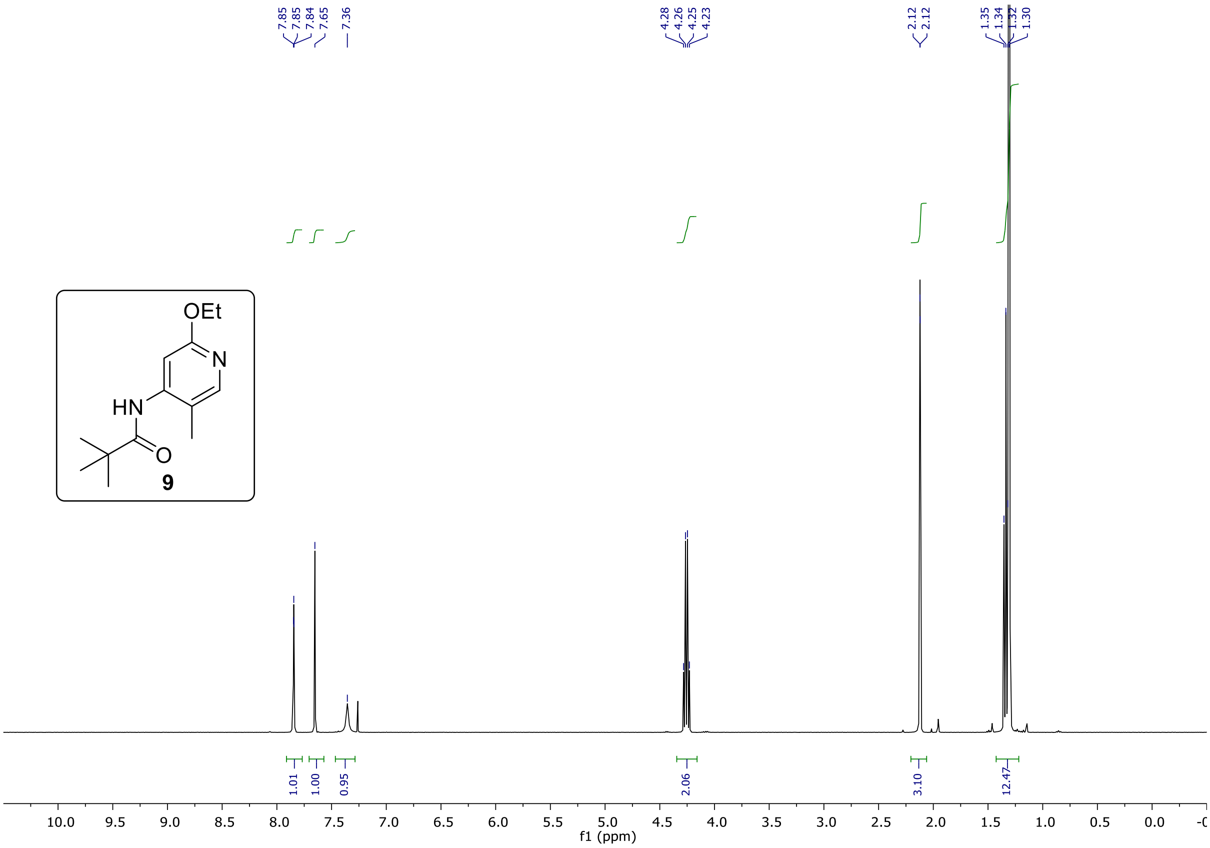

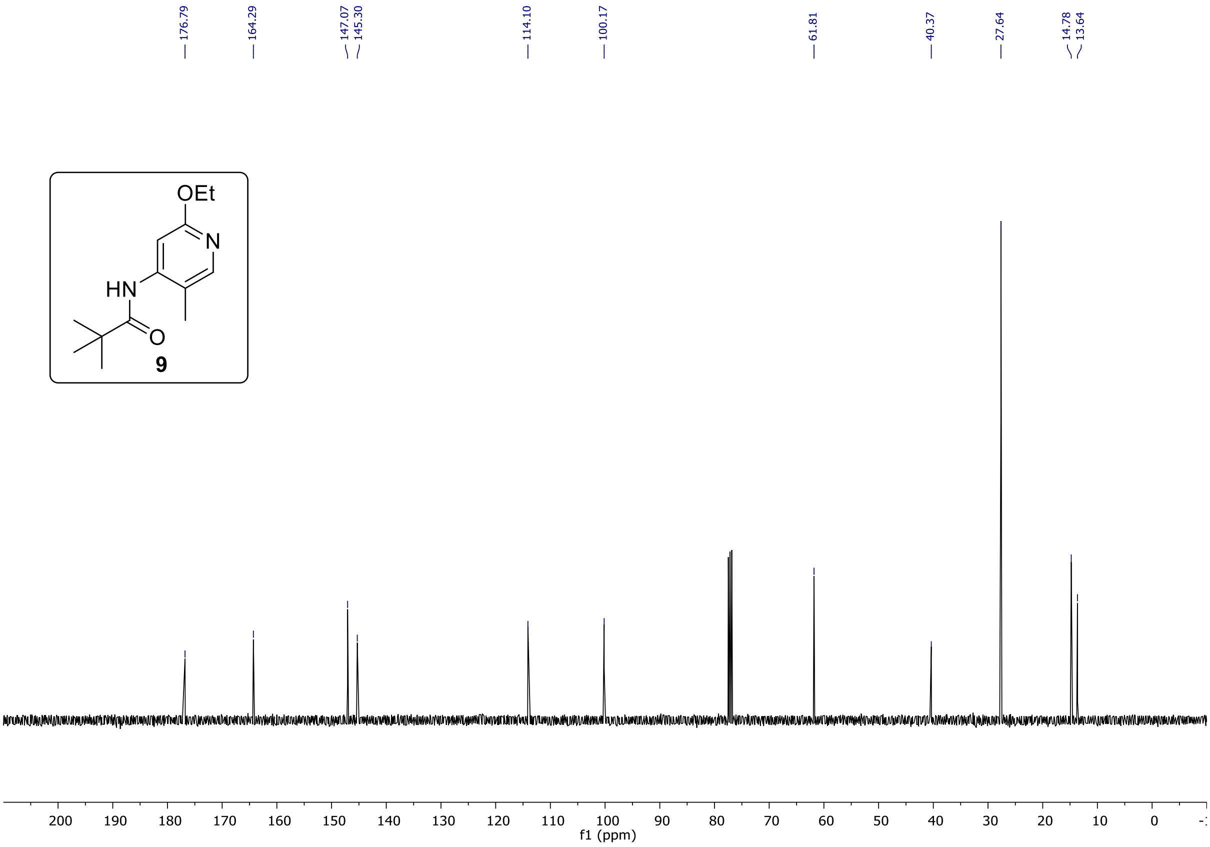

Spectra in $CDCl_3$

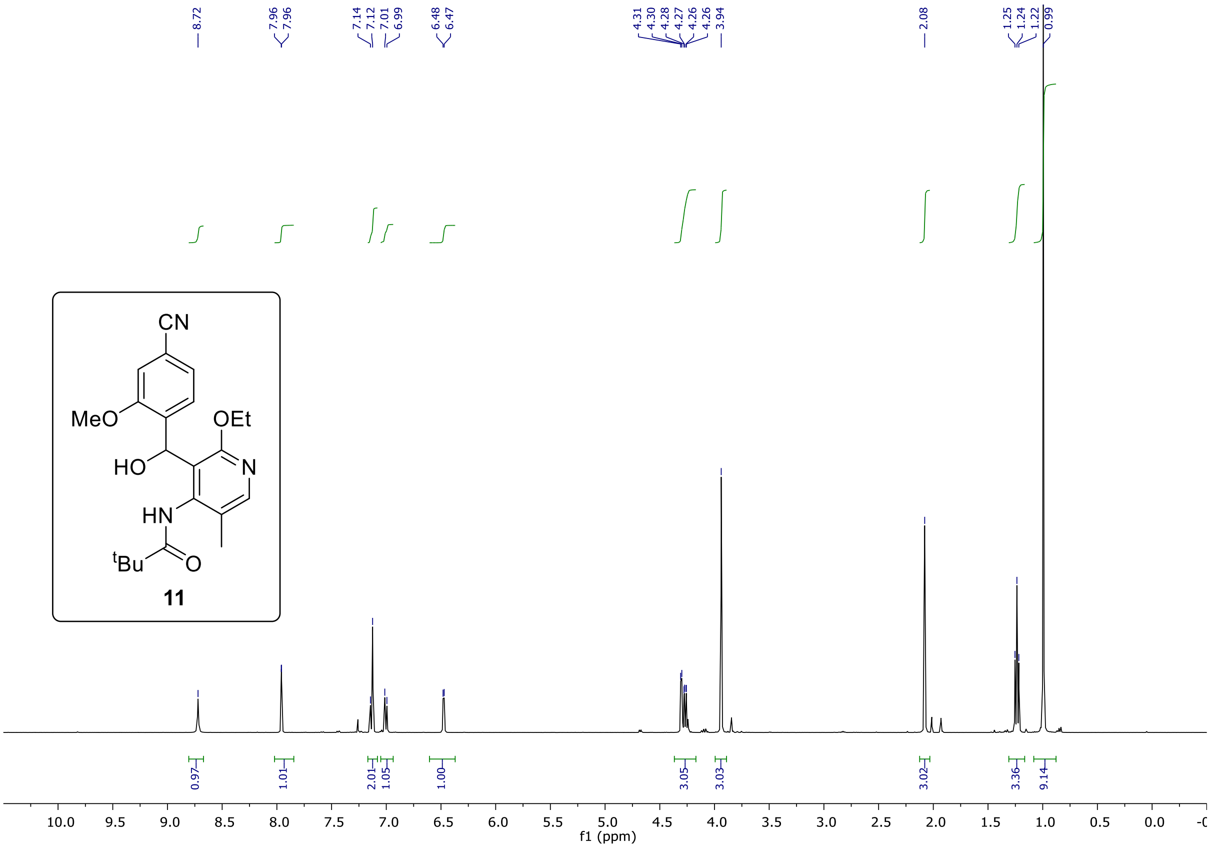

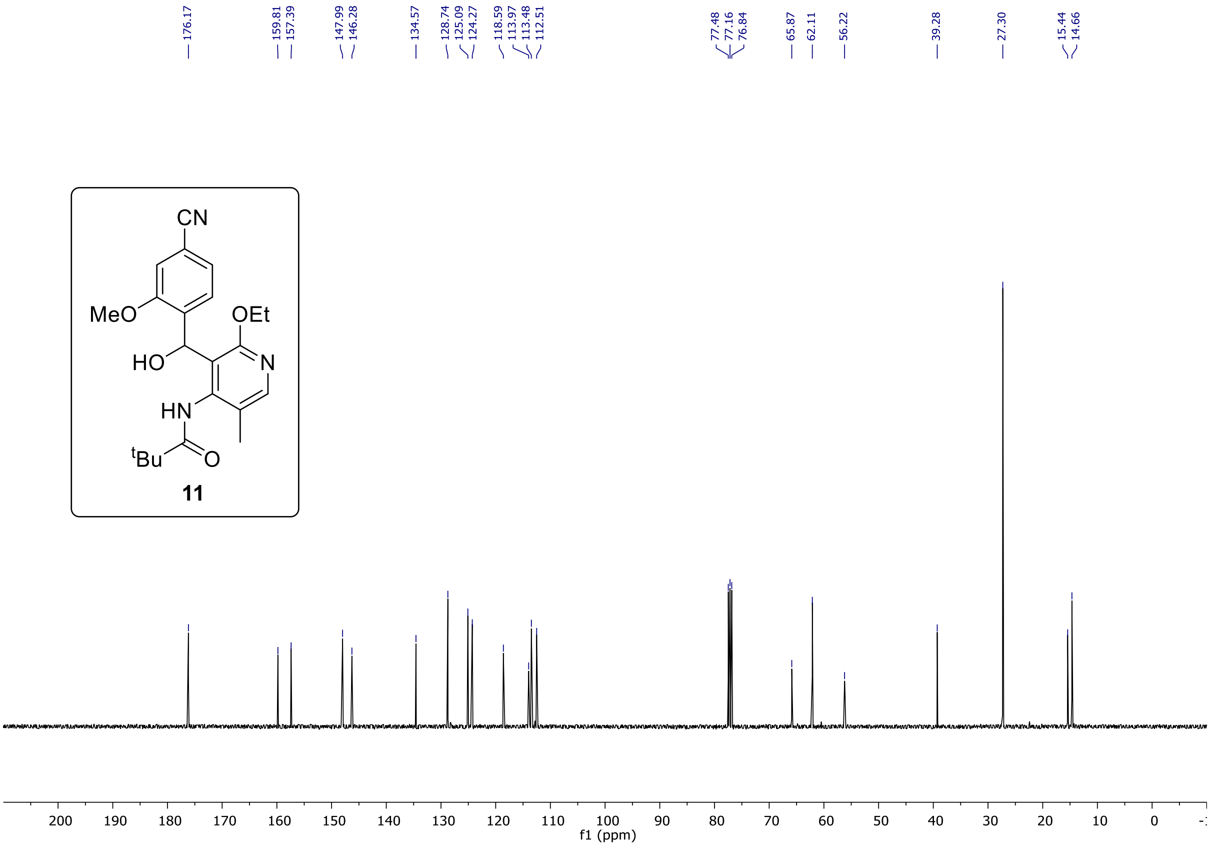

Spectra in $CDCl_3$

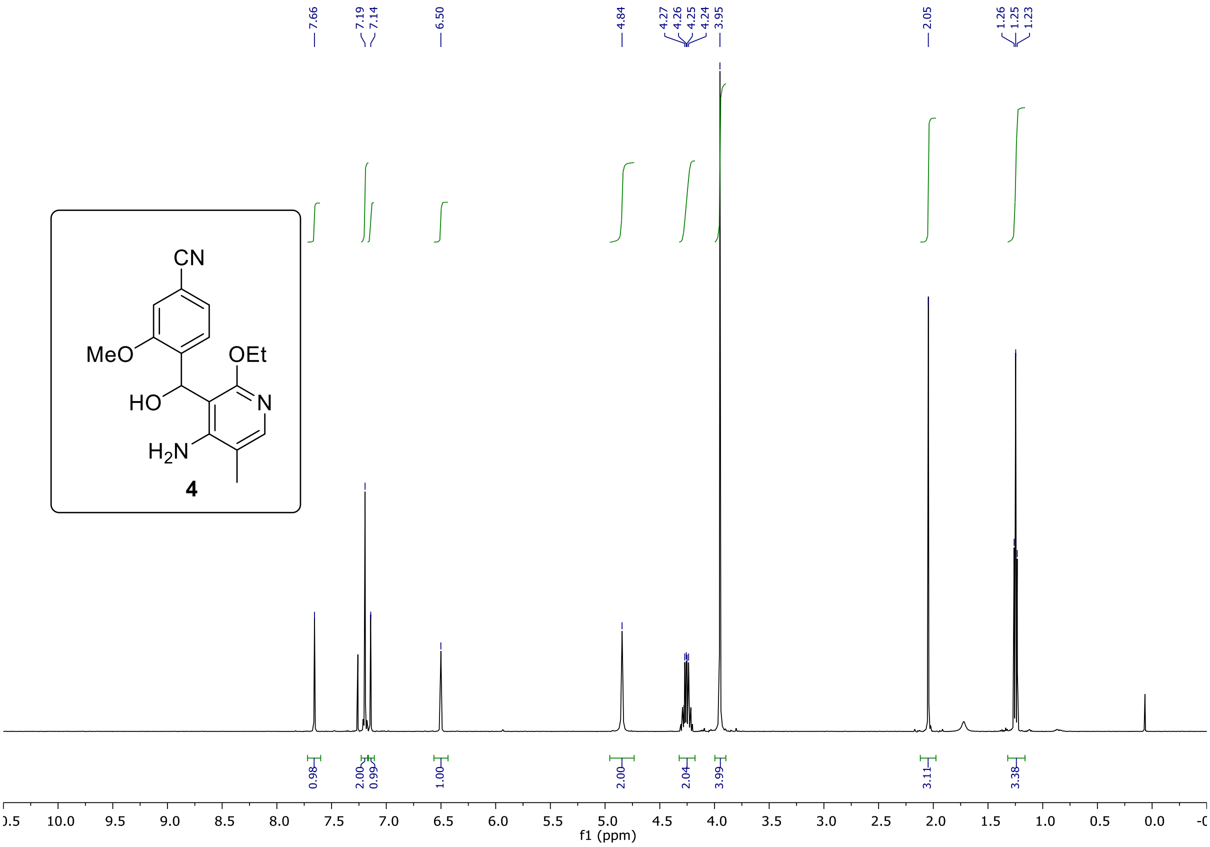

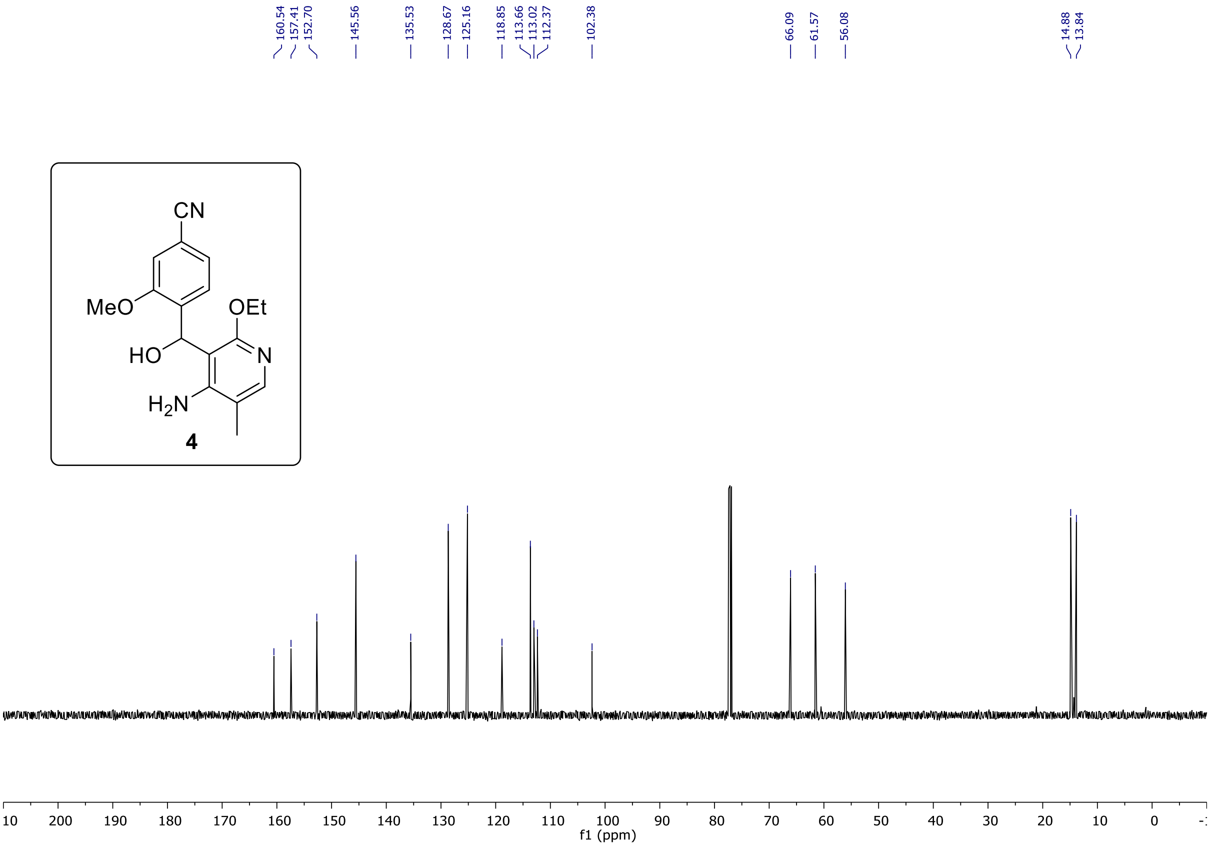

Spectra in Methanol-d_4

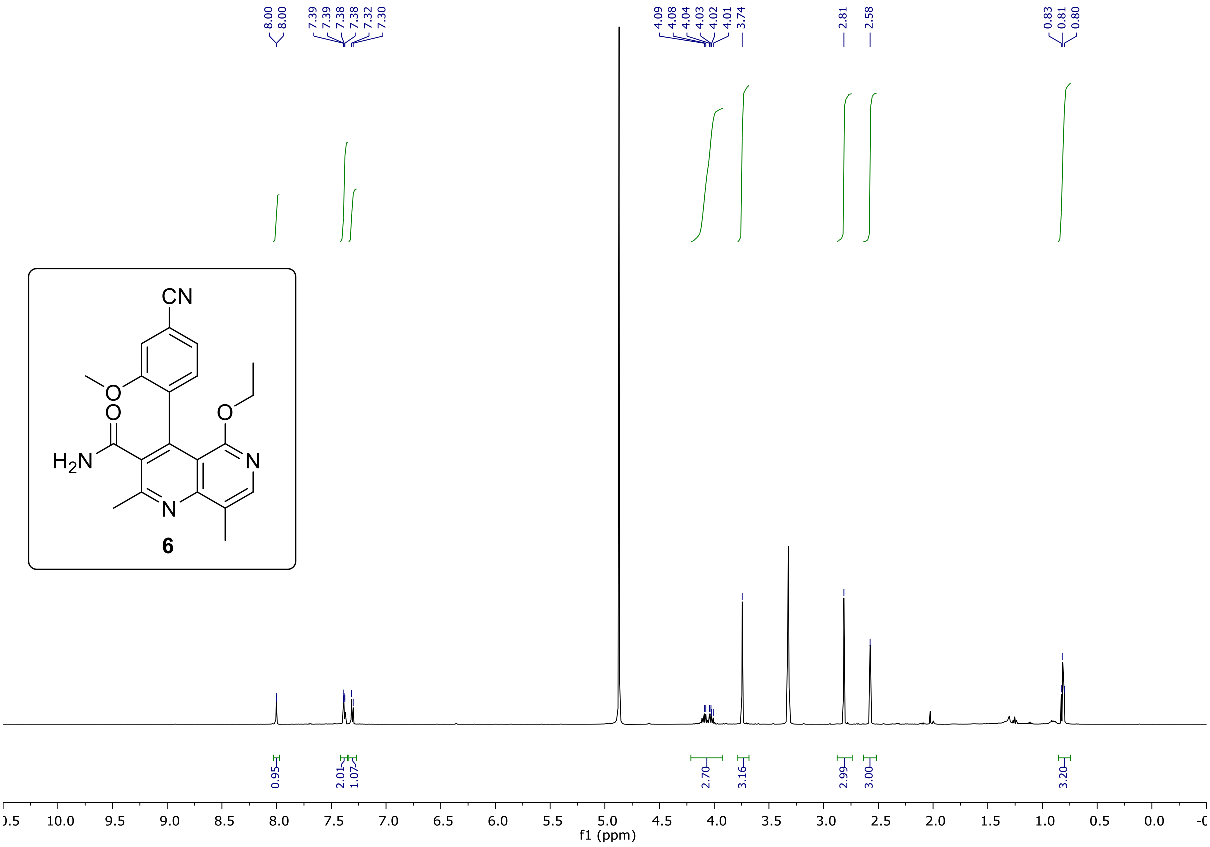

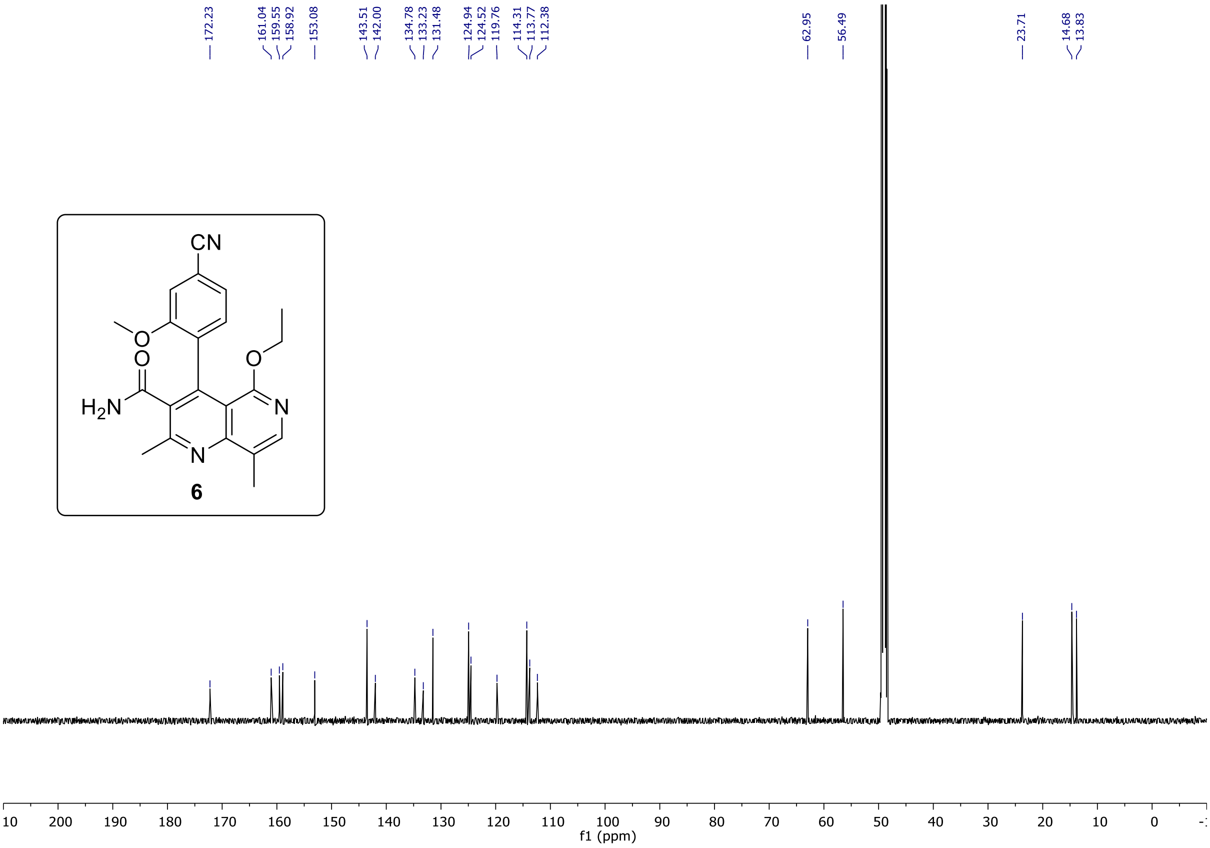

Spectra in $CDCl_3$

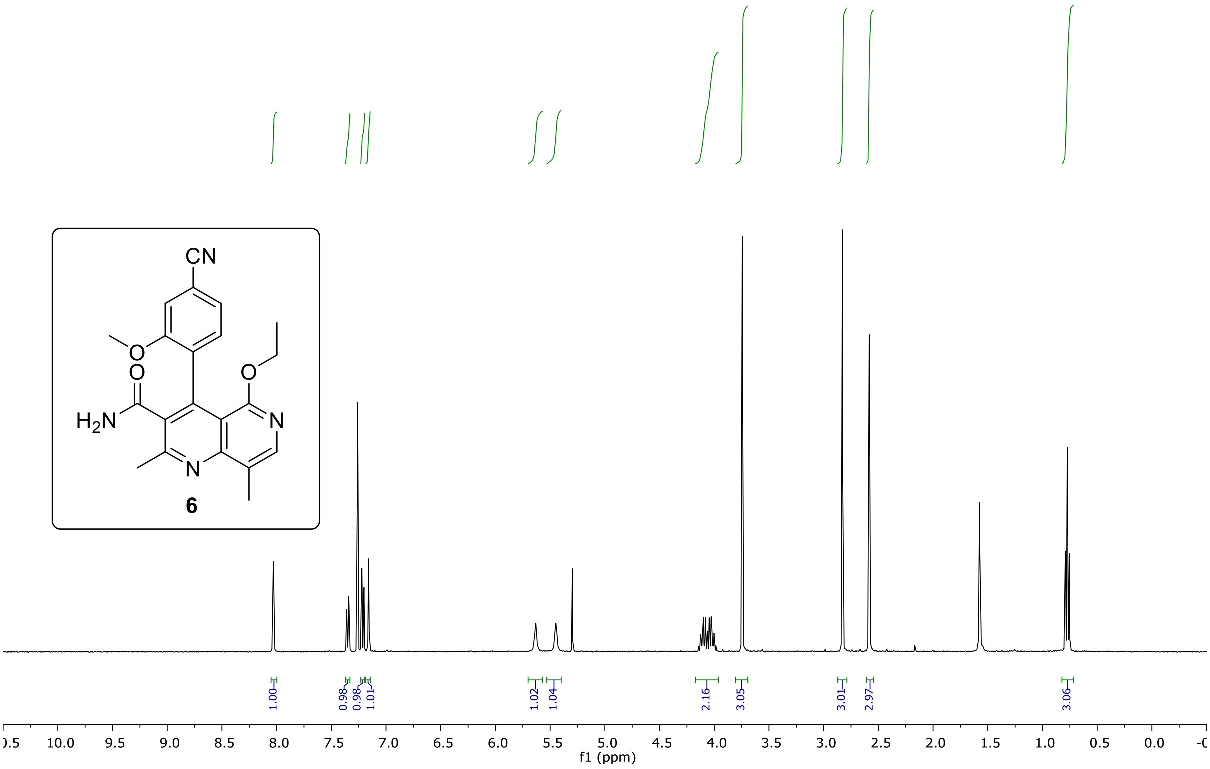

Spectra in DMSO-d_6

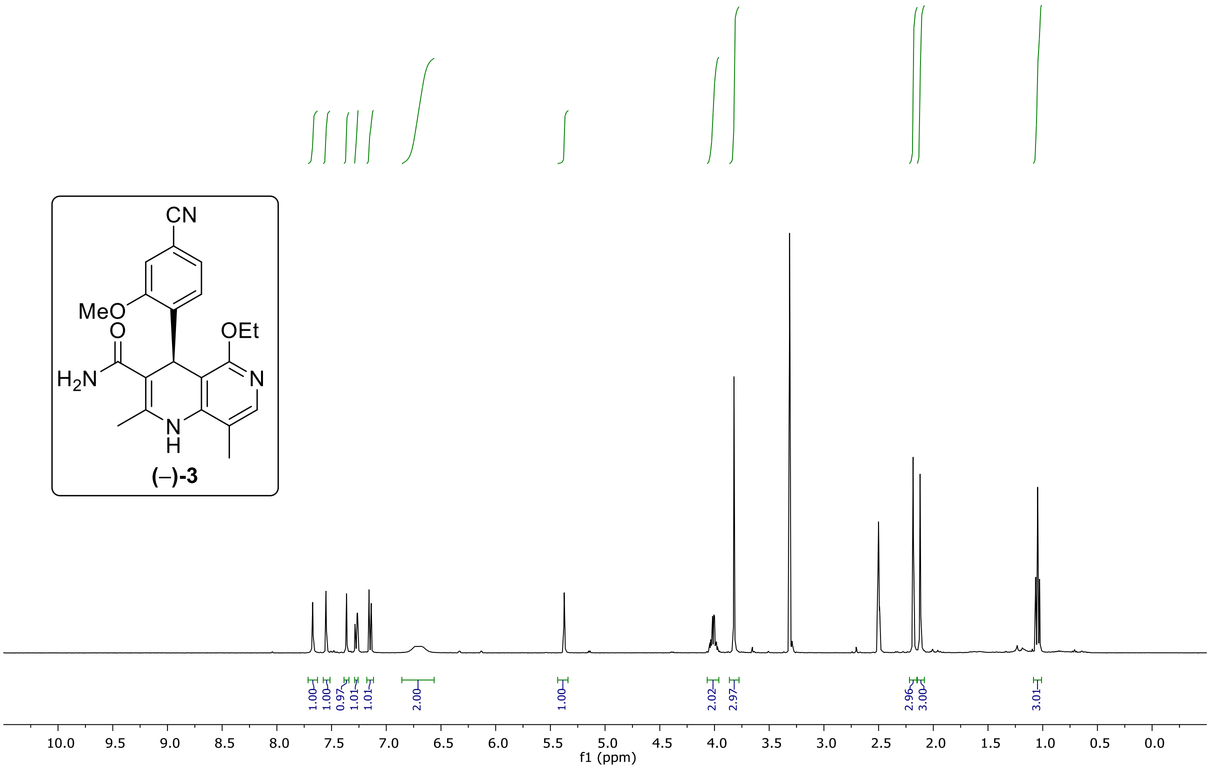

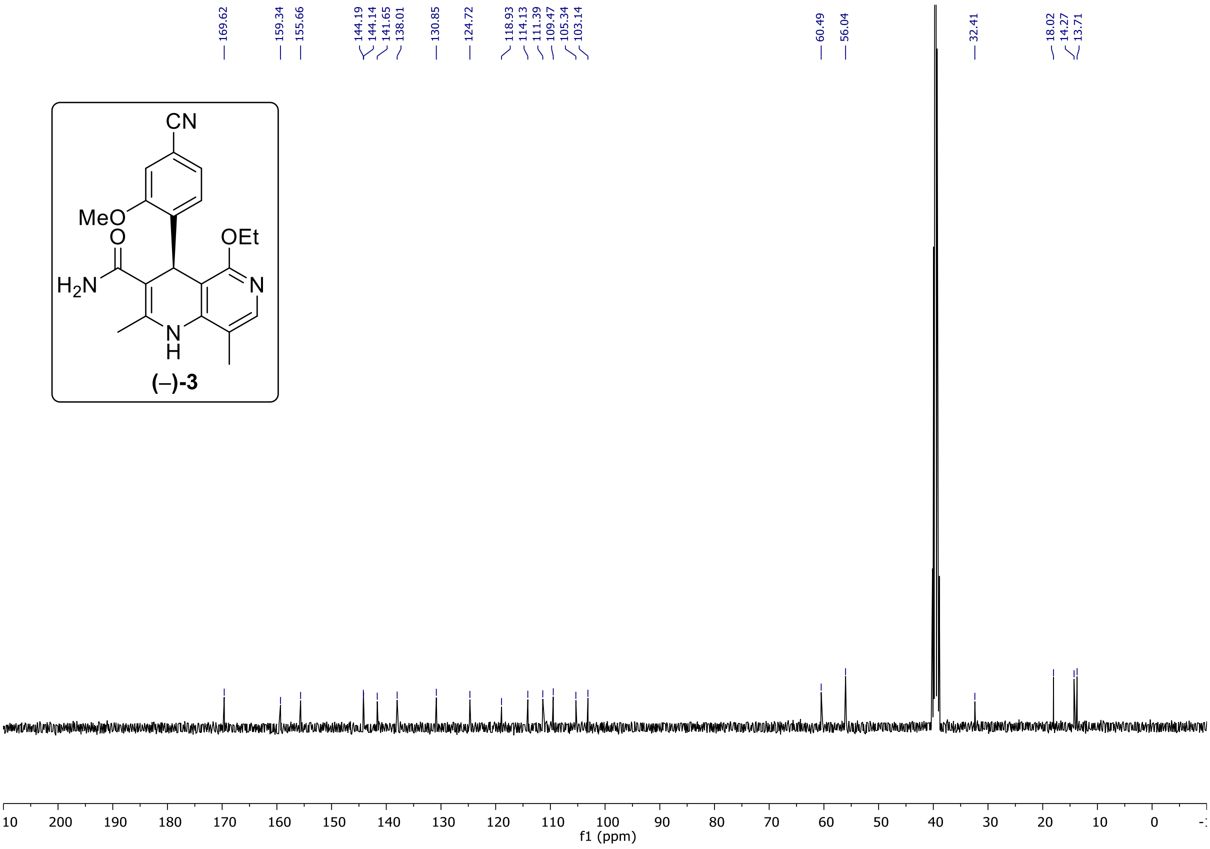

8. Computational Details

Conformational searches were carried out for all computed structures using the conformational search tool within Schrödinger's MacroModel (version 11.3)[1,2] with the OPLS3e force field[3]. A Monte Carlo Multiple Minimum (MMCM)[4] / low-mode sampling approach[5] was used to explore the possible conformations of each species. Conformations provided by these searches were subsequently optimized by DFT calculations carried out using Gaussian16 (Revision A.03)[6] with the B3LYP density functional[7,8] and a split-valence polarized 6-31G(d) basis set[9].

Single point energy (SPE) calculations were used to correct the Gibbs free energy derived from the original B3LYP calculations[10]. These were performed with an ultrafine integration grid using the M06-2X density functional[11] and the larger split-valence polarized 6-311G(d,p) basis set[12]. The Solvent Model based on Density (SMD)[13] (tetrahydrofuran) was used to incorporate the effect of solvent. All temperature (313.15 K) and concentration-corrected (1 mol/l) quasiharmonic (Grimme approximation[14]) free energies were calculated with GoodVibes[15] with a vibrational scaling factor of 0.977[16].

Similar methods have previously been used for the successful modelling of related chiral phosphoric acid-catalysed reactions[17,18].

All computed ratios were obtained by taking a Boltzmann weighting over all conformers within 5 kcal mol^{-1} of the lowest in free energy at 313.15 K.

9. Energies and molecular geometries of all computed structures

All energies in Hartrees, coordinates in Å. Cartesian coordinates generated by ESIgen software[19]. All structures contain (*R*)-catalyst. Relevant structures included in the manuscript were mirrored to give the (*S*)-enantiomer of the catalyst, and are marked with an asterisk (*).

R-forming TS Conformation 1

B3LYP/6-31G(d) Energy = -4490.493657
M06-2X/6-311G(d,p)-SMD(tetrahydrofuran) Energy = -4489.998935
M06-2X/6-311G(d,p)-SMD(tetrahydrofuran)-derived Free Energy (Quasiharmonic) = -4488.900943

Frequencies (Top 3 out of 468)

1. -1222.9552 cm^{-1}
2. 6.2991 cm^{-1}
3. 9.3963 cm^{-1}

B3LYP/6-31G(d) Molecular Geometry in Cartesian Coordinates

N	1.024413	-1.815366	1.154553
C	1.505644	-2.469792	0.051455
C	2.866526	-2.713530	-0.022530
C	3.107924	-1.789690	2.258958
C	3.741413	-2.157336	1.003312
H	4.213426	-0.956353	0.466456
H	0.030232	-1.485919	1.145997
C	3.547497	-3.505867	-1.072644
O	2.719596	-4.008737	-2.013476
O	4.752212	-3.698433	-1.077976
C	4.010238	-1.651497	3.418592
O	3.400744	-1.702048	4.616313
O	5.223659	-1.524586	3.302818
C	3.356215	-4.744269	-3.072871
H	3.952415	-4.066944	-3.689561
H	3.995674	-5.530734	-2.665736
H	2.540548	-5.171713	-3.656983
C	4.261657	-1.545071	5.759554
H	4.753138	-0.569283	5.735355
H	3.605637	-1.623539	6.626373
H	5.021299	-2.329977	5.775618
C	0.453215	-2.859135	-0.948599
H	0.721347	-2.518424	-1.949746
H	0.357618	-3.947244	-0.995136
H	-0.516444	-2.444731	-0.669402
C	0.936297	-1.036771	3.446245
H	0.753830	-1.849179	4.158180
H	1.469925	-0.249492	3.980330
H	-0.027631	-0.662524	3.095001
P	-1.989567	0.159685	0.086978
O	-1.400600	-0.587081	1.259655
O	-1.104318	0.697182	-1.000825
O	-3.135574	-0.778254	-0.668026

O	-2.937326	1.345881	0.745271
H	4.710831	-2.642942	1.079847
C	-3.899642	1.942578	-0.047557
C	-5.055886	1.234461	-0.348047
C	-4.656634	3.874573	-1.258075
C	-3.688359	3.287856	-0.468638
H	-4.523032	4.903776	-1.581423
C	-5.252109	-0.125781	0.231064
C	-4.284717	-1.102785	0.028218
C	-4.455856	-2.450874	0.458524
C	-5.601024	-2.765513	1.161730
H	-5.755670	-3.790411	1.489497
C	-6.757767	3.773136	-2.556609
C	-7.853915	3.072351	-3.002480
C	-8.041295	1.730432	-2.594674
C	-7.149962	1.124607	-1.736895
C	-6.016118	1.825618	-1.238921
C	-5.810743	3.171561	-1.684101
H	-6.589892	4.799464	-2.874614
H	-8.568549	3.540004	-3.674184
H	-8.895131	1.169812	-2.965803
H	-7.304676	0.093196	-1.441963
C	-8.446039	0.173142	2.209968
C	-7.363530	0.523525	1.433810
C	-6.401346	-0.445508	1.032811
C	-6.575250	-1.791360	1.490752
C	-7.711531	-2.123915	2.277058
C	-8.632850	-1.165515	2.628816
H	-9.161751	0.934658	2.508061
H	-7.231998	1.555520	1.130400
H	-7.830776	-3.153810	2.605356
H	-9.495019	-1.428898	3.235335
C	1.745794	-1.548123	2.285072
C	3.832214	-0.060169	-1.516210
C	4.365415	0.275740	-0.177298
C	3.523082	1.199136	0.600285
C	2.200086	1.398910	0.253105
C	2.488840	0.228575	-1.837341
H	0.673922	0.902376	-1.038062
N	1.700585	0.850214	-0.892922
C	1.963262	-0.083267	-3.122962
C	2.826802	-0.735586	-3.982722
C	4.583633	-0.741510	-2.523410
H	2.480251	-1.003612	-4.979697
C	-3.473569	-3.537421	0.146193
C	-2.670166	-4.078863	1.178127
C	-3.434416	-4.095904	-1.154108
C	-2.631626	-3.523472	2.498641
C	-1.842266	-5.233862	0.908375
C	-2.590268	-5.239859	-1.415823
C	-4.222353	-3.587821	-2.236435
C	-1.859703	-4.084222	3.482780
H	-3.211485	-2.632200	2.705505
C	-1.056160	-5.789984	1.966544
C	-1.828460	-5.784333	-0.376791

C	-2.567314	-5.802345	-2.730674
H	-4.868243	-2.735530	-2.058462
C	-4.169692	-4.154059	-3.483082
C	-1.066692	-5.238302	3.219209
H	-1.849319	-3.643415	4.476189
H	-0.448421	-6.665845	1.750685
H	-1.213212	-6.660778	-0.572589
C	-3.330819	-5.276470	-3.737611
H	-1.928250	-6.663921	-2.910612
H	-4.774376	-3.745121	-4.287941
H	-0.467855	-5.673629	4.014961
H	-3.304551	-5.713766	-4.732118
C	-2.482883	4.076778	-0.064362
C	-1.511173	4.421475	-1.034423
C	-2.360652	4.546861	1.265255
C	-1.557293	3.941105	-2.383979
C	-0.409635	5.283841	-0.666361
C	-1.241313	5.386931	1.625786
C	-3.319319	4.243014	2.284442
C	-0.613896	4.315474	-3.305534
H	-2.348858	3.257812	-2.667146
C	0.552406	5.650696	-1.660312
C	-0.303470	5.739714	0.650728
C	-1.125401	5.855476	2.972105
H	-4.174485	3.626104	2.032711
C	-3.173758	4.714000	3.562933
C	0.452403	5.189304	-2.944699
H	-0.676081	3.939788	-4.323540
H	1.368201	6.307138	-1.366663
H	0.530813	6.382295	0.924472
C	-2.060603	5.528882	3.916477
H	-0.273609	6.481774	3.226680
H	-3.914817	4.464478	4.317577
H	1.186865	5.478967	-3.691632
H	-1.961477	5.891791	4.936009
C	5.859161	0.564727	-0.076646
C	6.768163	-0.264601	0.577650
C	6.331238	1.776447	-0.637934
C	8.113869	0.074939	0.699118
H	6.421112	-1.187906	1.023869
C	7.674857	2.134382	-0.516486
C	8.567698	1.280507	0.151488
H	8.803137	-0.581873	1.218242
H	8.042600	3.062561	-0.935478
C	1.192144	2.159839	1.067074
H	0.413108	1.471641	1.415599
H	1.653139	2.654744	1.917653
H	0.698307	2.918167	0.453095
C	0.575754	0.305002	-3.564013
H	-0.210379	-0.144985	-2.951256
H	0.423997	1.389567	-3.497991
H	0.422299	0.005423	-4.605471
N	4.099749	-1.080414	-3.703919
O	5.853334	-1.057619	-2.224536
C	6.606509	-1.804982	-3.185461

H	6.182329	-2.804716	-3.303819
H	6.624279	-1.294766	-4.151801
H	7.610339	-1.870077	-2.763443
C	4.105730	2.060334	1.697656
N	4.807913	1.438759	2.691436
H	5.332552	2.054614	3.299775
H	5.139108	0.484713	2.618600
O	3.925001	3.275370	1.709553
O	5.408916	2.535010	-1.285314
C	5.722555	3.888415	-1.592260
H	4.799874	4.321461	-1.980592
H	6.030084	4.432548	-0.691782
H	6.506404	3.957398	-2.357274
C	9.946911	1.655440	0.270503
N	11.065855	1.959832	0.366732

R-forming TS Conformation 2

B3LYP/6-31G(d) Energy = -4490.492683
M06-2X/6-311G(d,p)-SMD(tetrahydrofuran) Energy = -4489.999261
M06-2X/6-311G(d,p)-SMD(tetrahydrofuran)-derived Free Energy (Quasiharmonic) = -4488.902281

Frequencies (Top 3 out of 468)

1. -1177.9165 cm^{-1}
2. 5.2781 cm^{-1}
3. 7.3777 cm^{-1}

B3LYP/6-31G(d) Molecular Geometry in Cartesian Coordinates

N	-1.142013	0.652623	1.914839
C	-1.674060	-0.504166	2.410456
C	-3.048124	-0.574443	2.570749
C	-3.209611	1.800807	1.891965
C	-3.874647	0.524630	2.093484
H	-4.241653	0.109281	0.815314
H	-0.106890	0.664027	1.730693
C	-3.681912	-1.756006	3.179757
O	-4.961567	-1.513087	3.547582
O	-3.159155	-2.853208	3.333414
C	-3.995101	3.057014	1.897104
O	-5.280797	2.831381	2.268559
O	-3.572361	4.170819	1.646756
C	-5.671187	-2.632112	4.107268
H	-5.785245	-3.420186	3.358516
H	-5.137776	-3.029918	4.973419
H	-6.644934	-2.239337	4.400008
C	-6.099250	4.002766	2.392962
H	-7.082995	3.639716	2.692126
H	-5.690979	4.676192	3.151665
H	-6.159769	4.536946	1.442292
C	-0.675062	-1.567733	2.776772
H	-0.781426	-2.443788	2.130609
H	0.342294	-1.183402	2.684579

H	-0.847814	-1.920976	3.796420
C	-0.991678	3.020398	1.413563
H	0.042623	2.722235	1.233505
H	-1.385051	3.554229	0.547303
H	-1.010540	3.732221	2.244885
P	2.058451	-0.069967	0.134357
O	1.510181	0.605229	1.367991
O	1.140352	-0.684542	-0.885419
O	3.042926	0.961854	-0.713878
O	3.162138	-1.167969	0.697989
H	-4.869262	0.587878	2.518512
C	4.120825	-1.682244	-0.151234
C	5.170230	-0.868717	-0.559765
C	5.004393	-3.573545	-1.342710
C	4.034529	-3.062131	-0.503435
H	4.969430	-4.626066	-1.611703
C	5.287581	0.518984	-0.025507
C	4.223097	1.403610	-0.149379
C	4.322094	2.779946	0.211920
C	5.506114	3.215323	0.772782
H	5.608842	4.263106	1.043053
C	6.968127	-3.297511	-2.817759
C	7.935451	-2.498992	-3.381562
C	7.997477	-1.127152	-3.040597
C	7.115310	-0.587171	-2.130840
C	6.114707	-1.387582	-1.511911
C	6.031290	-2.766278	-1.890181
H	6.894796	-4.349702	-3.082711
H	8.642512	-2.913313	-4.094992
H	8.746306	-0.490941	-3.504836
H	7.172828	0.467585	-1.888582
C	8.672824	0.559991	1.634865
C	7.552221	0.091157	0.985383
C	6.478999	0.962422	0.647066
C	6.585573	2.336852	1.034694
C	7.762731	2.793277	1.687508
C	8.789802	1.927041	1.980501
H	9.473654	-0.129371	1.888809
H	7.477281	-0.961058	0.737321
H	7.828068	3.843333	1.962785
H	9.682685	2.284560	2.485991
C	-1.835411	1.820957	1.729468
C	-3.653927	0.869001	-1.166201
C	-4.332487	-0.276633	-0.519365
C	-3.583090	-1.539132	-0.580839
C	-2.234796	-1.543761	-0.870012
C	-2.288918	0.778196	-1.513722
H	-0.571900	-0.407688	-1.290015
N	-1.607390	-0.386340	-1.232555
C	-1.630623	1.843839	-2.186146
C	-2.401362	2.964191	-2.435674
C	-4.277275	2.119947	-1.445721
H	-1.960705	3.805692	-2.967545
C	3.218284	3.763179	-0.019001
C	2.577971	4.367261	1.090901

C	2.887271	4.165834	-1.335960
C	2.826880	3.964439	2.444039
C	1.617809	5.426860	0.873230
C	1.897572	5.199421	-1.539825
C	3.512740	3.607810	-2.497041
C	2.214232	4.590664	3.497937
H	3.499796	3.135020	2.625585
C	1.009183	6.060933	2.002461
C	1.300377	5.809384	-0.432727
C	1.565581	5.595280	-2.873865
H	4.272302	2.845453	-2.368471
C	3.169086	4.016105	-3.759373
C	1.302871	5.663623	3.279234
H	2.418519	4.261092	4.513119
H	0.301247	6.866030	1.820154
H	0.573138	6.603232	-0.591473
C	2.178159	5.020430	-3.955259
H	0.818078	6.373994	-3.007876
H	3.658255	3.571949	-4.622018
H	0.835301	6.155324	4.128214
H	1.920479	5.333474	-4.963590
C	2.962969	-3.964259	0.020798
C	2.042090	-4.553052	-0.881506
C	2.926236	-4.303572	1.395887
C	1.997546	-4.221013	-2.275504
C	1.088801	-5.526906	-0.396221
C	1.941740	-5.248151	1.873135
C	3.850045	-3.770384	2.351247
C	1.121059	-4.838905	-3.129395
H	2.665198	-3.454650	-2.650550
C	0.195995	-6.152896	-1.322912
C	1.058530	-5.839859	0.965272
C	1.907938	-5.580374	3.264015
H	4.612336	-3.077654	2.014853
C	3.788379	-4.117988	3.675217
C	0.214969	-5.829788	-2.652956
H	1.110978	-4.566484	-4.181423
H	-0.500321	-6.897022	-0.942649
H	0.336899	-6.570169	1.326600
C	2.800880	-5.030818	4.143437
H	1.156094	-6.288323	3.605213
H	4.500950	-3.694159	4.377586
H	-0.465601	-6.316541	-3.346443
H	2.766250	-5.292905	5.197523
C	-5.835173	-0.436255	-0.697652
C	-6.700193	-0.590916	0.382546
C	-6.364897	-0.547887	-2.006548
C	-8.065404	-0.817621	0.208183
H	-6.305082	-0.552426	1.390470
C	-7.726841	-0.773432	-2.197817
C	-8.579836	-0.902946	-1.088275
H	-8.721825	-0.931218	1.063971
H	-8.141215	-0.859694	-3.194160
C	-1.366568	-2.770208	-0.930506
H	-0.352176	-2.534222	-0.597872

H	-1.769630	-3.588574	-0.335610
H	-1.285414	-3.118624	-1.967196
C	-0.206846	1.754163	-2.670317
H	0.517697	1.644496	-1.859396
H	-0.060980	0.891043	-3.331717
H	0.056552	2.656432	-3.229166
N	-3.687994	3.120733	-2.068193
O	-5.551580	2.277669	-1.028474
C	-6.203322	3.501767	-1.386846
H	-5.669693	4.361873	-0.973871
H	-6.254960	3.611124	-2.473927
H	-7.205806	3.422356	-0.963321
C	-4.325386	-2.865188	-0.553930
N	-4.195743	-3.634336	0.563435
H	-3.591705	-3.408443	1.344306
H	-4.619358	-4.552930	0.537630
O	-5.005718	-3.218557	-1.509472
O	-5.478562	-0.419405	-3.025048
C	-5.870612	-0.837971	-4.326648
H	-6.227609	-1.873976	-4.310280
H	-6.644112	-0.182468	-4.747106
H	-4.971826	-0.768759	-4.941371
C	-9.980959	-1.127875	-1.295475
N	-11.118976	-1.305602	-1.461046

R-forming TS Conformation 3

B3LYP/6-31G(d) Energy = -4490.490766
M06-2X/6-311G(d,p)-SMD(tetrahydrofuran) Energy = -4489.998649
M06-2X/6-311G(d,p)-SMD(tetrahydrofuran)-derived Free Energy (Quasiharmonic) = -4488.899884

Frequencies (Top 3 out of 468)

1. -1261.8556 cm^{-1}
2. 6.3875 cm^{-1}
3. 8.2418 cm^{-1}

B3LYP/6-31G(d) Molecular Geometry in Cartesian Coordinates

N	1.122451	-1.605779	1.152749
C	1.638226	-2.286270	0.082157
C	3.009215	-2.471825	0.026227
C	3.191782	-1.468634	2.279761
C	3.856605	-1.827374	1.029836
H	4.281268	-0.662245	0.459575
H	0.111799	-1.338141	1.132694
C	3.719741	-3.301402	-0.974333
O	2.908992	-3.874884	-1.892124
O	4.927945	-3.467865	-0.965233
C	4.054408	-1.365620	3.478321
O	3.382486	-1.226713	4.640017
O	5.274133	-1.433005	3.440585
C	3.565762	-4.654905	-2.904517
H	4.129734	-3.998852	-3.572837

H	4.239260	-5.386629	-2.452759
H	2.763906	-5.151981	-3.451663
C	4.199828	-1.124410	5.817584
H	4.851878	-0.249427	5.755532
H	3.499649	-1.022706	6.646962
H	4.814074	-2.020219	5.937749
C	0.604382	-2.770215	-0.896912
H	0.863429	-2.481185	-1.916085
H	0.544812	-3.861783	-0.878321
H	-0.380046	-2.373941	-0.643096
C	0.962612	-0.789411	3.421776
H	0.855883	-1.576903	4.175284
H	1.420230	0.068371	3.916180
H	-0.030432	-0.522708	3.054593
P	-2.032876	0.169372	0.062712
O	-1.399451	-0.554335	1.225715
O	-1.181334	0.762044	-1.024325
O	-3.131939	-0.819552	-0.696420
O	-3.037899	1.301765	0.730055
H	4.848067	-2.258827	1.130960
C	-4.022782	1.859175	-0.064299
C	-5.144658	1.100918	-0.372045
C	-4.860251	3.759250	-1.271879
C	-3.869205	3.213422	-0.481375
H	-4.770827	4.794099	-1.592439
C	-5.279319	-0.269823	0.199678
C	-4.266850	-1.199912	-0.004828
C	-4.376362	-2.556298	0.419290
C	-5.507799	-2.926855	1.117619
H	-5.615245	-3.959176	1.440888
C	-6.951749	3.569655	-2.576091
C	-8.014632	2.822659	-3.027447
C	-8.143458	1.472270	-2.624948
C	-7.228296	0.903339	-1.766998
C	-6.127960	1.652066	-1.263375
C	-5.981254	3.007455	-1.703102
H	-6.828690	4.603569	-2.889985
H	-8.747560	3.260703	-3.699408
H	-8.970742	0.875788	-3.000295
H	-7.338027	-0.134904	-1.475952
C	-8.488990	-0.128447	2.170516
C	-7.421804	0.275346	1.399125
C	-6.414664	-0.646222	0.996490
C	-6.527281	-2.000746	1.447815
C	-7.649060	-2.389322	2.229265
C	-8.614723	-1.476295	2.582710
H	-9.239987	0.597706	2.470044
H	-7.337403	1.313744	1.100854
H	-7.721251	-3.425153	2.552568
H	-9.465385	-1.782141	3.185548
C	1.819468	-1.280479	2.284587
C	3.749700	0.162444	-1.557314
C	4.311036	0.602645	-0.267366
C	3.430533	1.452468	0.541261
C	2.090014	1.585401	0.216595

C	2.392346	0.392870	-1.865233
H	0.570275	1.037405	-1.061495
N	1.601890	1.021916	-0.928184
C	1.864303	0.020043	-3.133887
C	2.739237	-0.638117	-3.978960
C	4.508257	-0.547311	-2.540439
H	2.391128	-0.947806	-4.963056
C	-3.344149	-3.595692	0.107600
C	-3.272350	-4.146444	-1.194544
C	-2.524535	-4.105826	1.142696
C	-4.073878	-3.668015	-2.280382
C	-2.378633	-5.252489	-1.454601
C	-1.648533	-5.225207	0.875142
C	-2.515191	-3.551510	2.464169
C	-3.988226	-4.225980	-3.528953
H	-4.757087	-2.845064	-2.103599
C	-2.322276	-5.807575	-2.771549
C	-1.603070	-5.769710	-0.411851
C	-0.847240	-5.751916	1.936997
H	-3.130752	-2.684001	2.668924
C	-1.726316	-4.082102	3.451426
C	-3.100208	-5.310282	-3.781978
H	-4.604292	-3.839855	-4.336457
H	-1.645889	-6.640451	-2.950284
H	-0.951010	-6.619443	-0.606350
C	-0.886880	-5.203417	3.190458
H	-0.203434	-6.602122	1.723017
H	-1.737129	-3.641617	4.444922
H	-3.048146	-5.741672	-4.778063
H	-0.275136	-5.615575	3.988733
C	-2.699050	4.052076	-0.073635
C	-1.739096	4.435973	-1.040690
C	-2.600178	4.526126	1.256429
C	-1.761875	3.955153	-2.390729
C	-0.673529	5.340756	-0.668454
C	-1.516618	5.410207	1.620895
C	-3.549020	4.183194	2.272387
C	-0.829887	4.365852	-3.308462
H	-2.525616	3.242287	-2.677285
C	0.277984	5.744177	-1.658407
C	-0.590119	5.800136	0.649151
C	-1.423877	5.882261	2.967777
H	-4.377819	3.532442	2.017593
C	-3.426624	4.659034	3.551496
C	0.201162	5.279223	-2.943117
H	-0.874225	3.989077	-4.327067
H	1.067533	6.430617	-1.361806
H	0.217328	6.474863	0.925948
C	-2.348266	5.517833	3.908939
H	-0.598433	6.541619	3.225496
H	-4.159387	4.379423	4.303704
H	0.927927	5.596411	-3.686322
H	-2.266882	5.883644	4.928996
C	5.752630	1.118423	-0.362343
C	5.921148	2.298661	-1.093062

C	6.897853	0.518367	0.202324
C	7.173079	2.873256	-1.300466
H	5.043791	2.777773	-1.516097
C	8.161674	1.073078	-0.004224
C	8.301214	2.249079	-0.760115
H	7.274061	3.787565	-1.874570
H	9.045481	0.609697	0.414574
C	1.050997	2.284473	1.046286
H	0.311662	1.552081	1.390999
H	1.495668	2.796899	1.893944
H	0.512756	3.018362	0.439648
C	0.462495	0.349714	-3.576748
H	-0.303414	-0.114418	-2.949553
H	0.272393	1.429210	-3.532473
H	0.316128	0.022531	-4.610808
N	4.023137	-0.943487	-3.701398
O	5.783391	-0.826597	-2.231143
C	6.562451	-1.566310	-3.177965
H	7.576510	-1.554925	-2.775917
H	6.197260	-2.593390	-3.245869
H	6.531523	-1.094773	-4.163075
C	3.989438	2.221347	1.712148
N	4.888056	1.562729	2.503759
H	5.363194	2.126678	3.195469
H	5.277743	0.651531	2.295883
O	3.650356	3.374886	1.962496
O	6.687536	-0.585627	0.962944
C	7.801192	-1.256804	1.546357
H	7.372479	-2.066972	2.132682
H	8.466466	-1.654776	0.771093
H	8.364098	-0.588807	2.209774
C	9.607742	2.803685	-0.964811
N	10.669827	3.249042	-1.130382

R-forming TS Conformation 4

B3LYP/6-31G(d) Energy = -4490.492190
M06-2X/6-311G(d,p)-SMD(tetrahydrofuran) Energy = -4489.998000
M06-2X/6-311G(d,p)-SMD(tetrahydrofuran)-derived Free Energy (Quasiharmonic) = -4488.900401

Frequencies (Top 3 out of 468)

1. -1224.2385 cm^{-1}
2. 6.6663 cm^{-1}
3. 8.2582 cm^{-1}

B3LYP/6-31G(d) Molecular Geometry in Cartesian Coordinates

N	0.990337	-1.843446	1.111391
C	1.487722	-2.505713	0.021785
C	2.850538	-2.742897	-0.039612
C	3.066965	-1.790558	2.229268
C	3.716350	-2.167080	0.982421
H	4.187340	-0.967524	0.438527

H	-0.009350	-1.528669	1.097061
C	3.418255	-3.582441	-1.115830
O	4.767845	-3.681097	-1.006356
O	2.789389	-4.151148	-1.990022
C	3.960343	-1.640330	3.394038
O	3.342924	-1.667053	4.588021
O	5.176123	-1.524088	3.285265
C	5.395256	-4.517157	-1.989855
H	5.065635	-5.553915	-1.877039
H	5.147003	-4.174811	-2.997094
H	6.466101	-4.433711	-1.802648
C	4.196274	-1.496438	5.734876
H	4.691078	-0.522662	5.699789
H	3.533726	-1.559662	6.597938
H	4.952954	-2.283644	5.768143
C	0.466058	-2.925239	-0.993624
H	-0.504908	-2.477880	-0.774144
H	0.784235	-2.655010	-2.001589
H	0.353861	-4.013293	-0.987822
C	0.879733	-1.060495	3.401420
H	-0.092740	-0.713009	3.046769
H	0.717855	-1.870214	4.121348
H	1.394702	-0.255972	3.928219
P	-2.029036	0.124430	0.069631
O	-1.447673	-0.646784	1.230480
O	-1.134551	0.672797	-1.005146
O	-3.182169	-0.787499	-0.702984
O	-2.966421	1.308962	0.746594
H	4.687789	-2.639509	1.092043
C	-3.927322	1.923652	-0.034087
C	-5.089315	1.228159	-0.342226
C	-4.675096	3.879656	-1.211908
C	-3.708338	3.273790	-0.435214
H	-4.535221	4.912780	-1.519767
C	-5.292252	-0.140653	0.213797
C	-4.331183	-1.119226	-0.009368
C	-4.507491	-2.473966	0.397159
C	-5.652421	-2.794823	1.097903
H	-5.811619	-3.824580	1.407737
C	-6.781385	3.813226	-2.504289
C	-7.883521	3.126862	-2.957719
C	-8.078282	1.779740	-2.571033
C	-7.188222	1.154392	-1.726063
C	-6.048323	1.839974	-1.220408
C	-5.835541	3.191481	-1.644738
H	-6.607701	4.843389	-2.806404
H	-8.597193	3.609869	-3.619519
H	-8.936762	1.230607	-2.948557
H	-7.348512	0.119282	-1.447599
C	-8.479050	0.139757	2.206874
C	-7.396845	0.498257	1.433966
C	-6.440965	-0.468469	1.012954
C	-6.620526	-1.821457	1.447164
C	-7.756376	-2.161948	2.230677
C	-8.671665	-1.205189	2.602228

H	-9.189965	0.899532	2.520531
H	-7.260684	1.534801	1.148528
H	-7.880196	-3.196934	2.540778
H	-9.533539	-1.474949	3.206386
C	1.701403	-1.564298	2.245521
C	3.824041	-0.039151	-1.539782
C	4.346127	0.271636	-0.189135
C	3.493521	1.178108	0.597124
C	2.164025	1.350695	0.260331
C	2.468025	0.214896	-1.845989
H	0.639974	0.835982	-1.026874
N	1.667988	0.791028	-0.881534
C	1.944851	-0.067429	-3.138744
C	2.835159	-0.619379	-4.040630
C	4.599021	-0.618208	-2.590508
H	2.495737	-0.852651	-5.048318
C	-3.529994	-3.557738	0.062046
C	-3.489551	-4.084901	-1.251211
C	-2.729865	-4.125196	1.082409
C	-4.276214	-3.551395	-2.322134
C	-2.644223	-5.221473	-1.539423
C	-1.903219	-5.274537	0.786255
C	-2.692231	-3.601860	2.416015
C	-4.219420	-4.086298	-3.582349
H	-4.924319	-2.705079	-2.124685
C	-2.615253	-5.749620	-2.868212
C	-1.885728	-5.792387	-0.512326
C	-1.119902	-5.857419	1.832109
H	-3.270971	-2.714852	2.643487
C	-1.922819	-4.187288	3.387668
C	-3.376795	-5.199461	-3.863472
H	-4.822985	-3.658315	-4.378159
H	-1.970918	-6.602357	-3.069008
H	-1.267871	-6.661739	-0.729604
C	-1.131467	-5.336200	3.097764
H	-0.513050	-6.728557	1.595635
H	-1.913140	-3.770258	4.391359
H	-3.344743	-5.609522	-4.869279
H	-0.534694	-5.791640	3.883825
C	-2.495833	4.048288	-0.023969
C	-1.523164	4.396437	-0.991752
C	-2.367623	4.502281	1.310625
C	-1.575081	3.930870	-2.346308
C	-0.414581	5.246404	-0.616113
C	-1.241717	5.330340	1.678441
C	-3.326343	4.193114	2.328159
C	-0.629795	4.307155	-3.265096
H	-2.372605	3.257136	-2.635560
C	0.548842	5.616313	-1.607612
C	-0.303005	5.687184	0.705658
C	-1.120236	5.782852	3.029754
H	-4.185922	3.584644	2.070938
C	-3.175193	4.648482	3.611659
C	0.443689	5.168897	-2.896524
H	-0.695919	3.942258	-4.286744

H	1.369926	6.263548	-1.308251
H	0.536547	6.320405	0.985046
C	-2.055943	5.451922	3.972153
H	-0.263785	6.400530	3.289768
H	-3.916413	4.395276	4.364917
H	1.179484	5.460467	-3.641416
H	-1.952562	5.802730	4.995508
C	5.837252	0.563203	-0.068876
C	6.741862	-0.278911	0.574664
C	6.311759	1.790213	-0.594577
C	8.085502	0.061391	0.718769
H	6.391988	-1.211116	0.999508
C	7.652278	2.149607	-0.448916
C	8.541181	1.281616	0.206610
H	8.771156	-0.604668	1.230963
H	8.020679	3.089685	-0.840106
C	1.148869	2.094139	1.081202
H	0.651109	2.855967	0.474832
H	0.373422	1.397444	1.419923
H	1.604159	2.582770	1.938651
C	0.533657	0.261628	-3.550567
H	-0.218792	-0.255815	-2.949455
H	0.321463	1.332009	-3.439534
H	0.385074	-0.006367	-4.601130
N	4.129194	-0.895463	-3.789981
O	5.893843	-0.893126	-2.330588
C	6.713604	-1.299573	-3.430921
H	6.363426	-2.241559	-3.860162
H	6.715413	-0.538274	-4.216405
H	7.713153	-1.416233	-3.008919
C	4.067460	2.051100	1.689880
N	4.776336	1.443156	2.686937
H	5.111019	0.489642	2.623325
H	5.292814	2.066937	3.294102
O	3.874994	3.264467	1.693296
O	5.394708	2.559799	-1.235156
C	5.689052	3.932592	-1.469726
H	5.966786	4.436352	-0.536937
H	6.486520	4.053031	-2.213945
H	4.766230	4.367136	-1.855815
C	9.918130	1.656502	0.349392
N	11.035748	1.959349	0.464025

R-forming TS Conformation 5

B3LYP/6-31G(d) Energy = -4490.492113
M06-2X/6-311G(d,p)-SMD(tetrahydrofuran) Energy = -4489.998347
M06-2X/6-311G(d,p)-SMD(tetrahydrofuran)-derived Free Energy (Quasiharmonic) = -4488.900596

Frequencies (Top 3 out of 468)

1. -1211.6423 cm^{-1}
2. 6.0570 cm^{-1}
3. 9.4052 cm^{-1}

B3LYP/6-31G(d) Molecular Geometry in Cartesian Coordinates

N	1.042118	-1.786326	1.129268
C	1.512560	-2.448774	0.026030
C	2.872220	-2.694222	-0.059300
C	3.137595	-1.750161	2.214118
C	3.754183	-2.137769	0.959180
H	4.184787	-0.910346	0.429328
H	0.043724	-1.467083	1.132530
C	3.545172	-3.491065	-1.111761
O	2.713404	-3.975427	-2.058326
O	4.747236	-3.698956	-1.115835
C	3.964056	-1.569652	3.423769
O	5.147666	-2.214679	3.334735
O	3.653405	-0.906859	4.404756
C	3.344644	-4.708332	-3.123115
H	2.525666	-5.113020	-3.718634
H	3.957774	-4.033603	-3.725756
H	3.966557	-5.511725	-2.721858
C	6.024222	-2.063940	4.466894
H	5.542477	-2.437074	5.373580
H	6.907331	-2.656131	4.227802
H	6.288455	-1.012771	4.606846
C	0.449896	-2.849233	-0.958434
H	-0.520084	-2.445698	-0.665108
H	0.700311	-2.504903	-1.962990
H	0.366193	-3.938299	-1.004864
C	0.984534	-0.987451	3.420557
H	1.270878	0.039112	3.666404
H	-0.082747	-1.007875	3.196871
H	1.194054	-1.585392	4.311322
P	-2.009095	0.144898	0.093583
O	-1.390938	-0.592502	1.256905
O	-1.150748	0.684774	-1.014902
O	-3.165185	-0.800928	-0.634512
O	-2.949235	1.329479	0.767244
H	4.729572	-2.612152	1.010142
C	-3.931691	1.916977	-0.007708
C	-5.090026	1.201097	-0.281046
C	-4.725395	3.839066	-1.211028
C	-3.737466	3.261396	-0.439372
H	-4.604372	4.867443	-1.541855
C	-5.266529	-0.158406	0.305943
C	-4.298298	-1.130519	0.085139
C	-4.453825	-2.479083	0.519701
C	-5.583439	-2.799057	1.245290
H	-5.726279	-3.824418	1.576932
C	-6.852803	3.720239	-2.464340
C	-7.953903	3.011181	-2.884052
C	-8.124630	1.669925	-2.466817
C	-7.211964	1.072987	-1.625432
C	-6.072162	1.782791	-1.154159
C	-5.884185	3.127842	-1.609408
H	-6.697644	4.746116	-2.790140

H	-8.685270	3.471725	-3.542515
H	-8.982649	1.102686	-2.817663
H	-7.354298	0.041938	-1.323220
C	-8.421375	0.126400	2.348514
C	-7.356473	0.481515	1.550451
C	-6.397756	-0.483076	1.130947
C	-6.555775	-1.829476	1.593049
C	-7.674381	-2.167012	2.402232
C	-8.593093	-1.212851	2.771895
H	-9.134688	0.884587	2.660509
H	-7.236156	1.513923	1.243716
H	-7.781921	-3.197208	2.733588
H	-9.441518	-1.479997	3.395884
C	1.775826	-1.498617	2.245766
C	3.799702	-0.015357	-1.526355
C	4.317024	0.325162	-0.186257
C	3.448691	1.224101	0.589467
C	2.130584	1.405187	0.235199
C	2.452915	0.246995	-1.855094
H	0.619240	0.897741	-1.072747
N	1.645011	0.855266	-0.918825
C	1.944714	-0.081582	-3.144233
C	2.827339	-0.720754	-3.994178
C	4.569666	-0.693070	-2.519489
H	2.493935	-0.998540	-4.992953
C	-3.469975	-3.559078	0.190611
C	-2.645838	-4.094532	1.209112
C	-3.446680	-4.115897	-1.110793
C	-2.591912	-3.540635	2.529823
C	-1.811467	-5.241271	0.924771
C	-2.596328	-5.251718	-1.387249
C	-4.256518	-3.614087	-2.179731
C	-1.798569	-4.094458	3.500703
H	-3.178521	-2.656341	2.747564
C	-1.003238	-5.790907	1.969679
C	-1.812843	-5.790041	-0.361157
C	-2.589190	-5.812646	-2.702920
H	-4.907465	-2.768099	-1.990442
C	-4.218696	-4.178653	-3.427691
C	-0.998622	-5.240393	3.222830
H	-1.775663	-3.654452	4.494155
H	-0.390852	-6.660824	1.743112
H	-1.192500	-6.660370	-0.567882
C	-3.373614	-5.292921	-3.696909
H	-1.945138	-6.668074	-2.894053
H	-4.840069	-3.774633	-4.222255
H	-0.382630	-5.670306	4.008338
H	-3.359243	-5.728983	-4.692198
C	-2.529972	4.062336	-0.065239
C	-1.581728	4.407756	-1.058113
C	-2.386322	4.547322	1.256878
C	-1.647837	3.909664	-2.400397
C	-0.485093	5.289501	-0.722373
C	-1.272261	5.407695	1.585106
C	-3.318675	4.239748	2.299135

C	-0.727489	4.284582	-3.344698
H	-2.434928	3.211691	-2.659014
C	0.451923	5.657005	-1.739689
C	-0.360005	5.763184	0.586871
C	-1.136434	5.893227	2.923608
H	-4.169020	3.606968	2.071753
C	-3.153629	4.726134	3.569464
C	0.333280	5.177467	-3.015801
H	-0.803745	3.894506	-4.356273
H	1.264212	6.328413	-1.471208
H	0.467764	6.424547	0.835187
C	-2.046524	5.562351	3.890794
H	-0.290691	6.537175	3.153565
H	-3.874423	4.472977	4.342238
H	1.049410	5.467117	-3.780256
H	-1.932409	5.938446	4.903948
C	5.799921	0.621905	-0.021102
C	6.621108	-0.086071	0.853051
C	6.340051	1.738861	-0.702920
C	7.953277	0.270042	1.059914
H	6.219608	-0.934968	1.393507
C	7.667644	2.113030	-0.499047
C	8.476705	1.374974	0.380847
H	8.578857	-0.296663	1.740870
H	8.086244	2.970678	-1.010442
C	1.131835	2.194714	1.033494
H	0.610197	2.915492	0.398326
H	0.371493	1.522202	1.449056
H	1.608430	2.729073	1.855091
C	0.551750	0.277004	-3.593534
H	-0.228102	-0.178982	-2.976677
H	0.381099	1.359664	-3.541595
H	0.404735	-0.038316	-4.631252
N	4.104279	-1.045020	-3.702952
O	5.837916	-0.992471	-2.194753
C	6.616332	-1.734843	-3.139672
H	6.205802	-2.739974	-3.260549
H	6.643753	-1.228051	-4.107631
H	7.613994	-1.786196	-2.701499
C	4.048830	2.148228	1.639279
N	3.952597	1.772766	2.941897
H	4.370790	2.386609	3.629068
H	3.580085	0.889861	3.265497
O	4.573400	3.205643	1.306542
O	5.500894	2.389247	-1.546260
C	5.798976	3.737177	-1.895128
H	4.920822	4.102619	-2.429874
H	5.958751	4.338884	-0.994359
H	6.672613	3.800917	-2.556354
C	9.842930	1.762862	0.580648
N	10.952349	2.073912	0.743290

R-forming TS Conformation 6

B3LYP/6-31G(d) Energy = -4490.487073
M06-2X/6-311G(d,p)-SMD(tetrahydrofuran) Energy = -4489.995357
M06-2X/6-311G(d,p)-SMD(tetrahydrofuran)-derived Free Energy (Quasiharmonic) = -4488.897337

Frequencies (Top 3 out of 468)

1. -1219.2657 cm^{-1}
2. 5.9410 cm^{-1}
3. 7.0534 cm^{-1}

B3LYP/6-31G(d) Molecular Geometry in Cartesian Coordinates

N	-1.257587	0.559176	1.713831
C	-1.965197	1.727613	1.585972
C	-3.339484	1.677415	1.747083
C	-3.142169	-0.707191	2.353461
C	-3.987695	0.378872	1.873969
H	-4.321957	0.003721	0.595228
H	-0.223087	0.591850	1.541245
C	-4.240673	2.848477	1.872199
O	-3.610071	4.043265	1.796645
O	-5.438675	2.751493	2.070848
C	-3.713182	-1.870361	3.059784
O	-4.823198	-1.560144	3.763695
O	-3.240463	-3.000704	3.064535
C	-4.441336	5.192483	2.020657
H	-3.773130	6.051074	1.948674
H	-5.228882	5.255288	1.265976
H	-4.902888	5.144400	3.010507
C	-5.387400	-2.644308	4.523834
H	-6.238652	-2.215409	5.052950
H	-5.709795	-3.451933	3.861673
H	-4.653300	-3.035356	5.232129
C	-1.116075	2.939281	1.320346
H	-0.098977	2.638405	1.062302
H	-1.536544	3.543756	0.516070
H	-1.066820	3.578226	2.207476
C	-0.751143	-1.664613	2.544899
H	-0.953356	-2.082336	3.533953
H	-0.791363	-2.503994	1.844460
H	0.254783	-1.241782	2.521452
P	2.042399	-0.084789	0.034001
O	1.416826	0.575486	1.238228
O	1.192988	-0.703974	-1.041024
O	3.058527	0.968333	-0.750044
O	3.129703	-1.171326	0.649474
H	-4.998801	0.417904	2.263212
C	4.137762	-1.666172	-0.153401
C	5.197368	-0.836252	-0.497557
C	5.103750	-3.532802	-1.319054
C	4.085465	-3.042247	-0.525980
H	5.094073	-4.581984	-1.602767
C	5.267902	0.547143	0.055615
C	4.199572	1.418989	-0.116976
C	4.259628	2.791911	0.265300

C	5.406038	3.236746	0.893256
H	5.479559	4.282518	1.180303
C	7.139749	-3.218192	-2.684340
C	8.126700	-2.402795	-3.186604
C	8.155479	-1.034694	-2.826746
C	7.220339	-0.515115	-1.958993
C	6.197209	-1.333422	-1.403562
C	6.149285	-2.708227	-1.801612
H	7.092140	-4.267718	-2.965202
H	8.875010	-2.800938	-3.866366
H	8.920848	-0.385102	-3.243009
H	7.253337	0.537115	-1.701653
C	8.558299	0.614025	1.895612
C	7.480433	0.137713	1.182419
C	6.415183	0.998670	0.795870
C	6.482289	2.370036	1.202635
C	7.616716	2.834728	1.921956
C	8.638260	1.978733	2.260684
H	9.353708	-0.067563	2.185052
H	7.433046	-0.912596	0.919959
H	7.653165	3.882535	2.210842
H	9.498085	2.342297	2.816645
C	-1.771328	-0.615905	2.188166
C	-3.582566	0.820677	-1.336874
C	-4.299749	-0.351134	-0.787770
C	-3.537899	-1.606794	-0.807905
C	-2.183986	-1.604300	-1.080081
C	-2.216822	0.734533	-1.677455
H	-0.514927	-0.469347	-1.484886
N	-1.551238	-0.445497	-1.437873
C	-1.549910	1.826755	-2.298496
C	-2.304522	2.972846	-2.470434
C	-4.183733	2.104184	-1.509883
H	-1.855253	3.834741	-2.960934
C	3.156312	3.764003	-0.010680
C	2.459272	4.353362	1.072858
C	2.882946	4.174987	-1.338360
C	2.646536	3.939551	2.432585
C	1.504792	5.410197	0.819721
C	1.896156	5.203374	-1.579637
C	3.565774	3.631302	-2.473594
C	1.984865	4.555099	3.462882
H	3.313032	3.110455	2.637573
C	0.845215	6.034381	1.925632
C	1.245767	5.801639	-0.496397
C	1.622678	5.607346	-2.924498
H	4.324560	2.873748	-2.316178
C	3.276976	4.046744	-3.747240
C	1.082428	5.628565	3.211536
H	2.143693	4.217723	4.483556
H	0.147992	6.843008	1.717753
H	0.524532	6.595182	-0.682691
C	2.288252	5.045063	-3.980849
H	0.876891	6.382301	-3.087142
H	3.808346	3.612864	-4.589840

H	0.578685	6.114797	4.042929
H	2.074355	5.363715	-4.997583
C	2.997232	-3.962016	-0.071391
C	2.128398	-4.542633	-1.028783
C	2.894757	-4.324986	1.294145
C	2.149337	-4.184939	-2.416938
C	1.161798	-5.533481	-0.608784
C	1.897217	-5.286939	1.705051
C	3.763179	-3.798970	2.303918
C	1.321299	-4.794067	-3.323757
H	2.826668	-3.405454	-2.744546
C	0.321471	-6.149824	-1.589375
C	1.066906	-5.870993	0.743869
C	1.796778	-5.643204	3.086747
H	4.534068	-3.093076	2.018050
C	3.638269	-4.169615	3.617167
C	0.402378	-5.801382	-2.910629
H	1.359408	-4.501586	-4.369721
H	-0.386593	-6.905733	-1.257326
H	0.335035	-6.613717	1.055812
C	2.638201	-5.100106	4.019419
H	1.036210	-6.364268	3.377441
H	4.309844	-3.750874	4.361755
H	-0.239457	-6.280079	-3.645374
H	2.553294	-5.380779	5.065866
C	-5.757079	-0.472571	-1.220417
C	-6.032061	-0.314709	-2.583353
C	-6.828412	-0.788982	-0.362495
C	-7.316361	-0.421984	-3.101170
H	-5.208228	-0.104264	-3.257338
C	-8.128751	-0.889173	-0.862714
C	-8.375524	-0.703303	-2.230758
H	-7.498722	-0.295343	-4.162598
H	-8.959375	-1.121365	-0.209495
C	-1.304675	-2.823165	-1.146801
H	-1.195381	-3.143630	-2.190120
H	-0.300180	-2.584466	-0.786777
H	-1.714916	-3.657792	-0.582306
C	-0.136045	1.741024	-2.811750
H	-0.019651	0.914620	-3.524233
H	0.133576	2.668754	-3.324072
H	0.599984	1.570867	-2.022297
N	-3.581264	3.136600	-2.067947
O	-5.438931	2.250285	-1.045127
C	-6.097367	3.497283	-1.287749
H	-6.248344	3.650582	-2.360639
H	-7.053651	3.416492	-0.770772
H	-5.513125	4.331888	-0.893342
C	-4.281824	-2.931853	-0.753212
N	-4.018303	-3.752881	0.299968
H	-4.530670	-4.624639	0.329353
H	-3.513124	-3.469777	1.132623
O	-5.055595	-3.248028	-1.649075
O	-6.530480	-0.996717	0.952534
C	-7.606673	-1.126200	1.874306

H	-8.287014	-0.268529	1.812474
H	-8.169005	-2.053510	1.705624
H	-7.143598	-1.150097	2.859575
C	-9.717160	-0.806751	-2.725494
N	-10.808046	-0.885106	-3.122963

R-forming TS Conformation 7

B3LYP/6-31G(d) Energy = -4490.492777
M06-2X/6-311G(d,p)-SMD(tetrahydrofuran) Energy = -4489.999951
M06-2X/6-311G(d,p)-SMD(tetrahydrofuran)-derived Free Energy (Quasiharmonic) = -4488.903323

Frequencies (Top 3 out of 468)

1. -1184.0032 cm^{-1}
2. 3.9882 cm^{-1}
3. 6.8989 cm^{-1}

B3LYP/6-31G(d) Molecular Geometry in Cartesian Coordinates

N	-1.119286	0.772971	1.933804
C	-1.762677	1.947322	1.635952
C	-3.138680	1.987031	1.768201
C	-3.079619	-0.317776	2.670843
C	-3.856900	0.759494	2.078275
H	-4.272354	0.283581	0.830631
H	-0.084709	0.725615	1.752331
C	-3.991946	3.186359	1.610182
O	-3.307221	4.341977	1.472200
O	-5.211155	3.139290	1.635128
C	-3.739708	-1.367710	3.471484
O	-5.102755	-1.239109	3.442377
O	-3.191560	-2.239004	4.117692
C	-4.109904	5.526132	1.343741
H	-3.401219	6.354365	1.321999
H	-4.690083	5.497054	0.417803
H	-4.792561	5.621902	2.191549
C	-5.812539	-2.161038	4.289340
H	-5.507642	-2.031851	5.330568
H	-6.867794	-1.916086	4.164900
H	-5.612356	-3.192002	3.986724
C	-0.850254	3.066477	1.220480
H	0.152103	2.682497	1.022816
H	-1.230591	3.576261	0.334764
H	-0.776862	3.816585	2.014223
C	-0.746235	-1.364523	3.015729
H	-1.055250	-2.358256	2.684950
H	0.266532	-1.153246	2.667196
H	-0.750272	-1.395152	4.109876
P	2.042865	-0.105353	0.148113
O	1.525184	0.599386	1.379089
O	1.096541	-0.689119	-0.863054
O	3.058837	0.886807	-0.711769
O	3.109412	-1.236675	0.713469

H	-4.847414	0.913353	2.494065
C	4.059862	-1.777673	-0.129213
C	5.137070	-0.994824	-0.525093
C	4.897441	-3.690742	-1.317978
C	3.933884	-3.152404	-0.488570
H	4.830864	-4.740111	-1.593204
C	5.284308	0.392055	0.003603
C	4.245886	1.303598	-0.143155
C	4.380120	2.681838	0.198799
C	5.568055	3.091439	0.771096
H	5.698041	4.139839	1.026881
C	6.888467	-3.473109	-2.765654
C	7.887446	-2.704110	-3.315091
C	7.987425	-1.335531	-2.970244
C	7.110552	-0.770125	-2.070843
C	6.077875	-1.540266	-1.466122
C	5.956771	-2.914931	-1.848805
H	6.785647	-4.521936	-3.034055
H	8.590496	-3.138865	-4.020289
H	8.761547	-0.721877	-3.423280
H	7.197987	0.281959	-1.826076
C	8.646290	0.358504	1.708946
C	7.522086	-0.086303	1.048934
C	6.478253	0.810639	0.687023
C	6.617844	2.186038	1.060521
C	7.798277	2.616908	1.724712
C	8.796427	1.725608	2.041558
H	9.424159	-0.349788	1.981406
H	7.420699	-1.138593	0.810609
H	7.889516	3.667820	1.989205
H	9.691915	2.063775	2.555681
C	-1.699975	-0.308961	2.530303
C	-3.708351	0.872068	-1.239344
C	-4.394940	-0.195681	-0.470352
C	-3.664688	-1.471230	-0.431930
C	-2.312536	-1.516100	-0.711613
C	-2.335353	0.744551	-1.548166
H	-0.634435	-0.437371	-1.210440
N	-1.669895	-0.398664	-1.157728
C	-1.651804	1.749739	-2.286009
C	-2.404347	2.856239	-2.634488
C	-4.327842	2.084273	-1.672814
H	-1.936857	3.658615	-3.203051
C	3.312088	3.694384	-0.070022
C	2.682896	4.351341	1.016086
C	3.006269	4.071263	-1.401117
C	2.897568	3.971093	2.381586
C	1.768898	5.442749	0.760140
C	2.058168	5.135222	-1.643030
C	3.618316	3.456743	-2.540570
C	2.297926	4.646322	3.412463
H	3.532079	3.118333	2.591047
C	1.176168	6.130317	1.866393
C	1.478781	5.801172	-0.558852
C	1.749068	5.502796	-2.990468

H	4.348442	2.671372	-2.384468
C	3.297218	3.838961	-3.816875
C	1.436644	5.752007	3.156234
H	2.474415	4.332225	4.437612
H	0.509763	6.963884	1.655728
H	0.787966	6.620910	-0.747236
C	2.345246	4.872493	-4.049707
H	1.032098	6.304432	-3.153235
H	3.774596	3.351130	-4.662284
H	0.982283	6.284872	3.987457
H	2.104756	5.163686	-5.068750
C	2.824960	-4.021978	0.012671
C	1.887638	-4.555347	-0.906400
C	2.767151	-4.386221	1.380156
C	1.862830	-4.190699	-2.292529
C	0.894531	-5.500925	-0.446230
C	1.745639	-5.303277	1.832492
C	3.702485	-3.904370	2.351367
C	0.964012	-4.751836	-3.161862
H	2.562894	-3.444334	-2.648403
C	-0.020645	-6.067903	-1.388580
C	0.846006	-5.842141	0.908048
C	1.690759	-5.661086	3.216269
H	4.490739	-3.232162	2.033020
C	3.619108	-4.274924	3.667959
C	0.015543	-5.714179	-2.710433
H	0.967525	-4.454006	-4.207066
H	-0.753065	-6.785411	-1.026379
H	0.093470	-6.549938	1.250315
C	2.596661	-5.161069	4.111936
H	0.911047	-6.347123	3.539125
H	4.340852	-3.889831	4.383250
H	-0.685393	-6.153173	-3.415266
H	2.544803	-5.441505	5.160563
C	-5.903083	-0.336430	-0.648557
C	-6.832331	0.113576	0.289314
C	-6.380202	-0.991273	-1.810105
C	-8.198406	-0.108574	0.133106
H	-6.488930	0.680433	1.145721
C	-7.745147	-1.237103	-1.972168
C	-8.654722	-0.799452	-0.996233
H	-8.904471	0.248594	0.874867
H	-8.115291	-1.755391	-2.847845
C	-1.450343	-2.741399	-0.593641
H	-1.490777	-3.317980	-1.523029
H	-0.410961	-2.456253	-0.416676
H	-1.797095	-3.400942	0.202609
C	-0.217497	1.619111	-2.728438
H	0.486329	1.545866	-1.895935
H	-0.064968	0.718669	-3.335953
H	0.068138	2.486105	-3.330925
N	-3.704507	3.040253	-2.336434
O	-5.629181	2.251369	-1.377460
C	-6.267255	3.449626	-1.824740
H	-5.794829	4.331923	-1.385117

H	-6.224111	3.531948	-2.914428
H	-7.299523	3.361672	-1.483735
C	-4.365240	-2.789137	-0.208635
N	-5.193740	-2.885383	0.870339
H	-5.759705	-3.720613	0.935206
H	-5.430770	-2.098467	1.458054
O	-4.181720	-3.742718	-0.960489
O	-5.442462	-1.343432	-2.726044
C	-5.775540	-2.303362	-3.723603
H	-4.842183	-2.519144	-4.244434
H	-6.156254	-3.223435	-3.266500
H	-6.506927	-1.904627	-4.437808
C	-10.054954	-1.057594	-1.168165
N	-11.191130	-1.268137	-1.304934

R-forming TS Conformation 8

B3LYP/6-31G(d) Energy = -4490.489261
M06-2X/6-311G(d,p)-SMD(tetrahydrofuran) Energy = -4489.997803
M06-2X/6-311G(d,p)-SMD(tetrahydrofuran)-derived Free Energy (Quasiharmonic) = -4488.900420

Frequencies (Top 3 out of 468)

1. -1195.5114 cm^{-1}
2. 6.0589 cm^{-1}
3. 9.6241 cm^{-1}

B3LYP/6-31G(d) Molecular Geometry in Cartesian Coordinates

N	1.045505	-1.826657	1.110367
C	1.511606	-2.470643	-0.005416
C	2.869826	-2.720441	-0.094644
C	3.138147	-1.818872	2.199306
C	3.748961	-2.180954	0.932312
H	4.188688	-0.948694	0.419629
H	0.052745	-1.493824	1.111540
C	3.538742	-3.510246	-1.155898
O	2.702992	-3.979335	-2.107195
O	4.738559	-3.726484	-1.161773
C	4.066228	-1.754723	3.350582
O	3.535846	-1.191903	4.467585
O	5.217600	-2.148962	3.308725
C	3.330065	-4.708606	-3.177163
H	3.954771	-4.035383	-3.769417
H	3.939883	-5.524001	-2.781522
H	2.508825	-5.096683	-3.780609
C	4.418618	-1.146727	5.606176
H	5.306401	-0.552197	5.377225
H	3.836387	-0.685699	6.404253
H	4.728404	-2.155852	5.886976
C	0.446633	-2.848110	-0.996328
H	0.701184	-2.492392	-1.995803
H	0.354520	-3.935822	-1.057486
H	-0.520535	-2.440919	-0.698947

C	0.958050	-1.098358	3.415302
H	-0.084642	-0.970021	3.121113
H	1.018188	-1.823698	4.232159
H	1.332440	-0.149521	3.806648
P	-1.982584	0.151851	0.065108
O	-1.367643	-0.582288	1.232713
O	-1.123883	0.676919	-1.049767
O	-3.148114	-0.789781	-0.652658
O	-2.911073	1.347636	0.735614
H	4.726119	-2.652449	0.990604
C	-3.893412	1.937547	-0.037608
C	-5.057605	1.227975	-0.302458
C	-4.679944	3.859461	-1.246013
C	-3.692653	3.278892	-0.475776
H	-4.553869	4.885636	-1.581759
C	-5.241273	-0.127390	0.292030
C	-4.280959	-1.107543	0.072482
C	-4.444809	-2.453185	0.513179
C	-5.574403	-2.761863	1.243672
H	-5.723923	-3.784811	1.579792
C	-6.812972	3.748427	-2.490391
C	-7.920271	3.044538	-2.902416
C	-8.097892	1.706347	-2.478310
C	-7.185718	1.107426	-1.637800
C	-6.039503	1.812029	-1.174362
C	-5.844756	3.153743	-1.636602
H	-6.652535	4.771755	-2.821588
H	-8.651284	3.506711	-3.560115
H	-8.960877	1.142998	-2.823174
H	-7.333431	0.078675	-1.330396
C	-8.386631	0.189278	2.344607
C	-7.321913	0.533157	1.541373
C	-6.371876	-0.440247	1.122516
C	-6.538275	-1.783557	1.590720
C	-7.656585	-2.109419	2.405092
C	-8.566871	-1.146921	2.774027
H	-9.093252	0.954011	2.655848
H	-7.195058	1.563352	1.229846
H	-7.770776	-3.137464	2.740877
H	-9.415186	-1.405228	3.401872
C	1.775664	-1.570521	2.239137
C	3.829967	-0.047898	-1.538543
C	4.335518	0.281144	-0.190421
C	3.462549	1.184818	0.575338
C	2.152085	1.381798	0.207326
C	2.488867	0.226480	-1.881389
H	0.651458	0.888341	-1.116838
N	1.674852	0.840610	-0.954355
C	1.991165	-0.095131	-3.176045
C	2.878208	-0.736551	-4.019664
C	4.606250	-0.725323	-2.527282
H	2.552811	-1.009108	-5.022581
C	-3.468969	-3.540659	0.185160
C	-3.448408	-4.097983	-1.116104
C	-2.648828	-4.081271	1.204266

C	-4.255650	-3.592485	-2.185266
C	-2.603899	-5.238261	-1.392190
C	-1.820141	-5.232189	0.920131
C	-2.594075	-3.529449	2.525782
C	-4.220609	-4.157725	-3.432980
H	-4.902489	-2.743278	-1.996426
C	-2.599323	-5.799603	-2.707710
C	-1.823254	-5.780434	-0.365993
C	-1.016419	-5.787344	1.965552
H	-3.176743	-2.642572	2.743768
C	-1.806369	-4.089652	3.497669
C	-3.381052	-5.276238	-3.701873
H	-4.839978	-3.750916	-4.227674
H	-1.959499	-6.658241	-2.898509
H	-1.207007	-6.653654	-0.572597
C	-1.011724	-5.239267	3.219791
H	-0.408012	-6.659950	1.738824
H	-1.784855	-3.652225	4.492400
H	-3.368760	-5.712593	-4.697042
H	-0.400184	-5.674515	4.005863
C	-2.478905	4.073819	-0.108964
C	-1.530660	4.407593	-1.105812
C	-2.328887	4.564904	1.210194
C	-1.603821	3.903449	-2.445450
C	-0.426953	5.283195	-0.777129
C	-1.208191	5.419335	1.531325
C	-3.261157	4.269596	2.256101
C	-0.683236	4.267270	-3.393829
H	-2.396547	3.209901	-2.698703
C	0.510164	5.638894	-1.798482
C	-0.295514	5.762837	0.529292
C	-1.066495	5.911698	2.866750
H	-4.116360	3.641520	2.033885
C	-3.090442	4.762287	3.523271
C	0.384805	5.154012	-3.071919
H	-0.764834	3.872893	-4.403297
H	1.327923	6.305763	-1.535383
H	0.537390	6.419754	0.772183
C	-1.977080	5.592899	3.837565
H	-0.216219	6.551663	3.091139
H	-3.811819	4.519011	4.298699
H	1.101124	5.434643	-3.839524
H	-1.858943	5.974946	4.848066
C	5.818292	0.569679	-0.003592
C	6.621830	-0.136175	0.889006
C	6.372843	1.681128	-0.683139
C	7.953466	0.215288	1.109786
H	6.211901	-0.973241	1.441790
C	7.700164	2.047407	-0.467730
C	8.493430	1.309738	0.427513
H	8.562362	-0.348929	1.807514
H	8.130936	2.898829	-0.979339
C	1.151773	2.175668	0.999770
H	0.618211	2.879891	0.356347
H	0.401092	1.503846	1.433814

H	1.629924	2.730883	1.806742
C	0.603967	0.273568	-3.635257
H	-0.183574	-0.175662	-3.022911
H	0.441499	1.357729	-3.586671
H	0.460799	-0.042472	-4.673295
N	4.150358	-1.068820	-3.717410
O	5.868470	-1.032943	-2.191180
C	6.652732	-1.778543	-3.129024
H	6.235099	-2.779562	-3.258446
H	6.696591	-1.268106	-4.094504
H	7.644008	-1.839740	-2.678050
C	4.052649	2.071397	1.658776
N	3.926799	1.639614	2.945180
H	3.496823	0.763364	3.198750
H	4.323479	2.217260	3.674655
O	4.594950	3.134089	1.383490
O	5.543776	2.337979	-1.534042
C	5.875627	3.668875	-1.912402
H	6.053426	4.287857	-1.026277
H	6.750624	3.696236	-2.574479
H	5.006963	4.045449	-2.455038
C	9.859248	1.692537	0.640119
N	10.967709	2.002615	0.811237

R-forming TS Conformation 9

B3LYP/6-31G(d) Energy = -4490.487949
M06-2X/6-311G(d,p)-SMD(tetrahydrofuran) Energy = -4489.999830
M06-2X/6-311G(d,p)-SMD(tetrahydrofuran)-derived Free Energy (Quasiharmonic) = -4488.901328

Frequencies (Top 3 out of 468)

1. -1222.8382 cm^{-1}
2. 6.8255 cm^{-1}
3. 8.3210 cm^{-1}

B3LYP/6-31G(d) Molecular Geometry in Cartesian Coordinates

N	1.213212	-0.367419	1.727827
C	1.626406	0.925470	1.905558
C	2.982025	1.165122	2.040470
C	3.373654	-1.277253	2.027631
C	3.920426	0.059389	1.857677
H	4.268316	0.135073	0.530124
H	0.184284	-0.514967	1.570080
C	3.472281	2.504270	2.427942
O	4.558408	2.439854	3.230736
O	2.975367	3.578246	2.109447
C	4.263602	-2.404110	2.386895
O	5.504566	-1.968957	2.723300
O	3.951744	-3.581206	2.432036
C	5.045873	3.708895	3.702535
H	5.887656	3.471974	4.353775
H	5.366843	4.334758	2.865856

H	4.266099	4.232735	4.260375
C	6.410233	-2.985641	3.173050
H	7.331507	-2.461816	3.430521
H	6.005055	-3.502346	4.047311
H	6.595635	-3.718681	2.384464
C	0.508838	1.926865	1.985555
H	-0.301224	1.538864	2.609402
H	0.849970	2.888023	2.361018
H	0.082828	2.081179	0.986997
C	1.289797	-2.787263	1.885240
H	1.730566	-3.477748	1.163456
H	1.390918	-3.267194	2.863183
H	0.229364	-2.646501	1.667599
P	-2.051516	0.013999	0.040724
O	-1.437366	-0.651655	1.246366
O	-1.188376	0.636544	-1.023144
O	-3.072163	-1.026891	-0.752507
O	-3.136353	1.109330	0.649620
H	4.915100	0.213898	2.256839
C	-4.145935	1.605327	-0.148778
C	-5.207459	0.777807	-0.493916
C	-5.117147	3.480149	-1.298575
C	-4.095798	2.983951	-0.513073
H	-5.109258	4.531392	-1.574754
C	-5.279444	-0.606024	0.057943
C	-4.210787	-1.477701	-0.112701
C	-4.264572	-2.846513	0.283707
C	-5.408558	-3.290092	0.916090
H	-5.476430	-4.332982	1.214922
C	-7.157039	3.172891	-2.660152
C	-8.144478	2.359781	-3.165202
C	-8.170901	0.989228	-2.814473
C	-7.232941	0.464902	-1.952679
C	-6.209082	1.280524	-1.394702
C	-6.163563	2.658050	-1.783657
H	-7.111397	4.224352	-2.934112
H	-8.895042	2.761677	-3.840264
H	-8.936694	0.341657	-3.233082
H	-7.263882	-0.588995	-1.701872
C	-8.568623	-0.670288	1.901549
C	-7.492476	-0.195405	1.184792
C	-6.425379	-1.055808	0.801822
C	-6.488311	-2.424722	1.217677
C	-7.620908	-2.887996	1.940715
C	-8.644784	-2.032947	2.274752
H	-9.365518	0.011029	2.187591
H	-7.448295	0.853556	0.916639
H	-7.653892	-3.934004	2.236420
H	-9.503218	-2.395550	2.833502
C	2.011200	-1.470668	1.868905
C	3.488836	-1.052021	-1.188430
C	4.267820	0.175628	-0.893666
C	3.571052	1.433471	-1.187008
C	2.209910	1.445749	-1.434245
C	2.135295	-0.961478	-1.573554

H	0.489258	0.320895	-1.576185
N	1.524955	0.273397	-1.568186
C	1.417585	-2.106284	-2.012325
C	2.096868	-3.307580	-1.927968
C	4.005175	-2.379212	-1.080322
H	1.608022	-4.220236	-2.263539
C	-3.152643	-3.813592	0.023362
C	-2.904139	-4.273633	-1.292092
C	-2.420874	-4.345210	1.113011
C	-3.622540	-3.786157	-2.430840
C	-1.905138	-5.294252	-1.515646
C	-1.444138	-5.385699	0.878597
C	-2.597732	-3.890902	2.460987
C	-3.360839	-4.251247	-3.693188
H	-4.386940	-3.031650	-2.284257
C	-1.663356	-5.753537	-2.849072
C	-1.210175	-5.827761	-0.426256
C	-0.740934	-5.946785	1.991125
H	-3.285293	-3.075477	2.650236
C	-1.900471	-4.452432	3.498687
C	-2.365544	-5.246850	-3.910026
H	-3.918759	-3.859683	-4.539400
H	-0.910957	-6.524761	-2.999023
H	-0.474749	-6.611623	-0.597280
C	-0.967203	-5.503406	3.266378
H	-0.019329	-6.737108	1.797968
H	-2.053059	-4.086775	4.510626
H	-2.173999	-5.606333	-4.917546
H	-0.429503	-5.941771	4.102948
C	-3.002211	3.896750	-0.057637
C	-2.135839	4.480663	-1.014862
C	-2.887094	4.245382	1.310850
C	-2.176039	4.145244	-2.408374
C	-1.153680	5.454159	-0.590685
C	-1.876686	5.192345	1.725013
C	-3.754447	3.718199	2.320893
C	-1.350729	4.758519	-3.314941
H	-2.868628	3.381569	-2.741304
C	-0.314769	6.073593	-1.570722
C	-1.044535	5.775387	0.764730
C	-1.764379	5.534487	3.109456
H	-4.534199	3.023031	2.032857
C	-3.617878	4.074885	3.636926
C	-0.413921	5.746828	-2.896251
H	-1.404702	4.483775	-4.364988
H	0.405276	6.816258	-1.234449
H	-0.300269	6.504222	1.079785
C	-2.606088	4.991345	4.041969
H	-0.995038	6.245432	3.402076
H	-4.289274	3.655863	4.381478
H	0.224841	6.229853	-3.630939
H	-2.512491	5.261338	5.090492
C	5.735347	0.124602	-1.299708
C	6.053012	-0.436885	-2.540727
C	6.785882	0.671675	-0.535996

C	7.356912	-0.504160	-3.015888
H	5.248743	-0.827136	-3.155402
C	8.104256	0.600373	-0.988293
C	8.392599	0.009391	-2.227791
H	7.571977	-0.943541	-3.983693
H	8.917783	1.009178	-0.403890
C	1.388103	2.685770	-1.684647
H	1.825610	3.278324	-2.493611
H	0.364097	2.422745	-1.953481
H	1.347912	3.330639	-0.803859
C	0.042839	-2.025007	-2.623004
H	-0.723515	-1.683141	-1.924190
H	0.030185	-1.324030	-3.467618
H	-0.260331	-3.006444	-2.996755
N	3.344616	-3.461904	-1.442462
O	5.242787	-2.519352	-0.561095
C	5.789433	-3.846321	-0.534537
H	5.207897	-4.487064	0.132188
H	5.799696	-4.281843	-1.536991
H	6.808122	-3.720891	-0.163825
C	4.370995	2.704035	-1.409404
N	4.050424	3.779314	-0.640770
H	3.466061	3.727933	0.186542
H	4.581040	4.624735	-0.803163
O	5.231589	2.758358	-2.281305
O	6.441958	1.274244	0.640003
C	7.478331	1.809844	1.452006
H	6.982263	2.184909	2.346059
H	8.203033	1.037056	1.737243
H	8.000852	2.629598	0.943344
C	9.752630	-0.058962	-2.675832
N	10.858864	-0.119128	-3.031956

R-forming TS Conformation 10

B3LYP/6-31G(d) Energy = -4490.495255
M06-2X/6-311G(d,p)-SMD(tetrahydrofuran) Energy = -4490.000283
M06-2X/6-311G(d,p)-SMD(tetrahydrofuran)-derived Free Energy (Quasiharmonic) = -4488.902754

Frequencies (Top 3 out of 468)

1. -1186.6354 cm^{-1}
2. 6.2989 cm^{-1}
3. 7.5562 cm^{-1}

B3LYP/6-31G(d) Molecular Geometry in Cartesian Coordinates

N	-1.094140	0.848101	1.964623
C	-1.690901	-0.215863	2.577777
C	-3.070098	-0.199850	2.710620
C	-3.098634	2.078208	1.738512
C	-3.832501	0.875321	2.096238
H	-4.291499	0.344715	0.878177
H	-0.061353	0.779708	1.780884

C	-3.870493	-1.210947	3.424576
O	-3.161829	-2.074545	4.173941
O	-5.094068	-1.254421	3.352290
C	-3.932281	3.282973	1.517091
O	-3.224518	4.418207	1.330930
O	-5.151170	3.254320	1.529219
C	-3.929043	-3.090957	4.845001
H	-4.666780	-2.636871	5.510480
H	-4.441134	-3.722109	4.114446
H	-3.201864	-3.672150	5.411961
C	-4.000217	5.604734	1.098974
H	-4.761040	5.726267	1.873140
H	-3.285118	6.427556	1.124401
H	-4.483149	5.553769	0.119330
C	-0.739330	-1.277551	3.061993
H	-0.710641	-1.285838	4.156123
H	-1.062676	-2.271585	2.746826
H	0.267396	-1.084211	2.686435
C	-0.789345	3.111590	1.182184
H	-1.150512	3.594264	0.273874
H	-0.718744	3.887880	1.950571
H	0.210992	2.709224	1.013082
P	2.021363	-0.094553	0.145519
O	1.541384	0.592486	1.402182
O	1.044958	-0.632846	-0.861468
O	3.068877	-1.264415	0.670037
O	3.047166	0.893002	-0.706911
H	-4.819210	1.048287	2.515923
C	4.244738	1.279328	-0.138476
C	5.267436	0.347157	-0.014338
C	5.602577	3.025618	0.802798
C	4.405735	2.648729	0.226555
H	5.753017	4.066753	1.076385
C	5.094641	-1.024798	-0.573169
C	4.006554	-1.799274	-0.190324
C	3.858383	-3.163208	-0.581098
C	4.808976	-3.696648	-1.428532
H	4.724559	-4.738091	-1.727897
C	7.826193	2.492317	1.741059
C	8.809474	1.577353	2.036341
C	8.634445	0.220169	1.676404
C	7.500428	-0.191381	1.011576
C	6.471213	0.730825	0.672211
C	6.636274	2.095831	1.072430
H	7.936963	3.535934	2.026152
H	9.712435	1.889299	2.553950
H	9.400873	-0.506904	1.931263
H	7.379910	-1.236585	0.751795
C	7.927774	-1.351947	-3.040104
C	7.064281	-0.794410	-2.123051
C	6.022217	-1.562388	-1.531710
C	5.877668	-2.925323	-1.946562
C	6.795922	-3.476158	-2.881246
C	7.804431	-2.710111	-3.417139
H	8.709419	-0.739920	-3.482262

H	7.169709	0.249973	-1.853766
H	6.675084	-4.516474	-3.174125
H	8.497137	-3.138816	-4.136123
C	-1.721546	2.018681	1.623072
C	-3.792045	0.898919	-1.192495
C	-4.451437	-0.166467	-0.397794
C	-3.712479	-1.437762	-0.372758
C	-2.365696	-1.469081	-0.673810
C	-2.419277	0.793609	-1.503906
H	-0.703081	-0.372950	-1.186412
N	-1.736398	-0.342036	-1.118313
C	-1.754018	1.815260	-2.236062
C	-2.525285	2.912916	-2.572858
C	-4.437781	2.096163	-1.626425
H	-2.070109	3.730045	-3.130498
C	2.741994	-4.031560	-0.093935
C	1.792264	-4.529881	-1.019887
C	2.692238	-4.435214	1.262776
C	1.758219	-4.122552	-2.393835
C	0.796478	-5.482251	-0.579967
C	1.670059	-5.360999	1.695614
C	3.637534	-3.986751	2.240367
C	0.846534	-4.649001	-3.271372
H	2.461340	-3.371279	-2.732933
C	-0.131901	-6.012533	-1.530675
C	0.758989	-5.865980	0.763235
C	1.627344	-5.762672	3.067787
H	4.425321	-3.307628	1.935963
C	3.564421	-4.397425	3.545656
C	-0.105519	-5.617389	-2.840936
H	0.842311	-4.318600	-4.306678
H	-0.866671	-6.735445	-1.184352
H	0.006653	-6.581602	1.089585
C	2.543378	-5.294546	3.970520
H	0.850055	-6.458775	3.375207
H	4.293933	-4.037329	4.266033
H	-0.817123	-6.027908	-3.552114
H	2.501958	-5.609999	5.009730
C	3.358002	3.686381	-0.025528
C	2.744073	4.340916	1.070666
C	3.057796	4.087561	-1.350875
C	2.951052	3.935864	2.430206
C	1.852814	5.454677	0.831518
C	2.131959	5.174474	-1.576232
C	3.653958	3.475534	-2.500028
C	2.364641	4.607131	3.471241
H	3.567867	3.067078	2.626421
C	1.273940	6.137270	1.948158
C	1.568786	5.837662	-0.481837
C	1.827259	5.566239	-2.917797
H	4.367813	2.673006	-2.356624
C	3.337448	3.881034	-3.770228
C	1.525839	5.733714	3.232029
H	2.534341	4.273907	4.491510
H	0.624508	6.987207	1.750375

H	0.894796	6.674023	-0.657655
C	2.406870	4.937214	-3.986838
H	1.126669	6.384607	-3.067991
H	3.801955	3.394157	-4.623318
H	1.081492	6.262447	4.071232
H	2.169394	5.246247	-5.001301
C	-5.959105	-0.332012	-0.542599
C	-6.869983	-0.006999	0.460600
C	-6.448701	-0.904223	-1.741907
C	-8.233973	-0.253184	0.319050
H	-6.514552	0.448593	1.375597
C	-7.810041	-1.170512	-1.892680
C	-8.704029	-0.844154	-0.859523
H	-8.925740	0.002557	1.114058
H	-8.190406	-1.620895	-2.800964
C	-1.498552	-2.693373	-0.587376
H	-0.458245	-2.410292	-0.411920
H	-1.838212	-3.369151	0.198818
H	-1.544317	-3.250636	-1.528335
C	-0.317046	1.710932	-2.677439
H	0.384933	1.618791	-1.844792
H	-0.154355	0.830017	-3.310645
H	-0.036644	2.597179	-3.254010
N	-3.829956	3.071512	-2.277639
O	-5.745648	2.222243	-1.345600
C	-6.411041	3.416623	-1.762393
H	-6.000556	4.288331	-1.246451
H	-6.317905	3.560948	-2.842121
H	-7.454061	3.269452	-1.479229
C	-4.410854	-2.768561	-0.196428
N	-5.169205	-2.947732	0.919827
H	-5.743480	-3.780065	0.943129
H	-5.379036	-2.209343	1.581334
O	-4.272547	-3.659451	-1.031758
O	-5.523805	-1.158616	-2.703580
C	-5.852038	-2.065183	-3.750895
H	-6.613829	-1.648308	-4.421827
H	-4.926492	-2.217067	-4.307446
H	-6.191533	-3.023497	-3.342508
C	-10.101954	-1.119935	-1.022754
N	-11.236045	-1.343954	-1.155789

R-forming TS Conformation 11

B3LYP/6-31G(d) Energy = -4490.496215
M06-2X/6-311G(d,p)-SMD(tetrahydrofuran) Energy = -4490.002659
M06-2X/6-311G(d,p)-SMD(tetrahydrofuran)-derived Free Energy (Quasiharmonic) = -4488.904211

Frequencies (Top 3 out of 468)

1. -1154.1846 cm^{-1}
2. 7.3727 cm^{-1}
3. 8.0344 cm^{-1}

B3LYP/6-31G(d) Molecular Geometry in Cartesian Coordinates

N	-1.163619	0.873712	1.966511
C	-1.717718	2.040923	1.516037
C	-3.094727	2.172137	1.567259
C	-3.210719	-0.008795	2.745036
C	-3.898237	1.033336	1.997355
H	-4.300790	0.403819	0.861116
H	-0.136954	0.745114	1.819283
C	-3.855424	3.391538	1.215534
O	-3.083069	4.462370	0.921381
O	-5.074450	3.435531	1.206967
C	-4.093236	-0.906503	3.529297
O	-3.444494	-1.824473	4.266640
O	-5.308061	-0.789311	3.534601
C	-3.790434	5.651561	0.538114
H	-3.032926	6.434411	0.484432
H	-4.258842	5.511089	-0.440026
H	-4.555550	5.902089	1.276338
C	-4.289369	-2.786883	4.920554
H	-4.847867	-3.351007	4.170521
H	-3.610686	-3.439475	5.470847
H	-4.984139	-2.289706	5.601861
C	-0.716740	3.056873	1.041839
H	0.274407	2.606131	0.969335
H	-1.004337	3.474302	0.076605
H	-0.662320	3.892714	1.746134
C	-0.955460	-1.185283	3.224418
H	-1.348794	-2.175317	2.988270
H	0.063971	-1.087047	2.843905
H	-0.931095	-1.106959	4.316125
P	2.013899	-0.127427	0.155706
O	1.498881	0.501022	1.425684
O	1.072360	-0.638896	-0.899794
O	3.043648	0.902987	-0.643583
O	3.065518	-1.308804	0.649952
H	-4.892241	1.289883	2.355695
C	4.000496	-1.813109	-0.230430
C	5.087496	-1.025891	-0.588475
C	4.792809	-3.660959	-1.548600
C	3.851165	-3.161440	-0.671269
H	4.706328	-4.690327	-1.886855
C	5.264087	0.323105	0.022281
C	4.244256	1.262649	-0.066962
C	4.416464	2.619990	0.336907
C	5.614504	2.970711	0.926836
H	5.772040	4.002299	1.230936
C	6.768738	-3.383070	-3.006856
C	7.776185	-2.597742	-3.516289
C	7.906231	-1.257269	-3.082519
C	7.050064	-0.736708	-2.137271
C	6.008952	-1.525980	-1.572873
C	5.858144	-2.869968	-2.043742
H	6.643438	-4.409957	-3.342279
H	8.463262	-2.997579	-4.257006

H	8.687408	-0.629402	-3.502770
H	7.160561	0.294827	-1.824097
C	8.632215	0.121334	1.702965
C	7.496108	-0.261021	1.024367
C	6.470338	0.677092	0.720026
C	6.642012	2.026302	1.168014
C	7.833629	2.393093	1.850264
C	8.812936	1.463727	2.112140
H	9.395791	-0.617680	1.930650
H	7.371039	-1.295601	0.726712
H	7.949219	3.425329	2.172473
H	9.717239	1.752806	2.640578
C	-1.836858	-0.112599	2.642455
C	-3.797993	0.777970	-1.258947
C	-4.418361	-0.249949	-0.398951
C	-3.611216	-1.456171	-0.232931
C	-2.272283	-1.473040	-0.544253
C	-2.421078	0.702625	-1.568450
H	-0.658549	-0.392972	-1.213881
N	-1.692783	-0.383442	-1.129760
C	-1.793720	1.716672	-2.345298
C	-2.601073	2.773910	-2.722451
C	-4.482665	1.937015	-1.734406
H	-2.173361	3.583950	-3.311259
C	3.382128	3.675034	0.102453
C	2.763018	4.307326	1.208310
C	3.101295	4.113568	-1.215389
C	2.947806	3.862006	2.558277
C	1.887315	5.437241	0.988743
C	2.192129	5.218021	-1.422283
C	3.701134	3.523061	-2.373880
C	2.351264	4.507671	3.609614
H	3.554670	2.982591	2.737997
C	1.298092	6.092545	2.116202
C	1.624722	5.859233	-0.316974
C	1.907260	5.648294	-2.756284
H	4.402734	2.707513	-2.244099
C	3.403287	3.965067	-3.636437
C	1.525627	5.647989	3.390978
H	2.501921	4.142957	4.622030
H	0.658938	6.953698	1.934207
H	0.962582	6.707949	-0.478085
C	2.489493	5.039166	-3.835239
H	1.218735	6.479286	-2.892375
H	3.870394	3.493846	-4.496933
H	1.071260	6.154390	4.238464
H	2.266938	5.377510	-4.843770
C	2.748459	-4.054346	-0.197022
C	1.775416	-4.512729	-1.118392
C	2.739085	-4.523867	1.139239
C	1.697041	-4.032546	-2.466701
C	0.800971	-5.495950	-0.699446
C	1.737186	-5.479571	1.553771
C	3.705920	-4.112190	2.111737
C	0.763466	-4.519412	-3.344154

H	2.383060	-3.256163	-2.783700
C	-0.149501	-5.985621	-1.650482
C	0.805091	-5.947651	0.622836
C	1.734705	-5.947230	2.905319
H	4.477656	-3.409470	1.820081
C	3.671071	-4.585725	3.397081
C	-0.165785	-5.520604	-2.938233
H	0.725752	-4.134630	-4.359835
H	-0.862509	-6.738817	-1.322772
H	0.068360	-6.685565	0.934618
C	2.670290	-5.513001	3.804782
H	0.970558	-6.662768	3.200251
H	4.414946	-4.251620	4.115232
H	-0.889892	-5.905147	-3.651905
H	2.658285	-5.876557	4.828815
C	-5.916249	-0.506882	-0.491272
C	-6.752864	-0.463229	0.622932
C	-6.479181	-0.881424	-1.735847
C	-8.121173	-0.717996	0.521043
H	-6.337222	-0.247568	1.599950
C	-7.844800	-1.119887	-1.858756
C	-8.672320	-1.028335	-0.724556
H	-8.752321	-0.671323	1.401546
H	-8.283897	-1.388144	-2.811392
C	-1.381902	-2.667129	-0.347091
H	-1.377500	-3.289747	-1.249946
H	-0.350929	-2.351820	-0.173498
H	-1.727154	-3.280912	0.485890
C	-0.349867	1.646426	-2.772090
H	0.345993	1.593195	-1.930239
H	-0.153206	0.757579	-3.384516
H	-0.090006	2.528460	-3.364725
N	-3.910316	2.904208	-2.427280
O	-5.791749	2.025255	-1.444147
C	-6.488892	3.217115	-1.821383
H	-6.115486	4.069751	-1.250107
H	-6.380770	3.413502	-2.891200
H	-7.531528	3.024330	-1.565111
C	-4.231298	-2.719717	0.326573
N	-4.766944	-3.536716	-0.624784
H	-5.306241	-4.335785	-0.316715
H	-4.902960	-3.195740	-1.566367
O	-4.209920	-3.015812	1.516031
O	-5.598483	-1.030191	-2.773862
C	-6.115547	-1.180619	-4.091741
H	-5.247304	-1.175307	-4.752546
H	-6.655509	-2.128972	-4.210001
H	-6.780941	-0.349197	-4.352723
C	-10.077975	-1.277570	-0.860648
N	-11.217225	-1.482523	-0.979345

R-forming TS Conformation 12

B3LYP/6-31G(d) Energy = -4490.490612

M06-2X/6-311G(d,p)-SMD(tetrahydrofuran) Energy = -4489.997232
M06-2X/6-311G(d,p)-SMD(tetrahydrofuran)-derived Free Energy (Quasiharmonic) = -4488.899774

Frequencies (Top 3 out of 468)

1. -1218.0418 cm^{-1}
2. 6.3158 cm^{-1}
3. 9.1048 cm^{-1}

B3LYP/6-31G(d) Molecular Geometry in Cartesian Coordinates

N	1.008705	-1.819953	1.073495
C	1.495126	-2.478397	-0.023169
C	2.857055	-2.717488	-0.098217
C	3.096786	-1.772304	2.173585
C	3.730160	-2.151330	0.922264
H	4.157920	-0.917172	0.399938
H	0.005592	-1.512833	1.073469
C	3.413383	-3.554344	-1.182886
O	4.761477	-3.671238	-1.078001
O	2.775252	-4.106504	-2.060918
C	3.910510	-1.595337	3.392078
O	5.100752	-2.231428	3.310251
O	3.586818	-0.943788	4.376155
C	5.375423	-4.502641	-2.074064
H	5.026616	-5.534865	-1.979162
H	5.135530	-4.138714	-3.075826
H	6.447254	-4.441780	-1.883647
C	5.963373	-2.081619	4.453334
H	5.474204	-2.463937	5.352128
H	6.853565	-2.665958	4.220935
H	6.218518	-1.029558	4.602898
C	0.462502	-2.897358	-1.027403
H	-0.506526	-2.452222	-0.795792
H	0.769127	-2.624909	-2.038241
H	0.352389	-3.985718	-1.022331
C	0.928000	-1.046241	3.373408
H	1.137421	-1.654188	4.257487
H	1.202850	-0.020533	3.634924
H	-0.137357	-1.073887	3.141984
P	-2.046174	0.110895	0.072525
O	-1.432824	-0.651826	1.222329
O	-1.181071	0.663237	-1.024529
O	-3.210781	-0.808842	-0.671928
O	-2.974943	1.292161	0.768436
H	4.708797	-2.612360	0.999500
C	-3.958573	1.897825	0.009204
C	-5.122857	1.193683	-0.269869
C	-4.747837	3.845456	-1.155946
C	-3.758957	3.248307	-0.400561
H	-4.622320	4.878635	-1.469733
C	-5.303639	-0.175745	0.292171
C	-4.341744	-1.148058	0.047050
C	-4.500573	-2.504349	0.455429
C	-5.627944	-2.833214	1.180465

H	-5.773981	-3.864284	1.492400
C	-6.883527	3.761446	-2.397843
C	-7.991178	3.066289	-2.823479
C	-8.167164	1.718777	-2.429193
C	-7.253107	1.101761	-1.603983
C	-6.106531	1.796593	-1.126765
C	-5.913393	3.148365	-1.559378
H	-6.724198	4.792010	-2.706410
H	-8.723662	3.542600	-3.469372
H	-9.030324	1.162655	-2.785179
H	-7.399482	0.066230	-1.319631
C	-8.446289	0.083148	2.356910
C	-7.383934	0.449092	1.560301
C	-6.432053	-0.511372	1.116605
C	-6.593798	-1.866042	1.552654
C	-7.709721	-2.214298	2.360978
C	-8.621987	-1.263447	2.754510
H	-9.154420	0.838248	2.687676
H	-7.260350	1.486818	1.273340
H	-7.820422	-3.250388	2.672323
H	-9.468369	-1.539107	3.377592
C	1.731770	-1.535410	2.197740
C	3.793071	0.020862	-1.544961
C	4.295286	0.330858	-0.190740
C	3.412470	1.204722	0.598195
C	2.089197	1.360022	0.249559
C	2.435838	0.250436	-1.863140
H	0.587017	0.844187	-1.065304
N	1.613342	0.808670	-0.907538
C	1.934362	-0.043376	-3.162652
C	2.844357	-0.582368	-4.052409
C	4.588359	-0.553817	-2.580438
H	2.520752	-0.822916	-5.063564
C	-3.521391	-3.579549	0.098898
C	-3.499026	-4.101351	-1.216939
C	-2.697599	-4.142506	1.102665
C	-4.310791	-3.572684	-2.271333
C	-2.646519	-5.227311	-1.524799
C	-1.863322	-5.281038	0.787028
C	-2.642847	-3.624812	2.437974
C	-4.271033	-4.102335	-3.534428
H	-4.964666	-2.734477	-2.058876
C	-2.635768	-5.750197	-2.855912
C	-1.863011	-5.793349	-0.513840
C	-1.055014	-5.859150	1.816471
H	-3.229288	-2.746716	2.680049
C	-1.849578	-4.205072	3.393384
C	-3.421341	-5.204942	-3.835080
H	-4.893720	-3.678249	-4.317483
H	-1.985792	-6.594969	-3.071647
H	-1.239276	-6.654687	-0.745765
C	-1.050036	-5.343303	3.084315
H	-0.442653	-6.722374	1.565558
H	-1.826549	-3.792240	4.398497
H	-3.403023	-5.610934	-4.842877

H	-0.434175	-5.794829	3.857783
C	-2.544324	4.035724	-0.020651
C	-1.597773	4.387990	-1.012673
C	-2.392112	4.502198	1.307131
C	-1.672431	3.907473	-2.360928
C	-0.494158	5.258266	-0.669938
C	-1.271515	5.351466	1.641997
C	-3.321847	4.185956	2.349130
C	-0.753046	4.287996	-3.303887
H	-2.465313	3.218293	-2.625343
C	0.441266	5.632596	-1.686232
C	-0.361038	5.714323	0.644747
C	-1.127467	5.818490	2.986223
H	-4.176561	3.560895	2.116803
C	-3.148729	4.654427	3.625115
C	0.314716	5.169813	-2.967756
H	-0.835407	3.910912	-4.319871
H	1.258778	6.295514	-1.412485
H	0.471822	6.367286	0.898267
C	-2.035542	5.480087	3.952734
H	-0.277116	6.454554	3.221150
H	-3.867677	4.395047	4.397543
H	1.029852	5.464347	-3.731294
H	-1.915158	5.842199	4.970261
C	5.774046	0.632992	-0.000936
C	6.585379	-0.087310	0.872175
C	6.319807	1.765925	-0.652135
C	7.913060	0.270308	1.106418
H	6.178749	-0.945171	1.394251
C	7.642145	2.141789	-0.420587
C	8.441673	1.390098	0.456914
H	8.530243	-0.305633	1.787365
H	8.064114	3.011382	-0.908594
C	1.075614	2.123710	1.054366
H	0.545432	2.843613	0.425400
H	0.323278	1.435327	1.457903
H	1.540641	2.654953	1.884418
C	0.521072	0.260347	-3.587086
H	-0.227572	-0.262392	-2.985229
H	0.292577	1.328788	-3.488528
H	0.381919	-0.020512	-4.635561
N	4.140100	-0.841360	-3.785133
O	5.879855	-0.814706	-2.290123
C	6.726647	-1.218137	-3.370458
H	6.394886	-2.165957	-3.801646
H	6.736633	-0.460748	-4.159680
H	7.718721	-1.322653	-2.928185
C	3.997183	2.126757	1.658404
N	3.877231	1.751034	2.958984
H	3.505429	0.864906	3.274930
H	4.282485	2.365398	3.653423
O	4.530053	3.183503	1.336357
O	5.492618	2.426748	-1.498328
C	5.780857	3.787232	-1.806180
H	4.908831	4.155816	-2.348664

H	5.915019	4.366240	-0.886783
H	6.666370	3.878646	-2.448097
C	9.803507	1.778490	0.683786
N	10.909965	2.088385	0.867406

R-forming TS Conformation 13

B3LYP/6-31G(d) Energy = -4490.496497
M06-2X/6-311G(d,p)-SMD(tetrahydrofuran) Energy = -4490.002793
M06-2X/6-311G(d,p)-SMD(tetrahydrofuran)-derived Free Energy (Quasiharmonic) = -4488.904405

Frequencies (Top 3 out of 468)

1. -1152.5466 cm^{-1}
2. 6.0958 cm^{-1}
3. 9.1849 cm^{-1}

B3LYP/6-31G(d) Molecular Geometry in Cartesian Coordinates

N	1.075194	-1.854438	1.146706
C	1.536729	-2.492891	0.029132
C	2.897752	-2.726688	-0.074376
C	3.176499	-1.834584	2.223269
C	3.787073	-2.172375	0.945753
H	4.205001	-0.988713	0.460788
H	0.085220	-1.531450	1.153320
C	3.561826	-3.500776	-1.144856
O	2.718710	-4.001844	-2.076847
O	4.767858	-3.684213	-1.183307
C	4.115575	-1.715609	3.363426
O	3.528380	-1.487356	4.552113
O	5.321847	-1.857909	3.241194
C	3.344300	-4.724680	-3.149948
H	3.951480	-4.046516	-3.755118
H	3.973584	-5.527791	-2.759136
H	2.522799	-5.130723	-3.741410
C	4.433769	-1.251181	5.642575
H	5.039521	-0.365833	5.435257
H	3.799735	-1.089015	6.514888
H	5.087968	-2.113127	5.795220
C	0.466855	-2.879774	-0.954397
H	0.713106	-2.529865	-1.958084
H	0.375984	-3.967879	-1.009063
H	-0.499345	-2.472865	-0.652519
C	1.029050	-1.067570	3.440661
H	0.966119	-1.829950	4.223693
H	1.528591	-0.200415	3.876176
H	0.018139	-0.800221	3.126125
P	-1.984845	0.150160	0.061532
O	-1.368068	-0.590102	1.220900
O	-1.131560	0.697221	-1.049096
O	-3.147789	-0.784780	-0.669685
O	-2.919817	1.340621	0.738728
H	4.761410	-2.652434	1.003279

C	-3.888217	1.941116	-0.041830
C	-5.051475	1.239032	-0.329834
C	-4.647987	3.875794	-1.248495
C	-3.676112	3.285127	-0.466398
H	-4.513050	4.904197	-1.573968
C	-5.248965	-0.119007	0.253926
C	-4.289012	-1.101452	0.042136
C	-4.462365	-2.447843	0.476896
C	-5.600989	-2.754554	1.193977
H	-5.756979	-3.777837	1.526079
C	-6.761490	3.782160	-2.527444
C	-7.865480	3.085984	-2.960950
C	-8.055897	1.746044	-2.547933
C	-7.159530	1.137605	-1.697341
C	-6.017166	1.833818	-1.212208
C	-5.809273	3.177646	-1.662654
H	-6.591526	4.806911	-2.849480
H	-8.584192	3.555689	-3.626845
H	-8.916216	1.189041	-2.909371
H	-7.316521	0.107772	-1.398227
C	-8.419381	0.200895	2.267390
C	-7.343873	0.544023	1.478314
C	-6.390894	-0.430645	1.069055
C	-6.566358	-1.774590	1.532008
C	-7.695250	-2.099480	2.331991
C	-8.607904	-1.135744	2.691781
H	-9.128065	0.966632	2.571482
H	-7.210868	1.574697	1.170908
H	-7.815722	-3.127936	2.664288
H	-9.464354	-1.393425	3.308762
C	1.819521	-1.586261	2.269931
C	3.820785	-0.010265	-1.506366
C	4.319122	0.312182	-0.159960
C	3.420754	1.147631	0.633299
C	2.109666	1.343626	0.266458
C	2.475593	0.251505	-1.850454
H	0.625431	0.888951	-1.088663
N	1.651341	0.850655	-0.922957
C	1.984755	-0.069315	-3.147963
C	2.881102	-0.694623	-3.993891
C	4.607168	-0.670724	-2.499946
H	2.559931	-0.966595	-4.998290
C	-3.488277	-3.539195	0.156006
C	-3.467410	-4.101746	-1.142821
C	-2.672897	-4.078606	1.179377
C	-4.268779	-3.596165	-2.216396
C	-2.628309	-5.247610	-1.411891
C	-1.850049	-5.235426	0.902324
C	-2.616047	-3.519070	2.497442
C	-4.233403	-4.166563	-3.461785
H	-4.911298	-2.742465	-2.032737
C	-2.623220	-5.814211	-2.725141
C	-1.853729	-5.789713	-0.381202
C	-1.049851	-5.788591	1.951359
H	-3.192190	-2.626590	2.709658

C	-1.830597	-4.076721	3.472414
C	-3.399269	-5.290796	-3.723751
H	-4.848290	-3.759507	-4.259902
H	-1.987263	-6.676902	-2.910645
H	-1.241256	-6.666822	-0.582517
C	-1.042082	-5.232154	3.201843
H	-0.444877	-6.664909	1.729852
H	-1.803703	-3.631062	4.463221
H	-3.386475	-5.731325	-4.717118
H	-0.430884	-5.663633	3.990147
C	-2.468034	4.074192	-0.069271
C	-1.501353	4.418264	-1.044552
C	-2.342485	4.552359	1.257402
C	-1.548566	3.925056	-2.389467
C	-0.406875	5.294567	-0.688089
C	-1.229963	5.406131	1.607276
C	-3.292692	4.244128	2.283127
C	-0.613972	4.301527	-3.319014
H	-2.332990	3.229158	-2.661191
C	0.544040	5.665627	-1.691167
C	-0.300770	5.762084	0.624881
C	-1.114076	5.885762	2.949653
H	-4.141356	3.615383	2.039381
C	-3.145966	4.724071	3.558094
C	0.442576	5.192314	-2.971151
H	-0.676209	3.915701	-4.333105
H	1.350497	6.339425	-1.409956
H	0.522844	6.423046	0.888640
C	-2.040967	5.554502	3.900563
H	-0.269947	6.525737	3.196345
H	-3.879564	4.469972	4.318295
H	1.167802	5.487040	-3.725228
H	-1.941547	5.925651	4.917054
C	5.794592	0.638151	0.023900
C	6.624926	-0.061790	0.897508
C	6.334710	1.748240	-0.671532
C	7.968936	0.276224	1.056412
H	6.223634	-0.880512	1.482906
C	7.678409	2.088231	-0.535645
C	8.501401	1.343203	0.327685
H	8.596160	-0.286617	1.738807
H	8.102492	2.924939	-1.076221
C	1.091650	2.066160	1.101910
H	0.355532	1.351922	1.489228
H	1.556794	2.577615	1.944617
H	0.544010	2.797798	0.501505
C	0.592451	0.278431	-3.607504
H	-0.187305	-0.191184	-3.000844
H	0.408759	1.358575	-3.548298
H	0.458034	-0.030739	-4.648754
N	4.157494	-1.011061	-3.692832
O	5.875050	-0.959891	-2.167099
C	6.653802	-1.731268	-3.090112
H	6.229007	-2.731859	-3.193394
H	6.698702	-1.243372	-4.067213

H	7.645435	-1.787724	-2.639262
C	3.946503	1.772483	1.907669
N	4.283360	3.089423	1.776247
H	4.763588	3.532366	2.549433
H	4.381226	3.493063	0.855208
O	4.046057	1.170533	2.970364
O	5.457933	2.463277	-1.441973
C	5.973510	3.458777	-2.318250
H	5.116879	3.823690	-2.887147
H	6.423695	4.293066	-1.764586
H	6.717190	3.037043	-3.005008
C	9.883147	1.702849	0.462371
N	11.002870	2.001465	0.567428

R-forming TS Conformation 14

B3LYP/6-31G(d) Energy = -4490.490949
M06-2X/6-311G(d,p)-SMD(tetrahydrofuran) Energy = -4490.001924
M06-2X/6-311G(d,p)-SMD(tetrahydrofuran)-derived Free Energy (Quasiharmonic) = -4488.904339

Frequencies (Top 3 out of 468)

1. -1181.2081 cm^{-1}
2. 5.5018 cm^{-1}
3. 6.6386 cm^{-1}

B3LYP/6-31G(d) Molecular Geometry in Cartesian Coordinates

N	-1.212456	0.472768	1.825915
C	-1.955096	1.619044	1.694599
C	-3.327654	1.523936	1.841339
C	-3.064674	-0.873297	2.404669
C	-3.939468	0.202868	1.944538
H	-4.308057	-0.118100	0.687839
H	-0.180606	0.536976	1.649603
C	-4.268680	2.662561	1.952403
O	-3.678854	3.878662	1.889113
O	-5.468111	2.527121	2.128626
C	-3.618873	-2.074216	3.063832
O	-4.970274	-1.982465	3.235373
O	-2.987692	-3.038083	3.455278
C	-4.552351	4.998936	2.095444
H	-3.912987	5.879865	2.032018
H	-5.328925	5.032880	1.327451
H	-5.028623	4.938230	3.077656
C	-5.561721	-3.099188	3.917869
H	-6.626268	-2.870679	3.984078
H	-5.397044	-4.022837	3.356913
H	-5.131109	-3.211179	4.916079
C	-1.142448	2.857215	1.436398
H	-0.112916	2.589047	1.191897
H	-1.571737	3.445138	0.624133
H	-1.126179	3.501017	2.321138
C	-0.643270	-1.734204	2.653720

H	-0.702851	-1.957491	3.723274
H	-0.807978	-2.684202	2.141243
H	0.352897	-1.355063	2.419435
P	2.061872	-0.072857	0.068742
O	1.450103	0.580377	1.283325
O	1.200210	-0.712906	-0.984733
O	3.036228	0.997763	-0.744720
O	3.185724	-1.132148	0.661481
H	-4.938410	0.211846	2.363127
C	4.190100	-1.605256	-0.159442
C	5.224163	-0.751747	-0.523053
C	5.177899	-3.451770	-1.337682
C	4.161049	-2.982441	-0.530303
H	5.185853	-4.501102	-1.620876
C	5.270041	0.635799	0.022401
C	4.177825	1.480265	-0.136354
C	4.210938	2.856874	0.235699
C	5.356978	3.334305	0.839892
H	5.409998	4.383584	1.118589
C	7.185705	-3.095290	-2.734021
C	8.145830	-2.259316	-3.254200
C	8.148710	-0.889706	-2.899126
C	7.215569	-0.388840	-2.018278
C	6.220578	-1.228504	-1.443848
C	6.197729	-2.605311	-1.837306
H	7.157549	-4.146526	-3.011085
H	8.892356	-2.642596	-3.944415
H	8.892229	-0.224046	-3.329430
H	7.228442	0.664821	-1.765011
C	8.588838	0.795485	1.804323
C	7.510657	0.288269	1.113297
C	6.418431	1.120570	0.739574
C	6.459253	2.496056	1.135916
C	7.594456	2.993093	1.832028
C	8.642098	2.164356	2.158583
H	9.405255	0.135288	2.084684
H	7.483777	-0.764755	0.858967
H	7.610528	4.043504	2.113254
H	9.502346	2.552473	2.697033
C	-1.692853	-0.723812	2.281032
C	-3.585377	0.714962	-1.277704
C	-4.300310	-0.458069	-0.728028
C	-3.544604	-1.708582	-0.753854
C	-2.176542	-1.705049	-0.997905
C	-2.219932	0.626884	-1.619123
H	-0.511110	-0.561972	-1.388819
N	-1.550686	-0.551821	-1.360963
C	-1.556734	1.707729	-2.261082
C	-2.313823	2.851058	-2.448043
C	-4.194755	1.988300	-1.486072
H	-1.865256	3.707293	-2.948650
C	3.078410	3.798577	-0.026999
C	2.385421	4.377598	1.064683
C	2.769848	4.191674	-1.352414
C	2.609008	3.980073	2.423805

C	1.397835	5.405950	0.821260
C	1.750721	5.190511	-1.583253
C	3.446947	3.658082	-2.495851
C	1.950197	4.586070	3.461571
H	3.301100	3.170685	2.622530
C	0.742167	6.021110	1.934534
C	1.103860	5.779346	-0.492752
C	1.441859	5.575953	-2.925921
H	4.229231	2.923130	-2.346478
C	3.123919	4.055246	-3.767161
C	1.014444	5.632638	3.218842
H	2.136954	4.261395	4.481613
H	0.019507	6.808913	1.733563
H	0.357774	6.551352	-0.671666
C	2.103686	5.023912	-3.990085
H	0.672495	6.329220	-3.080684
H	3.652166	3.629800	-4.616003
H	0.513372	6.112064	4.055808
H	1.863056	5.328851	-5.005076
C	3.094480	-3.921150	-0.063539
C	2.217994	-4.507429	-1.010229
C	3.016772	-4.289406	1.301898
C	2.215821	-4.146717	-2.397701
C	1.265414	-5.506668	-0.578444
C	2.033687	-5.260814	1.724900
C	3.895688	-3.758429	2.299871
C	1.378168	-4.759490	-3.292944
H	2.883022	-3.362259	-2.734175
C	0.413243	-6.125358	-1.547005
C	1.193244	-5.848311	0.774703
C	1.956417	-5.620929	3.107077
H	4.656140	-3.045083	2.004178
C	3.793575	-4.133637	3.613805
C	0.471376	-5.772780	-2.868371
H	1.397969	-4.463785	-4.338581
H	-0.289255	-6.881798	-1.205415
H	0.469582	-6.595059	1.095604
C	2.807125	-5.073166	4.028542
H	1.204823	-6.347287	3.407439
H	4.472486	-3.710945	4.349542
H	-0.181525	-6.251336	-3.593249
H	2.739074	-5.356053	5.075602
C	-5.750896	-0.570577	-1.211257
C	-5.919410	-0.747959	-2.588752
C	-6.908613	-0.491941	-0.409892
C	-7.176977	-0.809882	-3.183434
H	-5.035467	-0.827936	-3.213648
C	-8.177052	-0.534973	-0.988596
C	-8.313692	-0.688311	-2.377940
H	-7.276397	-0.942918	-4.255019
H	-9.067519	-0.461539	-0.377418
C	-1.280914	-2.909611	-0.933374
H	-0.250894	-2.600188	-0.742455
H	-1.611937	-3.615239	-0.171912
H	-1.307280	-3.447795	-1.885701

C	-0.147722	1.619761	-2.786931
H	-0.033164	0.775874	-3.478587
H	0.106975	2.536304	-3.326179
H	0.597766	1.474931	-2.002372
N	-3.592205	3.014599	-2.054532
O	-5.461439	2.135472	-1.051409
C	-6.116130	3.376742	-1.331085
H	-6.206155	3.532769	-2.410097
H	-7.100830	3.286633	-0.871416
H	-5.564187	4.215860	-0.900689
C	-4.222300	-3.040019	-0.570620
N	-5.237840	-3.095030	0.339109
H	-5.732729	-3.972888	0.410158
H	-5.532679	-2.321220	0.920011
O	-3.880760	-4.032440	-1.207621
O	-6.717556	-0.412517	0.935998
C	-7.810067	-0.047744	1.775147
H	-8.252395	0.899116	1.448319
H	-8.577694	-0.831578	1.796383
H	-7.385862	0.077987	2.771106
C	-9.624944	-0.727522	-2.957005
N	-10.690978	-0.755595	-3.422285

R-forming TS Conformation 15

B3LYP/6-31G(d) Energy = -4490.493192
M06-2X/6-311G(d,p)-SMD(tetrahydrofuran) Energy = -4490.001027
M06-2X/6-311G(d,p)-SMD(tetrahydrofuran)-derived Free Energy (Quasiharmonic) = -4488.902724

Frequencies (Top 3 out of 468)

1. -1198.5025 cm^{-1}
2. 6.7125 cm^{-1}
3. 7.4575 cm^{-1}

B3LYP/6-31G(d) Molecular Geometry in Cartesian Coordinates

N	-1.226179	0.560213	1.828428
C	-1.939839	1.717218	1.642559
C	-3.315147	1.660189	1.779292
C	-3.110301	-0.714304	2.454775
C	-3.955352	0.361214	1.941344
H	-4.333982	-0.025897	0.695581
H	-0.193664	0.592907	1.651077
C	-4.228186	2.826976	1.829423
O	-3.601096	4.023954	1.749514
O	-5.434053	2.727659	1.973218
C	-3.820272	-1.824781	3.124515
O	-3.019122	-2.800176	3.600533
O	-5.036412	-1.863029	3.252826
C	-4.449458	5.173411	1.888431
H	-3.782549	6.034085	1.830163
H	-5.190466	5.208611	1.086029
H	-4.969693	5.152568	2.849708

C	-3.693795	-3.898312	4.236771
H	-4.352063	-4.402342	3.524685
H	-2.901733	-4.569755	4.568823
H	-4.284936	-3.547120	5.086046
C	-1.094368	2.922843	1.340749
H	-1.510707	3.496570	0.512184
H	-1.055000	3.593161	2.205012
H	-0.073865	2.617998	1.101418
C	-0.692538	-1.617865	2.730539
H	-0.713823	-1.772247	3.813562
H	-0.886389	-2.589343	2.271912
H	0.302037	-1.271704	2.443857
P	2.039169	-0.068097	0.064195
O	1.443181	0.570775	1.294522
O	1.163469	-0.678562	-0.994606
O	3.023258	1.003263	-0.735551
O	3.151463	-1.153833	0.633337
H	-4.958702	0.398464	2.350665
C	4.149266	-1.621003	-0.198881
C	5.192334	-0.771958	-0.547132
C	5.114439	-3.453322	-1.418045
C	4.104685	-2.989524	-0.598579
H	5.109625	-4.496400	-1.723553
C	5.253549	0.604996	0.022914
C	4.171374	1.464433	-0.122988
C	4.222344	2.836094	0.265105
C	5.374075	3.290577	0.876325
H	5.441005	4.335683	1.167360
C	7.124306	-3.091022	-2.809660
C	8.093320	-2.255409	-3.313558
C	8.112353	-0.893678	-2.929759
C	7.186183	-0.400652	-2.037249
C	6.182292	-1.240720	-1.478981
C	6.143286	-2.608529	-1.901313
H	7.083607	-4.135794	-3.108724
H	8.834499	-2.632585	-4.012819
H	8.862899	-0.227703	-3.347147
H	7.211603	0.647245	-1.762145
C	8.573829	0.698409	1.806301
C	7.489788	0.214450	1.107778
C	6.407678	1.065264	0.746714
C	6.465072	2.434336	1.162605
C	7.605962	2.907296	1.866145
C	8.643325	2.061162	2.180799
H	9.382373	0.024509	2.076712
H	7.450326	-0.834243	0.837474
H	7.634886	3.953273	2.162433
H	9.508045	2.430940	2.724951
C	-1.734741	-0.606226	2.330960
C	-3.648443	0.734420	-1.291856
C	-4.346215	-0.425073	-0.691155
C	-3.579169	-1.671856	-0.694094
C	-2.214950	-1.661075	-0.951558
C	-2.280819	0.655393	-1.625259
H	-0.560915	-0.515436	-1.377622

N	-1.598891	-0.509082	-1.337560
C	-1.626709	1.730934	-2.285631
C	-2.398551	2.858199	-2.506926
C	-4.274958	1.993303	-1.537384
H	-1.957436	3.708949	-3.023546
C	3.104622	3.796960	0.008874
C	2.800625	4.201438	-1.314389
C	2.422692	4.382021	1.104366
C	3.466364	3.662081	-2.461787
C	1.798178	5.218438	-1.538691
C	1.453196	5.428932	0.867464
C	2.638693	3.971899	2.460980
C	3.146681	4.069857	-3.730542
H	4.237088	2.914009	-2.317960
C	1.492206	5.614461	-2.878852
C	1.163989	5.813790	-0.444263
C	0.808227	6.048502	1.984551
H	3.316475	3.149242	2.654661
C	1.989654	4.582113	3.502429
C	2.141825	5.055840	-3.946966
H	3.665863	3.639378	-4.582435
H	0.734433	6.380358	-3.028428
H	0.431005	6.599307	-0.618375
C	1.072536	5.646571	3.266380
H	0.099431	6.850010	1.788519
H	2.169860	4.247275	4.520373
H	1.903363	5.368527	-4.960096
H	0.579013	6.128721	4.106212
C	3.031656	-3.930219	-0.150108
C	2.962718	-4.340136	1.203827
C	2.144995	-4.483016	-1.107247
C	3.847792	-3.839060	2.211786
C	1.983898	-5.326070	1.602724
C	1.193920	-5.494005	-0.700376
C	2.132186	-4.076254	-2.481766
C	3.757407	-4.256936	3.513685
H	4.603385	-3.113738	1.933349
C	1.921044	-5.733206	2.972552
C	1.135008	-5.881729	0.640938
C	0.331048	-6.077493	-1.681162
H	2.799018	-3.282904	-2.797389
C	1.283915	-4.655717	-3.389006
C	2.778206	-5.214089	3.904724
H	4.440786	-3.856241	4.257484
H	1.177068	-6.475155	3.254122
H	0.415931	-6.641185	0.942217
C	0.377755	-5.679997	-2.990144
H	-0.370637	-6.843201	-1.359137
H	1.294462	-4.324867	-4.424128
H	2.722199	-5.534207	4.941826
H	-0.283852	-6.131345	-3.724449
C	-5.804410	-0.568198	-1.140805
C	-6.007288	-0.725286	-2.515709
C	-6.939211	-0.553371	-0.302062
C	-7.278848	-0.823526	-3.074446

H	-5.140129	-0.761853	-3.167637
C	-8.222313	-0.629726	-0.845351
C	-8.394148	-0.759527	-2.233150
H	-7.405714	-0.941076	-4.144938
H	-9.096426	-0.596548	-0.207999
C	-1.309614	-2.858508	-0.878464
H	-0.282081	-2.540249	-0.688658
H	-1.635578	-3.562386	-0.113068
H	-1.330421	-3.402247	-1.827723
C	-0.208584	1.653458	-2.788539
H	0.525914	1.524403	-1.990451
H	-0.072212	0.804595	-3.470068
H	0.045293	2.567816	-3.332118
N	-3.682726	3.012876	-2.129794
O	-5.544761	2.132049	-1.114219
C	-6.215504	3.359607	-1.414170
H	-5.672177	4.213356	-1.002353
H	-6.312345	3.492937	-2.495717
H	-7.196164	3.266464	-0.947087
C	-4.240159	-3.013755	-0.504156
N	-5.210927	-3.099621	0.446693
H	-5.690940	-3.985371	0.524259
H	-5.486338	-2.345409	1.064163
O	-3.910262	-3.988594	-1.175629
O	-6.705434	-0.497143	1.035000
C	-7.800524	-0.405986	1.942196
H	-7.350054	-0.407103	2.933888
H	-8.362994	0.520867	1.782868
H	-8.469690	-1.269910	1.845839
C	-9.719562	-0.834106	-2.775436
N	-10.796465	-0.891139	-3.212301

R-forming TS Conformation 16

B3LYP/6-31G(d) Energy = -4490.495488
M06-2X/6-311G(d,p)-SMD(tetrahydrofuran) Energy = -4490.003565
M06-2X/6-311G(d,p)-SMD(tetrahydrofuran)-derived Free Energy (Quasiharmonic) = -4488.906152

Frequencies (Top 3 out of 468)

1. -1172.8559 cm^{-1}
2. 4.1492 cm^{-1}
3. 6.7453 cm^{-1}

B3LYP/6-31G(d) Molecular Geometry in Cartesian Coordinates

N	-1.130939	0.441875	1.998798
C	-1.932606	1.550213	2.105182
C	-3.289910	1.359777	2.295543
C	-2.890406	-1.082962	2.373406
C	-3.830299	0.016722	2.195577
H	-4.255114	-0.084977	0.867278
H	-0.120809	0.589033	1.751265
C	-4.188979	2.498651	2.595073

O	-5.439636	2.069357	2.902829
O	-3.879760	3.676077	2.609901
C	-3.502531	-2.381745	2.696762
O	-2.638379	-3.360607	3.019023
O	-4.717434	-2.561130	2.680254
C	-6.366057	3.096831	3.284360
H	-7.294942	2.577377	3.521539
H	-5.995882	3.641061	4.157232
H	-6.519831	3.802581	2.465135
C	-3.225469	-4.644536	3.295965
H	-3.761977	-5.013583	2.418281
H	-2.385827	-5.296673	3.536316
H	-3.917017	-4.576278	4.139044
C	-1.215143	2.867898	2.052327
H	-0.170906	2.725341	1.767490
H	-1.705555	3.548301	1.354672
H	-1.249567	3.358626	3.030380
C	-0.418965	-1.849474	2.328178
H	0.542374	-1.377551	2.124649
H	-0.392214	-2.284373	3.331317
H	-0.567310	-2.679013	1.633026
P	2.002035	0.039152	-0.028912
O	1.431520	0.755505	1.170379
O	1.100290	-0.568247	-1.067663
O	3.055195	1.023740	-0.845907
O	3.046844	-1.085990	0.602597
H	-4.798568	-0.144423	2.656940
C	4.029034	-1.643711	-0.190271
C	5.114054	-0.865887	-0.576202
C	4.914608	-3.585296	-1.298320
C	3.929275	-3.031804	-0.505111
H	4.867738	-4.644339	-1.538468
C	5.252819	0.525113	-0.055964
C	4.218768	1.436860	-0.226803
C	4.334025	2.808603	0.145616
C	5.504167	3.215342	0.754175
H	5.618703	4.259280	1.034600
C	6.930389	-3.386652	-2.715340
C	7.935319	-2.623252	-3.261830
C	8.019293	-1.246226	-2.947268
C	7.119629	-0.665992	-2.080498
C	6.078496	-1.428953	-1.481619
C	5.975450	-2.813548	-1.832297
H	6.841256	-4.442459	-2.960301
H	8.656086	-3.069379	-3.941552
H	8.799182	-0.638270	-3.398003
H	7.193986	0.392088	-1.857744
C	8.572001	0.486183	1.734543
C	7.466192	0.043573	1.042648
C	6.428171	0.939885	0.662128
C	6.552840	2.311767	1.053789
C	7.714479	2.740482	1.751570
C	8.708585	1.850374	2.083988
H	9.345693	-0.221967	2.019117
H	7.376273	-1.006851	0.792322

H	7.794123	3.788944	2.029129
H	9.589743	2.187025	2.623251
C	-1.533272	-0.843663	2.228946
C	-3.669580	1.118825	-0.901714
C	-4.374540	-0.129237	-0.513073
C	-3.671112	-1.357349	-0.899063
C	-2.323513	-1.332255	-1.199181
C	-2.308831	1.073496	-1.279166
H	-0.624147	-0.179612	-1.340369
N	-1.659515	-0.144282	-1.273065
C	-1.626941	2.243097	-1.708004
C	-2.373354	3.407668	-1.708295
C	-4.263421	2.414468	-0.899956
H	-1.915102	4.332535	-2.054515
C	3.257354	3.814557	-0.115453
C	2.554796	4.384359	0.974053
C	3.013716	4.267549	-1.434270
C	2.730591	3.942537	2.326281
C	1.610782	5.453607	0.735019
C	2.049738	5.320420	-1.661681
C	3.701911	3.738890	-2.573246
C	2.061525	4.539325	3.362932
H	3.395486	3.109534	2.520093
C	0.934640	6.049401	1.846263
C	1.382204	5.889213	-0.573024
C	1.810698	5.770484	-2.998547
H	4.439290	2.958434	-2.424120
C	3.443393	4.196246	-3.838877
C	1.158369	5.615212	3.125156
H	2.213732	4.183477	4.378464
H	0.236914	6.860032	1.649425
H	0.672200	6.695498	-0.747421
C	2.482440	5.224167	-4.059166
H	1.083444	6.564768	-3.151679
H	3.976791	3.772686	-4.685489
H	0.642424	6.080907	3.960661
H	2.292176	5.575600	-5.069734
C	2.831358	-3.901080	0.020592
C	1.916727	-4.497752	-0.882096
C	2.764397	-4.202766	1.403504
C	1.907964	-4.206338	-2.285767
C	0.934796	-5.438412	-0.388525
C	1.756802	-5.119410	1.887392
C	3.678211	-3.657139	2.361706
C	1.033661	-4.828792	-3.138275
H	2.600392	-3.466471	-2.669016
C	0.043966	-6.068799	-1.313824
C	0.877168	-5.716939	0.979575
C	1.695786	-5.415953	3.285508
H	4.456126	-2.984120	2.021004
C	3.588193	-3.968119	3.693278
C	0.094579	-5.782859	-2.651371
H	1.048880	-4.586416	-4.197446
H	-0.677274	-6.785165	-0.926888
H	0.137716	-6.427110	1.345592

C	2.580567	-4.855808	4.166929
H	0.931950	-6.109380	3.631062
H	4.294013	-3.535607	4.397079
H	-0.587153	-6.269826	-3.343253
H	2.525483	-5.092091	5.226274
C	-5.880394	-0.185274	-0.739619
C	-6.815929	-0.216139	0.292929
C	-6.343630	-0.281558	-2.074474
C	-8.179023	-0.357510	0.044478
H	-6.478553	-0.125936	1.317854
C	-7.705545	-0.438306	-2.338902
C	-8.623976	-0.475935	-1.277680
H	-8.890469	-0.386151	0.862556
H	-8.066000	-0.526428	-3.356145
C	-1.503655	-2.548524	-1.527631
H	-1.593076	-2.773205	-2.595580
H	-0.449340	-2.372288	-1.302293
H	-1.854601	-3.428274	-0.986076
C	-0.194050	2.247003	-2.172106
H	0.509847	2.205028	-1.335584
H	0.036326	1.398560	-2.823893
H	0.016335	3.165997	-2.726254
N	-3.651963	3.511794	-1.302471
O	-5.530833	2.516192	-0.445975
C	-6.136416	3.814241	-0.499178
H	-5.590775	4.520633	0.132092
H	-6.154266	4.195009	-1.523876
H	-7.152612	3.664465	-0.130285
C	-4.419443	-2.645423	-1.174521
N	-5.130105	-3.193373	-0.155844
H	-5.193534	-2.794150	0.774419
H	-5.706141	-3.994250	-0.377657
O	-4.369296	-3.163801	-2.288266
O	-5.397383	-0.203863	-3.044274
C	-5.701996	-0.702901	-4.343277
H	-6.056583	-1.737579	-4.285896
H	-6.441936	-0.073890	-4.854054
H	-4.762276	-0.672190	-4.895910
C	-10.021832	-0.633782	-1.557151
N	-11.156549	-0.759954	-1.781699

R-forming TS Conformation 17

B3LYP/6-31G(d) Energy = -4490.493618
M06-2X/6-311G(d,p)-SMD(tetrahydrofuran) Energy = -4490.002589
M06-2X/6-311G(d,p)-SMD(tetrahydrofuran)-derived Free Energy (Quasiharmonic) = -4488.904289

Frequencies (Top 3 out of 468)

1. -1186.4804 cm^{-1}
2. 6.0606 cm^{-1}
3. 6.1571 cm^{-1}

B3LYP/6-31G(d) Molecular Geometry in Cartesian Coordinates

N	-1.219664	0.507966	1.825699
C	-1.693267	-0.699633	2.258732
C	-3.065538	-0.857475	2.371958
C	-3.341989	1.547246	1.850395
C	-3.946625	0.224991	1.933261
H	-4.308968	-0.083954	0.666307
H	-0.187850	0.580980	1.652699
C	-3.742969	-2.032927	2.952910
O	-2.917411	-2.999671	3.402567
O	-4.961703	-2.132700	3.033850
C	-4.184515	2.761124	1.953583
O	-5.431720	2.462547	2.399589
O	-3.831370	3.904485	1.725039
C	-3.563015	-4.159276	3.954719
H	-2.752798	-4.817089	4.269604
H	-4.189214	-3.882038	4.806251
H	-4.182230	-4.646723	3.197456
C	-6.284489	3.591269	2.633053
H	-7.213316	3.177755	3.027981
H	-5.830516	4.273458	3.356925
H	-6.473096	4.136543	1.705318
C	-0.624207	-1.703230	2.602848
H	0.361528	-1.309447	2.350635
H	-0.651514	-1.928318	3.673136
H	-0.783032	-2.648595	2.080091
C	-1.186429	2.908340	1.474992
H	-0.133724	2.676371	1.303299
H	-1.589156	3.467098	0.628735
H	-1.261419	3.572548	2.341664
P	2.056842	-0.038516	0.072525
O	1.447730	0.592101	1.300303
O	1.193805	-0.668200	-0.986053
O	3.025827	1.045812	-0.727090
O	3.185379	-1.105670	0.646132
H	-4.945623	0.189407	2.350084
C	4.187993	-1.561850	-0.186212
C	5.218449	-0.699744	-0.539955
C	5.175743	-3.385542	-1.400593
C	4.161396	-2.932321	-0.580846
H	5.184938	-4.429802	-1.701965
C	5.262816	0.679073	0.027170
C	4.168209	1.523257	-0.115341
C	4.199464	2.894630	0.275835
C	5.346803	3.365265	0.882826
H	5.398439	4.410523	1.176417
C	7.177007	-3.000355	-2.798571
C	8.132719	-2.153010	-3.308250
C	8.133366	-0.789607	-2.929876
C	7.202611	-0.306298	-2.036845
C	6.212224	-1.158358	-1.472720
C	6.191477	-2.528251	-1.889624
H	7.150446	-4.046771	-3.093484
H	8.877397	-2.522361	-4.007979
H	8.873245	-0.114684	-3.351898

H	7.213628	0.742900	-1.765769
C	8.587750	0.819916	1.799351
C	7.508300	0.320429	1.104640
C	6.412777	1.155549	0.747247
C	6.451766	2.525171	1.163445
C	7.588192	3.014458	1.863024
C	8.638912	2.183485	2.173754
H	9.406847	0.157676	2.066850
H	7.483160	-0.728767	0.834597
H	7.602803	4.060639	2.159625
H	9.500101	2.565706	2.714917
C	-1.971028	1.649653	1.704398
C	-3.578808	0.792842	-1.264376
C	-4.305345	-0.389952	-0.745667
C	-3.561079	-1.647435	-0.822276
C	-2.192023	-1.644935	-1.062485
C	-2.216334	0.701440	-1.616732
H	-0.515935	-0.502047	-1.408547
N	-1.555048	-0.487426	-1.385722
C	-1.546145	1.787281	-2.241470
C	-2.294565	2.939919	-2.403298
C	-4.171453	2.080010	-1.429486
H	-1.844717	3.800387	-2.895280
C	3.063320	3.836777	0.031218
C	2.749748	4.248714	-1.287144
C	2.370697	4.395292	1.133728
C	3.428538	3.738743	-2.440349
C	1.722930	5.243618	-1.499999
C	1.373794	5.418720	0.908289
C	2.602470	3.981157	2.486543
C	3.100766	4.154658	-3.704472
H	4.216704	3.007347	-2.304370
C	1.408821	5.648612	-2.835710
C	1.073192	5.809528	-0.399168
C	0.713618	6.010537	2.031456
H	3.303192	3.176110	2.672431
C	1.940879	4.566657	3.534290
C	2.073053	5.119334	-3.909948
H	3.631008	3.747350	-4.560995
H	0.633448	6.398396	-2.976541
H	0.318238	6.575817	-0.563931
C	0.993192	5.606229	3.309154
H	-0.021492	6.789604	1.843656
H	2.134141	4.229769	4.549242
H	1.828634	5.439331	-4.919394
H	0.486787	6.066344	4.153567
C	3.101527	-3.884869	-0.126318
C	2.222639	-4.456317	-1.079795
C	3.037265	-4.286084	1.230508
C	2.206533	-4.061294	-2.457747
C	1.284253	-5.476319	-0.665877
C	2.070744	-5.281326	1.636331
C	3.916129	-3.767223	2.234929
C	1.368116	-4.660069	-3.361659
H	2.863150	-3.261714	-2.778926

C	0.431031	-6.079437	-1.643246
C	1.229215	-5.854479	0.678337
C	2.013106	-5.679807	3.008968
H	4.662672	-3.034604	1.951393
C	3.831072	-4.177209	3.539669
C	0.475097	-5.692991	-2.955608
H	1.376405	-4.338171	-4.399608
H	-0.259865	-6.852939	-1.316251
H	0.520400	-6.621554	0.984812
C	2.863844	-5.143732	3.937394
H	1.278825	-6.429447	3.295717
H	4.509492	-3.762994	4.280559
H	-0.178692	-6.159760	-3.687269
H	2.812030	-5.457539	4.976645
C	-5.761651	-0.471468	-1.216332
C	-5.952399	-0.513298	-2.601583
C	-6.905606	-0.521660	-0.391074
C	-7.217698	-0.568433	-3.179900
H	-5.078955	-0.498686	-3.245764
C	-8.182526	-0.561778	-0.952585
C	-8.341203	-0.580361	-2.347758
H	-7.332978	-0.598682	-4.257683
H	-9.063652	-0.583968	-0.324527
C	-1.304976	-2.858227	-1.037597
H	-1.314172	-3.350062	-2.014718
H	-0.277190	-2.564762	-0.811209
H	-1.654038	-3.596645	-0.316095
C	-0.141183	1.694782	-2.777271
H	0.117788	2.616681	-3.305121
H	0.607814	1.534106	-1.999057
H	-0.037921	0.859706	-3.481402
N	-3.564991	3.107767	-1.988975
O	-5.426987	2.242467	-0.962881
C	-6.040764	3.519480	-1.187948
H	-5.484518	4.307625	-0.674592
H	-6.081873	3.748005	-2.256214
H	-7.048351	3.421773	-0.780456
C	-4.243193	-2.989892	-0.739700
N	-5.237205	-3.128604	0.180594
H	-5.431645	-2.460877	0.917268
H	-5.682626	-4.034680	0.225067
O	-3.919099	-3.914191	-1.481782
O	-6.691614	-0.552633	0.951810
C	-7.799732	-0.668178	1.837662
H	-8.443957	0.218003	1.784192
H	-8.392293	-1.565005	1.618604
H	-7.363672	-0.757097	2.832266
C	-9.661589	-0.617668	-2.905800
N	-10.735217	-0.644049	-3.353470

R-forming TS Conformation 18

B3LYP/6-31G(d) Energy = -4490.492892
M06-2X/6-311G(d,p)-SMD(tetrahydrofuran) Energy = -4489.998911

M06-2X/6-311G(d,p)-SMD(tetrahydrofuran)-derived Free Energy (Quasiharmonic) = -4488.901389

Frequencies (Top 3 out of 468)

1. -1193.3047 cm^{-1}
2. 6.7771 cm^{-1}
3. 8.0535 cm^{-1}

B3LYP/6-31G(d) Molecular Geometry in Cartesian Coordinates

N	-1.112647	0.882253	1.907641
C	-1.754927	-0.125188	2.568374
C	-3.132039	-0.045667	2.700400
C	-3.072263	2.162331	1.580434
C	-3.850856	1.008514	2.005605
H	-4.268249	0.381207	0.821061
H	-0.078327	0.776648	1.753341
C	-3.871351	-1.041491	3.497189
O	-5.118407	-0.622583	3.805426
O	-3.447975	-2.137855	3.842593
C	-3.859712	3.382466	1.282259
O	-3.109005	4.462862	0.977351
O	-5.077574	3.411322	1.328403
C	-5.925186	-1.558925	4.542798
H	-6.097358	-2.459984	3.948651
H	-5.434027	-1.831855	5.479459
H	-6.864339	-1.040642	4.735627
C	-3.837671	5.655578	0.645094
H	-4.324673	5.537030	-0.326629
H	-4.588897	5.874761	1.406880
H	-3.089792	6.447806	0.597586
C	-0.857503	-1.195840	3.128440
H	-1.048514	-2.157952	2.644785
H	0.190867	-0.929173	2.979916
H	-1.055238	-1.346322	4.192862
C	-0.719376	3.091976	1.021324
H	0.277017	2.658164	0.923189
H	-1.030896	3.521453	0.069043
H	-0.664417	3.915753	1.739792
P	2.036178	-0.122784	0.170043
O	1.533904	0.559523	1.420280
O	1.079316	-0.657007	-0.858249
O	3.075844	0.867117	-0.663354
O	3.073058	-1.296338	0.707033
H	-4.843366	1.253021	2.370896
C	4.017991	-1.833396	-0.143844
C	5.113021	-1.062419	-0.513102
C	4.825049	-3.730023	-1.380435
C	3.869312	-3.195993	-0.539147
H	4.740266	-4.770528	-1.682966
C	5.285917	0.307142	0.051479
C	4.270195	1.245416	-0.083511
C	4.437861	2.614678	0.279308
C	5.629657	2.983623	0.870863
H	5.784713	4.024324	1.143539

C	6.825785	-3.512124	-2.814485
C	7.842976	-2.748517	-3.337298
C	7.969179	-1.392680	-2.952946
C	7.099500	-0.835005	-2.041828
C	6.047941	-1.600496	-1.464250
C	5.901216	-2.960994	-1.886252
H	6.703084	-4.550656	-3.112873
H	8.540606	-3.177373	-4.051409
H	8.758126	-0.782544	-3.384617
H	7.207506	0.207576	-1.766693
C	8.631791	0.157869	1.781575
C	7.503384	-0.245950	1.102592
C	6.483959	0.682937	0.752193
C	6.652910	2.046639	1.155172
C	7.836939	2.434983	1.838795
C	8.810728	1.513534	2.145256
H	9.390625	-0.574166	2.044806
H	7.379573	-1.290104	0.840042
H	7.950905	3.477659	2.126049
H	9.709174	1.819302	2.674272
C	-1.695703	2.051850	1.492963
C	-3.768940	0.821743	-1.255360
C	-4.405696	-0.219508	-0.421139
C	-3.632069	-1.465701	-0.313540
C	-2.292603	-1.489006	-0.628989
C	-2.395540	0.731224	-1.566291
H	-0.656406	-0.396955	-1.224277
N	-1.689047	-0.375827	-1.146185
C	-1.752886	1.746117	-2.328987
C	-2.545478	2.819940	-2.690933
C	-4.436946	1.998688	-1.708443
H	-2.107704	3.629776	-3.272631
C	3.404439	3.660815	0.004458
C	2.777295	4.326626	1.086094
C	3.131420	4.057498	-1.328184
C	2.954733	3.925208	2.450824
C	1.901052	5.447423	0.825483
C	2.220792	5.152422	-1.575480
C	3.740780	3.432900	-2.463576
C	2.352713	4.604424	3.477684
H	3.560981	3.052475	2.662152
C	1.305668	6.138564	1.928096
C	1.645006	5.826869	-0.494573
C	1.943247	5.539695	-2.924172
H	4.443829	2.623983	-2.303688
C	3.450041	3.834245	-3.741229
C	1.527679	5.736592	3.217969
H	2.499165	4.273327	4.502225
H	0.667421	6.993217	1.714791
H	0.982389	6.668745	-0.686619
C	2.534327	4.898623	-3.979613
H	1.253567	6.364108	-3.090845
H	3.923947	3.337684	-4.583509
H	1.070048	6.271049	4.046308
H	2.317288	5.204180	-4.999695

C	2.750441	-4.065145	-0.059002
C	1.806149	-4.564244	-0.990021
C	2.695530	-4.471680	1.296798
C	1.775228	-4.151579	-2.362468
C	0.813053	-5.522734	-0.557196
C	1.674383	-5.401667	1.723081
C	3.634611	-4.021892	2.279685
C	0.869539	-4.678876	-3.245947
H	2.475907	-3.395217	-2.695364
C	-0.107490	-6.055484	-1.514387
C	0.770666	-5.909464	0.785020
C	1.625003	-5.804865	3.094506
H	4.421803	-3.339830	1.980548
C	3.555526	-4.434533	3.583947
C	-0.078221	-5.655207	-2.823192
H	0.867908	-4.344740	-4.280042
H	-0.836441	-6.787622	-1.174529
H	0.020823	-6.629886	1.106594
C	2.534627	-5.334696	4.002483
H	0.847050	-6.501878	3.397705
H	4.279837	-4.072936	4.308775
H	-0.782240	-6.069441	-3.539918
H	2.487278	-5.649944	5.041395
C	-5.906599	-0.443095	-0.522941
C	-6.751133	-0.383357	0.582380
C	-6.448952	-0.843515	-1.768061
C	-8.109208	-0.686984	0.487085
H	-6.347457	-0.104748	1.548054
C	-7.802267	-1.157087	-1.877834
C	-8.635152	-1.074155	-0.748813
H	-8.751872	-0.628657	1.358685
H	-8.224257	-1.469901	-2.824517
C	-1.413681	-2.704727	-0.536487
H	-0.391718	-2.418593	-0.275861
H	-1.793532	-3.423879	0.189190
H	-1.364853	-3.206135	-1.510598
C	-0.313468	1.654139	-2.766300
H	0.387171	1.568580	-1.931036
H	-0.141887	0.773957	-3.398722
H	-0.037946	2.541646	-3.343417
N	-3.851594	2.966986	-2.389073
O	-5.740775	2.106654	-1.404143
C	-6.437709	3.281338	-1.827671
H	-6.059986	4.161245	-1.301335
H	-6.336334	3.429497	-2.905899
H	-7.479087	3.102649	-1.556792
C	-4.340414	-2.791910	-0.082537
N	-4.255055	-3.339078	1.160430
H	-3.723693	-2.945506	1.927005
H	-4.680467	-4.247581	1.292486
O	-4.950138	-3.341465	-0.992802
O	-5.583342	-0.889627	-2.811150
C	-5.934861	-1.666721	-3.950297
H	-5.043250	-1.687264	-4.579137
H	-6.199876	-2.686892	-3.652441

H	-6.758846	-1.210594	-4.513949
C	-10.027824	-1.393579	-0.872270
N	-11.158525	-1.650261	-0.971026

R-forming TS Conformation 19*

B3LYP/6-31G(d) Energy = -4490.495243
M06-2X/6-311G(d,p)-SMD(tetrahydrofuran) Energy = -4490.004143
M06-2X/6-311G(d,p)-SMD(tetrahydrofuran)-derived Free Energy (Quasiharmonic) = -4488.907286

Frequencies (Top 3 out of 468)

1. -1089.0321 cm^{-1}
2. 6.5361 cm^{-1}
3. 7.8595 cm^{-1}

B3LYP/6-31G(d) Molecular Geometry in Cartesian Coordinates

N	-1.197203	0.639711	1.949318
C	-1.905000	1.792586	1.740288
C	-3.279873	1.754094	1.895076
C	-3.088743	-0.608082	2.622087
C	-3.930684	0.461464	2.089910
H	-4.263500	0.053064	0.863680
H	-0.168314	0.659947	1.769173
C	-4.080286	2.997362	1.894034
O	-5.359252	2.761702	2.289093
O	-3.679775	4.115925	1.622553
C	-3.703891	-1.772413	3.303220
O	-5.019318	-1.564014	3.550186
O	-3.119699	-2.783555	3.646610
C	-6.181920	3.926191	2.435841
H	-7.150975	3.557135	2.773777
H	-5.752696	4.608772	3.174626
H	-6.283936	4.455385	1.485624
C	-5.718960	-2.694564	4.092008
H	-6.737830	-2.348064	4.270420
H	-5.709749	-3.511652	3.367568
H	-5.255804	-3.023161	5.025739
C	-1.077272	3.000782	1.411271
H	-1.104888	3.720441	2.235550
H	-0.038777	2.714411	1.234553
H	-1.476417	3.521254	0.539572
C	-0.701700	-1.532369	2.919724
H	-0.772192	-1.694094	3.999845
H	-0.896975	-2.503629	2.461325
H	0.307400	-1.198247	2.670293
P	2.055002	-0.068007	0.137499
O	1.481617	0.597756	1.361033
O	1.164254	-0.701497	-0.897591
O	3.031860	0.977307	-0.705524
O	3.172095	-1.152089	0.703768
H	-4.924706	0.514588	2.520040
C	4.135547	-1.650492	-0.149137

C	5.179646	-0.824631	-0.546606
C	5.033243	-3.520420	-1.364036
C	4.057356	-3.025409	-0.521925
H	5.004196	-4.569135	-1.648321
C	5.277717	0.561846	-0.005298
C	4.202768	1.433217	-0.134098
C	4.282484	2.810241	0.229320
C	5.457449	3.259363	0.798405
H	5.545516	4.307893	1.070953
C	7.003954	-3.215579	-2.823852
C	7.969306	-2.404743	-3.373251
C	8.020911	-1.036578	-3.016150
C	7.130704	-0.512651	-2.104850
C	6.132222	-1.326383	-1.499833
C	6.059144	-2.700975	-1.894978
H	6.938529	-4.265104	-3.101260
H	8.682802	-2.806471	-4.087502
H	8.768210	-0.390381	-3.468947
H	7.180529	0.539510	-1.850157
C	8.652206	0.641834	1.674363
C	7.541426	0.160405	1.017286
C	6.459264	1.018871	0.674907
C	6.546060	2.393807	1.065830
C	7.713483	2.863399	1.726725
C	8.749641	2.009451	2.023909
H	9.460241	-0.037868	1.931525
H	7.480886	-0.892120	0.766331
H	7.763980	3.913521	2.004873
H	9.634811	2.377018	2.535727
C	-1.718438	-0.512665	2.483591
C	-3.616267	0.791024	-1.170141
C	-4.303794	-0.346817	-0.532628
C	-3.542575	-1.593443	-0.529393
C	-2.184968	-1.600830	-0.788948
C	-2.253197	0.692051	-1.527341
H	-0.528077	-0.475677	-1.258215
N	-1.568663	-0.466357	-1.223885
C	-1.598240	1.745321	-2.220768
C	-2.366389	2.867545	-2.472692
C	-4.235199	2.045020	-1.452156
H	-1.927274	3.700612	-3.018884
C	3.167283	3.778802	-0.008456
C	2.513028	4.375573	1.097233
C	2.836584	4.174113	-1.327764
C	2.760558	3.978290	2.452209
C	1.539388	5.421395	0.873023
C	1.832400	5.192264	-1.538259
C	3.475836	3.623673	-2.484988
C	2.134643	4.598269	3.501937
H	3.442982	3.157769	2.638591
C	0.917349	6.049711	1.998108
C	1.221955	5.796221	-0.435119
C	1.499796	5.579633	-2.874628
H	4.246989	2.873898	-2.351435
C	3.131470	4.023809	-3.749796

C	1.210512	5.658867	3.276919
H	2.337575	4.272598	4.518610
H	0.199136	6.844503	1.810919
H	0.483759	6.578890	-0.598717
C	2.125625	5.011907	-3.952227
H	0.741133	6.346662	-3.013442
H	3.631928	3.585952	-4.609236
H	0.732103	6.145430	4.122783
H	1.867712	5.319121	-4.962347
C	2.987275	-3.941258	-0.018451
C	2.069500	-4.513021	-0.934498
C	2.950571	-4.312295	1.348251
C	2.020385	-4.143803	-2.318848
C	1.124004	-5.506097	-0.473870
C	1.971465	-5.273792	1.802266
C	3.868732	-3.793685	2.316796
C	1.149371	-4.746817	-3.189013
H	2.680547	-3.361096	-2.672790
C	0.238282	-6.117723	-1.416979
C	1.094458	-5.851780	0.879727
C	1.936118	-5.636299	3.185388
H	4.626142	-3.087673	1.997402
C	3.805825	-4.170543	3.632614
C	0.254550	-5.760446	-2.738707
H	1.137293	-4.447179	-4.233685
H	-0.448602	-6.879931	-1.055635
H	0.377171	-6.594563	1.223481
C	2.823205	-5.099659	4.078463
H	1.186467	-6.354120	3.509877
H	4.512919	-3.756503	4.346245
H	-0.415733	-6.241570	-3.446673
H	2.786362	-5.382977	5.126868
C	-5.797273	-0.507190	-0.777262
C	-6.726940	-0.606287	0.256479
C	-6.261342	-0.628263	-2.110603
C	-8.087484	-0.774837	0.008771
H	-6.380279	-0.574286	1.280822
C	-7.620971	-0.792032	-2.375719
C	-8.537175	-0.858524	-1.312597
H	-8.792559	-0.846676	0.829517
H	-7.986936	-0.873879	-3.391112
C	-1.280482	-2.795265	-0.644813
H	-1.681517	-3.522843	0.060355
H	-1.134602	-3.299134	-1.607986
H	-0.289696	-2.470016	-0.318175
C	-0.179982	1.649022	-2.719881
H	0.554629	1.570799	-1.914924
H	-0.035331	0.765764	-3.354116
H	0.069057	2.535260	-3.310182
N	-3.646403	3.036697	-2.089571
O	-5.503414	2.213596	-1.023786
C	-6.130889	3.464525	-1.333234
H	-5.572516	4.294957	-0.893672
H	-6.190774	3.613690	-2.415050
H	-7.130306	3.391928	-0.901587

C	-4.255962	-2.853603	-0.121127
N	-4.157149	-3.895933	-0.995266
H	-3.726923	-3.798380	-1.901838
H	-4.637158	-4.756393	-0.769104
O	-4.904080	-2.939212	0.916937
O	-5.311309	-0.581798	-3.088092
C	-5.723397	-0.632557	-4.447306
H	-4.808761	-0.548989	-5.036417
H	-6.223002	-1.581403	-4.681897
H	-6.392685	0.200709	-4.695082
C	-9.933139	-1.023210	-1.596390
N	-11.065435	-1.154781	-1.830083

R-forming TS Conformation 20

B3LYP/6-31G(d) Energy = -4490.490983
M06-2X/6-311G(d,p)-SMD(tetrahydrofuran) Energy = -4490.001553
M06-2X/6-311G(d,p)-SMD(tetrahydrofuran)-derived Free Energy (Quasiharmonic) = -4488.904152

Frequencies (Top 3 out of 468)

1. -980.8391 cm^{-1}
2. 6.6173 cm^{-1}
3. 8.4005 cm^{-1}

B3LYP/6-31G(d) Molecular Geometry in Cartesian Coordinates

N	-1.190731	0.622902	1.845879
C	-1.924843	1.763923	1.655512
C	-3.300641	1.685854	1.769238
C	-3.070914	-0.709816	2.368133
C	-3.936447	0.370634	1.883871
H	-4.290764	0.041467	0.683680
H	-0.157729	0.675685	1.709084
C	-4.121555	2.915516	1.790901
O	-5.373271	2.672947	2.263168
O	-3.756001	4.035245	1.475541
C	-3.667516	-1.945678	2.931744
O	-4.937102	-1.743407	3.348402
O	-3.098593	-3.018376	3.040294
C	-6.208279	3.829607	2.395779
H	-7.142343	3.467798	2.827757
H	-5.743429	4.568782	3.053866
H	-6.392506	4.291939	1.422766
C	-5.635062	-2.927479	3.762236
H	-6.611366	-2.584289	4.107689
H	-5.742851	-3.602993	2.910922
H	-5.098225	-3.430969	4.570218
C	-1.117036	3.004012	1.397354
H	-1.228630	3.716328	2.220512
H	-0.059213	2.755350	1.291595
H	-1.469485	3.517554	0.501345
C	-0.674035	-1.571152	2.754601
H	-0.853499	-1.845965	3.798468

H	-0.746807	-2.499339	2.185531
H	0.332670	-1.160637	2.656151
P	2.090182	-0.052587	0.129899
O	1.512052	0.622313	1.345474
O	1.203051	-0.684029	-0.910608
O	3.082403	0.979465	-0.710455
O	3.192974	-1.146305	0.706127
H	-4.930047	0.375901	2.314486
C	4.156915	-1.654275	-0.140772
C	5.211910	-0.838728	-0.530964
C	5.042245	-3.532189	-1.352552
C	4.066283	-3.027866	-0.516020
H	5.004083	-4.580176	-1.638486
C	5.318061	0.547329	0.010432
C	4.251155	1.426918	-0.127605
C	4.335592	2.802233	0.240211
C	5.507785	3.242624	0.821364
H	5.599669	4.289728	1.098181
C	7.026531	-3.246905	-2.797752
C	8.004615	-2.445957	-3.339051
C	8.068617	-1.078888	-2.979752
C	7.177563	-0.546323	-2.074323
C	6.165928	-1.349865	-1.477629
C	6.080723	-2.723158	-1.875026
H	6.951698	-4.295283	-3.077081
H	8.718784	-2.854534	-4.048708
H	8.826220	-0.440392	-3.426280
H	7.236975	0.504909	-1.817876
C	8.678826	0.602805	1.718190
C	7.570595	0.129701	1.050837
C	6.496769	0.995630	0.701166
C	6.588720	2.369345	1.095706
C	7.753411	2.830052	1.767551
C	8.781713	1.969017	2.071575
H	9.480542	-0.082399	1.980477
H	7.505650	-0.921871	0.796936
H	7.808179	3.879233	2.048408
H	9.664809	2.330009	2.591580
C	-1.700823	-0.566909	2.301236
C	-3.552937	0.821838	-1.347491
C	-4.276847	-0.326039	-0.784486
C	-3.521511	-1.571727	-0.729837
C	-2.146638	-1.573268	-0.907922
C	-2.175523	0.723112	-1.647246
H	-0.470049	-0.453930	-1.316476
N	-1.512063	-0.442573	-1.324527
C	-1.491508	1.780703	-2.303126
C	-2.245847	2.911653	-2.563879
C	-4.152606	2.089501	-1.616484
H	-1.784193	3.748149	-3.085550
C	3.226057	3.775592	-0.004973
C	2.553104	4.360270	1.095654
C	2.916827	4.183536	-1.325447
C	2.780185	3.950577	2.450460
C	1.581162	5.406436	0.865792

C	1.915137	5.202773	-1.542477
C	3.574908	3.643981	-2.477233
C	2.136134	4.559150	3.495874
H	3.461628	3.129920	2.639727
C	0.939538	6.022294	1.986754
C	1.285141	5.794272	-0.443606
C	1.604445	5.602945	-2.880373
H	4.343999	2.893014	-2.337957
C	3.250970	4.055956	-3.743650
C	1.213077	5.619629	3.266197
H	2.323756	4.224402	4.512540
H	0.223205	6.817907	1.795737
H	0.548442	6.577438	-0.611582
C	2.248221	5.045827	-3.952976
H	0.847742	6.370954	-3.024395
H	3.765264	3.626306	-4.599045
H	0.720192	6.097010	4.109002
H	2.006659	5.362533	-4.964179
C	2.984141	-3.933400	-0.019133
C	2.065306	-4.496355	-0.939738
C	2.936660	-4.304457	1.347367
C	2.026387	-4.126734	-2.324422
C	1.107539	-5.480020	-0.483880
C	1.945566	-5.255968	1.796421
C	3.855373	-3.795572	2.320533
C	1.153503	-4.720626	-3.199107
H	2.696323	-3.350841	-2.675050
C	0.220642	-6.083281	-1.431634
C	1.067297	-5.825157	0.869533
C	1.899811	-5.618612	3.179198
H	4.621520	-3.097307	2.005092
C	3.782396	-4.172367	3.635846
C	0.246533	-5.725865	-2.753532
H	1.149479	-4.420868	-4.243731
H	-0.472806	-6.841513	-1.074119
H	0.340834	-6.560532	1.209830
C	2.788246	-5.091564	4.076622
H	1.141386	-6.328980	3.499622
H	4.490431	-3.766028	4.352938
H	-0.422842	-6.202139	-3.465696
H	2.743897	-5.375221	5.124653
C	-5.724401	-0.488476	-1.243802
C	-6.885630	-0.338632	-0.453420
C	-5.883102	-0.853200	-2.584765
C	-8.145590	-0.566891	-1.012041
C	-7.132887	-1.075209	-3.158763
C	-8.272000	-0.933809	-2.361668
H	-9.041352	-0.462782	-0.413809
H	-7.223090	-1.356973	-4.202038
C	-1.236492	-2.753375	-0.693773
H	-1.633637	-3.434941	0.058240
H	-1.083297	-3.313015	-1.625460
H	-0.246923	-2.403937	-0.390368
C	-0.057766	1.684814	-2.755671
H	0.650117	1.616080	-1.926434

H	0.110126	0.797508	-3.377943
H	0.207176	2.567408	-3.344388
N	-3.534837	3.087237	-2.216009
O	-5.425102	2.261219	-1.210994
C	-6.033127	3.527636	-1.500057
H	-5.488518	4.334795	-1.003735
H	-6.048251	3.715723	-2.576918
H	-7.049880	3.445185	-1.112735
C	-4.287753	-2.800402	-0.327791
N	-3.988726	-3.964208	-0.972219
H	-3.253555	-4.044897	-1.656678
H	-4.453364	-4.804525	-0.657057
O	-5.184055	-2.746962	0.511202
C	-9.576765	-1.159006	-2.912443
N	-10.636487	-1.339635	-3.357801
O	-6.714631	0.041649	0.832952
C	-7.831983	0.055367	1.707802
H	-8.309718	-0.930681	1.757563
H	-7.433078	0.317856	2.688284
H	-8.573286	0.806165	1.404024
H	-4.994527	-0.969575	-3.198098

S-forming TS Conformation 1

B3LYP/6-31G(d) Energy = -4490.489098
M06-2X/6-311G(d,p)-SMD(tetrahydrofuran) Energy = -4489.997758
M06-2X/6-311G(d,p)-SMD(tetrahydrofuran)-derived Free Energy (Quasiharmonic) = -4488.899551

Frequencies (Top 3 out of 468)

1. -1207.3648 cm^{-1}
2. 7.2300 cm^{-1}
3. 10.0079 cm^{-1}

B3LYP/6-31G(d) Molecular Geometry in Cartesian Coordinates

N	1.138270	0.041890	2.078190
C	1.912890	-1.030010	2.426010
C	3.273830	-0.826921	2.582770
C	2.929920	1.581519	2.122260
C	3.843980	0.446499	2.186200
H	4.226850	0.210059	0.856710
H	0.142050	-0.164320	1.822250
C	4.144290	-1.908861	3.083730
O	5.327250	-1.441301	3.540520
O	3.867699	-3.100711	3.086880
C	3.571340	2.915569	2.190040
O	2.712780	3.950019	2.068270
O	4.771850	3.064969	2.346850
C	6.244340	-2.439861	4.024240
H	6.508399	-3.134891	3.223210
H	7.120930	-1.886581	4.360830
H	5.798569	-2.997601	4.851090
C	3.313021	5.257249	2.075600

H	3.929271	5.393379	2.967270
H	3.925371	5.389619	1.180260
H	2.476651	5.956979	2.072790
C	1.162070	-2.309850	2.670820
H	0.088710	-2.150780	2.566200
H	1.463579	-3.091000	1.968290
H	1.385310	-2.699050	3.668080
C	0.467790	2.354600	1.881480
H	-0.501870	1.861040	1.799920
H	0.448060	3.016900	2.751580
H	0.623700	2.990050	1.007670
P	-1.963620	-0.145570	-0.045790
O	-1.273990	-0.761880	1.147640
O	-1.167420	0.556720	-1.107960
O	-2.896000	-1.256270	-0.839250
O	-3.126820	0.837880	0.615240
H	4.828800	0.692639	2.571650
C	-4.262950	1.204040	-0.072610
C	-5.228260	0.246831	-0.366430
C	-5.599830	2.964531	-1.023820
C	-4.448430	2.591700	-0.358840
H	-5.772200	4.017101	-1.232540
C	-5.079470	-1.156160	0.119750
C	-3.930920	-1.879330	-0.172390
C	-3.801971	-3.272260	0.109310
C	-4.842371	-3.892880	0.770710
H	-4.777041	-4.957440	0.980230
C	-7.682970	2.420531	-2.236040
C	-8.581060	1.493331	-2.710120
C	-8.376280	0.119661	-2.440260
C	-7.302400	-0.297639	-1.685090
C	-6.364320	0.634961	-1.160140
C	-6.553710	2.019291	-1.471840
H	-7.813750	3.478671	-2.450360
H	-9.437030	1.809011	-3.300180
H	-9.071090	-0.615749	-2.836990
H	-7.158620	-1.355099	-1.497290
C	-8.191140	-1.735399	2.170970
C	-7.232610	-1.088819	1.422030
C	-6.107400	-1.788599	0.903660
C	-5.984561	-3.181419	1.214070
C	-7.000771	-3.823819	1.972160
C	-8.085631	-3.120189	2.440500
H	-9.035320	-1.173989	2.562370
H	-7.326860	-0.025909	1.233000
H	-6.894541	-4.884789	2.186040
H	-8.853261	-3.619389	3.025400
C	1.570690	1.340800	2.019100
C	3.796840	1.352269	-0.950430
C	4.319700	0.042499	-0.515400
C	3.442010	-1.095591	-0.830920
C	2.121180	-0.896020	-1.166180
C	2.445730	1.486509	-1.330730
H	0.606700	0.482710	-1.407740
N	1.634920	0.371340	-1.323640

C	1.933960	2.744170	-1.754790
C	2.815020	3.806689	-1.688200
C	4.566540	2.554389	-0.929500
H	2.476501	4.795009	-1.994990
C	-2.605391	-4.064260	-0.312050
C	-2.353911	-4.294800	-1.687390
C	-1.769011	-4.648930	0.671250
C	-3.187031	-3.769490	-2.726730
C	-1.230981	-5.114850	-2.081660
C	-0.665781	-5.490480	0.262350
C	-1.958301	-4.443690	2.077440
C	-2.918041	-4.011230	-4.048320
H	-4.048441	-3.169860	-2.457050
C	-0.981651	-5.338550	-3.472290
C	-0.421781	-5.692360	-1.098580
C	0.151749	-6.106790	1.262000
H	-2.750121	-3.781120	2.405790
C	-1.155041	-5.057060	3.003780
C	-1.796931	-4.801250	-4.431070
H	-3.566851	-3.596680	-4.815050
H	-0.127921	-5.952640	-3.749300
H	0.410639	-6.324930	-1.401050
C	-0.088301	-5.908190	2.594910
H	0.968649	-6.745930	0.934590
H	-1.327231	-4.886850	4.063330
H	-1.597831	-4.979410	-5.484360
H	0.535879	-6.388770	3.343440
C	-3.471520	3.644980	0.058340
C	-2.836939	4.439950	-0.930210
C	-3.255280	3.917230	1.432790
C	-2.991750	4.207660	-2.336910
C	-1.988469	5.540330	-0.527740
C	-2.372699	4.994990	1.819010
C	-3.903300	3.186750	2.480480
C	-2.407339	5.027880	-3.266670
H	-3.579180	3.358440	-2.664540
C	-1.396439	6.371820	-1.530530
C	-1.770389	5.779390	0.831140
C	-2.153849	5.257300	3.208340
H	-4.591710	2.392250	2.219010
C	-3.672180	3.472500	3.800680
C	-1.608229	6.135550	-2.862140
H	-2.546109	4.826690	-4.325470
H	-0.777539	7.205430	-1.206130
H	-1.128479	6.606670	1.128340
C	-2.779529	4.516850	4.174660
H	-1.483979	6.070630	3.478450
H	-4.177200	2.898330	4.572620
H	-1.161609	6.781560	-3.613380
H	-2.608289	4.728610	5.226730
C	5.797110	-0.262941	-0.715290
C	6.653950	-0.592621	0.332260
C	6.293540	-0.320641	-2.040010
C	7.978780	-0.964151	0.108120
H	6.286890	-0.564901	1.351180

C	7.613670	-0.701141	-2.280590
C	8.458500	-1.019581	-1.204670
H	8.632310	-1.212601	0.937210
H	7.998830	-0.756151	-3.290980
C	1.120430	-1.996470	-1.383900
H	0.411160	-2.028460	-0.547790
H	0.535500	-1.817260	-2.290990
H	1.604100	-2.969820	-1.463580
C	0.542060	2.915650	-2.301370
H	-0.237400	2.608350	-1.598950
H	0.369730	3.961070	-2.568550
H	0.394720	2.309030	-3.204600
C	9.816340	-1.404402	-1.460040
N	10.919030	-1.715292	-1.663490
N	4.096360	3.739379	-1.269560
O	5.839940	2.443389	-0.515840
O	5.422600	0.018739	-3.022070
C	5.679930	-0.437681	-4.346440
H	5.835330	-1.521461	-4.353970
H	4.785380	-0.191391	-4.920550
H	6.542370	0.075219	-4.790860
C	6.619940	3.639989	-0.415130
H	6.636400	4.176949	-1.366970
H	6.218720	4.287669	0.367730
H	7.620890	3.299779	-0.146000
C	4.033800	-2.481881	-1.040370
N	4.046059	-3.330341	0.020050
H	3.703999	-3.110101	0.945580
H	4.453899	-4.244661	-0.125580
O	4.464800	-2.811791	-2.140530

S-forming TS Conformation 2

B3LYP/6-31G(d) Energy = -4490.491451
M06-2X/6-311G(d,p)-SMD(tetrahydrofuran) Energy = -4489.999411
M06-2X/6-311G(d,p)-SMD(tetrahydrofuran)-derived Free Energy (Quasiharmonic) = -4488.900932

Frequencies (Top 3 out of 468)

1. -1191.8919 cm^{-1}
2. 6.9571 cm^{-1}
3. 10.6307 cm^{-1}

B3LYP/6-31G(d) Molecular Geometry in Cartesian Coordinates

N	1.067039	-1.744359	0.854109
C	1.439103	-2.241908	-0.368453
C	2.777342	-2.525709	-0.575349
C	3.227484	-1.930160	1.790852
C	3.741956	-2.150726	0.451652
H	4.173749	-0.901694	0.013825
H	0.072441	-1.442763	0.977829
C	3.276226	-3.149750	-1.818019
O	4.503783	-3.692110	-1.640993

O	2.714243	-3.178480	-2.906219
C	4.138499	-2.017472	2.956009
O	5.376750	-2.428110	2.583368
O	3.846678	-1.790534	4.116252
C	5.102204	-4.279149	-2.809752
H	4.445324	-5.040522	-3.235977
H	5.300331	-3.509397	-3.560062
H	6.035453	-4.723227	-2.463207
C	6.312516	-2.613003	3.654986
H	6.474327	-1.676825	4.193836
H	5.946955	-3.367245	4.357150
H	7.235962	-2.945775	3.180176
C	0.306502	-2.443179	-1.328411
H	0.630760	-2.958410	-2.227932
H	-0.496831	-3.004910	-0.844336
H	-0.113763	-1.467945	-1.604105
C	1.181083	-1.347276	3.246751
H	1.722685	-0.580071	3.800808
H	0.154135	-1.029991	3.054959
H	1.164129	-2.238039	3.884378
P	-2.038659	0.076087	0.082796
O	-1.432515	-0.634123	1.268864
O	-1.143097	0.684949	-0.963251
O	-3.088193	1.200538	0.702884
O	-3.088877	-0.931872	-0.718732
H	4.697410	-2.658712	0.408837
C	-4.218469	-1.361726	-0.045201
C	-5.263004	-0.468015	0.154047
C	-5.412246	-3.133920	1.056894
C	-4.288994	-2.724529	0.367118
H	-5.489979	-4.171565	1.371532
C	-5.173098	0.903003	-0.424126
C	-4.085517	1.708275	-0.109697
C	-3.989125	3.064833	-0.537538
C	-4.990226	3.558534	-1.349757
H	-4.940803	4.593580	-1.678253
C	-7.574731	-2.669044	2.161639
C	-8.572153	-1.788972	2.510765
C	-8.489360	-0.437045	2.101037
C	-7.431402	0.003448	1.337016
C	-6.391274	-0.883387	0.940863
C	-6.461818	-2.242025	1.387753
H	-7.614850	-3.707340	2.482526
H	-9.415709	-2.123963	3.108055
H	-9.266457	0.262830	2.396515
H	-7.379731	1.044444	1.039892
C	-8.156089	1.122627	-2.717606
C	-7.229796	0.602565	-1.840930
C	-6.167201	1.402180	-1.333867
C	-6.071479	2.756370	-1.789356
C	-7.052027	3.267546	-2.682234
C	-8.077314	2.471842	-3.136924
H	-8.953457	0.487978	-3.095055
H	-7.301319	-0.435408	-1.537682
H	-6.967131	4.301639	-3.007930

H	-8.818656	2.870534	-3.823963
C	1.878160	-1.664858	1.954217
C	3.687009	1.092136	0.780691
C	4.327979	0.430365	-0.376950
C	3.571610	0.538632	-1.631916
C	2.235721	0.878088	-1.622616
C	2.320416	1.443997	0.721845
H	0.594255	1.284506	-0.467971
N	1.623396	1.208157	-0.446154
C	1.677135	2.063302	1.825913
C	2.474700	2.305063	2.929680
C	4.336166	1.345711	2.023580
H	2.047466	2.811094	3.793927
C	-2.880188	3.978665	-0.117453
C	-1.908045	4.384240	-1.062847
C	-2.872247	4.520298	1.190106
C	-1.844276	3.842617	-2.387991
C	-0.926418	5.381287	-0.695334
C	-1.875898	5.503726	1.549977
C	-3.831980	4.149257	2.185872
C	-0.907378	4.276809	-3.289354
H	-2.545076	3.065054	-2.667228
C	0.033961	5.805237	-1.667400
C	-0.938488	5.913616	0.597089
C	-1.878439	6.046083	2.873517
H	-4.593989	3.420714	1.933015
C	-3.799640	4.691589	3.443832
C	0.041969	5.277200	-2.930188
H	-0.883476	3.851489	-4.289213
H	0.758582	6.562784	-1.377620
H	-0.200364	6.666055	0.868231
C	-2.808965	5.651908	3.796995
H	-1.121524	6.784389	3.128415
H	-4.536619	4.387548	4.182159
H	0.773118	5.612839	-3.660932
H	-2.799672	6.071615	4.799334
C	-3.228124	-3.730848	0.044423
C	-2.384749	-4.221375	1.069839
C	-3.147837	-4.268189	-1.263066
C	-2.395962	-3.689117	2.400508
C	-1.462117	-5.298065	0.782041
C	-2.210061	-5.332425	-1.541980
C	-3.977904	-3.813268	-2.337577
C	-1.583791	-4.202927	3.377609
H	-3.047120	-2.852088	2.621020
C	-0.633211	-5.804536	1.832686
C	-1.401086	-5.823101	-0.512179
C	-2.139619	-5.870131	-2.865391
H	-4.694209	-3.022101	-2.146969
C	-3.879157	-4.355008	-3.592300
C	-0.694893	-5.280580	3.095697
H	-1.612179	-3.781255	4.378742
H	0.047128	-6.621390	1.602633
H	-0.708248	-6.635475	-0.723390
C	-2.946912	-5.396780	-3.864139

H	-1.427382	-6.668948	-3.058869
H	-4.518016	-3.987633	-4.390801
H	-0.064130	-5.678759	3.886210
H	-2.883243	-5.813891	-4.865535
C	5.835140	0.533675	-0.551395
C	6.642638	-0.588784	-0.714422
C	6.428248	1.814239	-0.669222
C	8.012165	-0.483360	-0.957022
H	6.197446	-1.575338	-0.668424
C	7.795173	1.937038	-0.910920
C	8.589659	0.785826	-1.049666
H	8.623461	-1.370989	-1.077642
H	8.258011	2.911348	-1.002401
C	1.378110	1.047415	-2.849118
H	1.379850	2.104937	-3.142828
H	1.746878	0.459170	-3.688907
H	0.341786	0.777821	-2.630449
C	0.223342	2.450291	1.817818
H	-0.060080	2.984294	0.905457
H	-0.427030	1.573197	1.903243
H	0.003957	3.108372	2.663243
C	9.996688	0.922332	-1.291226
N	11.139436	1.030576	-1.482221
N	3.768714	1.953492	3.046801
O	5.611376	0.921923	2.151625
O	5.594648	2.874547	-0.529670
C	6.042524	4.154287	-0.959256
H	6.382563	4.116880	-2.000377
H	5.175369	4.811663	-0.879249
H	6.845063	4.539197	-0.316946
C	6.274975	1.227719	3.384371
H	6.320888	2.308466	3.544717
H	5.753967	0.768486	4.228722
H	7.279156	0.815367	3.272838
C	4.298530	0.490758	-2.966073
N	4.096523	-0.612476	-3.740471
H	3.425835	-1.340243	-3.520056
H	4.497219	-0.596550	-4.669708
O	5.027226	1.410201	-3.318302

S-forming TS Conformation 3

B3LYP/6-31G(d) Energy = -4490.488834
M06-2X/6-311G(d,p)-SMD(tetrahydrofuran) Energy = -4489.996374
M06-2X/6-311G(d,p)-SMD(tetrahydrofuran)-derived Free Energy (Quasiharmonic) = -4488.898176

Frequencies (Top 3 out of 468)

1. -1225.0338 cm^{-1}
2. 7.1576 cm^{-1}
3. 10.1980 cm^{-1}

B3LYP/6-31G(d) Molecular Geometry in Cartesian Coordinates

N	1.082676	0.128878	2.109696
C	1.822518	-0.948924	2.507643
C	3.190917	-0.783652	2.635081
C	2.917860	1.623745	2.132617
C	3.802999	0.462748	2.202975
H	4.219492	0.233073	0.889861
H	0.079485	-0.058180	1.869015
C	4.118521	-1.811590	3.143511
O	3.544061	-2.773035	3.887142
O	5.325097	-1.781993	2.926418
C	3.496874	2.987655	2.207368
O	4.851802	2.936061	2.276583
O	2.889073	4.042081	2.238285
C	4.429813	-3.798454	4.371987
H	4.892707	-4.329268	3.536309
H	5.211814	-3.363007	4.998382
H	3.799353	-4.471687	4.952626
C	5.501042	4.210490	2.396446
H	6.569552	3.994304	2.384113
H	5.224146	4.859392	1.562421
H	5.217971	4.697318	3.333898
C	1.032669	-2.197158	2.787089
H	1.529585	-3.081223	2.384988
H	0.941797	-2.345552	3.868587
H	0.031636	-2.116730	2.361109
C	0.484512	2.443298	1.800402
H	0.908709	3.419683	1.588587
H	-0.147287	2.525086	2.693610
H	-0.162717	2.126108	0.975058
P	-2.006715	-0.068130	0.050289
O	-1.362335	-0.723849	1.247371
O	-1.158700	0.693261	-0.930390
O	-3.218192	0.870224	0.679125
O	-2.877330	-1.156883	-0.840009
H	4.790246	0.655315	2.610839
C	-3.941921	-1.812775	-0.253299
C	-5.114166	-1.111948	-0.001008
C	-4.877454	-3.864687	0.578370
C	-3.811226	-3.212245	-0.006818
H	-4.809018	-4.934312	0.759232
C	-5.252383	0.306092	-0.444119
C	-4.312195	1.256883	-0.063780
C	-4.484997	2.652695	-0.316480
C	-5.603003	3.044131	-1.025990
H	-5.768187	4.102412	-1.210292
C	-7.098420	-3.857726	1.661913
C	-8.215671	-3.181058	2.092235
C	-8.323828	-1.789677	1.860202
C	-7.334764	-1.109556	1.184280
C	-6.174834	-1.780522	0.705623
C	-6.051246	-3.180705	0.979893
H	-6.990222	-4.923606	1.848752
H	-9.007229	-3.706659	2.619287
H	-9.194509	-1.249879	2.222895
H	-7.431796	-0.042258	1.023945

C	-8.288693	0.239763	-2.680743
C	-7.251760	-0.199898	-1.887542
C	-6.347649	0.717138	-1.282031
C	-6.528904	2.111300	-1.552897
C	-7.620532	2.534739	-2.358720
C	-8.487863	1.621449	-2.910815
H	-8.958128	-0.483737	-3.138377
H	-7.111168	-1.262804	-1.730241
H	-7.746824	3.599346	-2.541426
H	-9.314839	1.954765	-3.531770
C	1.554214	1.413990	2.017071
C	3.829091	1.358609	-0.980389
C	4.336698	0.055085	-0.494993
C	3.454011	-1.088504	-0.784434
C	2.121325	-0.880925	-1.091151
C	2.469785	1.494153	-1.339052
H	0.621147	0.513178	-1.315715
N	1.648745	0.388279	-1.258097
C	1.961181	2.724555	-1.835419
C	2.864591	3.768857	-1.893954
C	4.621291	2.540848	-1.103687
H	2.534619	4.737526	-2.264800
C	-3.519723	3.684849	0.172793
C	-3.351761	3.905976	1.562711
C	-2.831573	4.498756	-0.763490
C	-4.062675	3.161830	2.558509
C	-2.452325	4.940967	2.019453
C	-1.966492	5.557285	-0.290250
C	-2.945253	4.327001	-2.182757
C	-3.873442	3.394416	3.895532
H	-4.766082	2.400523	2.243183
C	-2.275630	5.146017	3.424291
C	-1.788660	5.739188	1.083409
C	-1.314400	6.407087	-1.238712
H	-3.545823	3.510176	-2.564380
C	-2.308294	5.166863	-3.059132
C	-2.960663	4.393350	4.339442
H	-4.426194	2.811579	4.627329
H	-1.586260	5.922629	3.747204
H	-1.127478	6.529401	1.433390
C	-1.489093	6.231330	-2.584908
H	-0.679412	7.204822	-0.860282
H	-2.420212	5.013565	-4.129221
H	-2.820414	4.560575	5.404050
H	-0.998528	6.892487	-3.294507
C	-2.586235	-3.981471	-0.387091
C	-1.790811	-4.578975	0.621782
C	-2.269525	-4.181666	-1.753487
C	-2.044821	-4.400482	2.021465
C	-0.663816	-5.404844	0.247582
C	-1.123564	-4.986105	-2.111693
C	-3.056577	-3.638386	-2.819163
C	-1.279424	-5.025236	2.971879
H	-2.854943	-3.748525	2.325543
C	0.111950	-6.034965	1.271541

C	-0.356290	-5.578747	-1.104262
C	-0.807008	-5.177449	-3.493337
H	-3.933420	-3.049283	-2.577202
C	-2.723835	-3.849718	-4.131471
C	-0.190109	-5.862923	2.595606
H	-1.500476	-4.875377	4.025456
H	0.947114	-6.662557	0.968851
H	0.496305	-6.195852	-1.380592
C	-1.580225	-4.624301	-4.477654
H	0.065178	-5.777150	-3.742333
H	-3.338551	-3.422185	-4.918950
H	0.400033	-6.357349	3.362941
H	-1.329871	-4.777251	-5.523969
C	5.818549	-0.232566	-0.710894
C	6.754483	-0.296558	0.319466
C	6.251170	-0.497414	-2.033954
C	8.087923	-0.622887	0.080435
H	6.437772	-0.116548	1.338568
C	7.581441	-0.834810	-2.287767
C	8.501867	-0.894965	-1.228461
H	8.797461	-0.676319	0.898838
H	7.917949	-1.047234	-3.294723
C	1.076989	-1.952812	-1.226561
H	1.516849	-2.945980	-1.239262
H	0.364643	-1.877215	-0.396186
H	0.504307	-1.815322	-2.149247
C	0.556849	2.885457	-2.351761
H	0.373635	2.224581	-3.209366
H	-0.208248	2.642184	-1.610673
H	0.396055	3.914826	-2.680711
C	9.867646	-1.239644	-1.498212
N	10.976238	-1.517547	-1.716666
N	4.161489	3.697283	-1.537261
O	5.921981	2.459187	-0.754478
O	5.306788	-0.394607	-3.004305
C	5.569478	-0.959110	-4.284379
H	5.845385	-2.016044	-4.195542
H	4.634641	-0.873837	-4.839515
H	6.357350	-0.408677	-4.814039
C	6.757042	3.579735	-1.062204
H	6.738320	3.795688	-2.134436
H	6.437455	4.474053	-0.521657
H	7.758421	3.278297	-0.750342
C	3.998875	-2.494401	-0.891906
N	4.735807	-2.973514	0.154582
H	5.112956	-2.378251	0.881494
H	5.232608	-3.836561	-0.026576
O	3.762075	-3.192386	-1.874839

S-forming TS Conformation 4

B3LYP/6-31G(d) Energy = -4490.486442
M06-2X/6-311G(d,p)-SMD(tetrahydrofuran) Energy = -4489.996915
M06-2X/6-311G(d,p)-SMD(tetrahydrofuran)-derived Free Energy (Quasiharmonic) = -4488.899101

Frequencies (Top 3 out of 468)

1. -1191.7252 cm^{-1}
2. 7.1798 cm^{-1}
3. 9.8840 cm^{-1}

B3LYP/6-31G(d) Molecular Geometry in Cartesian Coordinates

N	1.144219	0.072375	2.112865
C	1.575524	1.370877	2.044886
C	2.934658	1.611127	2.146655
C	3.277935	-0.799115	2.616349
C	3.843211	0.474325	2.206999
H	4.228617	0.241550	0.875815
H	0.152698	-0.134498	1.844852
C	3.575699	2.945709	2.222615
O	2.714885	3.979775	2.108719
O	4.775054	3.095420	2.381782
C	4.249008	-1.778528	3.154167
O	3.749279	-3.030009	3.316831
O	5.406673	-1.503452	3.414167
C	3.313935	5.287582	2.130969
H	3.932456	5.427951	1.241175
H	2.476964	5.986633	2.128705
H	3.924032	5.416385	3.027968
C	4.674912	-3.991579	3.859485
H	5.537412	-4.106112	3.198349
H	5.021395	-3.672070	4.844970
H	4.113057	-4.923042	3.931289
C	0.472039	2.383211	1.901834
H	0.440851	3.037651	2.777738
H	0.637114	3.026408	1.035662
H	-0.495836	1.888787	1.805188
C	1.133710	-2.259430	2.746898
H	0.081029	-2.112775	2.503220
H	1.505750	-3.105637	2.165764
H	1.225170	-2.541492	3.800446
P	-1.938165	-0.120990	-0.056436
O	-1.244634	-0.749127	1.128780
O	-1.151089	0.615341	-1.101642
O	-2.846003	-1.235910	-0.874161
O	-3.122596	0.827349	0.616607
H	4.833169	0.703462	2.591787
C	-4.256565	1.190004	-0.076972
C	-5.204818	0.224034	-0.395903
C	-5.607463	2.946331	-1.014599
C	-4.457495	2.579118	-0.344064
H	-5.791926	3.999467	-1.209666
C	-5.038549	-1.184441	0.068430
C	-3.876272	-1.884925	-0.225590
C	-3.728432	-3.280143	0.035688
C	-4.764695	-3.927121	0.678328
H	-4.685163	-4.993899	0.871038
C	-7.671915	2.392902	-2.254216

C	-8.552972	1.461167	-2.750827
C	-8.332057	0.086354	-2.500274
C	-7.259429	-0.328148	-1.741776
C	-6.338957	0.608738	-1.193864
C	-6.544290	1.995174	-1.485779
H	-7.815169	3.452362	-2.453440
H	-9.407771	1.774442	-3.343855
H	-9.013278	-0.652047	-2.914623
H	-7.103048	-1.386396	-1.568944
C	-8.158054	-1.843818	2.082893
C	-7.203230	-1.170795	1.352778
C	-6.063008	-1.845029	0.833330
C	-5.921484	-3.240550	1.122776
C	-6.934162	-3.910512	1.861513
C	-8.033648	-3.231010	2.331338
H	-9.014105	-1.301696	2.475645
H	-7.312167	-0.106599	1.179670
H	-6.813635	-4.973079	2.059327
H	-8.798594	-3.751099	2.901339
C	1.914610	-0.999890	2.476346
C	3.816589	1.415121	-0.916337
C	4.330712	0.097576	-0.492098
C	3.447458	-1.029484	-0.832823
C	2.132962	-0.822036	-1.180890
C	2.469064	1.560609	-1.306206
H	0.626816	0.565341	-1.414163
N	1.653317	0.449513	-1.326448
C	1.964344	2.826442	-1.713279
C	2.849640	3.884021	-1.627434
C	4.592187	2.613339	-0.877010
H	2.516814	4.878277	-1.921286
C	-2.518059	-4.049472	-0.388754
C	-1.685404	-4.645285	0.591238
C	-2.250929	-4.252386	-1.765701
C	-1.889567	-4.468050	1.999257
C	-0.572077	-5.471007	0.177426
C	-1.117198	-5.055470	-2.164212
C	-3.078822	-3.715447	-2.803231
C	-1.092925	-5.095013	2.922262
H	-2.689048	-3.816971	2.331998
C	0.237963	-6.103121	1.173467
C	-0.312995	-5.645377	-1.184378
C	-0.852320	-5.251223	-3.556155
H	-3.948084	-3.128544	-2.531155
C	-2.794810	-3.930322	-4.126315
C	-0.018129	-5.933313	2.507587
H	-1.278245	-4.947556	3.982998
H	1.058788	-6.735009	0.841229
H	0.526549	-6.266518	-1.490823
C	-1.663061	-4.703180	-4.512640
H	0.009409	-5.852592	-3.836144
H	-3.439880	-3.507384	-4.891560
H	0.595654	-6.431719	3.253553
H	-1.452225	-4.859840	-5.567028
C	-3.497438	3.637998	0.097306

C	-2.862454	4.453664	-0.873897
C	-3.297483	3.893141	1.477430
C	-3.000331	4.238793	-2.285089
C	-2.031056	5.558174	-0.447878
C	-2.430821	4.975117	1.887198
C	-3.947014	3.140720	2.508496
C	-2.416706	5.078759	-3.197542
H	-3.573893	3.387037	-2.630234
C	-1.439579	6.410747	-1.433112
C	-1.828528	5.780389	0.916257
C	-2.227790	5.219998	3.282095
H	-4.624089	2.342470	2.229280
C	-3.731512	3.410305	3.834727
C	-1.635457	6.190473	-2.769866
H	-2.542399	4.890531	-4.260353
H	-0.833996	7.247063	-1.091090
H	-1.198992	6.610575	1.231219
C	-2.854098	4.459017	4.231882
H	-1.569490	6.036642	3.569984
H	-4.237380	2.819795	4.593695
H	-1.189538	6.852257	-3.507672
H	-2.694955	4.657619	5.288398
C	5.807935	-0.216793	-0.685938
C	6.653588	-0.579341	0.360179
C	6.311898	-0.250052	-2.008778
C	7.977766	-0.953792	0.134008
H	6.283584	-0.587052	1.378545
C	7.631542	-0.629155	-2.250063
C	8.467318	-0.977426	-1.175630
H	8.619644	-1.230741	0.962998
H	8.023609	-0.662771	-3.258688
C	1.131360	-1.916953	-1.423163
H	0.439625	-1.988258	-0.574958
H	0.527085	-1.702552	-2.309396
H	1.617604	-2.883477	-1.555885
C	0.574356	3.009865	-2.261334
H	0.429976	2.423079	-3.178129
H	-0.207801	2.688374	-1.567976
H	0.402280	4.060761	-2.506492
C	9.824465	-1.363231	-1.433630
N	10.925853	-1.675060	-1.642958
N	4.128810	3.804993	-1.204866
O	5.862133	2.490757	-0.459744
O	5.444683	0.106930	-2.990386
C	5.727825	-0.296097	-4.325626
H	6.589107	0.244741	-4.738271
H	5.902072	-1.376492	-4.372705
H	4.838232	-0.042394	-4.904413
C	6.648733	3.681295	-0.337897
H	6.675515	4.229831	-1.282925
H	6.245334	4.321019	0.450208
H	7.645229	3.331093	-0.065528
C	4.028859	-2.421592	-1.010512
N	3.999590	-3.241700	0.076591
H	4.370418	-4.176612	-0.029895

H	3.574643	-2.984901	0.954133
O	4.489929	-2.785969	-2.085134

S-forming TS Conformation 5

B3LYP/6-31G(d) Energy = -4490.489105
M06-2X/6-311G(d,p)-SMD(tetrahydrofuran) Energy = -4489.997345
M06-2X/6-311G(d,p)-SMD(tetrahydrofuran)-derived Free Energy (Quasiharmonic) = -4488.898958

Frequencies (Top 3 out of 468)

1. -1221.7880 cm^{-1}
2. 7.3995 cm^{-1}
3. 9.7268 cm^{-1}

B3LYP/6-31G(d) Molecular Geometry in Cartesian Coordinates

N	1.084402	-0.004935	2.115167
C	1.829685	-1.105341	2.438699
C	3.196767	-0.945175	2.582920
C	2.920713	1.481646	2.215161
C	3.810102	0.324323	2.223619
H	4.234443	0.158977	0.902409
H	0.083907	-0.177265	1.849871
C	4.122797	-1.999753	3.038182
O	3.543654	-3.004106	3.718415
O	5.331174	-1.954209	2.835306
C	3.467003	2.848677	2.350547
O	4.820083	2.833556	2.458370
O	2.819171	3.879261	2.389278
C	4.427022	-4.055047	4.150282
H	4.898137	-4.535151	3.288936
H	5.202549	-3.656015	4.808178
H	3.792622	-4.762049	4.684621
C	5.427020	4.122821	2.630063
H	6.501742	3.939532	2.640573
H	5.151951	4.786490	1.807002
H	5.105541	4.574699	3.572711
C	1.040100	-2.369619	2.632861
H	1.545047	-3.228471	2.188946
H	0.930398	-2.580569	3.702130
H	0.045228	-2.266120	2.197002
C	0.499489	2.343792	2.004710
H	0.726201	3.050287	1.204086
H	0.468048	2.933012	2.926278
H	-0.483619	1.899802	1.838367
P	-1.987000	-0.095140	-0.038198
O	-1.338787	-0.751995	1.157313
O	-1.146496	0.610482	-1.062880
O	-3.143334	0.897764	0.618092
O	-2.923877	-1.166691	-0.878054
H	4.795367	0.498517	2.645581
C	-3.980404	-1.788347	-0.244533
C	-5.121630	-1.053047	0.045809

C	-4.940652	-3.808860	0.632913
C	-3.878499	-3.189759	0.005565
H	-4.896724	-4.879231	0.816891
C	-5.239443	0.363810	-0.407865
C	-4.263368	1.297783	-0.076833
C	-4.423147	2.695547	-0.329329
C	-5.559664	3.103519	-0.998945
H	-5.713072	4.163801	-1.181853
C	-7.118178	-3.732901	1.798785
C	-8.198852	-3.022700	2.266937
C	-8.276328	-1.629931	2.031202
C	-7.294574	-0.981429	1.314682
C	-6.172839	-1.687243	0.796992
C	-6.078548	-3.089260	1.074242
H	-7.033387	-4.800602	1.987377
H	-8.984580	-3.523169	2.826195
H	-9.117241	-1.063933	2.423030
H	-7.367580	0.087365	1.151352
C	-8.358690	0.340287	-2.528802
C	-7.302102	-0.113277	-1.770094
C	-6.357634	0.790404	-1.207439
C	-6.521459	2.185242	-1.484924
C	-7.633528	2.623837	-2.253751
C	-8.538803	1.723571	-2.764745
H	-9.058717	-0.373630	-2.954610
H	-7.176987	-1.177443	-1.608558
H	-7.745141	3.689178	-2.441667
H	-9.381393	2.068034	-3.358005
C	1.554988	1.280160	2.104755
C	3.839657	1.369428	-0.905584
C	4.355470	0.046504	-0.486996
C	3.480791	-1.086703	-0.835999
C	2.147825	-0.872200	-1.138104
C	2.479449	1.514547	-1.255872
H	0.636781	0.516970	-1.313949
N	1.666067	0.400343	-1.238716
C	1.961933	2.769012	-1.678078
C	2.858568	3.820884	-1.679426
C	4.623720	2.562080	-0.961265
H	2.520987	4.806751	-1.993742
C	-3.431672	3.718524	0.126543
C	-3.222075	3.945075	1.510254
C	-2.768822	4.526489	-0.832568
C	-3.903342	3.205775	2.530279
C	-2.309862	4.982347	1.935791
C	-1.891084	5.587733	-0.389677
C	-2.920491	4.344953	-2.247125
C	-3.675956	3.445872	3.860160
H	-4.614919	2.442277	2.239673
C	-2.093536	5.195492	3.333915
C	-1.674271	5.776691	0.977262
C	-1.266703	6.432701	-1.361095
H	-3.530500	3.524965	-2.606498
C	-2.306465	5.177986	-3.145996
C	-2.752233	4.448018	4.272440

H	-4.207222	2.866652	4.610518
H	-1.396463	5.974930	3.632609
H	-1.003895	6.568881	1.304442
C	-1.476869	6.247111	-2.700997
H	-0.623762	7.234510	-1.005223
H	-2.444933	5.016079	-4.211643
H	-2.582520	4.621801	5.331698
H	-1.005928	6.903478	-3.428080
C	-2.686215	-3.989546	-0.413074
C	-1.880510	-4.616672	0.569877
C	-2.407268	-4.182784	-1.788944
C	-2.098118	-4.452656	1.977544
C	-0.779760	-5.460687	0.159423
C	-1.284821	-5.003322	-2.183176
C	-3.211678	-3.617727	-2.830005
C	-1.326649	-5.108758	2.901676
H	-2.886206	-3.787531	2.309629
C	0.004163	-6.122748	1.156641
C	-0.505364	-5.621604	-1.201027
C	-1.005138	-5.186165	-3.573917
H	-4.073100	-3.017572	-2.561577
C	-2.915406	-3.822511	-4.151983
C	-0.265188	-5.964907	2.489473
H	-1.521407	-4.969741	3.961942
H	0.818247	-6.763931	0.826202
H	0.328409	-6.251666	-1.504369
C	-1.793147	-4.611380	-4.533676
H	-0.149861	-5.798348	-3.849872
H	-3.542945	-3.378413	-4.919910
H	0.330057	-6.484146	3.236191
H	-1.571243	-4.758334	-5.587239
C	5.839564	-0.220346	-0.713807
C	6.772482	-0.336425	0.314710
C	6.278117	-0.408198	-2.048068
C	8.108917	-0.640096	0.062672
H	6.451160	-0.215909	1.341187
C	7.611510	-0.721940	-2.315537
C	8.528924	-0.835287	-1.257918
H	8.816157	-0.734590	0.879352
H	7.952878	-0.875403	-3.331509
C	1.112186	-1.943704	-1.336166
H	1.558713	-2.932756	-1.386843
H	0.387957	-1.907584	-0.513763
H	0.550817	-1.767182	-2.259091
C	0.551388	2.951933	-2.168467
H	0.349072	2.319950	-3.042668
H	-0.200872	2.683554	-1.422715
H	0.389399	3.992143	-2.460271
C	9.897928	-1.155013	-1.541671
N	11.009140	-1.412693	-1.771326
N	4.156874	3.738013	-1.329707
O	5.924150	2.468791	-0.613990
O	5.335924	-0.257991	-3.014201
C	5.610995	-0.737153	-4.325975
H	5.898100	-1.794646	-4.303319

H	4.678082	-0.626447	-4.879843
H	6.395611	-0.145927	-4.814966
C	6.751679	3.612142	-0.848850
H	6.735215	3.893618	-1.905810
H	6.423248	4.469024	-0.255546
H	7.754155	3.299574	-0.551861
C	4.035007	-2.481484	-1.015997
N	4.772500	-3.011433	0.005731
H	5.146698	-2.452105	0.762021
H	5.275853	-3.859820	-0.221072
O	3.804302	-3.128262	-2.034613

S-forming TS Conformation 6

B3LYP/6-31G(d) Energy = -4490.486333
M06-2X/6-311G(d,p)-SMD(tetrahydrofuran) Energy = -4489.995803
M06-2X/6-311G(d,p)-SMD(tetrahydrofuran)-derived Free Energy (Quasiharmonic) = -4488.896426

Frequencies (Top 3 out of 468)

1. -1230.5549 cm^{-1}
2. 6.8320 cm^{-1}
3. 11.1305 cm^{-1}

B3LYP/6-31G(d) Molecular Geometry in Cartesian Coordinates

N	1.205648	-1.523765	0.803442
C	1.590149	-2.025945	-0.413926
C	2.934142	-2.275903	-0.616943
C	3.369227	-1.663481	1.738666
C	3.892323	-1.839331	0.392376
H	4.268929	-0.587348	-0.043560
H	0.202042	-1.260351	0.928534
C	3.401695	-3.017696	-1.809074
O	4.452804	-3.817784	-1.526152
O	2.919517	-2.969317	-2.934040
C	4.368878	-1.808919	2.824759
O	3.850142	-1.725194	4.072279
O	5.549596	-2.027636	2.620635
C	4.935930	-4.607643	-2.627955
H	5.748200	-5.208789	-2.218742
H	4.140536	-5.249148	-3.014411
H	5.297933	-3.963795	-3.433583
C	4.780787	-1.960752	5.140908
H	4.196303	-1.876659	6.057548
H	5.220083	-2.957907	5.053048
H	5.582564	-1.218674	5.127640
C	0.458595	-2.293433	-1.360551
H	-0.034279	-1.347744	-1.616514
H	-0.295181	-2.919847	-0.875466
H	0.802204	-2.770827	-2.274166
C	1.283004	-1.153225	3.192791
H	1.807597	-0.400677	3.781786
H	0.264818	-0.820885	2.980053

H	1.230608	-2.063845	3.799053
P	-2.028047	0.109000	0.051496
O	-1.361215	-0.546184	1.236645
O	-1.187932	0.754645	-1.017863
O	-3.030437	-0.970012	-0.718828
O	-3.132920	1.178831	0.673359
H	4.880636	-2.282148	0.350787
C	-4.163987	1.621635	-0.135383
C	-5.209064	0.754256	-0.427978
C	-5.179152	3.402312	-1.391347
C	-4.144999	2.975174	-0.582845
H	-5.188899	4.433672	-1.734655
C	-5.218439	-0.611290	0.169634
C	-4.128214	-1.449317	-0.027312
C	-4.120398	-2.807773	0.404758
C	-5.213499	-3.268509	1.110430
H	-5.231584	-4.304308	1.439923
C	-7.234464	2.981390	-2.698881
C	-8.219053	2.124945	-3.132720
C	-8.220410	0.779397	-2.694675
C	-7.259252	0.322639	-1.820249
C	-6.237264	1.185805	-1.334356
C	-6.219424	2.536675	-1.808985
H	-7.208826	4.013870	-3.039383
H	-8.987161	2.473189	-3.817771
H	-8.985352	0.097159	-3.055882
H	-7.271314	-0.713501	-1.502776
C	-8.424224	-0.729169	2.147017
C	-7.398543	-0.242329	1.367271
C	-6.315114	-1.076626	0.972996
C	-6.307545	-2.430780	1.438702
C	-7.388787	-2.907060	2.228590
C	-8.429689	-2.077874	2.575322
H	-9.235723	-0.068598	2.440488
H	-7.406217	0.795779	1.055996
H	-7.369487	-3.941529	2.563508
H	-9.248618	-2.450058	3.184752
C	2.012464	-1.439652	1.907710
C	3.601055	1.375026	0.714012
C	4.294261	0.777960	-0.449040
C	3.524826	0.793804	-1.700450
C	2.175591	1.082892	-1.694517
C	2.224401	1.679513	0.647437
H	0.517362	1.461308	-0.554000
N	1.548570	1.424769	-0.526492
C	1.555674	2.267462	1.754219
C	2.332646	2.499971	2.874985
C	4.220581	1.582543	1.983030
H	1.883814	2.986897	3.739361
C	-3.007808	-3.758161	0.087107
C	-2.904806	-4.306137	-1.214322
C	-2.134388	-4.190461	1.113502
C	-3.762955	-3.908965	-2.289666
C	-1.913356	-5.322700	-1.485419
C	-1.157687	-5.220542	0.834095

C	-2.168381	-3.643748	2.437795
C	-3.641740	-4.460077	-3.538287
H	-4.518878	-3.154193	-2.104754
C	-1.821429	-5.872622	-2.802498
C	-1.074836	-5.757410	-0.454063
C	-0.300437	-5.671588	1.887166
H	-2.861104	-2.838745	2.650857
C	-1.328558	-4.104580	3.417961
C	-2.657356	-5.454799	-3.802450
H	-4.302678	-4.136591	-4.337763
H	-1.069511	-6.635715	-2.990124
H	-0.341900	-6.535599	-0.658719
C	-0.386199	-5.138211	3.144849
H	0.420751	-6.454544	1.663841
H	-1.376904	-3.674383	4.414783
H	-2.576864	-5.880545	-4.798988
H	0.265689	-5.495955	3.937644
C	-3.082963	3.953647	-0.187498
C	-2.141298	4.394616	-1.147796
C	-3.094257	4.516948	1.110815
C	-2.059000	3.835353	-2.464559
C	-1.211133	5.448225	-0.804770
C	-2.149323	5.558199	1.446000
C	-4.025465	4.112391	2.120573
C	-1.152534	4.303073	-3.380152
H	-2.719400	3.017149	-2.725428
C	-0.280916	5.905525	-1.791003
C	-1.242042	6.001015	0.478790
C	-2.171319	6.122343	2.760250
H	-4.749437	3.340184	1.886050
C	-4.013210	4.677371	3.368916
C	-0.254115	5.357350	-3.044918
H	-1.113104	3.862739	-4.372971
H	0.405759	6.703959	-1.519396
H	-0.542945	6.796015	0.731218
C	-3.072792	5.695170	3.697797
H	-1.453375	6.904541	2.996325
H	-4.727880	4.347528	4.118063
H	0.454029	5.717805	-3.786323
H	-3.079056	6.132446	4.692647
C	5.764624	1.145055	-0.601966
C	6.111182	2.493713	-0.467940
C	6.781940	0.229967	-0.935988
C	7.417267	2.943083	-0.612271
H	5.327570	3.210597	-0.245634
C	8.103785	0.660372	-1.071039
C	8.423808	2.015965	-0.906796
H	7.657163	3.995013	-0.503590
H	8.894099	-0.037325	-1.314493
C	1.306237	1.203021	-2.919084
H	1.249075	2.259750	-3.210773
H	1.704083	0.636138	-3.758925
H	0.286504	0.880139	-2.694438
C	0.095438	2.628192	1.735169
H	-0.140046	3.291855	2.571930

H	-0.193630	3.145847	0.815434
H	-0.537128	1.739341	1.828728
C	9.786054	2.440483	-1.046599
N	10.893513	2.781180	-1.154409
N	3.627055	2.154374	3.014091
O	5.485780	1.141947	2.122794
O	6.408211	-1.068314	-1.127620
C	7.427493	-2.047774	-1.287281
H	7.974981	-1.906597	-2.227901
H	8.130696	-2.029068	-0.446229
H	6.909796	-3.006146	-1.303342
C	6.148838	1.409556	3.362236
H	7.118295	0.918602	3.274368
H	6.272150	2.486590	3.510190
H	5.582868	1.003494	4.204192
C	4.254469	0.711652	-3.032519
N	3.973022	-0.359874	-3.820936
H	4.449402	-0.409189	-4.711741
H	3.411272	-1.151710	-3.525688
O	5.031636	1.594312	-3.376001

S-forming TS Conformation 7

B3LYP/6-31G(d) Energy = -4490.491104
M06-2X/6-311G(d,p)-SMD(tetrahydrofuran) Energy = -4489.999777
M06-2X/6-311G(d,p)-SMD(tetrahydrofuran)-derived Free Energy (Quasiharmonic) = -4488.901182

Frequencies (Top 3 out of 468)

1. -1181.2145 cm^{-1}
2. 7.5959 cm^{-1}
3. 11.0110 cm^{-1}

B3LYP/6-31G(d) Molecular Geometry in Cartesian Coordinates

N	1.071564	-1.796016	0.776725
C	1.467077	-2.294926	-0.435895
C	2.809473	-2.594664	-0.611557
C	3.203808	-2.011871	1.764251
C	3.750235	-2.212329	0.432133
H	4.215063	-0.949038	0.050541
H	0.078973	-1.483560	0.883259
C	3.292370	-3.337996	-1.796040
O	4.661845	-3.417541	-1.805514
O	2.624447	-3.870352	-2.660072
C	4.193485	-2.109044	2.862157
O	3.659605	-2.071683	4.101889
O	5.389870	-2.243948	2.664515
C	5.209724	-4.233207	-2.857687
H	6.288807	-4.222341	-2.700498
H	4.820991	-5.251812	-2.789335
H	4.953071	-3.819982	-3.836350
C	4.598964	-2.206211	5.181360
H	5.326674	-1.391234	5.162635

H	4.000711	-2.165466	6.091815
H	5.130110	-3.158776	5.109760
C	0.365261	-2.455677	-1.438102
H	0.752885	-2.739355	-2.411876
H	-0.330830	-3.233458	-1.106209
H	-0.198689	-1.519494	-1.513195
C	1.106868	-1.444015	3.174038
H	0.104846	-1.077347	2.943198
H	1.013736	-2.355466	3.774364
H	1.644422	-0.711882	3.777210
P	-2.023224	0.072614	0.059719
O	-1.430240	-0.671186	1.232502
O	-1.109257	0.673630	-0.974374
O	-3.045417	1.212575	0.697553
O	-3.096884	-0.894760	-0.757666
H	4.709052	-2.719265	0.415936
C	-4.229362	-1.317276	-0.084313
C	-5.255686	-0.408739	0.138576
C	-5.445664	-3.088312	0.993374
C	-4.320469	-2.686025	0.302664
H	-5.540049	-4.130210	1.288822
C	-5.144304	0.969233	-0.418750
C	-4.039037	1.749307	-0.101756
C	-3.919653	3.108894	-0.513839
C	-4.915769	3.631519	-1.313824
H	-4.847795	4.669199	-1.630486
C	-7.591677	-2.605125	2.122545
C	-8.571153	-1.714211	2.494438
C	-8.467696	-0.356785	2.108289
C	-7.407324	0.078888	1.344817
C	-6.385566	-0.818604	0.925818
C	-6.476661	-2.183864	1.348539
H	-7.648125	-3.648258	2.424790
H	-9.416506	-2.045295	3.091390
H	-9.230674	0.350933	2.421376
H	-7.339448	1.123866	1.065293
C	-8.137296	1.274733	-2.689481
C	-7.215916	0.726224	-1.825063
C	-6.134682	1.498838	-1.315389
C	-6.015393	2.856122	-1.755651
C	-6.991203	3.397024	-2.636076
C	-8.034759	2.627031	-3.093401
H	-8.949117	0.660207	-3.069409
H	-7.305837	-0.313833	-1.534211
H	-6.887795	4.432959	-2.950333
H	-8.772289	3.048196	-3.771068
C	1.852850	-1.749080	1.903447
C	3.716132	1.062012	0.830702
C	4.381528	0.384894	-0.309190
C	3.653213	0.501960	-1.579848
C	2.313022	0.830712	-1.599263
C	2.345352	1.395424	0.745885
H	0.643543	1.214982	-0.476295
N	1.673407	1.146851	-0.435415
C	1.674000	2.009407	1.835954

C	2.446482	2.261635	2.955522
C	4.348411	1.357240	2.075256
H	1.991914	2.754830	3.813349
C	-2.792682	3.998611	-0.089149
C	-1.804532	4.378761	-1.028188
C	-2.783572	4.544766	1.216357
C	-1.741670	3.829110	-2.350042
C	-0.804007	5.355193	-0.656234
C	-1.769392	5.508149	1.580548
C	-3.758002	4.196531	2.206121
C	-0.785217	4.235043	-3.243681
H	-2.457642	3.066503	-2.631960
C	0.174961	5.751687	-1.621323
C	-0.815501	5.893515	0.633737
C	-1.771176	6.055305	2.902144
H	-4.532536	3.482526	1.949521
C	-3.724131	4.742472	3.462536
C	0.183385	5.215084	-2.880428
H	-0.759164	3.801579	-4.239811
H	0.914148	6.493857	-1.328480
H	-0.063127	6.630573	0.907988
C	-2.716799	5.683832	3.819787
H	-1.001186	6.778816	3.160390
H	-4.472814	4.456265	4.196257
H	0.930142	5.527517	-3.605612
H	-2.706795	6.107376	4.820543
C	-3.277865	-3.700769	-0.050119
C	-2.438472	-4.231416	0.958072
C	-3.208817	-4.201476	-1.372576
C	-2.440583	-3.739556	2.304165
C	-1.530141	-5.310467	0.636413
C	-2.284274	-5.267752	-1.685522
C	-4.037865	-3.707333	-2.430415
C	-1.634949	-4.294180	3.264366
H	-3.080896	-2.901508	2.551137
C	-0.706361	-5.859305	1.669654
C	-1.477762	-5.797670	-0.673054
C	-2.222989	-5.767389	-3.024180
H	-4.745161	-2.914498	-2.214940
C	-3.949201	-4.214072	-3.700363
C	-0.760441	-5.374233	2.948492
H	-1.658941	-3.904185	4.278543
H	-0.036845	-6.677233	1.413319
H	-0.793302	-6.609559	-0.910944
C	-3.028524	-5.256833	-4.005709
H	-1.517668	-6.565424	-3.243546
H	-4.586733	-3.817078	-4.485639
H	-0.135178	-5.806129	3.725698
H	-2.971418	-5.644295	-5.019294
C	5.893571	0.527731	-0.440894
C	6.793038	-0.488436	-0.119018
C	6.403561	1.743721	-0.957207
C	8.161517	-0.350773	-0.338290
H	6.426692	-1.395320	0.346442
C	7.770752	1.888731	-1.200858

C	8.649917	0.837612	-0.895321
H	8.844980	-1.152944	-0.082014
H	8.165767	2.807763	-1.615350
C	1.473562	0.927339	-2.844713
H	1.795552	0.208751	-3.599979
H	0.419369	0.772431	-2.605772
H	1.586924	1.927466	-3.276591
C	0.216729	2.380755	1.804295
H	-0.062253	2.893823	0.879249
H	-0.425112	1.498747	1.898541
H	-0.018353	3.052361	2.634581
C	10.052950	0.993125	-1.148884
N	11.191353	1.117032	-1.355380
N	3.747200	1.945914	3.092748
O	5.640631	1.003620	2.211282
O	5.495034	2.727500	-1.178646
C	5.843460	3.808980	-2.037232
H	6.182380	3.437554	-3.010337
H	4.926004	4.383212	-2.170046
H	6.611119	4.450160	-1.585916
C	6.293452	1.332403	3.440065
H	7.305354	0.939406	3.334292
H	6.312727	2.415182	3.594118
H	5.786636	0.868116	4.290524
C	4.362226	0.404225	-2.910727
N	5.079424	-0.728329	-3.147117
H	5.192644	-1.464450	-2.463442
H	5.647181	-0.748541	-3.983309
O	4.279708	1.303366	-3.743085

S-forming TS Conformation 8

B3LYP/6-31G(d) Energy = -4490.490493
M06-2X/6-311G(d,p)-SMD(tetrahydrofuran) Energy = -4489.998005
M06-2X/6-311G(d,p)-SMD(tetrahydrofuran)-derived Free Energy (Quasiharmonic) = -4488.899208

Frequencies (Top 3 out of 468)

1. -1219.5495 cm^{-1}
2. 7.3591 cm^{-1}
3. 9.8023 cm^{-1}

B3LYP/6-31G(d) Molecular Geometry in Cartesian Coordinates

N	1.122300	-0.020427	2.122135
C	1.581674	1.269753	2.084737
C	2.945931	1.480203	2.192545
C	3.237951	-0.939196	2.613393
C	3.839798	0.326535	2.228977
H	4.260618	0.140891	0.911055
H	0.126490	-0.203637	1.850075
C	3.611145	2.800331	2.291024
O	2.768714	3.852706	2.213676
O	4.815705	2.924763	2.438593

C	4.169519	-1.982109	3.084436
O	3.596107	-2.969324	3.794636
O	5.375168	-1.942142	2.868020
C	3.389455	5.149208	2.259541
H	3.984865	5.308185	1.357030
H	2.564529	5.861201	2.301850
H	4.026259	5.240846	3.142376
C	4.484095	-4.009019	4.244202
H	4.949441	-4.508694	3.390885
H	5.264146	-3.593547	4.886387
H	3.854846	-4.703907	4.800135
C	0.499603	2.307702	1.958831
H	0.477994	2.945548	2.847096
H	0.679386	2.964136	1.105521
H	-0.477391	1.834123	1.849730
C	1.090472	-2.377501	2.672227
H	0.100263	-2.289185	2.222278
H	1.606725	-3.239572	2.247960
H	0.968738	-2.571769	3.743371
P	-1.948920	-0.123625	-0.052761
O	-1.292397	-0.772450	1.142081
O	-1.120426	0.585548	-1.084779
O	-2.884370	-1.203457	-0.883390
O	-3.108391	0.866367	0.605783
H	4.823540	0.535675	2.641518
C	-4.232281	1.256521	-0.088340
C	-5.204966	0.315924	-0.410759
C	-5.539443	3.049799	-1.020337
C	-4.399386	2.651390	-0.350777
H	-5.697889	4.108010	-1.210823
C	-5.078117	-1.097874	0.049818
C	-3.934384	-1.828505	-0.242438
C	-3.824418	-3.228410	0.012737
C	-4.880984	-3.849888	0.647225
H	-4.831071	-4.919276	0.835272
C	-7.615199	2.552311	-2.264743
C	-8.517892	1.644381	-2.766531
C	-8.330633	0.263585	-2.521538
C	-7.269668	-0.179964	-1.763078
C	-6.327605	0.731927	-1.209614
C	-6.498760	2.123985	-1.496418
H	-7.732249	3.615791	-2.459737
H	-9.363922	1.980786	-3.359492
H	-9.028687	-0.456428	-2.940217
H	-7.139192	-1.242384	-1.594663
C	-8.221831	-1.681857	2.050388
C	-7.246334	-1.031476	1.327040
C	-6.123078	-1.733832	0.807988
C	-6.020487	-3.134145	1.090712
C	-7.053802	-3.779884	1.822382
C	-8.136152	-3.073150	2.291942
H	-9.064154	-1.118539	2.443057
H	-7.325601	0.036205	1.159179
H	-6.962839	-4.846309	2.015201
H	-8.916992	-3.575183	2.856601

C	1.873313	-1.110952	2.465551
C	3.841512	1.355587	-0.880693
C	4.370302	0.033481	-0.480302
C	3.506144	-1.105287	-0.836633
C	2.178732	-0.899965	-1.168979
C	2.494096	1.490456	-1.273119
H	0.664439	0.479879	-1.378526
N	1.692731	0.368800	-1.295840
C	1.974870	2.750004	-1.679860
C	2.845114	3.819100	-1.581100
C	4.601811	2.565278	-0.833050
H	2.501111	4.810629	-1.870985
C	-2.630085	-4.024134	-0.407656
C	-1.818470	-4.645077	0.574413
C	-2.354864	-4.219866	-1.783930
C	-2.032833	-4.479457	1.982393
C	-0.714771	-5.484522	0.162565
C	-1.229523	-5.035732	-2.179575
C	-3.165729	-3.662223	-2.823983
C	-1.255599	-5.130140	2.905527
H	-2.823350	-3.817781	2.315611
C	0.075579	-6.140395	1.158757
C	-0.443734	-5.647348	-1.198321
C	-0.953293	-5.220958	-3.570700
H	-4.029669	-3.066131	-2.554554
C	-2.872719	-3.869283	-4.146341
C	-0.190802	-5.981493	2.492035
H	-1.448207	-4.990289	3.966086
H	0.892288	-6.777595	0.827206
H	0.392579	-6.273511	-1.502635
C	-1.747440	-4.653111	-4.529526
H	-0.095635	-5.829316	-3.847660
H	-3.505294	-3.430954	-4.913465
H	0.409349	-6.496054	3.238025
H	-1.528157	-4.801846	-5.583379
C	-3.414060	3.685026	0.094827
C	-2.760051	4.489748	-0.873033
C	-3.206998	3.929058	1.475954
C	-2.906337	4.286992	-2.285212
C	-1.899526	5.569971	-0.442485
C	-2.313179	4.986963	1.890111
C	-3.875100	3.188720	2.503911
C	-2.302378	5.116366	-3.194153
H	-3.503382	3.453111	-2.634137
C	-1.286492	6.411416	-1.424140
C	-1.690399	5.780536	0.922496
C	-2.103983	5.220731	3.286014
H	-4.571917	2.408897	2.221365
C	-3.652789	3.447097	3.831235
C	-1.490296	6.203908	-2.761801
H	-2.435404	4.937773	-4.257738
H	-0.658396	7.229490	-1.078552
H	-1.039250	6.592610	1.240776
C	-2.749257	4.471740	4.232655
H	-1.425423	6.019432	3.577266

H	-4.173170	2.866139	4.587736
H	-1.027852	6.857530	-3.496721
H	-2.585219	4.661859	5.290000
C	5.856908	-0.213878	-0.717078
C	6.794991	-0.345808	0.305079
C	6.292295	-0.361002	-2.057180
C	8.133777	-0.626210	0.040650
H	6.476934	-0.250897	1.335438
C	7.629180	-0.649116	-2.337292
C	8.551412	-0.779755	-1.286237
H	8.845236	-0.731237	0.852296
H	7.969684	-0.770292	-3.357784
C	1.150017	-1.977417	-1.371824
H	0.425230	-1.947166	-0.549377
H	0.588841	-1.802629	-2.295150
H	1.603204	-2.963215	-1.423347
C	0.590323	2.922056	-2.244365
H	0.458373	2.323624	-3.155355
H	-0.197916	2.605575	-1.556436
H	0.419837	3.969594	-2.504170
C	9.923011	-1.074815	-1.583931
N	11.035797	-1.313953	-1.825734
N	4.121794	3.752786	-1.152346
O	5.875747	2.465517	-0.421398
O	5.343744	-0.201058	-3.016381
C	5.643232	-0.587176	-4.352452
H	4.711156	-0.471865	-4.906979
H	6.411708	0.058790	-4.795811
H	5.966126	-1.633931	-4.395732
C	6.640808	3.669126	-0.296865
H	6.649251	4.226641	-1.236868
H	6.233803	4.295316	0.500315
H	7.645973	3.334739	-0.036246
C	4.068968	-2.499793	-0.989813
N	4.802998	-3.007954	0.045629
H	5.313060	-3.856481	-0.165148
H	5.173252	-2.433847	0.792625
O	3.847145	-3.166606	-1.997320

S-forming TS Conformation 9

B3LYP/6-31G(d) Energy = -4490.487282
M06-2X/6-311G(d,p)-SMD(tetrahydrofuran) Energy = -4489.997910
M06-2X/6-311G(d,p)-SMD(tetrahydrofuran)-derived Free Energy (Quasiharmonic) = -4488.898531

Frequencies (Top 3 out of 468)

1. -1257.3114 cm^{-1}
2. 7.4943 cm^{-1}
3. 9.7969 cm^{-1}

B3LYP/6-31G(d) Molecular Geometry in Cartesian Coordinates

N	1.190492	-0.136033	1.947840

C	1.908209	-1.262403	2.248648
C	3.280709	-1.140820	2.386047
C	3.060686	1.300692	2.058402
C	3.922504	0.117093	2.015019
H	4.316964	-0.012404	0.718107
H	0.182656	-0.274596	1.702518
C	4.164158	-2.203771	2.914901
O	3.511412	-3.257689	3.446817
O	5.384414	-2.133572	2.910632
C	3.759057	2.595233	2.233156
O	2.938292	3.669641	2.229759
O	4.966349	2.687592	2.379725
C	4.347769	-4.302919	3.968233
H	4.986098	-3.920990	4.768865
H	3.660832	-5.058384	4.350131
H	4.977162	-4.720050	3.177918
C	3.583904	4.947122	2.356332
H	4.249861	4.959915	3.222070
H	4.153516	5.167622	1.449782
H	2.774944	5.668129	2.479553
C	1.071282	-2.497388	2.440386
H	1.524348	-3.366468	1.962515
H	0.986327	-2.730596	3.506987
H	0.068656	-2.341480	2.039432
C	0.632057	2.211324	1.918696
H	-0.357261	1.769262	1.787423
H	0.631325	2.787500	2.848592
H	0.821008	2.919840	1.110676
P	-1.986051	-0.104237	-0.118157
O	-1.299067	-0.775665	1.045747
O	-1.179940	0.592211	-1.177256
O	-2.976136	-1.153143	-0.923838
O	-3.095682	0.907252	0.590538
H	4.921356	0.271865	2.412390
C	-4.235199	1.331890	-0.056024
C	-5.240776	0.418517	-0.354951
C	-5.533340	3.167934	-0.914874
C	-4.380086	2.734177	-0.291500
H	-5.674701	4.232240	-1.083570
C	-5.133542	-1.003647	0.084576
C	-4.019656	-1.759343	-0.255106
C	-3.938011	-3.165279	-0.022630
C	-4.988840	-3.767853	0.639040
H	-4.961235	-4.840799	0.810603
C	-7.661181	2.735254	-2.093818
C	-8.601409	1.855219	-2.576007
C	-8.437958	0.467237	-2.355910
C	-7.362140	-0.010921	-1.640609
C	-6.380529	0.871400	-1.108421
C	-6.529192	2.270875	-1.370543
H	-7.760180	3.803766	-2.270453
H	-9.459271	2.219068	-3.134777
H	-9.166503	-0.230786	-2.759607
H	-7.250656	-1.078284	-1.490867
C	-8.220877	-1.533516	2.185761

C	-7.254031	-0.898693	1.437372
C	-6.168287	-1.622657	0.870439
C	-6.093096	-3.028969	1.131120
C	-7.117396	-3.657795	1.889688
C	-8.163834	-2.929803	2.405899
H	-9.033888	-0.953988	2.614939
H	-7.310759	0.172956	1.286469
H	-7.048086	-4.728821	2.065239
H	-8.937770	-3.419233	2.990775
C	1.688463	1.139420	1.966143
C	3.775568	1.286156	-1.024816
C	4.322205	-0.052270	-0.743098
C	3.423270	-1.173789	-1.041736
C	2.084814	-0.947633	-1.325085
C	2.420192	1.446821	-1.377338
H	0.583689	0.452224	-1.495296
N	1.614217	0.329888	-1.432698
C	1.907649	2.728733	-1.718607
C	2.790778	3.784633	-1.585673
C	4.547144	2.486335	-0.924153
H	2.451228	4.790293	-1.827508
C	-2.779686	-3.985126	-0.494163
C	-2.552345	-4.162622	-1.881702
C	-1.954744	-4.644887	0.450948
C	-3.381335	-3.567559	-2.886133
C	-1.459831	-4.997469	-2.326612
C	-0.885970	-5.503629	-0.010244
C	-2.121273	-4.501085	1.867801
C	-3.134037	-3.757128	-4.220418
H	-4.222400	-2.956826	-2.579647
C	-1.230411	-5.163006	-3.728686
C	-0.659886	-5.646347	-1.381504
C	-0.082956	-6.198825	0.948442
H	-2.883446	-3.825927	2.238003
C	-1.333644	-5.189455	2.754041
C	-2.039698	-4.559276	-4.652264
H	-3.779435	-3.290434	-4.959635
H	-0.396235	-5.785850	-4.042661
H	0.152100	-6.285263	-1.722684
C	-0.304743	-6.059682	2.292015
H	0.707033	-6.849047	0.579678
H	-1.489894	-5.065028	3.822481
H	-1.856184	-4.693321	-5.714881
H	0.304339	-6.603695	3.009334
C	-3.358329	3.739008	0.137666
C	-2.722413	4.549550	-0.837444
C	-3.097463	3.951770	1.514996
C	-2.921568	4.376890	-2.247188
C	-1.825142	5.603743	-0.417463
C	-2.167220	4.982652	1.916782
C	-3.744655	3.205781	2.552205
C	-2.332028	5.210922	-3.161366
H	-3.547366	3.561885	-2.590328
C	-1.229509	6.451691	-1.404318
C	-1.563017	5.782754	0.942813

C	-1.903388	5.184419	3.308391
H	-4.467950	2.446549	2.280465
C	-3.469947	3.433448	3.875312
C	-1.483406	6.273714	-2.737731
H	-2.505094	5.055115	-4.222760
H	-0.573461	7.250597	-1.065824
H	-0.883855	6.574785	1.252910
C	-2.530622	4.430675	4.263319
H	-1.197800	5.962911	3.589980
H	-3.975735	2.848861	4.638872
H	-1.033166	6.931785	-3.476241
H	-2.325073	4.596503	5.317503
C	5.753832	-0.256077	-1.257041
C	5.893481	-0.269879	-2.648372
C	6.915277	-0.416172	-0.472105
C	7.131894	-0.408658	-3.271141
H	5.003841	-0.164123	-3.261183
C	8.165802	-0.546507	-1.077987
C	8.275989	-0.538569	-2.478549
H	7.209936	-0.412683	-4.352627
H	9.061748	-0.657045	-0.481104
C	1.029666	-2.003795	-1.499527
H	0.337539	-1.967737	-0.649552
H	0.435980	-1.802792	-2.396987
H	1.466416	-2.994243	-1.581343
C	0.517073	2.942040	-2.252335
H	-0.262634	2.615855	-1.559779
H	0.360872	3.999880	-2.476435
H	0.358327	2.374447	-3.178466
C	9.569408	-0.670724	-3.083671
N	10.621067	-0.776061	-3.570183
N	4.073339	3.690917	-1.178024
O	5.822384	2.349957	-0.530002
O	6.733705	-0.462275	0.871326
C	7.865000	-0.604067	1.726084
H	8.536989	0.256914	1.629672
H	7.458463	-0.658382	2.733837
H	8.414020	-1.528212	1.507837
C	6.615610	3.533590	-0.386460
H	6.586750	4.136200	-1.297411
H	6.261489	4.123666	0.461558
H	7.626815	3.171538	-0.195145
C	3.967434	-2.580050	-1.085172
N	4.877983	-2.911795	-0.120921
H	5.344120	-3.800646	-0.242672
H	5.286155	-2.257546	0.535046
O	3.611119	-3.400960	-1.926390

S-forming TS Conformation 10

B3LYP/6-31G(d) Energy = -4490.485362
M06-2X/6-311G(d,p)-SMD(tetrahydrofuran) Energy = -4489.995729
M06-2X/6-311G(d,p)-SMD(tetrahydrofuran)-derived Free Energy (Quasiharmonic) = -4488.898322

Frequencies (Top 3 out of 468)

1. -1200.8904 cm^{-1}
2. 7.2440 cm^{-1}
3. 10.2624 cm^{-1}

B3LYP/6-31G(d) Molecular Geometry in Cartesian Coordinates

N	1.108525	0.233674	2.098983
C	1.549755	1.525451	1.965106
C	2.908854	1.767648	2.067050
C	3.236152	-0.622601	2.643636
C	3.810189	0.625762	2.170879
H	4.188313	0.330402	0.848768
H	0.109494	0.020946	1.865028
C	3.459994	3.145840	2.105249
O	4.811928	3.125555	2.203626
O	2.828316	4.186598	2.086221
C	4.203576	-1.577463	3.228778
O	3.701772	-2.818676	3.449334
O	5.362358	-1.293195	3.477188
C	5.432848	4.416810	2.293377
H	5.170844	5.025974	1.425142
H	5.109970	4.933840	3.201240
H	6.504861	4.221139	2.324196
C	4.624388	-3.756400	4.036972
H	4.059349	-4.681431	4.153027
H	5.486069	-3.905287	3.381660
H	4.972144	-3.391698	5.006172
C	0.455167	2.523846	1.730523
H	0.856728	3.505024	1.497790
H	-0.186717	2.175116	0.913688
H	-0.176101	2.607851	2.623945
C	1.084393	-2.056972	2.876467
H	0.028589	-1.914185	2.645210
H	1.438843	-2.934882	2.332226
H	1.196891	-2.284375	3.941119
P	-1.993764	-0.058228	0.049560
O	-1.310316	-0.686599	1.240073
O	-1.192192	0.732843	-0.946440
O	-3.235528	0.829922	0.689400
O	-2.828347	-1.188461	-0.826883
H	4.804892	0.843236	2.545539
C	-3.871672	-1.872162	-0.234381
C	-5.065618	-1.207185	0.014897
C	-4.742326	-3.946702	0.613868
C	-3.698475	-3.265361	0.021071
H	-4.640430	-5.012486	0.801604
C	-5.248427	0.203486	-0.435504
C	-4.338783	1.183740	-0.057118
C	-4.550427	2.572246	-0.317548
C	-5.676743	2.926743	-1.033351
H	-5.871663	3.978630	-1.225008
C	-6.961072	-4.001714	1.700475
C	-8.098853	-3.357281	2.126317

C	-8.251148	-1.971882	1.883234
C	-7.284550	-1.266061	1.201263
C	-6.104142	-1.903787	0.727109
C	-5.936394	-3.297160	1.012190
H	-6.819235	-5.062175	1.895434
H	-8.873138	-3.903403	2.658092
H	-9.138361	-1.457157	2.242227
H	-7.415408	-0.203699	1.032558
C	-8.277475	0.036097	-2.676400
C	-7.230196	-0.368921	-1.878341
C	-6.353750	0.577341	-1.277263
C	-6.573882	1.964241	-1.557011
C	-7.675164	2.351207	-2.368009
C	-8.514929	1.410093	-2.916021
H	-8.925416	-0.708908	-3.130444
H	-7.060054	-1.426417	-1.713589
H	-7.831314	3.410567	-2.557864
H	-9.349561	1.715836	-3.540881
C	1.869713	-0.820199	2.525084
C	3.808590	1.409456	-1.017423
C	4.292469	0.108321	-0.511066
C	3.380889	-1.015746	-0.780436
C	2.062557	-0.795500	-1.104696
C	2.450313	1.568315	-1.371100
H	0.582447	0.615372	-1.357064
N	1.607202	0.479257	-1.293226
C	1.964702	2.812812	-1.856674
C	2.888922	3.838524	-1.917387
C	4.624107	2.573351	-1.140910
H	2.575688	4.815734	-2.280349
C	-3.616998	3.633806	0.170393
C	-3.463827	3.868100	1.559820
C	-2.946339	4.460434	-0.767127
C	-4.158379	3.109668	2.556370
C	-2.597700	4.931814	2.014977
C	-2.116764	5.547836	-0.295919
C	-3.043901	4.273952	-2.185681
C	-3.983673	3.355950	3.892935
H	-4.837580	2.326234	2.242002
C	-2.435131	5.150557	3.419438
C	-1.952933	5.743831	1.077555
C	-1.484990	6.410782	-1.246253
H	-3.615925	3.435821	-2.564983
C	-2.426236	5.126112	-3.063920
C	-3.103075	4.383872	4.335572
H	-4.523433	2.761824	4.625375
H	-1.770831	5.949173	3.741245
H	-1.317986	6.555842	1.426176
C	-1.644549	6.219521	-2.592169
H	-0.877409	7.230376	-0.869617
H	-2.524763	4.960981	-4.133553
H	-2.973905	4.561732	5.399846
H	-1.169631	6.890207	-3.303446
C	-2.455603	-4.004959	-0.361240
C	-1.638023	-4.572469	0.647189

C	-2.147554	-4.216636	-1.728016
C	-1.881381	-4.377343	2.046476
C	-0.501180	-5.384884	0.273184
C	-0.993168	-5.008958	-2.087068
C	-2.952260	-3.697825	-2.792712
C	-1.095940	-4.974035	2.998555
H	-2.700506	-3.735553	2.348097
C	0.295003	-5.986576	1.298847
C	-0.206594	-5.575588	-1.079310
C	-0.688873	-5.215796	-3.469282
H	-3.834218	-3.117096	-2.549506
C	-2.629102	-3.921289	-4.105431
C	0.003593	-5.799001	2.623384
H	-1.309313	-4.812932	4.051965
H	1.134331	-6.609528	0.997254
H	0.648897	-6.188795	-1.356248
C	-1.479283	-4.686039	-4.452818
H	0.187296	-5.809157	-3.719950
H	-3.256629	-3.511792	-4.892283
H	0.607790	-6.274035	3.392133
H	-1.237913	-4.850462	-5.499452
C	5.760131	-0.256560	-0.686105
C	6.598742	-0.570462	0.381067
C	6.259550	-0.394831	-2.004305
C	7.911490	-0.996858	0.179720
H	6.231032	-0.502753	1.397937
C	7.566757	-0.827829	-2.220462
C	8.396099	-1.124812	-1.125574
H	8.547210	-1.236176	1.025063
H	7.953944	-0.941961	-3.225142
C	1.033164	-1.877209	-1.273884
H	1.495688	-2.858277	-1.381208
H	0.363751	-1.901269	-0.405299
H	0.408785	-1.686524	-2.151566
C	0.556247	3.001311	-2.352980
H	0.356215	2.366066	-3.226209
H	-0.203245	2.746552	-1.609182
H	0.399639	4.040542	-2.651854
C	9.741409	-1.564819	-1.357472
N	10.833822	-1.919134	-1.545046
N	4.188047	3.740466	-1.570097
O	5.921092	2.453660	-0.791215
O	5.402461	-0.075953	-3.007039
C	5.652345	-0.601272	-4.306667
H	5.776781	-1.688343	-4.263127
H	4.768221	-0.356447	-4.897470
H	6.531739	-0.135820	-4.769891
C	6.787719	3.551299	-1.092714
H	6.775640	3.772222	-2.164073
H	6.492640	4.451481	-0.547995
H	7.780147	3.221416	-0.781471
C	3.928012	-2.427767	-0.901470
N	3.903875	-3.193045	0.224651
H	3.492750	-2.888530	1.093532
H	4.243250	-4.143176	0.153925

O	4.360187	-2.854455	-1.965467

S-forming TS Conformation 11

B3LYP/6-31G(d) Energy = -4490.493918
M06-2X/6-311G(d,p)-SMD(tetrahydrofuran) Energy = -4490.001407
M06-2X/6-311G(d,p)-SMD(tetrahydrofuran)-derived Free Energy (Quasiharmonic) = -4488.902519

Frequencies (Top 3 out of 468)

1. -1179.3310 cm^{-1}
2. 7.3585 cm^{-1}
3. 10.1293 cm^{-1}

B3LYP/6-31G(d) Molecular Geometry in Cartesian Coordinates

N	1.028500	-1.831546	0.932639
C	1.386969	-2.360684	-0.275093
C	2.728604	-2.631195	-0.493093
C	3.196922	-1.996089	1.855056
C	3.702525	-2.251861	0.518456
H	4.214905	-1.022959	0.074936
H	0.043842	-1.495587	1.048557
C	3.293061	-3.274588	-1.692166
O	2.391660	-3.725426	-2.583132
O	4.501949	-3.395237	-1.865988
C	4.215678	-2.057778	2.930565
O	3.711433	-1.981169	4.181389
O	5.405170	-2.200950	2.706410
C	2.937423	-4.317162	-3.776308
H	2.073050	-4.631485	-4.361175
H	3.528329	-3.581013	-4.326857
H	3.568956	-5.172704	-3.525680
C	4.677579	-2.081737	5.240852
H	5.401946	-1.265838	5.180101
H	4.101972	-2.015549	6.164346
H	5.209807	-3.034654	5.183843
C	0.252359	-2.606280	-1.227631
H	0.194662	-3.667468	-1.478388
H	-0.696951	-2.304027	-0.788967
H	0.399797	-2.061173	-2.162879
C	1.145363	-1.349083	3.300713
H	0.144575	-0.972743	3.078436
H	1.050283	-2.231051	3.943421
H	1.710050	-0.601166	3.857471
P	-1.984441	0.118750	0.102516
O	-1.424945	-0.623442	1.293743
O	-1.055831	0.699728	-0.926617
O	-3.009644	1.268785	0.714982
O	-3.054859	-0.860664	-0.713900
H	4.646426	-2.786932	0.473790
C	-4.207005	-1.253880	-0.056599
C	-5.224867	-0.326783	0.128506
C	-5.473988	-2.985775	1.028600

C	-4.328921	-2.613717	0.353498
H	-5.590359	-4.020457	1.341095
C	-5.082541	1.040625	-0.447683
C	-3.975691	1.811003	-0.114144
C	-3.828045	3.163341	-0.540463
C	-4.798233	3.687300	-1.371068
H	-4.709499	4.719695	-1.699585
C	-7.635292	-2.450094	2.103418
C	-8.608718	-1.537855	2.437280
C	-8.476528	-0.189788	2.027602
C	-7.393898	0.215518	1.278891
C	-6.377149	-0.704892	0.898859
C	-6.498149	-2.060045	1.345360
H	-7.713763	-3.486249	2.424161
H	-9.471334	-1.844734	3.022323
H	-9.235091	0.535008	2.310876
H	-7.304375	1.253811	0.981361
C	-8.017417	1.353548	-2.791793
C	-7.123919	0.804895	-1.898648
C	-6.044566	1.570214	-1.374340
C	-5.896876	2.919890	-1.829230
C	-6.844473	3.461290	-2.739675
C	-7.887159	2.698719	-3.211286
H	-8.828635	0.744800	-3.182189
H	-7.234799	-0.229671	-1.595465
H	-6.720134	4.491477	-3.065016
H	-8.602963	3.120241	-3.911641
C	1.851684	-1.722443	2.025362
C	3.795396	1.014801	0.783194
C	4.425040	0.278071	-0.340992
C	3.683331	0.374961	-1.606932
C	2.347334	0.719958	-1.610462
C	2.427585	1.360751	0.713374
H	0.701098	1.166409	-0.473466
N	1.730597	1.078653	-0.445721
C	1.785271	2.019599	1.795150
C	2.583431	2.300553	2.889798
C	4.458104	1.348283	2.001276
H	2.151235	2.826339	3.739930
C	-2.699690	4.043782	-0.100787
C	-1.692817	4.410275	-1.025509
C	-2.705706	4.594819	1.202737
C	-1.612383	3.852947	-2.343191
C	-0.689487	5.379224	-0.641884
C	-1.687741	5.549517	1.579133
C	-3.699604	4.261513	2.178168
C	-0.637503	4.245848	-3.222749
H	-2.329600	3.094426	-2.632794
C	0.308725	5.762376	-1.592529
C	-0.716013	5.922607	0.645674
C	-1.704712	6.102170	2.898346
H	-4.477835	3.554957	1.912372
C	-3.680146	4.812621	3.432616
C	0.333485	5.219347	-2.848584
H	-0.598709	3.807108	-4.216222

H	1.050054	6.498750	-1.290736
H	0.038954	6.653596	0.928830
C	-2.668689	5.744666	3.802247
H	-0.931347	6.818690	3.165795
H	-4.443684	4.537672	4.155260
H	1.095508	5.520675	-3.562417
H	-2.670125	6.172252	4.801323
C	-3.302280	-3.658769	0.041722
C	-2.482683	-4.174384	1.073866
C	-3.239049	-4.209928	-1.260848
C	-2.477118	-3.629964	2.399441
C	-1.605549	-5.291476	0.799655
C	-2.349215	-5.318097	-1.525823
C	-4.043807	-3.728295	-2.342981
C	-1.689998	-4.167914	3.384254
H	-3.094062	-2.764864	2.609568
C	-0.802397	-5.822594	1.857811
C	-1.565442	-5.833728	-0.488342
C	-2.302431	-5.874402	-2.842841
H	-4.722633	-2.902385	-2.162967
C	-3.966964	-4.287142	-3.591771
C	-0.846043	-5.284212	3.115603
H	-1.705826	-3.737028	4.381832
H	-0.156604	-6.669832	1.638420
H	-0.912603	-6.681736	-0.687271
C	-3.084800	-5.375242	-3.848983
H	-1.630375	-6.710143	-3.024654
H	-4.585603	-3.898820	-4.396197
H	-0.235629	-5.701931	3.912036
H	-3.040727	-5.807928	-4.844882
C	5.937174	0.371792	-0.499576
C	6.807167	-0.679344	-0.215324
C	6.474827	1.576955	-1.012506
C	8.175164	-0.581787	-0.459295
H	6.419239	-1.587688	0.227840
C	7.840487	1.683119	-1.279054
C	8.691258	0.599936	-1.004404
H	8.835570	-1.412186	-0.234964
H	8.256816	2.595143	-1.688231
C	1.485274	0.792639	-2.841993
H	1.535843	1.804536	-3.258934
H	1.832590	0.104071	-3.613554
H	0.443519	0.583533	-2.586847
C	0.332177	2.409292	1.779798
H	0.044154	2.904311	0.847741
H	-0.321724	1.539883	1.905213
H	0.120831	3.103293	2.598048
C	10.093982	0.717099	-1.279396
N	11.232087	0.812736	-1.502216
N	3.883839	1.977384	3.010868
O	5.750420	0.990090	2.119730
O	5.591631	2.590197	-1.206832
C	5.952078	3.660960	-2.073213
H	6.257001	3.279786	-3.053726
H	5.049474	4.262759	-2.184973

H	6.748859	4.279113	-1.640332
C	6.433092	1.352421	3.322176
H	7.439330	0.947858	3.206762
H	6.463964	2.439393	3.441189
H	5.942277	0.919423	4.198203
C	4.378980	0.278425	-2.949042
N	5.066439	-0.860181	-3.222093
H	5.131647	-1.650660	-2.590438
H	5.608195	-0.876180	-4.075584
O	4.307476	1.205003	-3.754172

S-forming TS Conformation 12

B3LYP/6-31G(d) Energy = -4490.492345
M06-2X/6-311G(d,p)-SMD(tetrahydrofuran) Energy = -4490.000483
M06-2X/6-311G(d,p)-SMD(tetrahydrofuran)-derived Free Energy (Quasiharmonic) = -4488.901850

Frequencies (Top 3 out of 468)

1. -1157.5223 cm^{-1}
2. 7.2685 cm^{-1}
3. 9.3143 cm^{-1}

B3LYP/6-31G(d) Molecular Geometry in Cartesian Coordinates

N	1.075292	-1.754148	1.054214
C	1.530589	-2.427161	-0.052810
C	2.885738	-2.682218	-0.144915
C	3.192483	-1.656189	2.091548
C	3.782324	-2.083995	0.831186
H	4.224565	-0.960376	0.190072
H	0.075164	-1.464265	1.084900
C	3.559845	-3.493235	-1.189213
O	2.731611	-4.047278	-2.090959
O	4.767716	-3.667018	-1.197913
C	4.145689	-1.483448	3.212544
O	3.557652	-1.332319	4.422009
O	5.355207	-1.507093	3.066209
C	3.383253	-4.734196	-3.174941
H	2.574838	-5.118234	-3.798123
H	4.004014	-4.029370	-3.730903
H	3.999618	-5.552620	-2.794622
C	4.456619	-1.179725	5.531244
H	3.823960	-1.181749	6.419460
H	5.173185	-2.003827	5.562860
H	5.000606	-0.234062	5.456175
C	0.466323	-2.834115	-1.031977
H	0.343278	-3.920578	-1.029521
H	-0.493603	-2.383397	-0.776137
H	0.744257	-2.548213	-2.047386
C	1.028489	-0.986691	3.349526
H	0.016455	-0.718214	3.041199
H	0.967370	-1.788109	4.093781
H	1.510141	-0.136167	3.831912

P	-2.009105	0.125491	0.086314
O	-1.436426	-0.570706	1.295660
O	-1.094744	0.703197	-0.958565
O	-3.054602	1.279575	0.660553
O	-3.061492	-0.892750	-0.704562
H	4.755653	-2.558476	0.923773
C	-4.214571	-1.276344	-0.043167
C	-5.243957	-0.354357	0.102518
C	-5.472956	-2.981551	1.092527
C	-4.326176	-2.622256	0.412991
H	-5.580979	-4.005800	1.440294
C	-5.109996	0.994275	-0.517847
C	-4.013768	1.785252	-0.198007
C	-3.870483	3.121423	-0.673677
C	-4.835314	3.607701	-1.533209
H	-4.749987	4.627758	-1.899151
C	-7.648481	-2.432044	2.131326
C	-8.634279	-1.519253	2.424959
C	-8.512928	-0.185085	1.968908
C	-7.428325	0.205281	1.215136
C	-6.398779	-0.716972	0.876001
C	-6.509261	-2.056679	1.368945
H	-7.718605	-3.457052	2.487820
H	-9.498475	-1.814669	3.013587
H	-9.281525	0.540966	2.220228
H	-7.347224	1.233436	0.881986
C	-8.022736	1.199767	-2.900573
C	-7.134049	0.690328	-1.979885
C	-6.066161	1.482630	-1.472398
C	-5.924121	2.816408	-1.973664
C	-6.866702	3.317549	-2.911993
C	-7.898710	2.530328	-3.366230
H	-8.825250	0.571046	-3.277043
H	-7.239635	-0.333672	-1.641076
H	-6.747164	4.336568	-3.272518
H	-8.610808	2.920974	-4.087966
C	1.824673	-1.459906	2.162506
C	3.827018	1.154819	0.583050
C	4.399600	0.281154	-0.463598
C	3.572686	0.173580	-1.664721
C	2.238680	0.508803	-1.648562
C	2.447499	1.462550	0.555819
H	0.657846	1.153484	-0.520809
N	1.687238	1.049396	-0.521596
C	1.848380	2.202635	1.610581
C	2.694482	2.593804	2.632702
C	4.546615	1.617666	1.723370
H	2.291475	3.173279	3.462041
C	-2.752658	4.027065	-0.258882
C	-1.744719	4.370842	-1.191292
C	-2.772714	4.627947	1.022339
C	-1.645052	3.758158	-2.482948
C	-0.760913	5.372538	-0.843144
C	-1.771726	5.612719	1.365489
C	-3.766232	4.317128	2.005501

C	-0.673108	4.132282	-3.374019
H	-2.344038	2.972110	-2.741618
C	0.233758	5.735822	-1.805390
C	-0.803019	5.966592	0.421388
C	-1.803833	6.215847	2.662103
H	-4.531527	3.588193	1.763979
C	-3.761333	4.916550	3.237689
C	0.275019	5.141488	-3.037543
H	-0.621356	3.652716	-4.348004
H	0.957687	6.500366	-1.532383
H	-0.063305	6.722675	0.677852
C	-2.766460	5.878217	3.574977
H	-1.043390	6.954670	2.904852
H	-4.523640	4.657477	3.967404
H	1.032503	5.430987	-3.761402
H	-2.779452	6.344045	4.556719
C	-3.291205	-3.671877	0.146911
C	-2.483924	-4.151213	1.205793
C	-3.216376	-4.270374	-1.133905
C	-2.484088	-3.553086	2.508015
C	-1.614027	-5.285366	0.984366
C	-2.331601	-5.393533	-1.347103
C	-4.006016	-3.824611	-2.242245
C	-1.707427	-4.055364	3.519572
H	-3.094747	-2.674613	2.677369
C	-0.824051	-5.779671	2.069881
C	-1.565819	-5.877584	-0.281320
C	-2.272225	-5.997580	-2.642204
H	-4.681795	-2.988685	-2.101013
C	-3.917706	-4.429294	-3.468696
C	-0.871522	-5.188892	3.303711
H	-1.724783	-3.582121	4.497768
H	-0.182717	-6.639499	1.890110
H	-0.918454	-6.738029	-0.440668
C	-3.038920	-5.530747	-3.675471
H	-1.601667	-6.841801	-2.785768
H	-4.524181	-4.066988	-4.294318
H	-0.268595	-5.576776	4.120605
H	-2.984069	-5.998665	-4.654734
C	5.897097	0.283749	-0.735579
C	6.643042	-0.894066	-0.763064
C	6.551914	1.496438	-1.059138
C	8.013023	-0.886949	-1.029131
H	6.151095	-1.845121	-0.597006
C	7.920837	1.525101	-1.306281
C	8.657772	0.326562	-1.280329
H	8.573512	-1.815114	-1.036065
H	8.430681	2.452888	-1.533512
C	1.329920	0.402616	-2.842864
H	0.320350	0.126305	-2.528946
H	1.250000	1.382200	-3.331814
H	1.712269	-0.320411	-3.563714
C	0.382148	2.540074	1.639833
H	0.174748	3.250280	2.445068
H	0.042304	2.995610	0.704733

H	-0.236640	1.652482	1.812053
C	10.068103	0.365505	-1.537067
N	11.211806	0.406003	-1.747447
N	4.010399	2.316216	2.707437
O	5.856645	1.314107	1.780379
O	5.750665	2.602329	-1.156408
C	6.363615	3.884758	-1.239570
H	6.912106	4.011859	-2.181791
H	5.546322	4.606364	-1.197781
H	7.044963	4.051424	-0.397007
C	6.582739	1.700107	2.950319
H	7.607788	1.377432	2.763721
H	6.536038	2.781941	3.102887
H	6.185286	1.194930	3.833727
C	4.162027	-0.326107	-2.967940
N	4.766117	0.648213	-3.707551
H	5.291209	0.364804	-4.525055
H	4.967262	1.547985	-3.292950
O	4.052547	-1.481624	-3.360257

S-forming TS Conformation 13

B3LYP/6-31G(d) Energy = -4490.487691
M06-2X/6-311G(d,p)-SMD(tetrahydrofuran) Energy = -4489.997039
M06-2X/6-311G(d,p)-SMD(tetrahydrofuran)-derived Free Energy (Quasiharmonic) = -4488.899133

Frequencies (Top 3 out of 468)

1. -1215.6005 cm^{-1}
2. 7.1209 cm^{-1}
3. 9.4636 cm^{-1}

B3LYP/6-31G(d) Molecular Geometry in Cartesian Coordinates

N	1.100579	0.057820	2.072343
C	1.870270	-1.021354	2.408033
C	3.233662	-0.828076	2.560490
C	2.904766	1.585295	2.135130
C	3.815186	0.446263	2.177239
H	4.199709	0.218445	0.843407
H	0.099829	-0.139397	1.822774
C	4.096401	-1.919412	3.053781
O	5.282033	-1.463669	3.518763
O	3.813501	-3.109585	3.047474
C	3.422687	2.966737	2.232895
O	4.774384	2.984115	2.353357
O	2.753078	3.983979	2.231715
C	6.189682	-2.474228	3.995794
H	7.069419	-1.931428	4.341336
H	5.736624	-3.036770	4.815336
H	6.451436	-3.163195	3.188820
C	5.351816	4.291980	2.482685
H	6.429603	4.131591	2.521095
H	5.079922	4.914427	1.626919

H	5.002755	4.775916	3.399174
C	1.113319	-2.298448	2.648324
H	1.406607	-3.077290	1.939859
H	1.339057	-2.694722	3.642261
H	0.040569	-2.132029	2.549846
C	0.465131	2.390355	1.916280
H	0.667651	3.078123	1.093154
H	-0.510670	1.922345	1.775916
H	0.434067	3.005308	2.820822
P	-2.000814	-0.114556	-0.031602
O	-1.318631	-0.739800	1.162105
O	-1.193685	0.588270	-1.085104
O	-2.932111	-1.218363	-0.836195
O	-3.163523	0.868168	0.627832
H	4.803288	0.662002	2.568974
C	-4.294193	1.244761	-0.063729
C	-5.261686	0.293755	-0.369798
C	-5.618438	3.018145	-1.007468
C	-4.471936	2.635669	-0.339693
H	-5.785549	4.073056	-1.208479
C	-5.122044	-1.112827	0.108683
C	-3.974519	-1.839447	-0.179124
C	-3.853049	-3.234031	0.097607
C	-4.900227	-3.853722	0.749014
H	-4.840293	-4.919408	0.954443
C	-7.696786	2.491924	-2.235986
C	-8.595948	1.572189	-2.722518
C	-8.398365	0.195620	-2.462417
C	-7.330449	-0.231933	-1.704474
C	-6.391601	0.692632	-1.166952
C	-6.573494	2.080209	-1.468602
H	-7.822068	3.552252	-2.442654
H	-9.447247	1.896096	-3.314918
H	-9.093907	-0.533808	-2.868840
H	-7.191986	-1.291442	-1.524140
C	-8.249646	-1.689250	2.136451
C	-7.283415	-1.043073	1.397131
C	-6.157709	-1.745033	0.882725
C	-6.042748	-3.139790	1.187419
C	-7.066723	-3.781645	1.935456
C	-8.151829	-3.075784	2.399840
H	-9.093976	-1.126117	2.525033
H	-7.371858	0.021133	1.212881
H	-6.966268	-4.844068	2.144942
H	-8.925483	-3.574597	2.977103
C	1.543496	1.351994	2.032416
C	3.796799	1.355131	-0.977776
C	4.301938	0.042594	-0.527422
C	3.408430	-1.086826	-0.832210
C	2.082841	-0.870998	-1.137923
C	2.434593	1.506729	-1.316765
H	0.579466	0.524288	-1.350626
N	1.607974	0.403076	-1.273378
C	1.929363	2.762950	-1.750921
C	2.838213	3.803879	-1.774586

C	4.593380	2.535913	-1.052145
H	2.508919	4.789346	-2.098700
C	-2.656605	-4.029086	-0.318506
C	-1.827971	-4.618587	0.668404
C	-2.397969	-4.257929	-1.692768
C	-2.024008	-4.414018	2.073770
C	-0.726067	-5.464229	0.264369
C	-1.276496	-5.082333	-2.082142
C	-3.222443	-3.726316	-2.735777
C	-1.228112	-5.031474	3.003751
H	-2.814826	-3.748461	2.398449
C	0.083258	-6.085307	1.267760
C	-0.475462	-5.665192	-1.095518
C	-1.020356	-5.304571	-3.471763
H	-4.082259	-3.122802	-2.469709
C	-2.946931	-3.966604	-4.056291
C	-0.163002	-5.886901	2.599592
H	-1.405142	-4.861457	4.062541
H	0.898940	-6.727942	0.944106
H	0.355659	-6.301192	-1.394391
C	-1.827552	-4.761453	-4.434100
H	-0.167995	-5.922195	-3.745153
H	-3.589103	-3.547139	-4.825920
H	0.454980	-6.371164	3.350922
H	-1.623270	-4.938504	-5.486599
C	-3.489718	3.678543	0.090296
C	-2.844617	4.477532	-0.888168
C	-3.273312	3.932909	1.468113
C	-3.002129	4.266556	-2.297980
C	-1.979950	5.559749	-0.471150
C	-2.373082	4.990713	1.867956
C	-3.936194	3.202483	2.506480
C	-2.406373	5.091449	-3.216409
H	-3.601465	3.430451	-2.637655
C	-1.375181	6.395885	-1.462407
C	-1.756458	5.776922	0.890505
C	-2.149407	5.232047	3.260321
H	-4.638870	2.423777	2.235193
C	-3.702212	3.469599	3.830059
C	-1.591002	6.181701	-2.797106
H	-2.548561	4.906973	-4.277885
H	-0.742233	7.213882	-1.126028
H	-1.095675	6.584775	1.198296
C	-2.790035	4.492141	4.217174
H	-1.461566	6.026747	3.539582
H	-4.219364	2.896391	4.594759
H	-1.134862	6.831272	-3.539561
H	-2.614889	4.687330	5.271808
C	5.774756	-0.287503	-0.719802
C	6.620085	-0.623611	0.334832
C	6.278359	-0.364636	-2.041339
C	7.940114	-1.019595	0.121705
H	6.247139	-0.585656	1.351230
C	7.592707	-0.770294	-2.270957
C	8.426236	-1.094201	-1.187484

H	8.584070	-1.273921	0.956549
H	7.982293	-0.840459	-3.278743
C	1.066441	-1.958567	-1.347727
H	0.362530	-1.981864	-0.506961
H	0.476627	-1.770414	-2.249827
H	1.536633	-2.938109	-1.431417
C	0.514262	2.953127	-2.225728
H	0.298872	2.321812	-3.097424
H	-0.231094	2.689118	-1.470989
H	0.352674	3.993829	-2.516146
C	9.779226	-1.502835	-1.431227
N	10.878615	-1.831450	-1.624468
N	4.139678	3.712420	-1.433052
O	5.892399	2.422503	-0.703940
O	5.421411	-0.013332	-3.030618
C	5.669446	-0.494460	-4.348496
H	6.546121	-0.010590	-4.797619
H	5.795355	-1.581832	-4.340115
H	4.782940	-0.232107	-4.927866
C	6.739034	3.548570	-0.951864
H	6.725118	3.819104	-2.011729
H	6.426070	4.416907	-0.366772
H	7.737015	3.223314	-0.653451
C	3.982024	-2.478519	-1.056726
N	3.970993	-3.342528	-0.009047
H	4.365729	-4.260890	-0.165159
H	3.637002	-3.125994	0.920475
O	4.417431	-2.800209	-2.157700

S-forming TS Conformation 14

B3LYP/6-31G(d) Energy = -4490.491956
M06-2X/6-311G(d,p)-SMD(tetrahydrofuran) Energy = -4490.002181
M06-2X/6-311G(d,p)-SMD(tetrahydrofuran)-derived Free Energy (Quasiharmonic) = -4488.902651

Frequencies (Top 3 out of 468)

1. -1204.6634 cm^{-1}
2. 7.5105 cm^{-1}
3. 12.0645 cm^{-1}

B3LYP/6-31G(d) Molecular Geometry in Cartesian Coordinates

N	1.171704	-1.594039	0.794051
C	1.550584	-2.118427	-0.414153
C	2.896486	-2.378084	-0.615555
C	3.340260	-1.718849	1.725727
C	3.862559	-1.920832	0.381973
H	4.274782	-0.695076	-0.048860
H	0.174297	-1.311740	0.921741
C	3.474454	-3.113390	-1.758178
O	2.591574	-3.807129	-2.502735
O	4.677878	-3.140638	-1.994080
C	4.249509	-1.786987	2.894112

O	5.473423	-2.258098	2.545232
O	3.965383	-1.504609	4.044680
C	3.147069	-4.528369	-3.616220
H	2.295915	-5.006106	-4.101397
H	3.649724	-3.844405	-4.304654
H	3.863534	-5.276227	-3.267583
C	6.393781	-2.431772	3.631174
H	5.981307	-3.112562	4.380620
H	7.294649	-2.854337	3.184586
H	6.616924	-1.474747	4.108774
C	0.421855	-2.334550	-1.378469
H	-0.173226	-3.203472	-1.076894
H	-0.234716	-1.459678	-1.378102
H	0.784497	-2.507390	-2.387181
C	1.283320	-1.187900	3.187587
H	0.242235	-0.918597	2.999363
H	1.309715	-2.068815	3.838354
H	1.793811	-0.389421	3.726965
P	-2.041443	0.113646	0.087543
O	-1.379603	-0.547337	1.271661
O	-1.188825	0.715016	-0.998613
O	-3.081256	-0.942753	-0.663648
O	-3.108466	1.225211	0.700440
H	4.837019	-2.389196	0.322197
C	-4.140909	1.679137	-0.101278
C	-5.212197	0.833813	-0.362648
C	-5.129558	3.462630	-1.374410
C	-4.094641	3.023535	-0.573513
H	-5.117737	4.487537	-1.736456
C	-5.245395	-0.522822	0.254068
C	-4.179473	-1.389239	0.049032
C	-4.196582	-2.742804	0.494901
C	-5.288192	-3.169826	1.223581
H	-5.325641	-4.201339	1.564685
C	-7.215300	3.072381	-2.642462
C	-8.228520	2.234467	-3.045401
C	-8.257728	0.897487	-2.582833
C	-7.294954	0.431401	-1.715136
C	-6.243434	1.276065	-1.260579
C	-6.198294	2.617219	-1.760156
H	-7.168171	4.097562	-3.002152
H	-8.998087	2.590395	-3.724847
H	-9.045698	0.229113	-2.919660
H	-7.328964	-0.598263	-1.378881
C	-8.420200	-0.542661	2.283613
C	-7.396319	-0.089050	1.481792
C	-6.339464	-0.953187	1.079983
C	-6.356099	-2.302125	1.560690
C	-7.435098	-2.743578	2.373524
C	-8.450201	-1.885961	2.727591
H	-9.211025	0.140151	2.582544
H	-7.384548	0.945321	1.158339
H	-7.434743	-3.774365	2.720129
H	-9.267367	-2.231709	3.354696
C	1.985893	-1.491938	1.894135

C	3.617232	1.294653	0.687162
C	4.318811	0.677675	-0.462043
C	3.567189	0.702528	-1.718109
C	2.207710	0.974962	-1.725814
C	2.242701	1.605332	0.604340
H	0.539138	1.349022	-0.599072
N	1.572640	1.320578	-0.570575
C	1.571176	2.231296	1.686514
C	2.346989	2.502800	2.800498
C	4.234308	1.549215	1.947141
H	1.898125	3.021713	3.645787
C	-3.111143	-3.720567	0.166704
C	-2.224398	-4.157439	1.179176
C	-3.047039	-4.285292	-1.129859
C	-2.220777	-3.595836	2.497563
C	-1.273035	-5.208490	0.891306
C	-2.082310	-5.324948	-1.409695
C	-3.920516	-3.884067	-2.191354
C	-1.368241	-4.060431	3.464813
H	-2.894062	-2.775975	2.715872
C	-0.401475	-5.663175	1.930926
C	-1.228636	-5.762630	-0.391637
C	-2.032744	-5.894776	-2.720707
H	-4.656322	-3.111291	-1.999717
C	-3.839511	-4.453867	-3.434864
C	-0.450472	-5.113739	3.183650
H	-1.386217	-3.617616	4.456977
H	0.300496	-6.461669	1.701411
H	-0.515456	-6.557414	-0.602193
C	-2.882929	-5.473324	-3.707101
H	-1.302869	-6.677876	-2.913653
H	-4.511777	-4.127174	-4.223499
H	0.212340	-5.473161	3.966502
H	-2.836042	-5.915719	-4.698626
C	-3.003698	3.983190	-0.211399
C	-2.052509	4.367193	-1.186400
C	-2.996480	4.586369	1.068743
C	-1.988099	3.763479	-2.484388
C	-1.092170	5.404167	-0.877491
C	-2.022876	5.611500	1.369725
C	-3.934686	4.237190	2.092587
C	-1.067152	4.172436	-3.413335
H	-2.673042	2.957531	-2.718839
C	-0.149311	5.801093	-1.877741
C	-1.105817	5.998302	0.387763
C	-2.026501	6.216348	2.665945
H	-4.678808	3.476858	1.883421
C	-3.903769	4.839559	3.323009
C	-0.137831	5.209863	-3.112111
H	-1.038916	3.697260	-4.390233
H	0.560516	6.587699	-1.632207
H	-0.384333	6.781067	0.614229
C	-2.936222	5.842175	3.618104
H	-1.287323	6.985942	2.876572
H	-4.624029	4.551293	4.083860

H	0.582239	5.522095	-3.863742
H	-2.927862	6.309654	4.599094
C	5.787443	1.086854	-0.601578
C	6.029758	2.456170	-0.751876
C	6.894781	0.212246	-0.620481
C	7.314346	2.975917	-0.885155
H	5.182337	3.134339	-0.763264
C	8.190809	0.716577	-0.740024
C	8.403131	2.098798	-0.868008
H	7.471061	4.042712	-0.999914
H	9.045910	0.053127	-0.739806
C	1.336816	0.982746	-2.953628
H	1.675023	0.253335	-3.690068
H	0.297115	0.792156	-2.679126
H	1.395832	1.967459	-3.428790
C	0.111964	2.594410	1.659600
H	-0.189665	3.047090	0.710880
H	-0.518453	1.714500	1.823061
H	-0.110854	3.313948	2.452289
C	9.742571	2.597110	-0.985662
N	10.831679	2.996400	-1.076526
N	3.639252	2.161199	2.952293
O	5.502312	1.118201	2.112112
O	6.628352	-1.118042	-0.535191
C	7.693955	-2.052714	-0.648369
H	8.388416	-1.970293	0.197103
H	7.219495	-3.033975	-0.646907
H	8.244562	-1.916817	-1.587453
C	6.133992	1.424969	3.363205
H	5.600947	0.948233	4.189631
H	7.146489	1.028680	3.269792
H	6.161088	2.504860	3.530937
C	4.253594	0.562195	-3.056486
N	5.144918	-0.456771	-3.187548
H	5.582793	-0.553716	-4.093582
H	5.221975	-1.226947	-2.531468
O	4.017861	1.346098	-3.972419

S-forming TS Conformation 15*

B3LYP/6-31G(d) Energy = -4490.494472
M06-2X/6-311G(d,p)-SMD(tetrahydrofuran) Energy = -4490.003382
M06-2X/6-311G(d,p)-SMD(tetrahydrofuran)-derived Free Energy (Quasiharmonic) = -4488.904625

Frequencies (Top 3 out of 468)

1. -1181.5923 cm^{-1}
2. 7.6408 cm^{-1}
3. 10.0636 cm^{-1}

B3LYP/6-31G(d) Molecular Geometry in Cartesian Coordinates

N	1.044761	-1.822480	0.929682
C	1.374521	-2.326836	-0.297334

C	2.709914	-2.594075	-0.552807
C	3.232446	-2.031751	1.802440
C	3.709971	-2.250415	0.449513
H	4.201410	-1.008948	0.036712
H	0.063634	-1.485710	1.071286
C	3.248229	-3.191035	-1.785942
O	2.331061	-3.609267	-2.676041
O	4.454762	-3.299779	-1.990791
C	4.175525	-2.121582	2.942004
O	5.406526	-2.518881	2.531442
O	3.914288	-1.906196	4.111408
C	2.853016	-4.152271	-3.902518
H	1.977242	-4.448630	-4.479695
H	3.428157	-3.392624	-4.437753
H	3.493670	-5.013450	-3.698783
C	6.374351	-2.698297	3.576107
H	6.032112	-3.452820	4.289349
H	7.284929	-3.028298	3.075385
H	6.547009	-1.759943	4.107659
C	0.218261	-2.552088	-1.228258
H	-0.724066	-2.305125	-0.743266
H	0.318000	-1.946531	-2.132573
H	0.184193	-3.597295	-1.540571
C	1.239555	-1.394689	3.310753
H	0.218430	-1.046586	3.140653
H	1.209366	-2.278803	3.957063
H	1.816536	-0.639070	3.844714
P	-1.994107	0.112302	0.116342
O	-1.412716	-0.619652	1.302808
O	-1.084740	0.706036	-0.923751
O	-3.059556	-0.881852	-0.689148
O	-3.029305	1.249886	0.736332
H	4.644346	-2.793179	0.354848
C	-4.008115	1.779507	-0.085743
C	-5.108018	0.995413	-0.410171
C	-4.863319	3.644449	-1.338027
C	-3.880626	3.133298	-0.514150
H	-4.789997	4.677665	-1.667779
C	-5.229552	-0.372788	0.168767
C	-4.202038	-1.287864	-0.022709
C	-4.305135	-2.648646	0.389668
C	-5.440727	-3.033398	1.073510
H	-5.542457	-4.069002	1.388058
C	-6.916655	3.391749	-2.691345
C	-7.953413	2.615912	-3.154322
C	-8.064015	1.269724	-2.732448
C	-7.157157	0.733102	-1.845481
C	-6.083356	1.512179	-1.330129
C	-5.955677	2.863052	-1.787482
H	-6.807496	4.423062	-3.018586
H	-8.679621	3.027864	-3.849654
H	-8.870664	0.650646	-3.116019
H	-7.253039	-0.302353	-1.540245
C	-8.468266	-0.271761	2.092153
C	-7.396167	0.145353	1.334825

C	-6.371555	-0.763497	0.948115
C	-6.473316	-2.119432	1.396968
C	-7.600016	-2.521933	2.164028
C	-8.581532	-1.620796	2.504279
H	-9.233043	0.444451	2.380515
H	-7.321066	1.184333	1.035697
H	-7.663755	-3.558513	2.486643
H	-9.435940	-1.937051	3.096327
C	1.894003	-1.742524	2.003725
C	3.730056	0.998061	0.819111
C	4.380750	0.317895	-0.327791
C	3.651333	0.452551	-1.593909
C	2.313158	0.790479	-1.601865
C	2.368202	1.366090	0.737805
H	0.652973	1.197755	-0.470169
N	1.683947	1.119941	-0.437163
C	1.720555	2.012536	1.823187
C	2.508020	2.261049	2.933244
C	4.369982	1.270220	2.062954
H	2.076417	2.786086	3.783884
C	-3.268336	-3.681715	0.071598
C	-2.430993	-4.181812	1.097073
C	-3.210715	-4.236534	-1.229643
C	-2.420302	-3.633166	2.420894
C	-1.539431	-5.286033	0.817097
C	-2.307001	-5.331999	-1.500337
C	-4.033725	-3.770598	-2.304890
C	-1.614328	-4.155202	3.398922
H	-3.048456	-2.777160	2.634975
C	-0.716684	-5.800692	1.868334
C	-1.504311	-5.831679	-0.469571
C	-2.265752	-5.892088	-2.815963
H	-4.722915	-2.954298	-2.120324
C	-3.961763	-4.332608	-3.552588
C	-0.755395	-5.258675	3.124727
H	-1.625279	-3.720778	4.394909
H	-0.059849	-6.638312	1.644723
H	-0.840091	-6.669794	-0.672783
C	-3.066188	-5.408340	-3.815455
H	-1.583300	-6.718445	-3.002024
H	-4.594717	-3.956467	-4.351667
H	-0.129527	-5.663080	3.915961
H	-3.026339	-5.844046	-4.810240
C	-2.760738	4.028422	-0.082677
C	-1.766299	4.409344	-1.014984
C	-2.763638	4.578081	1.221479
C	-1.689445	3.855129	-2.334207
C	-0.772715	5.390938	-0.638210
C	-1.755382	5.545743	1.590847
C	-3.745094	4.230531	2.204490
C	-0.727332	4.262315	-3.221323
H	-2.399178	3.087817	-2.619072
C	0.212428	5.788561	-1.596522
C	-0.796051	5.932487	0.650166
C	-1.768881	6.096578	2.910845

H	-4.515985	3.513954	1.944269
C	-3.722595	4.780272	3.459475
C	0.233909	5.247959	-2.853720
H	-0.691401	3.825937	-4.215962
H	0.946094	6.534674	-1.299858
H	-0.048550	6.673051	0.928233
C	-2.720566	5.725135	3.822107
H	-1.002792	6.822778	3.173039
H	-4.476248	4.494096	4.188081
H	0.985663	5.560803	-3.573488
H	-2.719265	6.151081	4.821860
C	5.890775	0.451705	-0.472078
C	6.776641	-0.612242	-0.313438
C	6.408210	1.710904	-0.861789
C	8.142964	-0.469319	-0.543714
H	6.396836	-1.577544	-0.002759
C	7.772977	1.866149	-1.110589
C	8.641177	0.774257	-0.950240
H	8.815699	-1.311167	-0.421374
H	8.174491	2.822429	-1.421773
C	1.468542	0.890343	-2.843306
H	1.823929	0.212403	-3.621270
H	0.422629	0.679058	-2.607116
H	1.528239	1.909584	-3.240774
C	0.274400	2.425731	1.797741
H	-0.001701	2.920367	0.861993
H	-0.390940	1.565592	1.925563
H	0.069831	3.126334	2.612067
C	10.042816	0.941924	-1.203833
N	11.180593	1.076279	-1.407158
N	3.795740	1.898155	3.070998
O	5.643347	0.847323	2.213621
O	5.508430	2.721642	-0.962551
C	5.847438	3.869733	-1.734470
H	6.157989	3.578411	-2.743558
H	4.933488	4.461886	-1.793515
H	6.632685	4.464057	-1.250405
C	6.293468	1.173566	3.448489
H	5.770867	0.717321	4.293590
H	7.302618	0.770044	3.349080
H	6.327750	2.256176	3.597715
C	4.369875	0.424465	-2.927993
N	5.013393	-0.720003	-3.273090
H	5.574724	-0.695654	-4.113850
H	5.046808	-1.549647	-2.690230
O	4.351725	1.411463	-3.660394

S-forming TS Conformation 16

B3LYP/6-31G(d) Energy = -4490.491618
M06-2X/6-311G(d,p)-SMD(tetrahydrofuran) Energy = -4490.002695
M06-2X/6-311G(d,p)-SMD(tetrahydrofuran)-derived Free Energy (Quasiharmonic) = -4488.903616

Frequencies (Top 3 out of 468)

1. -1209.8389 cm^{-1}
2. 7.3740 cm^{-1}
3. 9.7109 cm^{-1}

B3LYP/6-31G(d) Molecular Geometry in Cartesian Coordinates

N	1.148008	-1.631219	0.875686
C	1.991040	-1.488976	1.949134
C	3.340290	-1.721074	1.749052
C	2.839659	-2.390291	-0.583940
C	3.827143	-1.948785	0.396182
H	4.275357	-0.746083	-0.061925
H	0.154651	-1.339112	1.013488
C	4.371705	-1.809535	2.811944
O	3.884068	-1.693290	4.069379
O	5.551189	-2.011336	2.583783
C	3.389126	-3.074395	-1.772158
O	2.474301	-3.436190	-2.694399
O	4.584159	-3.297982	-1.919260
C	4.848897	-1.860393	5.120102
H	5.629122	-1.098177	5.054703
H	4.287252	-1.754590	6.048671
H	5.313248	-2.848229	5.060148
C	2.999431	-4.075199	-3.870598
H	3.672610	-3.396863	-4.400633
H	3.542185	-4.985541	-3.604512
H	2.128803	-4.309105	-4.483481
C	1.297438	-1.135847	3.237638
H	1.239070	-2.019423	3.882426
H	1.849818	-0.371109	3.783682
H	0.281815	-0.789957	3.033249
C	0.338757	-2.484525	-1.239528
H	-0.608336	-2.260083	-0.751429
H	0.400150	-1.914869	-2.169711
H	0.347032	-3.542112	-1.509448
P	-2.009022	0.142391	0.094951
O	-1.367820	-0.530836	1.284796
O	-1.151432	0.757444	-0.976309
O	-3.038114	-0.921327	-0.666918
O	-3.087620	1.243132	0.707845
H	4.792569	-2.438024	0.335540
C	-4.098872	1.708980	-0.114072
C	-5.166368	0.868835	-0.404388
C	-5.050466	3.500911	-1.402498
C	-4.035905	3.055049	-0.579328
H	-5.026419	4.527092	-1.760283
C	-5.218107	-0.488867	0.208661
C	-4.152514	-1.361163	0.024785
C	-4.188940	-2.714898	0.470346
C	-5.297593	-3.133866	1.177965
H	-5.348580	-4.165224	1.517784
C	-7.107829	3.123635	-2.720192
C	-8.114931	2.291318	-3.149282
C	-8.160430	0.952828	-2.692342

C	-7.219623	0.479560	-1.804672
C	-6.175195	1.318188	-1.323589
C	-6.113114	2.661133	-1.816520
H	-7.048456	4.149911	-3.074908
H	-8.867249	2.652898	-3.844867
H	-8.943451	0.289155	-3.049390
H	-7.265484	-0.551051	-1.472596
C	-8.433623	-0.487189	2.173054
C	-7.390867	-0.040613	1.391936
C	-6.331303	-0.911695	1.012434
C	-6.366383	-2.259796	1.493819
C	-7.464273	-2.694039	2.284984
C	-8.481043	-1.829880	2.617543
H	-9.226227	0.200741	2.455071
H	-7.366259	0.993345	1.067988
H	-7.477369	-3.724580	2.632085
H	-9.313069	-2.169967	3.227988
C	1.495340	-2.169099	-0.333830
C	3.683568	1.276153	0.611226
C	4.351046	0.606992	-0.529098
C	3.575718	0.599512	-1.770554
C	2.218771	0.884453	-1.757804
C	2.308429	1.586481	0.550891
H	0.576975	1.316859	-0.612491
N	1.611621	1.275085	-0.601468
C	1.662371	2.236614	1.635150
C	2.461672	2.525922	2.728058
C	4.332988	1.565222	1.847680
H	2.028945	3.057226	3.574263
C	-3.110454	-3.708043	0.163865
C	-3.042310	-4.291167	-1.124367
C	-2.241940	-4.147077	1.191327
C	-3.895334	-3.886632	-2.201058
C	-2.095837	-5.353915	-1.378845
C	-1.307973	-5.219845	0.928228
C	-2.241276	-3.566383	2.501363
C	-3.812067	-4.475145	-3.435734
H	-4.616467	-3.095717	-2.028434
C	-2.044194	-5.943564	-2.681101
C	-1.262730	-5.794629	-0.345348
C	-0.455476	-5.675367	1.982981
H	-2.901795	-2.731444	2.700913
C	-1.406500	-4.032219	3.483635
C	-2.874374	-5.518216	-3.682846
H	-4.468149	-4.145302	-4.236591
H	-1.328996	-6.744413	-2.855188
H	-0.566104	-6.609089	-0.535758
C	-0.505845	-5.106280	3.226949
H	0.233463	-6.490253	1.772459
H	-1.427355	-3.575423	4.469513
H	-2.825804	-5.975492	-4.667488
H	0.142378	-5.467110	4.021340
C	-2.950241	4.009234	-0.188544
C	-1.983536	4.403603	-1.144190
C	-2.962355	4.597647	1.098487

C	-1.896214	3.811658	-2.446299
C	-1.029272	5.438140	-0.809497
C	-1.993490	5.619265	1.425787
C	-3.916680	4.238141	2.103707
C	-0.960045	4.229939	-3.355701
H	-2.574976	3.006089	-2.699054
C	-0.070938	5.846106	-1.790446
C	-1.062562	6.018173	0.461879
C	-2.016971	6.209694	2.728463
H	-4.658146	3.480958	1.874718
C	-3.904833	4.827077	3.340951
C	-0.037883	5.266162	-3.029698
H	-0.914092	3.763120	-4.335965
H	0.633674	6.631166	-1.525673
H	-0.345402	6.799049	0.707810
C	-2.941550	5.825724	3.662176
H	-1.281108	6.976844	2.958780
H	-4.637442	4.531270	4.087001
H	0.694387	5.585886	-3.766226
H	-2.948506	6.282692	4.648142
C	5.826146	0.973351	-0.706092
C	6.106239	2.328384	-0.908251
C	6.907439	0.066971	-0.687565
C	7.406798	2.806098	-1.044315
H	5.278405	3.029419	-0.949111
C	8.219217	0.530127	-0.800134
C	8.470758	1.900896	-0.973415
H	7.595711	3.863035	-1.196165
H	9.054409	-0.157306	-0.763549
C	1.321158	0.852877	-2.965703
H	1.645037	0.099274	-3.683999
H	0.287982	0.673099	-2.660383
H	1.368987	1.820845	-3.475512
C	0.203975	2.605336	1.631425
H	-0.110892	3.054793	0.685584
H	-0.429967	1.730297	1.808058
H	-0.002292	3.329718	2.424242
C	9.824601	2.360285	-1.085133
N	10.924444	2.730041	-1.171797
N	3.758908	2.192307	2.857803
O	5.607481	1.150772	1.981725
O	6.594926	-1.251061	-0.589330
C	7.633883	-2.214461	-0.446635
H	8.308857	-2.203956	-1.311438
H	8.202254	-2.042698	0.474174
H	7.127365	-3.177934	-0.396519
C	6.291770	1.491173	3.191124
H	5.762180	1.100389	4.063729
H	7.275176	1.029532	3.097555
H	6.382232	2.576549	3.293504
C	4.233405	0.404311	-3.115951
N	5.121542	-0.619660	-3.227878
H	5.594064	-0.717751	-4.115803
H	5.299404	-1.306365	-2.504193
O	3.970784	1.142770	-4.061885

S-forming TS Conformation 17

B3LYP/6-31G(d) Energy = -4490.494472
M06-2X/6-311G(d,p)-SMD(tetrahydrofuran) Energy = -4490.00338
M06-2X/6-311G(d,p)-SMD(tetrahydrofuran)-derived Free Energy (Quasiharmonic) = -4488.904623

Frequencies (Top 3 out of 468)

1. -1181.5770 cm^{-1}
2. 7.6410 cm^{-1}
3. 10.0647 cm^{-1}

B3LYP/6-31G(d) Molecular Geometry in Cartesian Coordinates

N	1.044727	-1.822604	0.929595
C	1.374467	-2.326902	-0.297449
C	2.709859	-2.594110	-0.552962
C	3.232429	-2.031908	1.802305
C	3.709932	-2.250498	0.449359
H	4.201370	-1.009000	0.036625
H	0.063607	-1.485827	1.071229
C	3.248154	-3.191000	-1.786139
O	2.330972	-3.609251	-2.676214
O	4.454685	-3.299676	-1.991039
C	4.175535	-2.121790	2.941844
O	5.406520	-2.519084	2.531233
O	3.914327	-1.906442	4.111262
C	2.852907	-4.152201	-3.902724
H	3.493614	-5.013351	-3.699032
H	1.977126	-4.448593	-4.479873
H	3.427989	-3.392513	-4.437966
C	6.374380	-2.698545	3.575858
H	6.032170	-3.453110	4.289070
H	7.284946	-3.028511	3.075091
H	6.547045	-1.760218	4.107455
C	0.218187	-2.552153	-1.228349
H	0.184120	-3.597360	-1.540665
H	-0.724130	-2.305203	-0.743330
H	0.317890	-1.946594	-2.132667
C	1.239555	-1.394954	3.310687
H	0.218395	-1.046935	3.140633
H	1.209469	-2.279082	3.956979
H	1.816489	-0.639290	3.844638
P	-1.994095	0.112268	0.116321
O	-1.412705	-0.619705	1.302777
O	-1.084727	0.705986	-0.923781
O	-3.029257	1.249877	0.736325
O	-3.059579	-0.881853	-0.689161
H	4.644307	-2.793254	0.354652
C	-4.202085	-1.287803	-0.022729
C	-5.229566	-0.372682	0.168703
C	-5.440862	-3.033262	1.073508
C	-4.305240	-2.648569	0.389683

H	-5.542638	-4.068853	1.388081
C	-5.107946	0.995508	-0.410243
C	-4.008020	1.779553	-0.085775
C	-3.880457	3.133340	-0.514177
C	-4.863088	3.644525	-1.338107
H	-4.789708	4.677736	-1.667860
C	-7.600166	-2.521692	2.163946
C	-8.581665	-1.620513	2.504138
C	-8.468343	-0.271495	2.091974
C	-7.396205	0.145563	1.334670
C	-6.371608	-0.763330	0.948023
C	-6.473428	-2.119250	1.396910
H	-7.663949	-3.558260	2.486588
H	-9.436102	-1.936723	3.096168
H	-9.233108	0.444750	2.380287
H	-7.321063	1.184532	1.035511
C	-8.063818	1.269926	-2.732665
C	-7.157032	0.733275	-1.845642
C	-6.083220	1.512310	-1.330249
C	-5.955456	2.863171	-1.787613
C	-6.916362	3.391900	-2.691534
C	-7.953130	2.616103	-3.154555
H	-8.870477	0.650881	-3.116269
H	-7.252980	-0.302170	-1.540395
H	-6.807138	4.423202	-3.018784
H	-8.679282	3.028077	-3.849932
C	1.893988	-1.742703	2.003629
C	3.730051	0.997946	0.819172
C	4.380739	0.317854	-0.327776
C	3.651331	0.452621	-1.593886
C	2.313158	0.790546	-1.601821
C	2.368199	1.365990	0.737890
H	0.652968	1.197721	-0.470094
N	1.683943	1.119925	-0.437096
C	1.720558	2.012372	1.823315
C	2.508028	2.260802	2.933388
C	4.369984	1.270024	2.063029
H	2.076430	2.785786	3.784063
C	-2.760574	4.028439	-0.082636
C	-1.766078	4.409355	-1.014884
C	-2.763547	4.578099	1.221520
C	-1.689150	3.855137	-2.334101
C	-0.772513	5.390947	-0.638054
C	-1.755305	5.545752	1.590948
C	-3.745065	4.230560	2.204473
C	-0.726987	4.262323	-3.221163
H	-2.398861	3.087816	-2.618999
C	0.212684	5.788568	-1.596312
C	-0.795919	5.932492	0.650322
C	-1.768875	6.096586	2.910945
H	-4.515949	3.513992	1.944206
C	-3.722635	4.780300	3.459459
C	0.234234	5.247967	-2.853508
H	-0.691000	3.825943	-4.215800
H	0.946337	6.534677	-1.299605

H	-0.048431	6.673053	0.928433
C	-2.720617	5.725152	3.822151
H	-1.002794	6.822777	3.173185
H	-4.476335	4.494133	4.188020
H	0.986028	5.560809	-3.573235
H	-2.719371	6.151097	4.821904
C	-3.268470	-3.681682	0.071659
C	-2.431172	-4.181792	1.097164
C	-3.210843	-4.236532	-1.229569
C	-2.420491	-3.633125	2.420976
C	-1.539647	-5.286055	0.817230
C	-2.307170	-5.332042	-1.500219
C	-4.033811	-3.770587	-2.304844
C	-1.614562	-4.155175	3.399033
H	-3.048620	-2.777092	2.635029
C	-0.716947	-5.800729	1.868497
C	-1.504521	-5.831731	-0.469425
C	-2.265917	-5.892163	-2.815831
H	-4.722970	-2.954254	-2.120311
C	-3.961847	-4.332629	-3.552528
C	-0.755668	-5.258688	3.124880
H	-1.625522	-3.720733	4.395012
H	-0.060141	-6.638379	1.644916
H	-0.840332	-6.669877	-0.672604
C	-3.066311	-5.408404	-3.815351
H	-1.583495	-6.718553	-3.001858
H	-4.594768	-3.956479	-4.351629
H	-0.129837	-5.663105	3.916136
H	-3.026459	-5.844135	-4.810125
C	5.890763	0.451652	-0.472068
C	6.776603	-0.612350	-0.313658
C	6.408217	1.710904	-0.861585
C	8.142922	-0.469424	-0.543961
H	6.396781	-1.577703	-0.003161
C	7.772978	1.866155	-1.110411
C	8.641154	0.774210	-0.950284
H	8.815638	-1.311315	-0.421802
H	8.174506	2.822476	-1.421449
C	1.468566	0.890505	-2.843270
H	1.528265	1.909780	-3.240652
H	1.823977	0.212627	-3.621278
H	0.422650	0.679193	-2.607125
C	0.274412	2.425600	1.797891
H	-0.001669	2.920309	0.862176
H	-0.390956	1.565472	1.925652
H	0.069857	3.126155	2.612262
C	10.042789	0.941882	-1.203901
N	11.180562	1.076240	-1.407245
N	3.795747	1.897894	3.071115
O	5.643351	0.847116	2.213661
O	5.508459	2.721680	-0.962141
C	5.847464	3.869895	-1.733879
H	6.632765	4.464103	-1.249759
H	6.157939	3.578738	-2.743038
H	4.933534	4.462095	-1.792759

C	6.293485	1.173305	3.448537
H	7.302634	0.769787	3.349103
H	6.327770	2.255909	3.597807
H	5.770893	0.717026	4.293624
C	4.369898	0.424667	-2.927962
N	5.013248	-0.719837	-3.273256
H	5.046621	-1.549548	-2.690488
H	5.574629	-0.695404	-4.113981
O	4.351894	1.411798	-3.660187

S-forming TS Conformation 18

B3LYP/6-31G(d) Energy = -4490.492185
M06-2X/6-311G(d,p)-SMD(tetrahydrofuran) Energy = -4490.004559
M06-2X/6-311G(d,p)-SMD(tetrahydrofuran)-derived Free Energy (Quasiharmonic) = -4488.905105

Frequencies (Top 3 out of 468)

1. -1205.9031 cm^{-1}
2. 7.4605 cm^{-1}
3. 10.8157 cm^{-1}

B3LYP/6-31G(d) Molecular Geometry in Cartesian Coordinates

N	1.145366	-1.610991	0.907863
C	1.485266	-2.140970	-0.306788
C	2.828748	-2.355470	-0.570561
C	3.346873	-1.704251	1.762230
C	3.826785	-1.922702	0.405895
H	4.259720	-0.711844	-0.038484
H	0.151316	-1.323887	1.054839
C	3.369573	-3.016269	-1.773397
O	2.447521	-3.406879	-2.675452
O	4.569136	-3.198829	-1.952363
C	4.291391	-1.760445	2.903096
O	5.502981	-2.237988	2.522826
O	4.041973	-1.464429	4.058383
C	2.964735	-4.024834	-3.866269
H	2.087607	-4.283337	-4.459595
H	3.604298	-3.324677	-4.409539
H	3.541130	-4.919036	-3.616758
C	6.454559	-2.403872	3.582888
H	7.339480	-2.836720	3.114797
H	6.696728	-1.442356	4.041815
H	6.061226	-3.073436	4.352428
C	0.323558	-2.456678	-1.205435
H	-0.620301	-2.244348	-0.706518
H	0.371500	-1.877116	-2.130445
H	0.337780	-3.511534	-1.485123
C	1.339619	-1.128735	3.278386
H	1.873493	-0.323994	3.784599
H	0.296441	-0.850658	3.113420
H	1.373330	-1.994830	3.948490
P	-2.025915	0.133993	0.111970

O	-1.378975	-0.529047	1.304091
O	-1.173749	0.754000	-0.961434
O	-3.044397	-0.941742	-0.647821
O	-3.115665	1.226602	0.719732
H	4.788527	-2.413108	0.318091
C	-4.131716	1.679122	-0.103600
C	-5.190767	0.827216	-0.390556
C	-5.102232	3.456968	-1.397546
C	-4.082763	3.024217	-0.573453
H	-5.088875	4.482179	-1.758662
C	-5.228528	-0.528887	0.226959
C	-4.154343	-1.391006	0.045207
C	-4.177416	-2.743870	0.494349
C	-5.281389	-3.171649	1.203948
H	-5.321988	-4.202475	1.546765
C	-7.156304	3.054302	-2.712892
C	-8.155026	2.210285	-3.138716
C	-8.186547	0.872909	-2.677350
C	-7.240505	0.412268	-1.788602
C	-6.204503	1.263159	-1.310807
C	-6.156447	2.605015	-1.808228
H	-7.107674	4.079984	-3.070960
H	-8.911404	2.561854	-3.835037
H	-8.962921	0.200065	-3.031754
H	-7.275667	-0.617639	-1.453049
C	-8.442673	-0.553084	2.193418
C	-7.404977	-0.098608	1.410121
C	-6.337070	-0.960300	1.032598
C	-6.358458	-2.307186	1.518131
C	-7.451367	-2.749835	2.311504
C	-8.476408	-1.894768	2.642158
H	-9.241796	0.127851	2.474011
H	-7.390794	0.934584	1.083102
H	-7.453878	-3.779309	2.661978
H	-9.304463	-2.241134	3.254466
C	1.999723	-1.469266	1.971532
C	3.632635	1.294191	0.667744
C	4.319578	0.653368	-0.477681
C	3.557207	0.661800	-1.726715
C	2.196717	0.932361	-1.723176
C	2.257957	1.607158	0.592943
H	0.539467	1.334198	-0.589004
N	1.574971	1.304250	-0.569876
C	1.598592	2.251765	1.671878
C	2.385548	2.537064	2.774612
C	4.261818	1.564212	1.918426
H	1.946055	3.069679	3.616317
C	-3.089854	-3.727557	0.189251
C	-2.212964	-4.152109	1.215625
C	-3.020237	-4.316127	-1.096410
C	-2.213466	-3.564529	2.522616
C	-1.268031	-5.215636	0.954051
C	-2.063442	-5.369959	-1.349287
C	-3.881237	-3.925651	-2.171956
C	-1.368222	-4.014550	3.503234

H	-2.882725	-2.736249	2.720722
C	-0.405496	-5.655336	2.007378
C	-1.221589	-5.796322	-0.316822
C	-2.010551	-5.965435	-2.648854
H	-4.609917	-3.141435	-2.000373
C	-3.796378	-4.519210	-3.404127
C	-0.456211	-5.079341	3.248137
H	-1.388361	-3.551725	4.486192
H	0.291773	-6.463501	1.798200
H	-0.516771	-6.604044	-0.505934
C	-2.848833	-5.553736	-3.649592
H	-1.287838	-6.759837	-2.821634
H	-4.458839	-4.200145	-4.204113
H	0.200549	-5.426953	4.041344
H	-2.799320	-6.015549	-4.632094
C	-3.006694	3.990756	-0.186512
C	-2.044249	4.391283	-1.143876
C	-3.024332	4.583666	1.098381
C	-1.951856	3.796072	-2.444142
C	-1.099951	5.436154	-0.813080
C	-2.065458	5.615910	1.421752
C	-3.974629	4.218163	2.105237
C	-1.020371	4.220608	-3.355479
H	-2.623101	2.983323	-2.694016
C	-0.146002	5.850096	-1.795793
C	-1.138592	6.020332	0.456239
C	-2.094200	6.210549	2.722397
H	-4.708563	3.452783	1.879285
C	-3.967961	4.811333	3.340483
C	-0.107982	5.266653	-3.033277
H	-0.970750	3.751417	-4.334452
H	0.551109	6.642783	-1.533895
H	-0.428947	6.808938	0.699284
C	-3.014452	5.820526	3.657863
H	-1.365705	6.985525	2.949874
H	-4.696991	4.510580	4.088036
H	0.620738	5.591203	-3.771223
H	-3.025234	6.280411	4.642416
C	5.792212	1.039075	-0.633091
C	6.059419	2.403584	-0.784758
C	6.882497	0.143600	-0.663568
C	7.352865	2.898359	-0.926559
H	5.225133	3.097942	-0.787839
C	8.187346	0.622428	-0.789761
C	8.425041	2.000672	-0.916513
H	7.529466	3.962077	-1.041107
H	9.029355	-0.057569	-0.796839
C	1.311670	0.905328	-2.940555
H	1.651915	0.165060	-3.665356
H	0.278160	0.707064	-2.647698
H	1.350396	1.880845	-3.436452
C	0.140478	2.620462	1.654310
H	-0.492869	1.745992	1.835371
H	-0.071205	3.351289	2.439722
H	-0.168520	3.061408	0.702644

C	9.772949	2.473742	-1.041358
N	10.868840	2.852496	-1.138223
N	3.677729	2.192872	2.919966
O	5.529853	1.131137	2.079419
O	6.588779	-1.182224	-0.586635
C	7.636893	-2.139335	-0.679970
H	8.201304	-2.022328	-1.613456
H	8.322528	-2.062862	0.173042
H	7.141439	-3.110116	-0.676161
C	6.172699	1.453666	3.320991
H	6.201101	2.535532	3.474988
H	5.647564	0.986751	4.157966
H	7.184440	1.056398	3.223294
C	4.227769	0.508520	-3.071663
N	5.113225	-0.515077	-3.209275
H	5.565413	-0.604793	-4.108758
H	5.234577	-1.255762	-2.528116
O	3.981032	1.283578	-3.991864

S-forming TS Conformation 19

B3LYP/6-31G(d) Energy = -4490.490688
M06-2X/6-311G(d,p)-SMD(tetrahydrofuran) Energy = -4489.996864
M06-2X/6-311G(d,p)-SMD(tetrahydrofuran)-derived Free Energy (Quasiharmonic) = -4488.898314

Frequencies (Top 3 out of 468)

1. -1196.1213 cm^{-1}
2. 7.1930 cm^{-1}
3. 10.7651 cm^{-1}

B3LYP/6-31G(d) Molecular Geometry in Cartesian Coordinates

N	1.051110	-1.783250	0.856501
C	1.461200	-2.306190	-0.342469
C	2.805850	-2.593020	-0.500759
C	3.183590	-1.919740	1.857301
C	3.736700	-2.179590	0.539731
H	4.194340	-0.948040	0.060811
H	0.053170	-1.482920	0.946831
C	3.342970	-3.258460	-1.706849
O	4.567440	-3.782820	-1.476289
O	2.809730	-3.333150	-2.807479
C	4.161900	-1.980890	2.970321
O	3.609410	-1.895830	4.201321
O	5.357800	-2.130250	2.793331
C	5.204920	-4.406240	-2.605259
H	6.134380	-4.822401	-2.216799
H	4.569780	-5.193200	-3.017809
H	5.411860	-3.663201	-3.379679
C	4.532280	-2.000300	5.297471
H	5.253750	-1.179800	5.273751
H	3.919770	-1.944400	6.197721
H	5.072260	-2.949580	5.254711

C	0.355940	-2.529100	-1.329989
H	0.721450	-2.997400	-2.239149
H	-0.424500	-3.150540	-0.881209
H	-0.107840	-1.566340	-1.578019
C	1.067650	-1.337120	3.235281
H	0.061170	-0.996010	2.985431
H	0.987010	-2.228380	3.867001
H	1.588690	-0.575660	3.815611
P	-2.025820	0.071410	0.059451
O	-1.450560	-0.662680	1.246651
O	-1.102490	0.661890	-0.971859
O	-3.052020	1.218360	0.677741
O	-3.093420	-0.902800	-0.759339
H	4.700150	-2.677080	0.559031
C	-4.238190	-1.310169	-0.097919
C	-5.262880	-0.393289	0.098011
C	-5.482300	-3.060789	0.982191
C	-4.343480	-2.673269	0.305381
H	-5.587000	-4.098219	1.289621
C	-5.134220	0.979211	-0.469199
C	-4.030470	1.755771	-0.138799
C	-3.898769	3.113260	-0.554049
C	-4.880149	3.636771	-1.371649
H	-4.803489	4.673061	-1.690849
C	-7.644350	-2.552869	2.068671
C	-8.624670	-1.652229	2.413781
C	-8.506670	-0.299979	2.013851
C	-7.431250	0.120801	1.263201
C	-6.408020	-0.787299	0.871331
C	-6.514220	-2.146829	1.308541
H	-7.711740	-3.592039	2.382091
H	-9.481750	-1.971499	3.000331
H	-9.270450	0.415721	2.306121
H	-7.352730	1.162121	0.973201
C	-8.088450	1.286711	-2.789619
C	-7.183510	0.738321	-1.907929
C	-6.107440	1.508761	-1.384329
C	-5.975400	2.863701	-1.828299
C	-6.934679	3.404661	-2.726699
C	-7.973770	2.636821	-3.197649
H	-8.896660	0.673911	-3.179899
H	-7.282460	-0.299989	-1.613649
H	-6.822179	4.438871	-3.043519
H	-8.698569	3.058031	-3.888889
C	1.826880	-1.674420	1.980481
C	3.758040	1.067900	0.760901
C	4.376050	0.359590	-0.380589
C	3.607530	0.445380	-1.631449
C	2.276600	0.798680	-1.616109
C	2.387940	1.404630	0.717741
H	0.648840	1.237150	-0.454439
N	1.676360	1.148690	-0.437719
C	1.755680	2.026190	1.827371
C	2.566450	2.280420	2.919191
C	4.432750	1.359460	1.982271

H	2.142450	2.778880	3.789691
C	-2.774229	3.998570	-0.114679
C	-1.782859	4.389590	-1.046109
C	-2.769599	4.530850	1.196751
C	-1.715519	3.855560	-2.374139
C	-0.784639	5.363120	-0.660629
C	-1.756739	5.490320	1.574721
C	-3.748369	4.173720	2.178991
C	-0.759489	4.275940	-3.261859
H	-2.429279	3.094770	-2.666529
C	0.195391	5.773280	-1.618909
C	-0.800139	5.886800	0.635291
C	-1.762919	6.023680	2.901901
H	-4.522839	3.463441	1.912361
C	-3.719009	4.706840	3.440971
C	0.206521	5.253650	-2.885119
H	-0.733119	3.856970	-4.264319
H	0.932631	6.513140	-1.315629
H	-0.049389	6.621530	0.919951
C	-2.712239	5.643400	3.812001
H	-0.993499	6.744030	3.170391
H	-4.471009	4.413951	4.168621
H	0.952931	5.578330	-3.605259
H	-2.705759	6.056450	4.817151
C	-3.302130	-3.700740	-0.013559
C	-2.484670	-4.221170	1.018041
C	-3.216160	-4.226620	-1.325289
C	-2.500540	-3.701190	2.353481
C	-1.585130	-5.317740	0.732331
C	-2.300620	-5.310600	-1.602129
C	-4.020050	-3.741409	-2.406429
C	-1.715040	-4.244500	3.336481
H	-3.133730	-2.850300	2.573251
C	-0.783960	-5.855100	1.789161
C	-1.518850	-5.831680	-0.566159
C	-2.224220	-5.836630	-2.929899
H	-4.719970	-2.935289	-2.217779
C	-3.916500	-4.272800	-3.665159
C	-0.849990	-5.341820	3.056291
H	-1.747610	-3.832040	4.341431
H	-0.121261	-6.686650	1.560381
H	-0.843411	-6.658900	-0.775989
C	-3.005620	-5.333860	-3.934799
H	-1.528541	-6.650270	-3.121699
H	-4.535030	-3.882219	-4.468619
H	-0.240830	-5.764070	3.851301
H	-2.937670	-5.742220	-4.939509
C	5.881480	0.434469	-0.580989
C	6.681530	-0.701161	-0.672399
C	6.476490	1.700439	-0.798799
C	8.046080	-0.619101	-0.949329
H	6.237010	-1.679251	-0.536359
C	7.837050	1.799079	-1.081939
C	8.624520	0.636929	-1.152559
H	8.653060	-1.515661	-1.009999

H	8.299630	2.762529	-1.254939
C	1.414780	0.956990	-2.840699
H	0.377590	0.696690	-2.615779
H	1.422520	2.010330	-3.148929
H	1.778650	0.354800	-3.673079
C	0.297990	2.399430	1.833841
H	-0.347740	1.516980	1.900101
H	0.075481	3.037440	2.693751
H	0.008021	2.951500	0.934211
C	10.025340	0.748959	-1.438949
N	11.162520	0.839219	-1.668719
N	3.870330	1.957470	3.016461
O	5.722440	0.985689	2.069601
O	5.651250	2.773339	-0.707219
C	6.059481	3.995529	-1.310349
H	6.332971	3.834439	-2.358769
H	5.192491	4.655830	-1.253549
H	6.895481	4.456269	-0.768459
C	6.425070	1.296679	3.275011
H	7.424000	0.882839	3.133761
H	6.471370	2.378579	3.430321
H	5.939330	0.838579	4.140511
C	4.315960	0.367350	-2.975269
N	4.129650	-0.766460	-3.706679
H	3.479040	-1.501450	-3.453139
H	4.522790	-0.776850	-4.639229
O	5.013490	1.292260	-3.374399

S-forming TS Conformation 20

B3LYP/6-31G(d) Energy = -4490.489028
M06-2X/6-311G(d,p)-SMD(tetrahydrofuran) Energy = -4489.999167
M06-2X/6-311G(d,p)-SMD(tetrahydrofuran)-derived Free Energy (Quasiharmonic) = -4488.902099

Frequencies (Top 3 out of 468)

1. -1119.5130 cm^{-1}
2. 5.5723 cm^{-1}
3. 7.2174 cm^{-1}

B3LYP/6-31G(d) Molecular Geometry in Cartesian Coordinates

N	1.243866	-0.520423	2.031026
C	1.884901	-1.719163	1.838682
C	3.262132	-1.752324	1.937306
C	3.227503	0.639947	2.576715
C	3.993408	-0.495997	2.056834
H	4.325393	-0.188018	0.796312
H	0.209517	-0.502718	1.878397
C	3.987753	-3.043875	1.898711
O	5.330478	-2.864265	1.996698
O	3.479191	-4.146358	1.839137
C	3.914838	1.772722	3.228446
O	5.262662	1.594777	3.248946

O	3.380634	2.748225	3.726563
C	6.106230	-4.064681	1.860280
H	7.144561	-3.759151	1.997055
H	5.818989	-4.797482	2.618267
H	5.955598	-4.489339	0.865142
C	5.996189	2.604844	3.954687
H	7.048015	2.351175	3.818257
H	5.778883	3.595989	3.549781
H	5.735481	2.595219	5.017082
C	0.982539	-2.887999	1.580110
H	0.959407	-3.554145	2.448287
H	-0.033218	-2.546515	1.374618
H	1.355292	-3.491662	0.751734
C	0.900696	1.706982	2.916016
H	0.953529	1.853554	3.999448
H	1.182894	2.661310	2.467904
H	-0.124519	1.448771	2.640270
P	-2.020907	0.019796	0.177268
O	-1.446771	-0.509189	1.465913
O	-1.148261	0.732459	-0.820502
O	-2.759315	-1.185038	-0.697563
O	-3.319671	0.943436	0.633332
H	4.998206	-0.580141	2.458940
C	-4.305478	1.294703	-0.264130
C	-5.155663	0.313692	-0.759841
C	-5.474771	3.020647	-1.464754
C	-4.487924	2.681392	-0.559151
H	-5.646574	4.070167	-1.688206
C	-5.098712	-1.077685	-0.227065
C	-3.911119	-1.798364	-0.251317
C	-3.856501	-3.189223	0.069969
C	-5.017663	-3.794762	0.509197
H	-5.002452	-4.856162	0.742137
C	-7.202865	2.419102	-3.127483
C	-7.941562	1.466398	-3.789382
C	-7.760869	0.098229	-3.476939
C	-6.870496	-0.289307	-2.499800
C	-6.100979	0.669802	-1.783847
C	-6.261304	2.049218	-2.129107
H	-7.317803	3.473940	-3.365953
H	-8.653528	1.758388	-4.556517
H	-8.327937	-0.655975	-4.016080
H	-6.740376	-1.342432	-2.279155
C	-8.577067	-1.615640	1.130225
C	-7.479986	-0.983797	0.587208
C	-6.272238	-1.693002	0.334433
C	-6.223976	-3.077801	0.692508
C	-7.378893	-3.706214	1.232343
C	-8.535783	-2.994216	1.445892
H	-9.482889	-1.046802	1.322460
H	-7.529854	0.074688	0.360835
H	-7.322977	-4.762206	1.486186
H	-9.411300	-3.481380	1.866297
C	1.844638	0.613911	2.497402
C	3.833285	1.587197	-0.468603

C	4.365596	0.222589	-0.599652
C	3.463642	-0.718704	-1.259287
C	2.123624	-0.436992	-1.420370
C	2.454383	1.827158	-0.663389
H	0.603687	0.896099	-1.027626
N	1.637122	0.780895	-1.043189
C	1.916139	3.134111	-0.521772
C	2.816681	4.125767	-0.179012
C	4.614651	2.722435	-0.096227
H	2.463940	5.149680	-0.069894
C	-2.626955	-4.026010	-0.087190
C	-2.066514	-4.659528	1.051070
C	-2.095504	-4.283239	-1.375756
C	-2.516050	-4.398296	2.387363
C	-0.979565	-5.599091	0.885003
C	-0.981050	-5.192655	-1.520609
C	-2.634427	-3.702809	-2.568987
C	-1.968613	-5.046007	3.464054
H	-3.292048	-3.658275	2.542196
C	-0.443473	-6.260705	2.034624
C	-0.463528	-5.834845	-0.391755
C	-0.444647	-5.440457	-2.823569
H	-3.487082	-3.038927	-2.492063
C	-2.095500	-3.969692	-3.800569
C	-0.926637	-6.001207	3.288650
H	-2.325643	-4.821787	4.465657
H	0.368787	-6.968285	1.887656
H	0.360737	-6.535333	-0.509712
C	-0.980125	-4.844936	-3.933625
H	0.396969	-6.123933	-2.910327
H	-2.525693	-3.511985	-4.687360
H	-0.509130	-6.509041	4.153886
H	-0.566703	-5.045388	-4.918646
C	-3.714004	3.773665	0.108105
C	-2.949892	4.672202	-0.679837
C	-3.842035	3.988608	1.504388
C	-2.749759	4.498972	-2.089350
C	-2.329139	5.820830	-0.057336
C	-3.177462	5.116119	2.118091
C	-4.640283	3.154038	2.351140
C	-2.044142	5.411313	-2.830573
H	-3.159502	3.618386	-2.569690
C	-1.604693	6.751414	-0.868255
C	-2.449245	6.004217	1.321908
C	-3.303121	5.322764	3.527714
H	-5.174960	2.318732	1.916074
C	-4.742400	3.389937	3.697038
C	-1.472746	6.562865	-2.217723
H	-1.911425	5.251465	-3.897393
H	-1.162353	7.618561	-0.382819
H	-1.973540	6.865604	1.786849
C	-4.058911	4.483478	4.299908
H	-2.786413	6.171207	3.970348
H	-5.352638	2.735239	4.313159
H	-0.925252	7.280883	-2.822611

H	-4.148070	4.651321	5.369842
C	5.828694	0.040214	-0.972767
C	6.735278	-0.654030	-0.174874
C	6.277669	0.541749	-2.219596
C	8.066760	-0.820962	-0.552130
H	6.393573	-1.090607	0.754408
C	7.607639	0.389396	-2.608318
C	8.507067	-0.288710	-1.767541
H	8.757397	-1.360028	0.087067
H	7.963803	0.780698	-3.552700
C	1.114768	-1.375199	-2.031820
H	0.113174	-1.162201	-1.652257
H	1.077645	-1.236433	-3.119519
H	1.363378	-2.419971	-1.840255
C	0.469278	3.450734	-0.778486
H	0.167271	3.177781	-1.796064
H	-0.207526	2.910448	-0.110978
H	0.291451	4.521512	-0.656183
C	9.874004	-0.437796	-2.174462
N	10.982806	-0.555397	-2.507124
N	4.134655	3.942336	0.032408
O	5.928487	2.516951	0.131455
O	5.338585	1.159574	-2.995129
C	5.755125	1.793803	-4.197957
H	6.512422	2.562960	-4.002921
H	6.152682	1.069154	-4.920421
H	4.860221	2.262662	-4.610237
C	6.734867	3.668367	0.399151
H	6.709029	4.366644	-0.442758
H	6.390456	4.191839	1.294246
H	7.744009	3.279159	0.542856
C	4.027985	-2.042371	-1.709908
N	3.979537	-2.255655	-3.058696
H	4.427908	-3.088280	-3.418619
H	3.811898	-1.488868	-3.693253
O	4.495325	-2.867127	-0.933811

10. References

1. Mohamadi, F. *et al.* Macromodel - an integrated software system for modeling organic and bioorganic molecules using molecular mechanics. *J. Comput. Chem.* **11,** 440–467 (1990).
2. Schrödinger Release 2019-2: MacroModel, Schrödinger, LLC, New York, NY, **2019**.
3. Harder, E. *et al.* OPLS3: A Force Field Providing Broad Coverage of Drug-like Small Molecules and Proteins. *J. Chem. Theory Comput.* **12,** 281–296 (2016).
4. Chang, G., Guida, W. C. & Still, W. C. An internal-coordinate Monte Carlo method for searching conformational space. *J. Am. Chem. Soc.* **111,** 4379–4386 (1989).
5. Kolossvary, I. & Guida, W. C. Low-mode conformational search elucidated: Application to C39H80 and flexible docking of 9-deazaguanine inhibitors into PNP. *J. Comput. Chem.* **20,** 1671–1684 (1999).
6. Gaussian 16, Revision A.03, Frisch, M. J.; Trucks, G. W.; Schlegel, H. B.; Scuseria, G. E.; Robb, M. A.; Cheeseman, J. R.; Scalmani, G.; Barone, V.; Petersson, G. A.; Nakatsuji, H.; Li, X.; Caricato, M.; Marenich, A. V.; Bloino, J.; Janesko, B. G.; Gomperts, R.; Mennucci, B.; Hratchian, H. P.; Ortiz, J. V.; Izmaylov, A. F.; Sonnenberg, J. L.; Williams-Young, D.; Ding, F.; Lipparini, F.; Egidi, F.; Goings, J.; Peng, B.; Petrone, A.; Henderson, T.; Ranasinghe, D.; Z\akrzewski, V. G.; Gao, J.; Rega, N.; Zheng, G.; Liang, W.; Hada, M.; Ehara, M.; Toyota, K.; Fukuda, R.; Hasegawa, J.; Ishida, M.; Nakajima, T.; Honda, Y.; Kitao, O.; Nakai, H.; Vreven, T.; Throssell, K.; Montgomery, J. A., Jr.; Peralta, J. E.; Ogliaro, F.; Bearpark, M. J.; Heyd, J. J.; Brothers, E. N.; Kudin, K. N.; Staroverov, V. N.; Keith, T. A.; Kobayashi, R.; Normand, J.; Raghavachari, K.; Rendell, A. P.; Burant, J. C.; Iyengar, S. S.; Tomasi, J.; Cossi, M.; Millam, J. M.; Klene, M.; Adamo, C.; Cammi, R.; Ochterski, J. W.; Martin, R. L.; Morokuma, K.; Farkas, O.; Foresman, J. B.; Fox, D. J. Gaussian, Inc., Wallingford CT, **2016**.
7. Becke, A. D. Density-functional thermochemistry. III. The role of exact exchange. *J. Chem. Phys.* **98,** 5648–5652 (1993).
8. Stephens, P. J., Devlin, F. J., Chabalowski, C. F. & Frisch, M. J. Ab Initio Calculation of Vibrational Absorption and Circular Dichroism Spectra Using Density Functional Force Fields. *J. Phys. Chem.* **98,** 11623–11627 (1994).
9. Hehre, W. J., Ditchfield, R. & Pople, J. A. Self—Consistent Molecular Orbital Methods. XII. Further Extensions of Gaussian—Type Basis Sets for Use in Molecular Orbital Studies of Organic Molecules. *J. Chem. Phys.* **56,** 2257–2261 (1972).
10. Simón, L. & Goodman, J. M. How reliable are DFT transition structures? Comparison of GGA, hybrid-meta-GGA and meta-GGA functionals. *Org. Biomol. Chem.* **9,** 689–700 (2011).
11. Zhao, Y. & Truhlar, D. G. The M06 suite of density functionals for main group thermochemistry, thermochemical kinetics, noncovalent interactions, excited states, and transition elements: two new functionals and systematic testing of four M06-class functionals and 12 other function. *Theor. Chem. Acc.* **120,** 215–241 (2008).
12. Krishnan, R., Binkley, J. S., Seeger, R. & Pople, J. A. Self-consistent molecular orbital methods. XX. A basis set for correlated wave functions. *J. Chem. Phys.* **72,** 650–654 (1980).
13. Marenich, A. V, Cramer, C. J. & Truhlar, D. G. Universal Solvation Model Based on Solute Electron Density and on a Continuum Model of the Solvent Defined by the Bulk Dielectric Constant and Atomic Surface Tensions. *J. Phys. Chem. B* **113,** 6378–6396 (2009).
14. Grimme, S. Supramolecular Binding Thermodynamics by Dispersion-Corrected Density. *Chem. - A Eur. J.* **18,** 9955–9964 (2012).
15. Funes-Ardoiz, I. & Paton, R. S. Goodvibes: version 2.0.3. **2018**. doi:10.5281/zenodo.595246
16. Alecu, I. M., Zheng, J., Zhao, Y. & Truhlar, D. G. Computational Thermochemistry : Scale Factor Databases and Scale Factors for Vibrational Frequencies Obtained from Electronic Model Chemistries. *J. Chem. Theory Comput.* **6,** 2872–2887 (2010).
17. Sedgwick, D. M., Grayson, M. N., Fustero, S. & Barrio, P. Recent Developments and Applications of the Chiral Bronsted Acid Catalyzed Allylboration of Carbonyl Compounds. *Synth.* **50,** 1935–1957 (2018).
18. Falcone, B. N., Grayson, M. N. & Rodriguez, J. B. Mechanistic Insights into a Chiral Phosphoric Acid-Catalyzed Asymmetric Pinacol Rearrangement. *J. Org. Chem.* **83,** 14683–14687 (2018).
19. Rodríguez-Guerra Pedregal, J., Gómez-Orellana, P. & Maréchal, J.-D. ESIgen: Electronic Supporting Information Generator for Computational Chemistry Publications. *J. Chem. Inf. Model.* **58,** 561–564 (2018).
